# Risk factors for herpes zoster infections: a systematic review and meta-analysis unveiling common trends and heterogeneity patterns

**DOI:** 10.1007/s15010-023-02156-y

**Published:** 2024-01-18

**Authors:** Maren Steinmann, David Lampe, John Grosser, Juliana Schmidt, Marla Louise Hohoff, Anita Fischer, Wolfgang Greiner

**Affiliations:** Department of Health Economics and Health Care Management, School of Public Health, Bielefeld University, Bielefeld, Germany

**Keywords:** Herpes zoster, Risk factors, Meta-analysis, Systematic review, Random-effects model, Meta-regression

## Abstract

**Purpose:**

The burden of herpes zoster (HZ) is substantial and numerous chronic underlying conditions are known as predisposing risk factors for HZ onset. Thus, a comprehensive study is needed to synthesize existing evidence. This study aims to comprehensively identify these risk factors.

**Methods:**

A systematic literature search was done using MEDLINE via PubMed, EMBASE and Web of Science for studies published from January 1, 2003 to January 1, 2023. A random-effects model was used to estimate pooled Odds Ratios (OR). Heterogeneity was assessed using the *I*^2^ statistic. For sensitivity analyses basic outlier removal, leave-one-out validation and Graphic Display of Heterogeneity (GOSH) plots with different algorithms were employed to further analyze heterogeneity patterns. Finally, a multiple meta-regression was conducted.

**Results:**

Of 6392 considered records, 80 were included in the meta-analysis. 21 different conditions were identified as potential risk factors for HZ: asthma, autoimmune disorders, cancer, cardiovascular disorders, chronic heart failure (CHF), chronic obstructive pulmonary disorder (COPD), depression, diabetes, digestive disorders, endocrine and metabolic disorders, hematological disorders, HIV, inflammatory bowel disease (IBD), mental health conditions, musculoskeletal disorders, neurological disorders, psoriasis, renal disorders, rheumatoid arthritis (RA), systemic lupus erythematosus (SLE) and transplantation. Transplantation was associated with the highest risk of HZ (OR = 4.51 (95% CI [1.9–10.7])). Other risk factors ranged from OR = 1.17–2.87, indicating an increased risk for all underlying conditions. Heterogeneity was substantial in all provided analyses. Sensitivity analyses showed comparable results regarding the pooled effects and heterogeneity.

**Conclusions:**

This study showed an increased risk of HZ infections for all identified factors.

**Supplementary Information:**

The online version contains supplementary material available at 10.1007/s15010-023-02156-y.

## Introduction

Herpes zoster (HZ) infection is caused by reactivation of varicella zoster virus (VZV), which has established latency in the sensory ganglia after primary infection with VZV [1]. HZ causes a painful unilateral blistering dermatomal rash. It is thought to cause nerve damage that can lead to postherpetic neuralgia (PHN). PHN is a dermatomal nerve pain that can continue for months or years [2]. HZ substantially impacts the Quality of Life as well as psychological and physical functioning aspects of patients’ lives [3, 4]. In addition to neurologic complications, ophthalmic, vascular, and visceral complications of HZ are also evident [5]. These complications lead to increased healthcare costs and an economic burden in older adults [6].

The incidence rate (IR) of HZ ranges between 3 and 5 per 1000 person-years (PY) in North America, Europe and Pacific Asia [7]. In Germany, the overall IR of HZ varied between 6.76 (95% CI 6.71–6.82) per 1000 PY in 2006 and 7.52 (95% confidence interval (CI) 7.47–7.58) per 1000 PY in 2012 [8]. During the last years the incidence of HZ infection was increasing [7]. Furthermore, the incidence of HZ is rising with age [9]. As a result, the number of HZ infections will increase in the upcoming years due to the demographic change in developed countries [10].

In addition to age, patients with immunocompromising conditions have an elevated risk of developing HZ. This includes both immunocompromising diseases and immunosuppressive medications [11, 12]. A recent German claims data analysis showed, that patients suffering from asthma, chronic heart disease, chronic obstructive pulmonary disorder (COPD), depression or rheumatoid arthritis (RA) on average had a 30% higher chance of developing acute HZ compared to those without any underlying condition. Among these conditions, RA had the highest odds ratio, ranging from 1.37 to 1.57 for all age groups [13].

Previous meta-analyses by Marra et al. [12] and Kawai et al. [14] reviewed data up to 2019. Since then, additional relevant studies examining risk factors for HZ have been published. Our aim is extending already existing evidence by broadening the search and including studies from 2019 to 2022. Furthermore, our methodology addresses concerns raised by Marra et al. [12] and Kawai et al. [14], providing a more accurate representation of the association between exposure and outcome. We specifically focus on heterogeneity patterns, using different models to elucidate those. The objective of this meta-analysis is to identify different risk factors for HZ to determine the importance of certain possibly underestimated risks regarding HZ infections. This could provide helpful information for health care professionals to identify patients at elevated risk in all age groups.

## Methods

### Systematic review

This systematic review and meta-analysis was conducted following the Preferred Reporting Items for Systematic Reviews and Meta-Analyses statement (PRISMA) [15] (see supplementary information [SI], S1–2).

#### Search strategy and study selection

The electronic databases MEDLINE via PubMed, EMBASE and Web of Science were used to identify relevant articles reporting HZ infections and associated risk factors. The search strategy included search terms associated with HZ, epidemiological effect size estimators and underlying conditions. Details of the search strategy are provided in SI S3. In addition, reference lists of identified studies were searched manually for further publications. The searches were limited to English- and German-language studies published from January 1, 2003 until January 1, 2023.

Eligible studies were case–control or cohort studies that assessed the association between HZ and underlying diseases. All other publication types (letters, editorials, comments, case reports and articles without full text, e.g. conference abstracts) were excluded. Studies with a focus on the impact of immunosuppressive or antirheumatic medications, including biologics and corticosteroids as well as studies investigating only family history, age, race or sex as potential risk factors for HZ were excluded as well, since these studies analyzed mostly too narrow subgroups. Two authors (MS and MLH) independently screened titles and abstracts, and full-text articles were screened based on predetermined inclusion and exclusion criteria. Discrepancies were resolved by consensus. A third reviewer (WG) was involved, if consensus could not be reached.

#### Data extraction

Data were extracted by two review authors (MS and MLH) using an Excel spreadsheet. The following information was extracted from the included studies: details about the study design and analysis, data base (questionnaire, claims, data linkage or medical records), country, study period, and population.

Numerous researchers emphasize the importance of including a substantial number of studies in a meta-analysis to ensure reliability of inferential outcomes [16, 17]. When the number of studies (*k*) is small, conventional thresholds for statistical significance (*p* < 0.05) often cannot be met. Permutation tests, which were used later in the analyses, can only achieve significance when *k* > 4 [18]. To address this issue and enhance reliability and interpretability of our results, we chose to include at least *k* ≥ 5 studies for each risk factor in our meta-analyses. Since there were not at least five studies for every disease found in our systematic review, we grouped them into superordinate risk factor groups if *k* < 5. The risk factors for HZ were categorized into 21 risk factor groups: asthma, autoimmune disorders (e.g. primary Sjörgen’s syndrome, multiple sclerosis, vasculitis), cancer, cardiovascular disorders, chronic heart failure (CHF), COPD, depression, diabetes, digestive disorders, endocrine and metabolic disorders, hematological disorders, HIV, inflammatory bowel disease (IBD), mental health conditions, musculoskeletal disorders, neurological disorders, psoriasis, renal disorders, RA, systemic lupus erythematosus (SLE) and transplantation. If a study reported more than one risk factor of interest, it was included in all analyses of each corresponding group. When risk factors are referred to in the following, the aforementioned risk factor groups are meant.

As effect estimates, odds ratios (OR) were extracted. If authors did not report OR, the absolute case numbers (HZ positive and HZ negative cases, cases with and without underlying condition, total study population) were extracted, to calculate the OR as a standard measure for the meta-analysis. Furthermore, characteristics of the study population (e.g. gender, age, follow-up period) were obtained.

#### Quality assessment

Two independent reviewers (MS and AF) used the Newcastle–Ottawa Quality Assessment Scale (NOS) [19], a standard tool from the Cochrane Collaboration Non-Randomized Studies Working Group, to assess the methodological quality of the studies. This scale evaluates three main categories: selection of the study sample (four items), comparability of the sample groups (two points), and ascertainment of exposure (for case–control and cross-sectional studies) or outcome (for cohort studies) (three points). Case–control and cohort studies were rated out of a total of nine points. A predefined threshold of six points was chosen as fair quality, a threshold of seven or more was chosen to indicate good methodological quality [19]. Those studies below the threshold of five points were categorized as poor quality and excluded for meta-analysis. Furthermore, NOS was combined with a critical revision of the appropriateness of statistical adjustment for confounding (e.g. matching, stratification, use of multivariate models).

### Meta-analysis

#### Statistical analyses

All statistical analyses were conducted in R, version 4.3.1, using the meta, dmetar and metafor packages [20–23]. To ensure consistency and transparency of effect sizes for heterogenous studies, recalculation is recommended [24]. However, not all studies provided complete raw data that would have enabled the recalculation of effect sizes. In such cases, the reported effect estimates were used. This approach allowed the inclusion of studies that might otherwise have been excluded due to data unavailability. Furthermore, multiple effect size estimates for the same risk factor (group) in the same study were included as separate effect sizes in the meta-analysis. For pooled OR and their corresponding standard errors (SE) a log transformation was implemented to normalize the distribution of the data.

In anticipation of substantial heterogeneity with varying sample sizes and potential deviations from normality across studies, random-effects models using the Hartung–Knapp and Sidik–Jonkman adjustment were applied across all analyses for every risk factor. This adjustment widens the confidence interval to reflect uncertainty in the estimation of between-study heterogeneity [25–27]. Prediction intervals (95% PI) were computed around the pooled effect sizes to delineate the range within which the actual effects of analogous future trials are likely to reside [28]. A two-sided significance level of *α* = 0.05 was adopted for all analyses.

Heterogeneity was estimated using the *I*^2^ statistics [29]. Following general recommendations for the interpretation of *I*^2^ statistics, a value of 25% was considered as insignificant or low, a value of 50% as moderate, and 75% as substantial heterogeneity [24, 30]. To investigate further variations in heterogeneity between studies, meta-analyses on resampled sets of effect sizes were conducted. This allows to create graphical representations of between-study heterogeneity, known as Graphical Display of Study Heterogeneity (GOSH) plots [31].

#### Subgroup analyses

To examine potential sources of heterogeneity, subgroup analyses were conducted. We applied the rule of thumb that at least 10 studies must be available per risk factor to achieve sufficient statistical power in our subgroup analyses [32]. Subgroup analyses were done for predefined moderators; including study design (cohort study or case–control study), study year (2003–2008, 2011–2016 or 2017–2022), region (Europe, Northern America, Asia or Middle East) and sample size (< 100,000, 100,000–999,999 or ≥ 1,000,000).

#### Sensitivity analyses

Further sensitivity analyses that excluded statistical outliers were conducted if substantial heterogeneity (*I*^2^ ≥ 75%) was detected. With this basic outlier removal studies were excluded if their 95% CI was outside the range of the pooled OR [22].

To assess the impact of individual studies on the overall outcome, “leave-one-out” influence analyses were conducted. This process involved recalculating the pooled effect estimate iteratively while omitting one study at a time. Furthermore, diagnostic plots, containing externally standardized residuals, DFFITS values, Cook’s distance, covariance ratio, leave-one-out *τ*^2^ and *Q* values as well as hat values and study weights where used in combination with Baujat plots to identify influential studies [22, 33]. The study which had the largest impact on the effect estimates and the study which had the largest impact on heterogeneity was subsequently excluded.

To assess potential publication bias, contour-enhanced funnel plots and Egger’s tests of the intercept were used to evaluate potential asymmetry. If publication bias was identified, the Duval and Tweedie trim-and-fill method was used to rectify publication bias [34].

#### Meta-regression

Furthermore, multiple meta-regression analyses were conducted to explore potential sources of heterogeneity. Multiple meta-regression analyses were conducted for each risk factor, incorporating multiple predictors (*β*_*k*_) to account for potential variations in effect sizes and heterogeneity. Predefined moderators from the subgroup analyses were used. To assess the presence of multicollinearity among the predictor variables, correlation matrix analyses were performed. Hierarchical multiple meta-regression was done, by including the covariates in a stepwise procedure. The best-fitting model for each risk factor was chosen based on the Akaike Information Criterion (AIC) values, with preference given to models demonstrating the lowest AIC. To assess the validity of the coefficients capturing the underlying data patterns, permutation tests were employed [35].

## Results

### Study characteristics

**Fig. 1:**
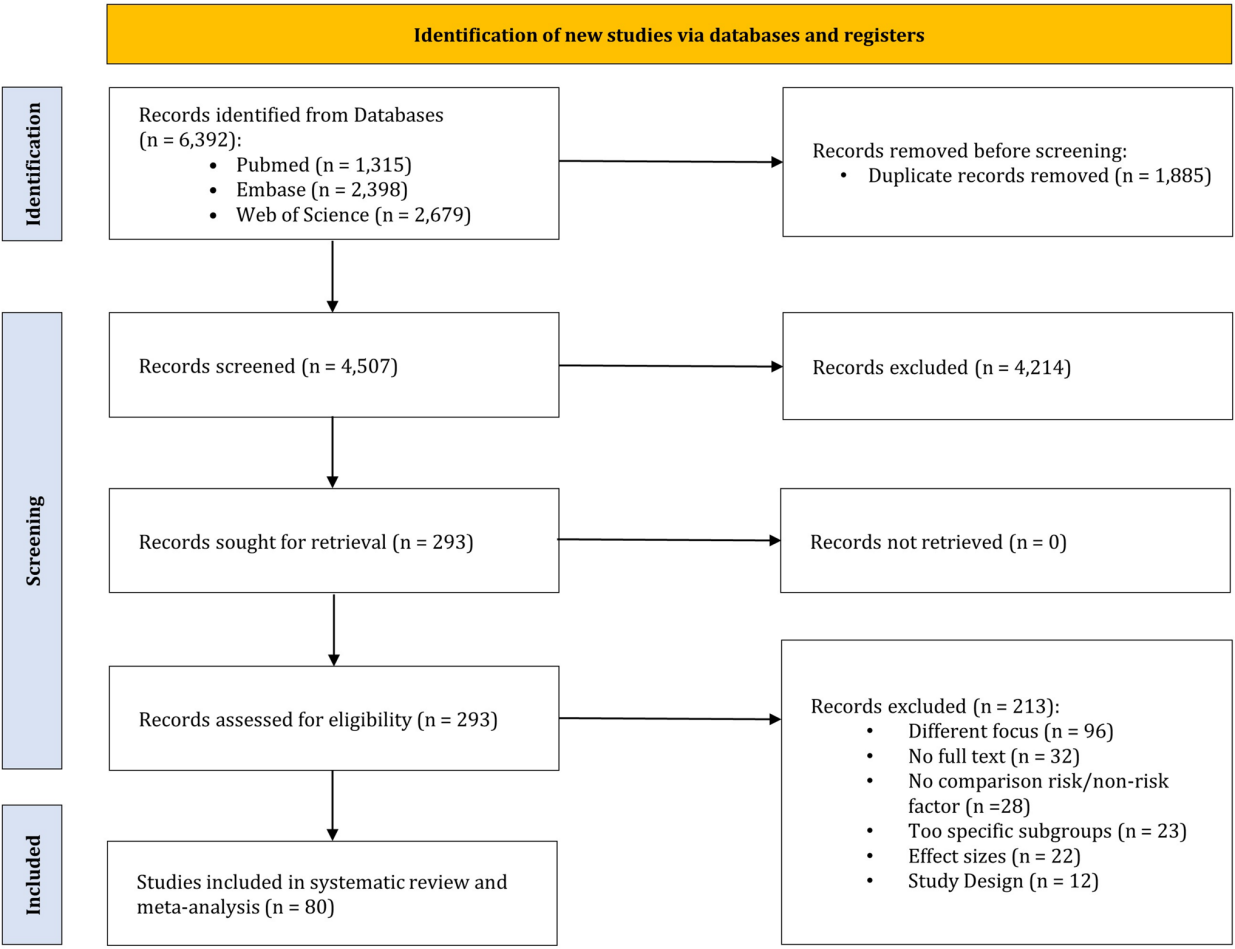
PRISMA flowchart

The initial database search identified 6392 records. Following the removal of duplicates and the screening of titles and abstracts, 293 studies remained for full-text analysis. Reasons for exclusion after full-text screening were: (i) different focus, no focus on underlying risk factors, or risk factors could not be assigned to one of our risk factors (*n* = 96), (ii) conference abstracts or no full text available (*n* = 32), (iii) no comparison between risk factor and non-risk factor (*n* = 28), (iv) analysis of subgroups with risk specific drug use or other overly specific subgroups (*n* = 23), (v) different effect sizes or calculation of odds ratios is not possible with available data (*n* = 22) and (vi) study designs other than cohort or case–control studies (*n* = 12). Ultimately, 80 studies met all eligibility criteria and were included in the analysis. The study selection process and the rationale for exclusions are presented in Fig. 1. 56 of the included studies were cohort studies [13, 36–90] and 24 case–control studies [88, 91–113].

An overview of the distribution of studies on risk factors can be found in Table 1. The most frequent evidence was found for patients with cancer (*n* = 20). Details of all grouped risk factors can be found in SI S4.

**Table 1:** Distribution of risk factors among included studies

Risk factor	Total number of analyses in all studies	Studies including analyses of risk factors^a^
Cancer	20	[45, 49, 51, 52, 57, 86, 89, 93, 103]
Diabetes	17	[13, 38, 41, 45, 49, 51, 52, 56, 59, 73, 82, 92, 94, 99, 100, 103]
Autoimmune disorders	16	[40, 45, 49, 51, 52, 77, 86, 90, 92, 103]
Mental health condition	15	[38, 44, 74, 85, 106, 109, 110, 113, 114]
Musculoskeletal disorders	14	[51, 52, 81, 86, 95–97, 99, 103, 107]
RA	13	[13, 49, 51, 52, 69, 80, 86, 88, 89, 92]
HIV	12	[37, 45–47, 63, 71, 82, 86, 89, 92, 94, 103]
Asthma	11	[13, 43, 45, 51, 52, 68, 92, 101–103, 112]
Cardiovascular disorders	11	[13, 45, 49, 51, 52, 99, 103, 111]
Digestive disorders	11	[38, 53, 55, 58, 61, 65, 72, 90, 103]
COPD	10	[13, 45, 51, 52, 67, 75, 85, 92, 99, 103],
Transplantation	10	[36, 48, 51, 52, 82, 86, 89, 92]
SLE	10	[39, 42, 49, 51, 52, 66, 69, 86, 89, 92]
Renal disorders	10	[49, 51, 52, 62, 84, 86, 87, 92, 104, 105]
Depression	9	[13, 51, 52, 91, 92, 99, 106, 109, 110]
Endocrine and metabolic disorders	8	[50–52, 86, 99]
IBD	8	[51, 52, 55, 64, 72, 86, 89, 92]
CHF	7	[13, 45, 51, 52, 83, 103, 111]
Hematological disorders	7	[60, 92, 94, 98]
Neurological disorders	6	[51, 52, 54, 78, 79, 103]
Psoriasis	6	[51, 52, 76, 86, 89, 108]

RA, rheumatoid arthritis; COPD, chronic obstructive pulmonary disorder; SLE, systemic lupus erythematosus; IBD, inflammatory bowel disease; CHF, chronic heart failure

^a^Please note that a single study may include both analyses of multiple risk factors and multiple analyses of the same risk factor

The included studies were from Asia (*n* = 44), Northern America (*n* = 20), Europe (*n* = 13) and the Middle East (*n* = 3). Most studies were from Taiwan (*n* = 30) [40, 41, 43, 44, 50, 54, 58–63, 68, 76–79, 81, 83–85, 87, 90, 96–98, 100, 104, 105, 115] and the US (*n* = 19) [36, 37, 39, 46, 47, 55, 57, 64, 73, 75, 80, 88, 89, 99, 101, 102, 106, 112, 113]. In sum a total study population of 796,796,295 was included, with 10,904,736 HZ cases reported in the included studies. Sample sizes varied substantially from *n* = 94 to *n* = 51,022,838. The age of participants ranged from 3 months to 103 years with a median age of 52.5 years across all studies. The frequency of women ranged from 0 to 100% with a median of 54.7% across all studies.

### Quality assessment

The quality of the included studies, based on the Newcastle Ottawa scale, ranged from six to nine for cohort and from five to nine for case–control studies. Most (91%) of the cohort studies showed good overall quality and a sufficient adjustment for confounding, as well as most of the case–control studies (92%). Since no study was below the predefined threshold of five points, all were included for further analyses.

### Main analyses

**Fig. 2:**
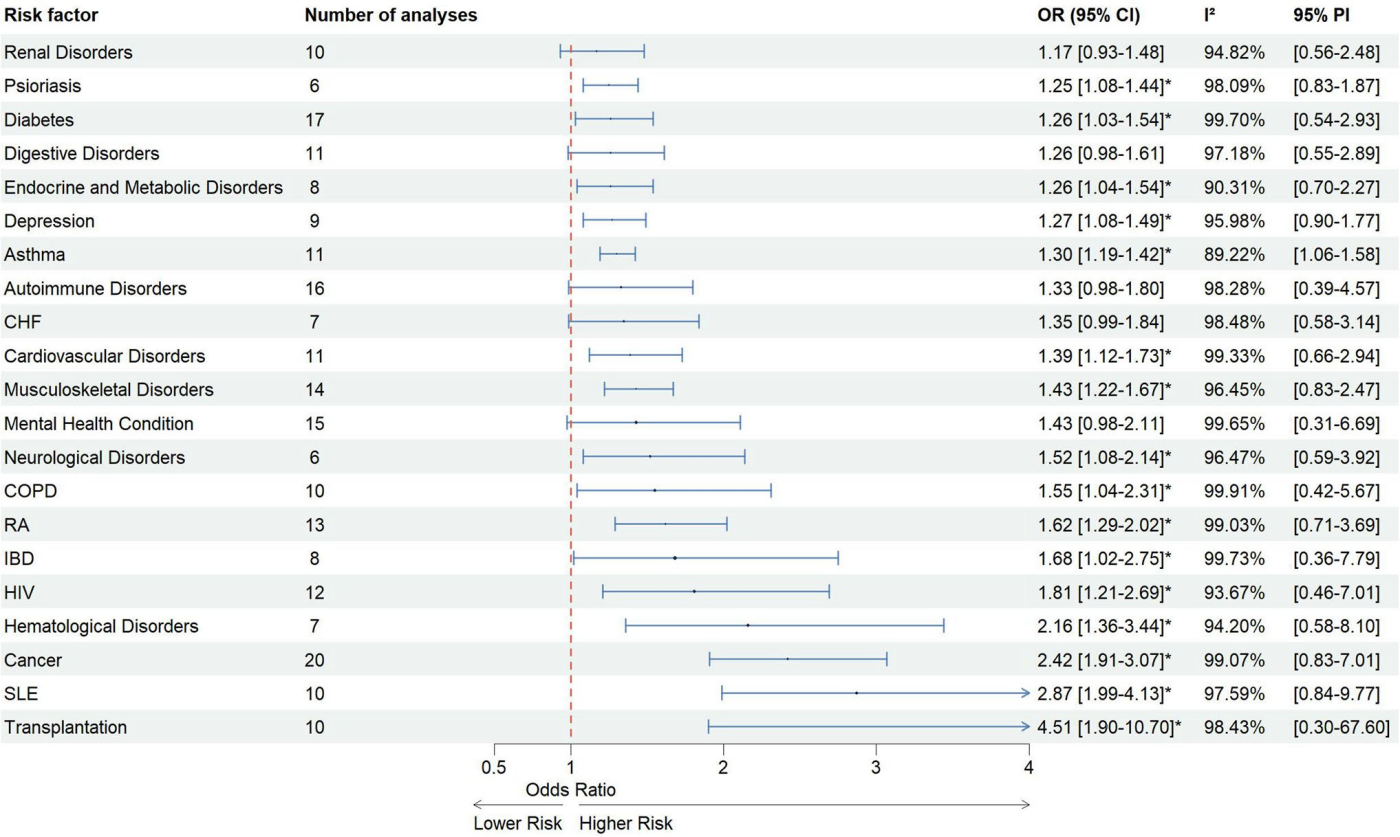
Pooled analysis for risk of herpes zoster

Pooled results showed an increased risk of HZ infections for all included risk factors and are presented in Fig. 2. Data demonstrated a noteworthy association between all analyzed risk factors and HZ, ranging from a pooled OR of 1.17 (95% CI [0.93–1.48]) for renal disorders up to 2.87 for SLE (95% CI [1.99–4.13]).

Between-study heterogeneity was high for all risk factors, varying from *I*^2^ = 89.22% for asthma to *I*^2^ = 99.91% for COPD. Prediction intervals, estimating the range within which future observations are expected to fall with a 95% level of confidence, showed quite precise estimates (e.g. *g* = 1.06–1.58 for asthma), but were also notably broad and thus, indicating a considerable degree of uncertainty in estimating future values (e.g. *g* = 0.30–67.60 for transplantation). Detailed forest plots for all risk factors not shown here can be found in SI S5.

**Fig. 3:**
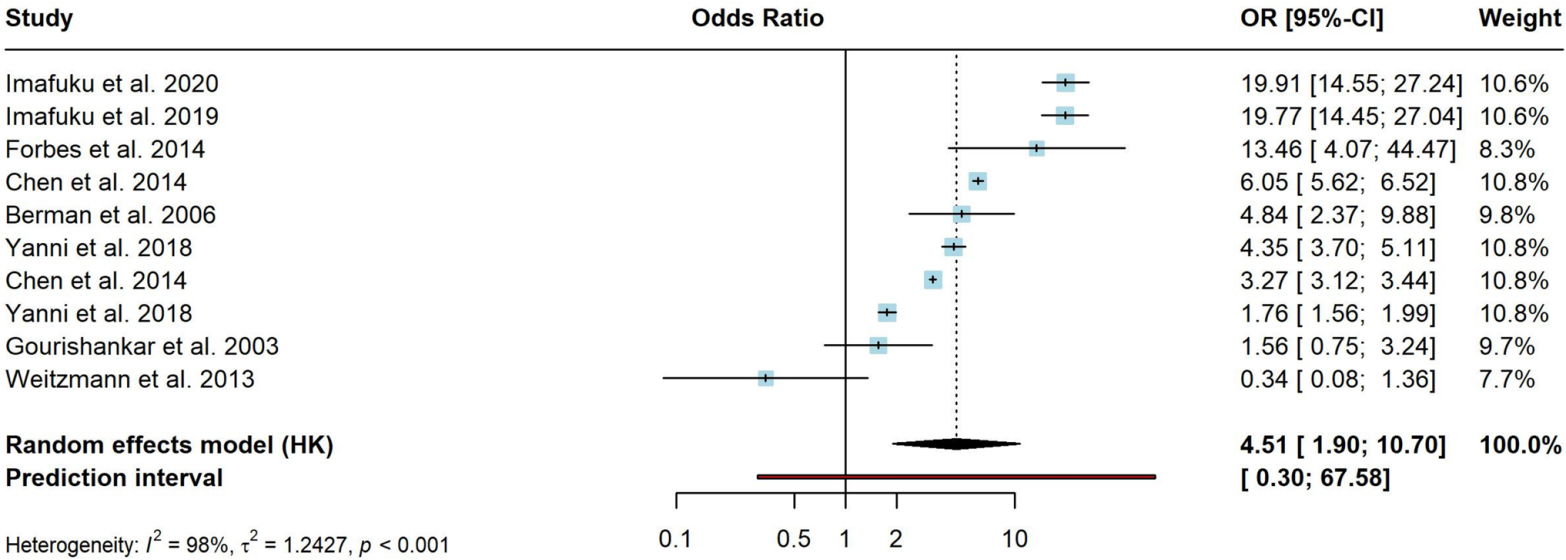
Forest plot for risk of herpes zoster in transplantation subgroup. The transplantation subgroup summarizes allogenic, bone marrow, (solid) organ and hematopoietic stem cell transplantation

Transplantation (Fig. 3) was associated with the highest risk of HZ with a pooled OR = 4.51 (95% CI [1.9–10.7]). However, the effects between studies varied substantially, as evidenced by high between-study heterogeneity (*I*^2^ = 98.4%). The prediction interval ranged from *g* = 0.3–67.58. The heterogeneity remained substantial (*I*^2^ = 97.3%) after sensitivity analyses with basic outlier removal and leave-one-out analysis, but reduced the pooled effect size to OR = 3.55 (95% CI [1.3–9.8]). GOSH diagnostics did not provide a unimodal, symmetrical or contiguous distribution; thus, effect sizes were still quite heterogenous.

**Fig. 4:**
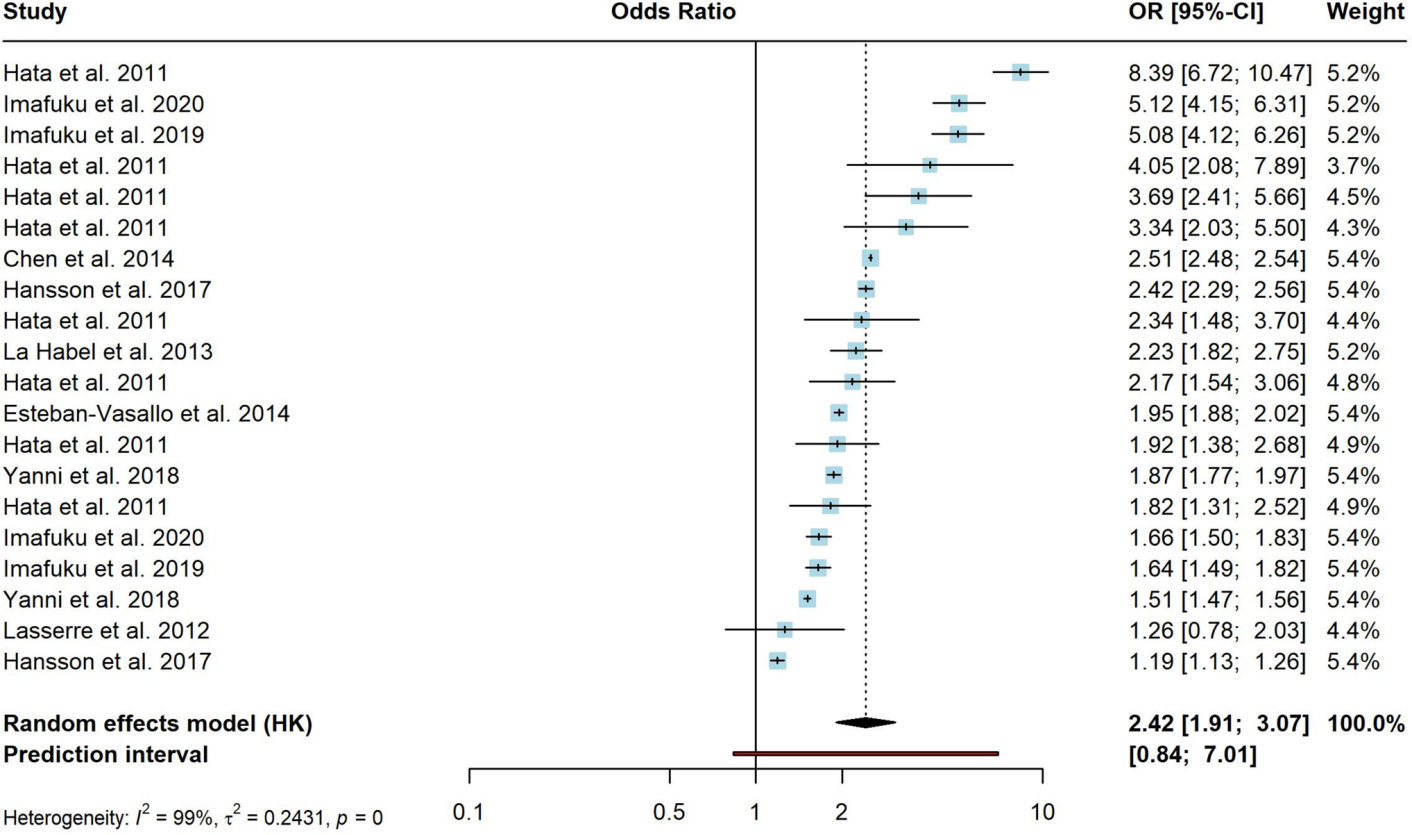
Forest plot for risk of herpes zoster in cancer subgroup. The cancer subgroup summarizes any solid malignancy, hematological malignancies, solid organ malignancies, brain tumor, lung cancer, breast cancer, esophageal cancer, gastric cancer, colorectal cancer, gynecologic cancer and malignant lymphoma

The risk factor with most available evidence, various forms of malignancies and cancer, were associated with a significant higher risk of HZ, indicating a pooled OR of 2.42 (95% CI [1.91–3.07]) (Fig. 4). Reported effect sizes differed substantially between studies (*I*^2^ = 99.1%). Prediction interval ranged between *g* = 0.84–7.01. After sensitivity analyses, between-study heterogeneity was still high (*I*^2^ = 95.3%), but an increased risk of HZ remained (OR = 2.21; 95% CI [1.9–2.6]).

### Subgroup analyses

The subgroup analyses only revealed a significant difference between studies when testing for regional differences (asthma, digestive disorder, SLE and transplantation, see SI S6 for further details). However, all conducted *Q* tests for within-subgroup heterogeneity showed significance, therefore, indicating, that there is excess variability in the subgroups.

### Sensitivity analyses

Since heterogeneity was high for all risk factors and subgroup analyses revealed a clear tendency for heterogeneity within the subgroups, five different sensitivity analyses were done for each of the risk factors. Even after outlier removal, for all risk factors the pooled estimates still provided OR ≥ 1 for developing a HZ infection.

For cancer, cardiovascular disorders, COPD, diabetes, digestive disorders, IBD, mental health disorders, musculoskeletal disorders, neurological disorders, renal disorders, RA and SLE the basic outlier removal emerged as the most effective method in mitigating heterogeneity (see Table 2 for further details and SI S7 for corresponding plots). Systematically eliminating data points whose confidence interval did not overlap with the confidence interval of the pooled effect exerted a profound influence on the results. After basic outlier removal, all pooled OR decreased compared to the unadjusted random effects model. The effect was highest for mental health conditions (adj. OR = 1.16, 95% CI [1.0–1.3], *I*^2^ = 87.85%). This reduction indicates that by excluding extreme data points, consistency and robustness of the findings is enhanced, leading to a clearer representation of the risk patterns within the studied populations. Influence analyses, based on the “leave-one-out” approach was done, iteratively reassessing the results and excluding one study at a time. These revealed the greatest impact on the heterogeneity of asthma, autoimmune disorders, COPD, endocrine and metabolic disorders, hematological disorders, neurological disorders, transplantation and psoriasis. Remarkably, “leave-one-out” analysis increased the pooled effect estimates simultaneously, with the highest value for autoimmune disorders (prev. OR = 1.33, adj. OR = 1.46, 95% CI [1.3–1.7]; *I*^2^ = 92.55%, resp.). GOSH diagnostics helped reducing the heterogeneity for depression, HIV and CHF. For psoriasis, heterogeneity could even be reduced to 0%, but showed wide confidence intervals for *I*^2^ (adj. OR = 1.21, 95% CI [1.1–1.3], *I*^2^ = 0% (95% CI [0–85.00])).

**Table 2:** Pooled effects of risk factors on HZ, sensitivity analyses

Risk factor	Effect size			Heterogeneity			Excluded studies
	*n*_*k*_	OR	95% CI	*I*^2^ (%)	95% CI	95% PI	
*Asthma*							
Random effects model, unadjusted	11	1.30	[1.19–1.42]^a^	89.22	[83–93.00]^a^	0.06–0.46	–
Basic outlier removal	11	1.30	[1.20–1.40]^a^	89.22	[83–93.00]^a^	0.06–0.46	–
Influence analysis („leave-one-out”)	10	1.27	[1.20–1.40]^a^	65.59	[33–82.00]^a^	1.08–1.48	[45]
GOSH-Diagnostics Connectivity (DBSCAN) clustering	10	1.27	[1.20–1.40]^a^	65.59	[33–82.00]^a^	1.08–1.48	[45]
Gaussian mixture model (GMM) clustering	10	1.28	[1.20–1.40]^a^	89.66	[83–94.00]^a^	1.05–1.56	[68]
*K*-means clustering	10	1.30	[1.20–1.40]^a^	90.11	[84–94.00]^a^	1.06–1.60	[52]
*Autoimmune disorders*							
Random effects model, unadjusted	16	1.33	[0.98–1.80]	98.28	[98–99.00]^a^	− 0.95–1.52	–
Basic outlier removal	13	1.40	[1.20–1.60]^a^	91.11	[87–94.00]^a^	− 0.14–0.81	[40, 45, 77]
Influence analysis („leave-one-out”)	14	1.46	[1.30–1.70]^a^	92.55	[89–95.00]^a^	0.84–2.54	[45, 77]
GOSH-Diagnostics Connectivity (DBSCAN) clustering	9	1.49	[1.20–1.90]^a^	96.22	[94–97.00]^a^	0.71–3.09	[49, 51, 52, 77, 89, 92]
Gaussian mixture model (GMM) clustering	9	1.49	[1.20–1.90]^a^	96.22	[94–97.00]^a^	0.71–3.09	[49, 51, 52, 77, 89, 92]
*K*-means clustering	15	1.53	[1.30–1.80]^a^	95.35	[94–97.00]^a^	0.82–2.84	[89]
*Cancer*							
Random effects model, unadjusted	20	2.42	[1.91–3.07]^a^	99.07	[99–99.00]^a^	− 0.18–1.95	–
Basic outlier removal	13	2.21	[1.90–2.60]^a^	95.93	[94–97.00]^a^	0.43–1.16	[49, 86, 93, 116, 117]
Influence analysis („leave-one-out”)	18	2.24	[1.80–2.80]^a^	97.58	[97–98.00]^a^	0.90–5.53	[49, 89]
GOSH-Diagnostics Connectivity (DBSCAN) clustering	13	2.45	[2.00–3.10]^a^	97.26	[96–98.00]^a^	1.09–5.55	[49, 52, 86, 89, 93, 103]
Gaussian mixture model (GMM) clustering	13	2.45	[2.00–3.10]^a^	97.26	[96–98.00]^a^	1.09–5.55	[49, 52, 86, 89, 93, 103]
*K*-means clustering	19	2.25	[1.80–2.80]^a^	99.05	[99–99.00]^a^	0.94–5.37	[103]
*Cardiovascular disorders*							
Random effects model, unadjusted	11	1.39	[1.12–1.73]^a^	99.33	[99–99.00]^a^	− 0.42–1.08	–
Basic outlier removal	8	1.30	[1.00–1.60]^a^	92.62	[88–96.00]^a^	− 0.44–0.96	[13, 45]
Influence analysis („leave-one-out”)	9	1.37	[1.10–1.80]^a^	98.62	[98–99.00]^a^	0.64–2.94	[13, 45]
GOSH-Diagnostics Connectivity (DBSCAN) clustering	7	1.26	[0.92–1.70]	99.39	[99–100.00]^a^	0.50–3.17	[45, 103, 111]
Gaussian mixture model (GMM) clustering	7	1.26	[0.92–1.70]	99.39	[99–100.00]^a^	0.50–3.17	[45, 103, 111]
*K*-means clustering	10	1.40	[1.10–1.80]^a^	99.40	[99–100.00]^a^	0.64–3.07	[45]
*CHF*							
Random effects model, unadjusted	7	1.35	[0.99–1.84]	98.48	[98–99.00]^a^	− 0.55–1.15	–
Basic outlier removal	6	1.24	[0.93–1.70]	91.74	[85–96.00]^a^	− 0.46–0.90	[45]
Influence analysis („leave-one-out”)	6	1.24	[0.93–1.70]	91.74	[85–96.00]^a^	0.63–2.45	[45]
GOSH-Diagnostics Connectivity (DBSCAN) clustering	5	1.32	[0.93–1.90]	76.13	[42–90.00]^a^	0.64–2.73	[83, 111]
Gaussian mixture model (GMM) clustering	5	1.32	[0.93–1.90]	76.13	[42–90.00]^a^	0.64–2.73	[83, 111]
*K*-means clustering	5	1.26	[0.85–1.90]	93.35	[87–96.00]^a^	0.49–3.24	[45, 111]
*COPD*							
Random effects model, unadjusted	10	1.55	[1.04–2.31]^a^	99.91	[100–100.00]^a^	− 0.86–1.74	–
Basic outlier removal	9	1.38	[1.10–1.70]^a^	99.08	[99–99.00]^a^	− 0.28–0.92	[75]
Influence analysis („leave-one-out”)	9	1.38	[1.10–1.70]^a^	99.08	[99–99.00]^a^	0.76–2.51	[75]
GOSH-Diagnostics Connectivity (DBSCAN) clustering	8	1.34	[1.10–1.70]^a^	99.19	[99–99.00]^a^	0.70–2.54	[45, 85]
Gaussian mixture model (GMM) clustering	8	1.34	[1.10–1.70]^a^	99.19	[99–99.00]^a^	0.70–2.54	[45, 85]
*K*-means clustering	9	1.38	[1.10–1.70]^a^	99.08	[99–99.00]^a^	0.76–2.51	[85]
*Depression*							
Random effects model, unadjusted	9	1.27	[1.08–1.49]^a^	95.98	[94–97.00]^a^	− 0.10–0.57	–
Basic outlier removal	8	1.23	[1.10–1.40]^a^	96.18	[94–97.00]^a^	− 0.08–0.50	[106]
Influence analysis („leave-one-out”)	8	1.21	[1.10–1.40]^a^	84.52	[71–92.00]^a^	0.98–1.48	[99]
GOSH-Diagnostics Connectivity (DBSCAN) clustering	6	1.20	[1.10–1.30]^a^	82.43	[63–92.00]^a^	0.96–1.50	[13, 51, 52]
Gaussian mixture model (GMM) clustering	6	1.20	[1.10–1.30]^a^	82.43	[63–92.00]^a^	0.96–1.50	[13, 51, 52]
*K*-means clustering	8	1.23	[1.10–1.40]^a^	96.18	[94–97.00]^a^	0.93–1.64	[13]
*Diabetes*							
Random effects model, unadjusted	17	1.26	[1.03–1.54]^a^	99.70	[100–100.00]^a^	− 0.62–1.08	–
Basic outlier removal	13	1.21	[1.10–1.40]^a^	96.33	[95–97.00]^a^	− 0.22–0.60	[45, 49, 73, 82]
Influence analysis („leave-one-out”)	14	1.26	[1.10–1.40]^a^	99.38	[99–99.00]^a^	0.76–2.09	[49, 73, 82]
GOSH-Diagnostics Connectivity (DBSCAN) clustering	15	1.24	[1.00–1.50]	99.74	[100–100.00]^a^	0.52–2.99	[13, 45]
Gaussian mixture model (GMM) clustering	15	1.24	[1.00–1.50]	99.74	[100–100.00]^a^	0.52–2.99	[13, 45]
*K*-means clustering	15	1.29	[1.10–1.50]^a^	99.69	[100–100.00]^a^	0.76–2.18	[41, 100]
*Digestive disorders*							
Random effects model, unadjusted	11	1.26	[0.98–1.61]	97.18	[96–98.00]^a^	− 0.60–1.06	–
Basic outlier removal	6	1.36	[1.20–1.60]^a^	73.86	[40–89.00]^a^	0.04–0.57	[55, 61, 72, 90]
Influence analysis („leave-one-out”)	9	1.20	[0.92–1.60]	96.68	[95–98.00]^a^	0.53–2.75	[58, 72]
GOSH-Diagnostics Connectivity (DBSCAN) clustering	7	1.14	[0.78–1.70]	98.24	[98–99.00]^a^	0.37–3.48	[38, 53, 65, 72]
Gaussian mixture model (GMM) clustering	7	1.14	[0.78–1.70]	98.24	[98–99.00]^a^	0.37–3.48	[38, 53, 65, 72]
*K*-means clustering	9	1.16	[0.91–1.50]	96.69	[95–98.00]^a^	0.53–2.54	[38, 58]
*Endocrine and metabolic disorders*							
Random effects model, unadjusted	8	1.26	[1.04–1.54]^a^	90.31	[83–94.00]^a^	− 0.35–0.82	–
Basic outlier removal	7	1.33	[1.10–1.60]^a^	88.96	[80–94.00]^a^	− 0.18–0.76	[86]
Influence analysis („leave-one-out”)	6	1.29	[1.10–1.50]^a^	86.50	[73–93.00]^a^	0.79–2.09	[51, 86]
GOSH-Diagnostics Connectivity (DBSCAN) clustering	7	1.33	[1.10–1.60]^a^	88.96	[80–94.00]^a^	0.83–2.13	[52]
Gaussian mixture model (GMM) clustering	7	1.33	[1.10–1.60]^a^	88.96	[80–94.00]^a^	0.83–2.13	[52]
*K*-means clustering	7	1.29	[1.00–1.60]^a^	91.67	[85–95.00]^a^	0.67–2.49	[50]
*Hematological disorders*							
Random effects model, unadjusted	7	2.16	[1.36–3.44]^a^	94.20	[90–96.00]^a^	− 0.55–2.09	–
Basic outlier removal	7	2.16	[1.40–3.40]^a^	94.20	[90–96.00]^a^	− 0.55–2.09	–
Influence analysis („leave-one-out”)	5	2.19	[1.50–3.30]^a^	81.02	[56–92.00]^a^	0.76–6.32	[60, 118]
GOSH-Diagnostics Connectivity (DBSCAN) clustering	5	2.19	[1.50–3.30]^a^	81.02	[56–92.00]^a^	0.76–6.32	[92, 98]
Gaussian mixture model (GMM) clustering	5	2.19	[1.50–3.30]^a^	81.02	[56–92.00]^a^	0.76–6.32	[92, 98]
*K*-means clustering	5	2.43	[1.50–4.00]^a^	94.94	[91–97.00]^a^	0.62–9.50	[92]
*HIV*							
Random effects model, unadjusted	12	1.81	[1.21–2.69]^a^	93.67	[91–96.00]^a^	− 0.77–1.95	–
Basic outlier removal	10	1.43	[1.00–2.00]^a^	87.19	[78–92.00]^a^	− 0.61–1.32	[89, 118]
Influence analysis („leave-one-out”)	10	1.69	[1.10–2.50]^a^	87.43	[79–93.00]^a^	0.52–5.52	[86, 92]
GOSH-Diagnostics Connectivity (DBSCAN) clustering	8	1.64	[0.90–3.00]	92.07	[87–95.00]^a^	0.28–9.60	[82, 86, 89, 118]
Gaussian mixture model (GMM) clustering	8	1.64	[0.90–3.00]	92.07	[87–95.00]^a^	0.28–9.60	[82, 86, 89, 118]
*K*-means clustering	9	1.55	[1.10–2.10]^a^	87.12	[78–93.00]^a^	0.61–3.92	[63, 71, 86]
*IBD*							
Random effects model, unadjusted	8	1.68	[1.02–2.75]^a^	99.73	[100–100.00]^a^	− 1.02–2.05	–
Basic outlier removal	6	1.50	[1.30–1.80]^a^	97.38	[96–98.00]^a^	− 0.05–0.86	[55, 64]
Influence analysis („leave-one-out”)	7	1.86	[1.10–3.10]^a^	99.75	[100–100.00]^a^	0.41–8.57	[64]
GOSH-Diagnostics Connectivity (DBSCAN) clustering	7	1.38	[1.10–1.80]^a^	98.02	[97–99.00]^a^	0.66–2.87	[52]
Gaussian mixture model (GMM) clustering	7	1.38	[1.10–1.80]^a^	98.02	[97–99.00]^a^	0.66–2.87	[52]
*K*-means clustering	7	1.38	[1.10–1.80]^a^	98.02	[97–99.00]^a^	0.66–2.87	[52]
*Mental health condition*							
Random effects model, unadjusted	15	1.43	[0.98–2.11]	99.65	[100–100.00]^a^	− 1.18–1.90	–
Basic outlier removal	13	1.16	[1.00–1.30]^a^	87.85	[81–92.00]^a^	− 0.19–0.48	[106, 114]
Influence analysis („leave-one-out”)	14	1.23	[1.00–1.50]^a^	91.08	[87–94.00]^a^	0.66–2.28	[114]
GOSH-Diagnostics Connectivity (DBSCAN) clustering	9	1.64	[0.83–3.20]	99.80	[100–100.00]^a^	0.18–14.50	[44, 74, 109, 110, 114]
Gaussian mixture model (GMM) clustering	9	1.64	[0.83–3.20]	99.80	[100–100.00]^a^	0.18–14.50	[44, 74, 109, 110, 114]
*K*-means clustering	14	1.23	[1.00–1.50]^a^	91.08	[87–94.00]^a^	0.66–2.28	[110]
*Musculoskeletal disorders*							
Random effects model, unadjusted	14	1.43	[1.22–1.67]^a^	96.45	[95–97.00]^a^	− 0.19–0.90	–
Basic outlier removal	11	1.3	[1.20–1.40]^a^	88.21	[81–93.00]^a^	0.00–0.52	[51, 52, 86]
Influence analysis („leave-one-out”)	12	1.37	[1.20–1.60]^a^	91.37	[87–94.00]^a^	0.78–2.39	[52, 86]
GOSH-Diagnostics Connectivity (DBSCAN) clustering	9	1.45	[1.10–1.90]^a^	93.61	[90–96.00]^a^	0.66–3.23	[52, 81, 99, 103]
Gaussian mixture model (GMM) clustering	9	1.45	[1.10–1.90]^a^	93.61	[90–96.00]^a^	0.66–3.23	[52, 81, 99, 103]
*K*-means clustering	12	1.34	[1.20–1.50]^a^	96.48	[95–97.00]^a^	0.88–2.04	[86, 107]
*Neurological disorders*							
Random effects model, unadjusted	6	1.52	[1.08–2.14]^a^	96.47	[94–98.00]^a^	− 0.53–1.37	–
Basic outlier removal	5	1.32	[1.10–1.60]^a^	88.15	[75–94.00]^a^	− 0.26–0.82	[79]
Influence analysis („leave-one-out”)	5	1.32	[1.10–1.60]^a^	88.15	[75–94.00]^a^	0.77–2.27	[79]
GOSH-Diagnostics Connectivity (DBSCAN) clustering	5	1.32	[1.10–1.60]^a^	88.15	[75–94.00]^a^	0.77–2.27	[51]
Gaussian mixture model (GMM) clustering	5	1.32	[1.10–1.60]^a^	88.15	[75–94.00]^a^	0.77–2.27	[51]
*K*-means clustering	4	1.35	[1.00–1.80]^a^	91.00	[80–96.00]^a^	0.61–2.97	[51, 54]
*Psoriasis*							
Random effects model, unadjusted	6	1.25	[1.08–1.44]^a^	98.09	[97–99.00]^a^	− 0.18–0.63	–
Basic outlier removal	5	1.16	[1.10–1.30]^a^	59.33	[0 -85.00]^a^	− 0.10–0.40	[89]
Influence analysis („leave-one-out”)	4	1.21	[1.10–1.30]^a^	0.00	[0–85.00]	1.04–1.42	[86, 89]
GOSH-Diagnostics Connectivity (DBSCAN) clustering	6	1.25	[1.10–1.40]^a^	98.09	[97–99.00]^a^	0.83–1.87	–
Gaussian mixture model (GMM) clustering	6	1.25	[1.10–1.40]^a^	98.09	[97–99.00]^a^	0.83–1.87	–
*K*-means clustering	4	1.21	[1.10–1.30]^a^	0.00	[0–85.00]	1.04–1.42	[51, 86]
*Renal disorders*							
Random effects model, unadjusted	10	1.17	[0.93–1.48]	94.82	[92–97.00]^a^	− 0.59–0.91	–
Basic outlier removal	8	1.15	[1.00–1.30]	84.85	[72–92.00]^a^	− 0.22–0.51	[49, 60]
Influence analysis („leave-one-out”)	8	1.28	[1.00–1.60]	90.00	[83–94.00]^a^	0.65–2.51	[62, 118]
GOSH-Diagnostics Connectivity (DBSCAN) clustering	9	1.18	[0.91–1.50]	95.39	[93–97.00]^a^	0.53–2.64	[52]
Gaussian mixture model (GMM) clustering	9	1.26	[1.00–1.50]	89.47	[82–94.00]^a^	0.69–2.28	[52]
*K*-means clustering	8	1.27	[1.00–1.60]	90.79	[84–95.00]^a^	0.66–2.43	[52, 60]
*RA*							
Random effects model, unadjusted	13	1.62	[1.29–2.02]^a^	99.03	[99–99.00]^a^	− 0.34–1.30	–
Basic outlier removal	10	1.62	[1.40–1.90]^a^	93.26	[90–96.00]^a^	− 0.02–0.99	[69, 88, 89]
Influence analysis („leave-one-out”)	10	1.74	[1.40–2.10]^a^	97.16	[96–98.00]^a^	0.98–3.10	[69, 88, 89]
GOSH-Diagnostics Connectivity (DBSCAN) clustering	5	2.08	[1.70–2.50]^a^	95.61	[92–98.00]^a^	1.25–3.47	[13, 49, 51, 86, 88, 89, 92]
Gaussian mixture model (GMM) clustering	5	2.08	[1.70–2.50]^a^	95.61	[92–98.00]^a^	1.25–3.47	[13, 49, 51, 86, 88, 89, 92]
*K*-means clustering	12	1.75	[1.50–2.10]^a^	98.56	[98–99.00]^a^	0.96–3.17	[51]
*SLE*							
Random effects model, unadjusted	10	2.87	[1.99–4.13]^a^	97.59	[97–98.00]^a^	− 0.17–2.28	–
Basic outlier removal	6	2.91	[2.10–4.00]^a^	91.97	[85–96.00]^a^	0.14–2.00	[39, 66, 69, 92]
Influence analysis („leave-one-out”)	8	2.93	[2.10–4.10]^a^	96.95	[96–98.00]^a^	1.05–8.23	[39, 66]
GOSH-Diagnostics Connectivity (DBSCAN) clustering	6	2.63	[1.40–4.90]^a^	94.15	[90–97.00]^a^	0.44–15.90	[49, 51, 52, 92]
Gaussian mixture model (GMM) clustering	6	2.63	[1.40–4.90]^a^	94.15	[90–97.00]^a^	0.44–15.90	[49, 51, 52, 92]
*K*-means clustering	9	3.18	[2.30–4.40]^a^	96.76	[95–98.00]^a^	1.09–9.29	[89]
*Transplantation*							
Random effects model, unadjusted	10	4.51	[1.90–10.70]^a^	98.43	[98–99.00]^a^	− 1.20–4.21	–
Basic outlier removal	7	3.68	[2.00–6.80]^a^	98.27	[98–99.00]^a^	− 0.26–2.87	[51, 52, 82]
Influence analysis („leave-one-out”)	8	3.55	[1.30–9.80]^a^	97.27	[96–98.00]^a^	0.19–64.80	[89, 117]
GOSH-Diagnostics Connectivity (DBSCAN) clustering	9	5.59	[2.70–12.00]^a^	98.58	[98–99.00]^a^	0.56–56.10	[52]
Gaussian mixture model (GMM) clustering	9	5.59	[2.70–12.00]^a^	98.58	[98–99.00]^a^	0.56–56.10	[52]
*K*-means clustering	8	5.15	[2.30–12.00]^a^	98.75	[98–99.00]^a^	0.45–59.30	[48, 52]

*n*_*k*_, number of studies; OR, odds ratio; CI, confidence interval; PI, prediction interval

^a^Indicates significant values *p* < 0.005

### Publication bias analyses

Examination of the funnel plots revealed a potential issue of publication bias within all conducted analyses. For 12 of 21 risk factors, Egger’s test revealed a negative intercept, which suggests that smaller studies included in our analysis may exhibit a systematic tendency to report larger effect sizes than larger studies. Such bias could lead to an overestimation of the true effect estimate, and it underscores the importance of interpreting our meta-analysis results with caution. The negative intercept was highest for diabetes, COPD and RA. For asthma, digestive disorders, musculoskeletal disorders, renal disorders and SLE the intercept was close to zero, indicating less publication bias. Positive intercept, i.e., demonstrating a potential underestimation of the true effect, was largest for the mental health conditions. However, for all analyzed risk factors, the results of Egger’s test for funnel plot asymmetry yielded non-significant values (*p* > 0.05) indicating that there might be no evidence for publication bias within the selected studies (see Table 3). Even though results of the Egger’s test were non-significant, the trim-and-fill procedure was applied as a precautionary measure to assess the impact of potentially missing studies on the overall effect size estimation. However, results of the trim-and-fill procedure were similar to previous results. Since heterogeneity was still quite high in all risk factors, results of the trim-and-fill procedure were not very robust (data available on request).

**Table 3:** Egger's test results for publication bias

Risk factor	Intercept	95% CI	*t* value	*p* value
Asthma	0.647	[− 1.99–3.28]	0.482	0.642
Autoimmune disorders	− 3.278	[− 9.04–2.48]	− 1.116	0.283
Cancer	− 1.852	[− 7.46–3.76]	− 0.647	0.526
Cardiovascular disorders	− 4.191	[− 16.15–7.77]	− 0.687	0.509
CHF	1.614	[− 10.40–13.63]	0.263	0.803
COPD	− 8.910	[− 45.18–27.36]	− 0.482	0.643
Depression	2.688	[− 3.08–8.46]	0.913	0.392
Diabetes	− 10.285	[− 15.74–4.83]	− 1.886	0.079
Digestive disorders	− 0.387	[− 5.94–5.17]	− 0.136	0.895
Endocrine and metabolic disorders	2.038	[− 0.78–4.85]	1.419	0.206
Hematological disorders	− 2.006	[− 8.91–4.90]	− 0.569	0.594
HIV	− 2.349	[− 4.72–0.02]	− 1.943	0.081
IBD	− 3.261	[− 29.32–22.80]	− 0.245	0.814
Mental health condition	6.213	[− 4.09–16.52]	1.182	0.258
Musculoskeletal disorders	0.916	[− 2.83–6.42]	0.388	0.705
Neurological disorders	1.796	[− 5.57–9.16]	0.478	0.658
Psioriasis	− 3.240	[-12.33–5.85]	− 0.699	0.523
RA	− 7.584	[− 14.72–0.45]	− 2.082	0.061
Renal disorders	0.003	[− 3.95–3.96]	0.001	0.999
SLE	− 0.928	[− 6.61–4.76]	− 0.320	0.757
Transplantation	2.305	[− 4.77–9.38]	0.638	0.541

### Meta-regression

A comprehensive meta-regression analysis was conducted to explore potential sources of heterogeneity across the included studies. This aimed to identify the factors that might contribute to variations in effect sizes and heterogeneity. Correlation matrices (see SI S9) showed that there are correlations among variables; however, these correlations do not appear to be substantial enough to justify removing any of these variables from meta-regression analysis. A permutation test was incorporated to ensure the robustness of the findings. While publication year, sample size, region and study design were explored as potential moderators, only two of these moderators (region and year) demonstrated a statistically significant influence on any of the analyzed risk factors. In total, the meta-regression model could explain heterogeneity for psoriasis (*R*^2^ = 100.00%), endocrine and metabolic disorders (*R*^2^ = 98.63%), cardiovascular disorders (*R*^2^ = 72.78%) and CHF (*R*^2^ = 71.88%), but showed no significance for any predictor after permutation testing. Results of the meta-regression and potential moderators are shown in the SI S8.

For CHF, the model indicated a substantial level of residual heterogeneity (*τ*^2^ = 0.0263), contributing to a notable unaccounted variability (*I*^2^ = 83.95%). The meta-regression results revealed significant associations for publication year (*p* = 0.0053). These findings suggest that the publication year of the included studies might have statistically significant impacts on the outcome. However, the permutation test could not confirm the robustness of these associations (*p* > 0.05).

Within the IBD group substantial unexplained variability in effect sizes was observed (*I*^2^ = 96.14%), with the meta-regression model explaining 53.24% of this heterogeneity. Publication year showed a significant negative association with effect sizes (*p* = 0.0007), while study design, region and sample size did not exhibit significant associations (*p* > 0.05). Permutation testing could not verify the robustness of these findings, as moderator effects were not statistically significant (*p* = 0.1160).

For mental health conditions significant unexplained variability in effect sizes was observed, with our model explaining a moderate portion of this heterogeneity (*R*^2^ = 41.97%). Moderator effects were evident, as indicated by significant tests for residual heterogeneity and moderators. Specifically, publication year demonstrated significant association with effect sizes. Permutation testing supported the moderator effect showing significance.

Within the SLE subgroup, our analysis suggests that geographical location (region) significantly influences effect sizes even after permutation testing, while other factors (publication year, study design, and sample size) did not exhibit statistically significant associations after permutation testing.

## Discussion

In this meta-analysis, we aimed to summarize and quantify a range of risk factors associated with HZ incidence. Our findings show, that patients with immunosuppressed conditions, such as transplantation (OR = 4.51) or cancer (OR = 2.42), have the highest risk of HZ. The presence of autoimmune disorders such as RA, SLE, IBD, psoriasis and HIV also increases the risk of HZ. In addition, our analysis underscores the significance of various comorbidities, including renal disorders, hematological disorders, endocrine and metabolic disorders, cardiovascular disorders, CHF, COPD, diabetes, asthma, mental health conditions and depression, in elevating HZ risk.

Two previous meta-analyses examining risk factors for HZ corroborate our results. Marra et al. 2020 determined HIV as the disease with the highest risk for HZ (RR = 3.22; 95% CI [2.4–4.33]), whereas in our pooled analyses, HIV was only associated with an OR = 1.81 (95% CI [1.21–2.69]). Kawai et al. 2017 estimate SLE as disease with the highest risk (RR = 2.1; 95% CI = 1.40–3.15), which is in line with the results by Marra et al. (RR = 2.08; 95% CI [1.56–2.78]) and our results (OR = 2.87; 95% CI [1.99–4.13]). However, both meta-analyses focused not only on diseases as potential risk factors, but also included family history, race, gender and age. They found that, family history of HZ is a risk factor of HZ (OR = 2.48; 95% CI [1.70–3.60] [12] resp. OR = 3.59; 95% CI [2.39–5.40] [14]), indicating a genetic inclination due to the absence of temporal links among cases in relatives. Female gender (OR = 1.19; 95% CI [1.14–1.24] [12] resp. RR = 1.31; 95% CI [1.27–1.34] [14] and older age (RR = 1.65; 95% CI [1.37–1.97] [12]) were also associated with an increased risk of HZ.

Varicella-zoster virus usually remains dormant in sensory ganglia due to cell-mediated immunity (CMI) [119, 120]. Most HZ risk factors relate to weakened CMI. Patients with autoimmune diseases have an elevated HZ risk due to their compromised immunity and medication [8, 121, 122]. For example, individuals with diabetes mellitus have reduced VZV-specific CMI [123]. In addition, depression, characterized by an inflammatory response, is associated with a decreased VZV-specific cell-mediated immune response [124].

Reactivation of VZV is typically associated with a decline in cell-mediated immunity, placing older and immunocompromised (IC) individuals at a higher risk of developing HZ and its complications, such as postherpetic neuralgia (PHN) and VZV vasculopathy [8, 121, 122]. A systematic review reported HZ incidence rates (IR) between 6 and 8 per 1000 PY in 60 years and between 8 and 12 per 1000 PY at age 80 [2]. Studies have consistently observed significantly higher IRs in IC individuals. For instance, Weitzman et al. reported a HZ IR of 12.8 per 1000 PY in IC subjects, compared to 3.5 per 1000 PY in the general population [125, 126]. In a large analysis of German health insurance data, Hillebrand et al. found a HZ IR approximately 75% higher in IC patients than in immunocompetent individuals [126]. Another German claims data analysis revealed higher HZ incidences with decreasing immune status and higher prevalences of complications and healthcare resource utilization [8]. These findings highlight the substantial impact of HZ on immunocompromised patients, with the disease burden being most pronounced in severely immunocompromised individuals.

In the context of substantial variation in pooled OR with large confidence intervals, it is prudent to approach those estimates cautiously. To unravel the origins of this variability, further research is indispensable. For instance, exploring whether the relationship between diabetes mellitus and HZ risk is influenced by variations in glycemic control levels is of paramount importance. In addition, discrepancies in the impact of psychological disorders on HZ risk may stem from divergent disease definitions, including factors, such as acute versus chronic stress, stress severity, duration, and cumulative exposure. Future research should prioritize investigating modifiable risk factors like physical activity, dietary patterns, and environmental exposures to comprehensively understand their roles in HZ incidence [2].

Furthermore, funnel plot asymmetry can originate from variations in between-study heterogeneity, which was often unaccounted for in previous meta-analyses. Smaller studies may ensure precise treatment adherence, potentially yielding higher observed effects, while larger studies might encounter challenges in maintaining treatment fidelity, leading to lower observed effects. Thus, a thorough examination of study characteristics is warranted to assess this alternative explanation. Moreover, it is not uncommon for lower-quality studies to exhibit larger effect sizes due to increased susceptibility to bias. The resource-intensive nature of larger studies can result in more robust methodologies but can also introduce funnel plot asymmetry even in the absence of publication bias. Finally, it is important to recognize that funnel plot asymmetry can occasionally occur purely by chance, highlighting the need for a comprehensive evaluation of potential causes beyond publication bias [127].

Considering the substantial heterogeneity and diverse findings, Bayesian meta-analysis presents an alternative approach [128, 129]. It accommodates prior knowledge and handle complex relationships and uncertainties effectively, providing a versatile tool to address the multifaceted aspects of HZ risk factors. In addition, Bayesian techniques offer probabilistic statements about the effects, enhancing interpretability when traditional frequentist statistics fall short capturing uncertainties [130, 131].

### Limitations

Notwithstanding, the following limitations need to be considered when interpreting the results. All selected studies were observational studies, which can introduce bias, due to the study design. Although adjusting for key variables, those studies often relied on administrative or electronic medical records, and raising concerns about potential exposure to misclassification due to coding errors or unaccounted confounders [132]. However, cumulative risk was challenging to determine due to varying study designs and populations or study locations, since most of the studies were conducted in the northern hemisphere. An inherent limitation in this meta-analysis stems from the variability in available data across the selected studies. While efforts were made to recalculate effect estimates, not all studies provided complete raw data for this purpose. Consequently, precalculated effect estimates were used for studies with limited data availability, aiming to encompass the widest scope of evidence. It is essential to note that when one study investigated multiple risk factors or multiple types of disease (e.g. lung cancer and breast cancer), each factor was counted individually in the specific risk factors, thus potentially inflating heterogeneity. These factors underline the importance of considering the potential impact of data availability and study count methodology on the interpretation of results.

Notably, this study extends beyond previous meta-analyses, encompassing the latest literature published over the past decade. Furthermore, conducting not only classical random effects models but also using other methods, e.g. sensitivity analyses and meta-regression to quantitatively synthesize these findings and providing a robust estimate for understanding the multifaceted risk factors contributing to HZ occurrence.

## Conclusion

This meta-analysis revealed an increased risk of HZ for all considered risk factors (SLE, hematological disorders, transplantation, asthma, diabetes, cardiovascular disorders, CHF, COPD, musculoskeletal disorders, neurological disorders, digestive disorders, HIV, autoimmune disorders, cancer, mental health conditions, depression, rheumatoid arthritis, renal disorders, psoriasis, endocrine and metabolic disorders, IBD). Further analyses considering the high level of heterogeneity are needed to provide more robust pooled estimates.

### Supplementary Information

Below is the link to the electronic supplementary material.

Supplementary file 1 (PDF 394 kb)

Supplementary file 2 (PDF 111 kb)

Supplementary file 3 (PDF 69 kb)

Supplementary file 4 (PDF 185 kb)

Supplementary file 5 (PDF 3613 kb)

Supplementary file 6 (PDF 338 kb)

Supplementary file 7 (PDF 29379 kb)

Supplementary file 8 (PDF 190 kb)

Supplementary file 9 (PDF 693 kb)

## Data Availability

The Corresponding author has access to all data included into the analysis. Requests should be submitted to the corresponding author in the first instance.
