# Supplementary material for: Risk factors for herpes zoster infections: a systematic review and meta-analysis unveiling common trends and heterogeneity patterns
Source: Infection. 2024 Jan 18;52(3):1009–26. doi: 10.1007/s15010-023-02156-y (PMC11142967; doi:10.1007/s15010-023-02156-y)
Supplement: Supplementary file 3 — (PDF 69 kb) [file 15010_2023_2156_MOESM3_ESM.pdf]

S3. Search Strategy.

Databases: PubMed, EMBASE, Web of Science; Accessed on: January 1st 2023

Medline via PubMed

#	Search Term
1	herpes zoster[MeSH Terms]
2	herpes zoster[Title/Abstract]
3	zoster[Title/Abstract]
4	shingles[Title/Abstract]
**5**	**# 1 OR #2 OR #3 OR #4**
6	epidemiologic studies[MeSH Terms]
7	epidemiolog*[Title/Abstract]
8	risk[MeSH Terms]
9	odds ratio[Title/Abstract]
10	odds ratio[MeSH Terms]
11	morbidity[MeSH Terms]
12	incidence[Title/Abstract]
13	prevalence[Title/Abstract]
**14**	**#6 OR #7 OR #8 OR #9 OR #10 OR #11 OR #12 OR #13**
15	risk factor* [Title/Abstract]
16	underlying disease*[Title/Abstract]
17	underlying condition*[Title/Abstract]
18	chronic disease*[Title/Abstract]
19	comorbid*[Title/Abstract]
20	association[Title/Abstract]
21	sequelae[Title/Abstract]
**22**	**#15 OR #16 OR #17 OR #18 OR #19 OR #20 OR #21**
**23**	**#5 AND #14 AND #22**

EMBASE

#	Search Term
1	'herpes zoster'/exp
2	'herpes zoster':ab,ti
**3**	**#1 OR #2**
4	epidemiolog*:ab,ti
5	'epidemiological data'/exp
6	'risk'/exp
7	risk:ab,ti
8	'odds ratio'/exp
9	'odds ratio':ab,ti
10	incidence:ab,ti
11	prevalence:ab,ti
12	comorbid*:ab,ti
**13**	**#4 OR #5 OR #6 OR #7 OR #8 OR #9 OR #10 OR #11 OR #12**
14	'risk factor'/exp
15	'risk factor*':ab,ti
16	'underlying disease*':ab,ti
17	'underlying condition*':ab,ti
**18**	**#14 OR #15 OR #16 OR #17**
**19**	**#3 AND #13 AND #18**

Web of Science

#	Searchterm
1	TS=(herpes zoster)

2	TS=(zoster)
3	TS=(shingles)
**4**	**#1 OR #2 OR #3**
5	TS=(epidemiolog*)
6	TS=(risk)
7	TS=(odds ratio)
8	TS=(prevalence)
9	TS=(incidence)
**10**	**#5 OR #6 OR #7 OR #8 OR #9**
11	TS=(risk factor*)
12	TS=(underlying disease*)
13	TS=(underlying condition*)
14	TS=(chronic disease*)
15	TS=(comorbid*)
16	TS=(association)
17	TS=(sequelae)
**18**	**#11 OR #12 OR #13 OR #14 OR #15 OR #16 OR #17**
**19**	**#4 AND #10 AND #18**
