# Supplementary material for: Risk factors for herpes zoster infections: a systematic review and meta-analysis unveiling common trends and heterogeneity patterns
Source: Infection. 2024 Jan 18;52(3):1009–26. doi: 10.1007/s15010-023-02156-y (PMC11142967; doi:10.1007/s15010-023-02156-y)
Supplement: Supplementary file 4 — (PDF 185 kb) [file 15010_2023_2156_MOESM4_ESM.pdf]

S4. Study characteristics

Author, Publication Year	Country, Study period	Study Design	Data base	Outcome definition	Study population	HZ cases total	Risk factor	Cumulated risk group	Reported OR	Women (%)	Age Range (years)	Study Quality
Batram et al. 2021	Germany (2008-2018)	Cohort	Claims data	ICD-10	7,008,996	-	Asthma<br>CHD<br>CHF<br>COPD<br>Depression<br>Diabetes type 1<br>Diabetes type 2<br>Rheumatoid arthritis	Asthma<br>Cardiovascular disorders<br>CHF<br>COPD<br>Depression<br>Diabetes<br>Diabetes<br>Rheumatoid arthritis	1.22<br>1.03<br>1.01<br>1.05<br>1.17<br>1.01<br>0.99<br>1.44	62.00	18-100+	9
Berman et al. 2012	USA (1995-2002)	Cohort	Medical records	Symptomatic	310	76	Transplantation	Transplantation	-	-	0-21	6
Blank et al. 2012	USA (2002-2009)	Cohort	Medical records	Symptomatic	4,353	183	HIV	HIV	2.46	35.38	-	7
Cadogan et al. 2022	UK (2015)	Cohort	Survey	Self-reported	8,022	1,012	Diabetes<br>Digestive disorder<br>Mental health condition	Diabetes<br>Digestive disorders<br>Mental health condition	1.0<br>1.51<br>1.15	55.52	16-75+	7
Chakravarty et al. 2013	USA (2001-2009)	Cohort	Medical record	Self-reported	4,260	1,056	SLE	SLE	-	93.50	-	7
Chen et al. 2017	China (2009-2013)	Cohort	Medical record	Symptomatic	94	46	SLE	SLE	2.9	90.43	18-75	8
Chen et al. 2017	Taiwan (2000-2007)	Cohort	Claims data	ICD-9	600,891	2,488	Asthma	Asthma	-	41.14	0-18	8
Chen et al. 2016	Taiwan (2003-2011)	Cohort	Claims data	ICD-9	23,680	280	Diabetes type 1	Diabetes	-	53.48	-	9
Chen et al. 2015	Taiwan (2001-2008)	Cohort	Claims data	ICD-9	30,009	1,808	Primary Sjögren's syndrome	Autoimmune disorders	-	85.91	20-60+	8
Chen et al. 2014	USA (2005-2009)	Cohort	Claims data	ICD-9	51,022,838	435,378	HIV<br>Psoriasis<br>Multiple sclerosis<br>SLE<br>IBD<br>Bone marrow transplant<br>Solid organ transplant<br>RA<br>Cancer	HIV<br>Psoriasis<br>Autoimmune disorders<br>SLE<br>IBD<br>Transplantation<br>Transplantation<br>RA<br>Cancer	3.15<br>1.53<br>1.73<br>2.94<br>1.80<br>6.05<br>3.27<br>2.41<br>2.51	57.93	18-65+	8
Chen et al. 2013	Taiwan (2002-2008)	Cohort	Claims data	ICD-9	82,458	3,187	Peptic ulcer disease	Digestive disorders	-	46.94	18-65+	8
Choi et al. 2019	Korea (2002-2013)	Case-control	Claims data	ICD-10	291,390	18,656	Depression	Depression	-	65.70	20-85+	8
Chung et al. 2016	Taiwan (2002-2005)	Cohort	Claims data	ICD-10	131,001	5,001	Sleep disorder	Mental health condition	-	61.31	20+	7
Esteban-Vasallo et al. 2014	Spain (2009-2012)	Cohort	Claims data	-	5,244,402	81,541	Asthma<br>COPD<br>Ischemic heart disease<br>Heart failure<br>Other cardiovascular disease<br>Diabetes<br>HIV	Asthma<br>COPD<br>Cardiovascular disorders<br>CHF<br>Cardiovascular disorders<br>Diabetes<br>HIV	1.42<br>2.20<br>2.12<br>2.12<br>2.07<br>1.92<br>2.47	-	18-75+	7

							Other immunodeficiency	Autoimmune disorders	2.45			
							Cancer	Cancer	1.95			
Forbes et al. 2014	UK (2000-2011)	Case-control	Data linkage	ICD-10	694,295	144,959	RA	RA	1.46	60.68	18-90+	8
							SLE	SLE	1.72			
							IBD	IBD	1.36			
							COPD	COPD	1.32			
							Asthma	Asthma	1.21			
							Chronic kidney disease	Renal disorders	1.14			
							Depression	Depression	1.15			
							Diabetes	Diabetes	1.02			
							HIV	HIV	5.07			
							Leukaemia	Hematological disorders	1.78			
							Lymphoma	Hematological disorders	3.90			
							Myeloma	Hematological disorders	2.16			
							Haematopoietic stem cell transplantation	Transplantation	13.46			
							Other immunodeficiency	Autoimmune disorders	1.57			
Gebo et al. 2005	USA (1997-2001)	Cohort	Medical records	Symptomatic	2,543	158	HIV	HIV	1.67	37.00	23-58	7
Glesby et al. 2004	USA (1994-1995)	Cohort	Medical records	Self-reported	2,321	337	HIV	HIV	0.9	100.00	34-45	8
Gourishankar et al. 2003	Canada (1994-1999)	Cohort	Medical records	Symptomatic	869	75	Organ transplant	Transplantation	-	35.40	16-74	7
Hansson et al. 2017	UK (2000-2015)	Case-control	Data linkage	ICD-10	64,833	16,129	Any solid malignancy	Cancer	1.19	60.56	18-90+	7
							Any haematological malignancy	Cancer	2.42			
Hata et al. 2011	Japan (2001-2007)	Cohort	Medical records	ICD-10	55,492	759	Brain tumor	Cancer	3.69	53.16	20-103	7
							Lung cancer	Cancer	2.17			
							Breast cancer	Cancer	2.34			
							Esophageal cancer	Cancer	4.05			
							Gastric cancer	Cancer	1.92			
							Colorectal cancer	Cancer	1.82			
							Gynecologic cancer	Cancer	3.34			
							Malignant lymphoma	Cancer	8.39			
							SLE	SLE	4.11			
							RA	RA	2.03			
							Sjörgens disease	Autoimmune disorders	1.30			
							Diabetes	Diabetes	2.38			
							Hypertension	Cardiovascular disorders	1.93			
							Renal failure	Renal disorders	2.21			
Heymann et al. 2008	Israel (2002-2006)	Case-control	Claims data	ICD-9	111,145	22,250	Diabetes	Diabetes	1.53	55.57	25-75+	8
							HIV/AIDS	HIV	1.16			
							Leukaemia	Hematological disorders	3.55			
							Lymphoma	Hematological disorders	1.59			
Hsieh et al. 2022	Taiwan (2000-2017)	Cohort	Claims data	ICD-9	40,284	1,207	Polycystic ovary syndrome	Endocrine and metabolic disorders	-	100.00	20-65+	8
Hsu et al. 2021	Taiwan (2000-2012)	Case-control	Claims data	ICD-9	30,502	1,631	Lateral epicondylitis	Musculoskeletal disorders	-	61.30	20-65+	7
Hsu et al. 2021	Taiwan (2000-2012)	Case-control	Claims data	ICD-9	8,390	360	de Quervain syndrome	Musculoskeletal disorders	-	76.97	20-65+	7

Hsu et al. 2021	Taiwan (2000-2012)	Case-control	Claims data	ICD-9	23,645	1,099	Plantar facial fibromatosis	Musculoskeletal disorders	-	63.22	20-65+	7
Hu et al. 2013	Taiwan (1999-2008)	Case-control	Medical records	ICD-9	130	65	Lymphoma	Hematological disorders	2.87	-	-	8
Imafuku et al. 2019	Japan (2005-2014)	Cohort	Claims data	ICD-10	2,778,476	27,995	Autoimmune thyroiditis	Endocrine and metabolic disorders	1.51	45.24	18-65+	8
							Congenital immune deficiency	Autoimmune disorders	1.29			
							Hematological malignancies	Cancer	5.08			
							Hematopoietic stem cell transplant	Transplantation	19.77			
							IBD	IBD	1.60			
							Psoriasis	Psoriasis	1.18			
							RA	RA	2.11			
							SLE	SLE	3.37			
							Solid organ malignancies	Cancer	1.64			
							Vasculitis	Autoimmune disorders	1.18			
							Other autoimmune disease	Autoimmune disorders	2.19			
							Asthma	Asthma	1.15			
							Chronic hepatitis	Autoimmune disorders	1.24			
							COPD	COPD	1.39			
							Depression	Depression	1.31			
							Heart failure	CHF	1.34			
							Ischemic heart disease	Cardiovascular disorders	1.64			
							Osteoarthritis	Musculoskeletal disorders	1.53			
							Osteoporosis	Musculoskeletal disorders	2.18			
							Stroke	Neurological disorders	1.58			
							Renal failure	Renal disorders	1.11			
							Diabetes type 2	Diabetes	1.38			
							Viral hepatitis	Endocrine and metabolic disorders	1.61			
Imafuku et al. 2020	Japan (2005-2014)	Cohort	Claims data	ICD-10	2,778,476	27,995	Autoimmune thyroiditis	Endocrine and metabolic disorders	1.52	45.24	18-65+	7
							Congenital immune deficiency	Autoimmune disorders	1.30			
							Hematological malignancies	Cancer	5.12			
							Hematopoietic stem cell transplant	Transplantation	19.91			
							IBD	IBD	1.61			
							Psoriasis	Psoriasis	1.19			
							RA	RA	2.13			
							SLE	SLE	3.40			
							Solid organ malignancies	Cancer	1.66			
							Vasculitis	Autoimmune disorders	1.18			
							Asthma	Asthma	1.16			
							Chronic hepatitis	Autoimmune disorders	1.25			
							Other autoimmune disease	Autoimmune disorders	2.20			

							COPD	COPD	1.40			
							Depression	Depression	1.31			
							Heart failure	CHF	1.35			
							Ischemic heart disease	Cardiovascular disorders	1.66			
							Osteoarthritis	Musculoskeletal disorders	1.53			
							Osteoporosis	Musculoskeletal disorders	2.20			
							Stroke	Neurological disorders	1.60			
							Renal failure	Renal disorders	1.11			
							Diabetes type 2	Diabetes	1.39			
							Viral hepatitis	Endocrine and metabolic disorders	1.62			
Jin et al. 2020	Korea (2002-2013)	Case-control	Claims data	ICD-10	255,604	21,066	Pepetic ulcer	Digestive disorders	-	51.51	20-85+	7
Joesoef et al. 2012	USA (2007)	Case-control	Data linkage	ICD-9	675,350	59,173	COPD	COPD	1.35	54.24	20-64	7
							Depression	Depression	1.52			
							Diabetes	Diabetes	1.06			
							Hypothyroidism	Endocrine and metabolic disorders	1.16			
							Osteoarthritis	Musculoskeletal disorders	1.24			
							CAD	Cardiovascular disorders	1.17			
							Hyperlipidemia	Endocrine and metabolic disorders	1.11			
Ke et al. 2016	Taiwan (2005-2011)	Case-control	Claims data	ICD-9	126,725	25,345	Diabetes	Diabetes	1.24	53.48	18-70+	7
Ke et al. 2020	Taiwan (2000-2012)	Cohort	Data linkage	ICD-9	98,046	6,880	Sciatica	Neurological disorders	-	55.85	-	8
Khan et al. 2018	USA (2000-2016)	Cohort	Claims data	ICD-9	40,142	1,185	Ulcerative colitis	Digestive disorders	-	6.97	-	7
Khan et al. 2018	USA (2000-2016)	Cohort	Claims data	ICD-9	43,879	1,288	Crohns disease	Digestive disorders	-	6.84	-	7
Khan et al. 2018	USA (2000-2016)	Cohort	Claims data	ICD-9	48,511	1,397	IBD	IBD	-	6.83	-	7
Kim et al. 2013	USA (1996-2001)	Case-control	Data linkage	ICD-9	554	277	Asthma	Asthma	2.09	52.71	0-17	7
Kobayashi et al. 2019	Japan (2005-2016)	Cohort	Medical records	ICD-10	81,466	59	Diabetes	Diabetes	1.06	51.34	-	7
Kwon et al. 2016	USA (2010-2011)	Case-control	Medical records	ICD-9	1,113	371	Asthma	Asthma	1.70	66.31	-	8
La Habel et al. 2013	USA (2001-2005)	Cohort	Claims data	ICD-9	14,670	424	Hematologic malignancy	Cancer	-	42.46	18-80+	8
Lai et al. 2021	Taiwan (2000-2013)	Cohort	Claims data	ICD-9	32,380	1,644	Cirrhosis	Digestive disorders	-	32.17	20-84	8
Lai et al. 2020	Taiwan (2005-2012)	Cohort	Claims data	ICD-9	49,965	2,129	Chronic kidney disease	Renal disorders	-	43.22	20-84	8
Lai et al. 2020	Taiwan (2000-2012)	Cohort	Claims data	ICD-9	3,186	179	Splenectomy	Hematological disorders	-	38.98	20-84	8
Lai et al. 2019	Taiwan (2000-2012)	Cohort	Claims data	ICD-9	135,074	7,733	Diabetes	Diabetes	-	44.62	20-84	8
Lai et al. 2018	Taiwan (2000-2012)	Cohort	Claims data	ICD-9	6,567	243	Chronic pancreatitis	Digestive disorders	-	31.17	20-84	8
Lasserre et al. 2012	France (2009-2010)	Case-control	Questionnaire	Self-reported	750	250	Asthma	Asthma	0.78	59.00	50-91	7
							Diabetes	Diabetes	1.95			
							Arterial hypertension	Cardiovascular disorders	0.84			
							Heart failure	CHF	0.74			

							Cerebrovascular disease	Cardiovascular disorders	1.20			
							COPD	COPD	0.63			
							Arthritis	Musculoskeletal disorders	1.12			
							Rheumatism	Musculoskeletal disorders	0.67			
							Headache	Neurological disorders	1.09			
							Gastric ulcer	Digestive disorders	2.09			
							HIV/AIDS	HIV	1.00			
							Osteoporosis	Musculoskeletal disorders	0.81			
							Autoimmune disease	Autoimmune disorders	1.25			
							Cancer	Cancer	1.26			
Lee et al. 2018	Taiwan (2012-2015)	Cohort	Medical records	-	826	54	HIV	HIV	2.03	3.00	-	8
Lin et al. 2012	Taiwan (1996-2008)	Case-control	Data linkage	ICD-9	95,383	6,623	Chronic kidney disease	Renal disorders	-	43.60	18-65+	9
Lin et al. 2014	Taiwan (1996-2008)	Case-control	Claims data	ICD-9	4,999	1,144	End stage renal disease	Renal disorders	-	48.31	18-64+	8
Long et al. 2013	USA (1997-2009)	Cohort	Data linkage	ICD-9	543,020	7,017	IBD	IBD	-	54.97	31-52	8
Ludvigsson et al. 2017	Sweden (1969-2008)	Cohort	Registry	ICD-10, ICD-9	173,591	653	Coeliac disease	Digestive disorders	-	61.89	18-60+	9
Marin et al. 2016	USA (2010-2011)	Case-control	Data linkage	ICD-9	895	384	Stress	Mental health condition	2.80	64.25	50-95	8
							Depression	Depression	3.81			
Min et al. 2021	Korea (2002-2013)	Case-control	Data linkage	ICD-10	55,045	3,229	Psoriasis	Psoriasis	-	43.79	20-85+	7
Min et al. 2019	Korea (2002-2013)	Case-control	Data linkage	ICD-10	136,984	6,225	Osteoporosis	Musculoskeletal disorders	1.17	85.78	50-85+	8
Mok et al. 2022	China (2019)	Cohort	Medical records	Symptomatic	1,479	219	SLE	SLE	-	88.30	-	6
Munoz-Quiles et al. 2018	Spain (2009-2014)	Cohort	Claims data	ICD-9	2,289,485	69,438	COPD	COPD	-	53.63	50-80+	7
Peng et al. 2017	Taiwan (1997-2011)	Cohort	Claims data	ICD-9	80,138	2,404	Asthma	Asthma	-	48.97	0-61+	9
Ryu et al. 2021	Korea (2009-2013)	Cohort	Claims data	KCD-6	285,792	1,869	Rheumatoid arthritis	Rheumatoid arthritis	-	69.04	20-70+	7
							SLE	SLE				
Schmidt et al. 2021	Denmark (2010-2014)	Cohort	Data linkage	Symptomatic	77,310	1,686	Stress	Mental health condition	-	48.90	40-80+	9
Schmidt et al. 2018	Denmark (1997-2013)	Case-control	Registry	Antiviral prescription	953,355	190,671	Depression	Depression	1.16	65.83	18-90+	5
							Anxiety	Mental health condition	1.71			
							Stress	Mental health condition	1.37			
	UK (2000-2013)	Case-control	Data linkage	Symptomatic	516,146	109,331	Depression	Depression	1.18	60.63	18-90+	
							Anxiety	Mental health condition	1.23			
							Stress	Mental health condition	1.22			
Schmidt et al. 2017	Denmark (1997-2013)	Case-control	Registry	Antiviral prescription	953,355	190,671	Partner bereavement	Mental health condition	1.05	65.83	40-90+	5
	UK (2000-2013)	Case-control	Data linkage	Symptomatic	727,085	150,207	Partner bereavement	Mental health condition	1.01	61.14	40-90+	
Seo et al. 2018	Korea (2006-2013)	Case-control	Claims data	ICD-10	17,682	1,985	Heart failure	CHF	-	63.90	-	7
					25,008	1,424	Myocardial infarction	Cardiovascular disorders	-			
					41,190	2,800	Ischemic stroke	Cardiovascular disorders	-			

Sinayobye et al. 2015	Rwanda (2005)	Cohort	Questionnaire	Self-reported	936	89	HIV	HIV	2.43	100.00	-	6
Smitten et al. 2007	UK (1990-2001)	Case-control	Medical records	OXMIS code	13,752	1,719	RA	RA	1.27	72.67	18-65+	9
	USA (1998-2002)		Claims data	ICD-9	12,888	1,611			1.37	73.39		
	UK (1990-2001)	Cohort	Medical records	OXMIS code	538,621	10,928	RA	RA	-	71.70		8
	USA (1998-2002)		Claims data	ICD-9	1,122,272	8,192			-	73.10		
Soh et al. 2019	Korea (2010-2013)	Cohort	Claims data	ICD-10	63,102	2,321	Crohns disease	Digestive disorders	-	27.81	0-60+	8
					117,498	6,422	Ulcerative colitis	Digestive disorders	-	90.17		
					180,600	8,743	IBD	IBD	-	36.41		
Suaya et al. 2014	USA (2005-2009)	Cohort	Claims data	ICD-9	51,007,975	420,515	Diabetes	Diabetes	-	50.10	18-65+	8
Takao et al. 2018	Japan (2008-2009)	Cohort	Questionnaire	Self-reported	12,522	400	Very high mental stress	Mental health condition	-	54.70	50-103	6
							Very high sense of purpose in life	Mental health condition	-			
							Negative life events	Mental health condition	-			
Thomson-Leduc et al. 2022	USA (2007-2009)	Cohort	Data linkage	-	9,805,492	75,954	COPD	COPD	-	53.31	40-80+	7
Tsai et al. 2015	Taiwan (2000-2006)	Case-control	Claims data	ICD-9	9,432	662	Dermatomyositis/ polymyositis	Autoimmune disorders	-	67.00	20-65+	8
Tsai et al. 2017	Taiwan (1998-2011)	Case-control	Claims data	ICD-9	20,385	590	Psoriasis	Psoriasis	-	44.34	20-60+	8
Tung et al. 2015	Taiwan (1996-2010)	Cohort	Claims data	ICD-9	62,319	3,087	Brain injury	Neurological disorders	-	41.04	18-70+	7
Tung et al. 2020	Taiwan (1996-2010)	Cohort	Claims data	ICD-9	41,102	1,301	Stroke	Neurological disorders	-	44.33	18-70+	7
Veetil et al. 2013	USA (1980-2007)	Cohort	Medical records	ICD-9	1,626	118	RA	RA	-	68.39	18-94	8
Wang et al. 2020	Taiwan (1997-2013)	Cohort	Claims data	ICD-9	14,095	488	Ankylosing spondylitis	Musculoskeletal disorders	-	38.77	0-60+	9
Weitzmann et al. 2013	Israel (2006-2010)	Cohort	Claims data	ICD-9	2,020,709	28,977	Diabetes	Diabetes	-	82.64	0-85+	7
							HIV/AIDS	HIV	-			
							Transplantation	Transplantation	-			
Wi et al. 2015	USA (1996-2001)	Case-control	Medical records	ICD-9	459	77	Asthma	Asthma	-	52.94	3-18	8
Wu et al. 2015	Taiwan (1997-2010)	Cohort	Claims data	ICD-9	19,140	211	Heart failure	CHF	-	46.77	18-80+	8
Wu et al. 2012	Taiwan (2004-2005)	Cohort	Claims data	ICD-9	79,926	1,602	Chronic kidney disease	Renal disorders	-	51.77	18-70+	9
Yang et al. 2011	Taiwan (2004-2005)	Cohort	Claims data	ICD-9	42,430	1,080	COPD	COPD	-	30.97	50-70+	8
Yang et al. 2011	Taiwan (2004-2006)	Cohort	Claims data	ICD-9	211,700	3,441	Psychiatric disorder	Mental health condition	-	60.93	18-70+	8
Yanni et al. 2018	UK (2000-2012)	Cohort	Claims data	ICD-9	622,900	21,146	Hematological stem cell transplantation	Transplantation	-	56.11	18-80+	8
							HIV/AIDS	HIV	-			
							Solid organ transplant	Transplantation	-			
							SLE	SLE	-			

							Autoimmune thyroiditis	Endocrine and metabolic disorders	-			
							Multiple sclerosis	Autoimmune disorders	-			
							Polymyalgia rheumatica	Musculoskeletal disorders				
							Hematological malignancies	Cancer	-			
							IIBD	IBD	-			
							Rheumatoid arthritis	Rheumatoid arthritis	-			
							End stage renal disease	Renal disorders	-			
							Psoriasis	Psoriasis	-			
							Solid organ malignancies	Cancer	-			
Yu et al. 2021	Taiwan (1996-2013)	Cohort	Claims data	ICD-9	8,732	60	Polycystic kidney disease	Renal disorders	-	43.09	20-65+	8
Zhang et al. 2013	USA (2006-2007)	Case-control	Claims data	ICD-9	184,473	16,771	Trauma	Mental health condition	1.09	-	65+	8
