# Supplementary material for: Risk factors for herpes zoster infections: a systematic review and meta-analysis unveiling common trends and heterogeneity patterns
Source: Infection. 2024 Jan 18;52(3):1009–26. doi: 10.1007/s15010-023-02156-y (PMC11142967; doi:10.1007/s15010-023-02156-y)

S5. Forest plots for each subgroup.

Asthma

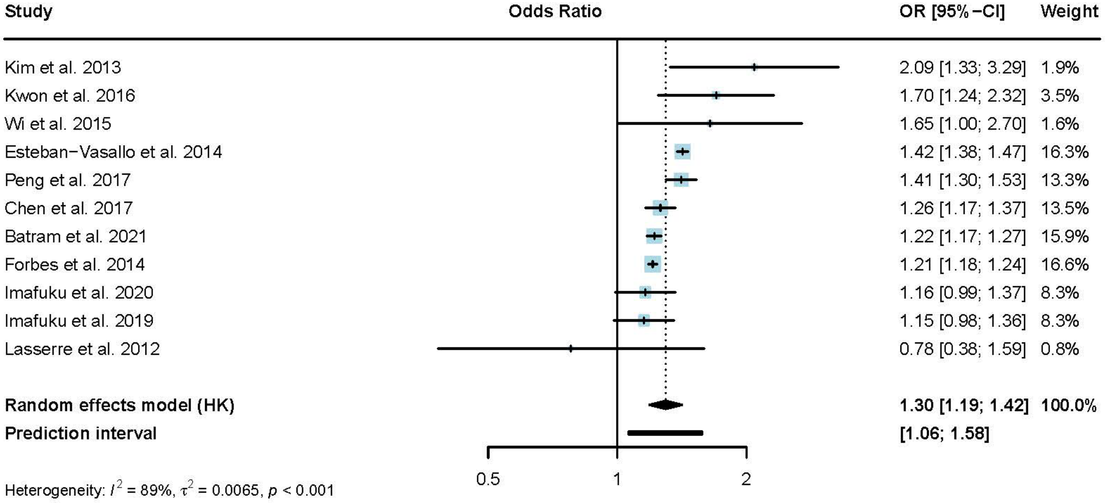

Study	OR [95%−CI]	Weight
Kim et al. 2013	2.09 [1.33; 3.29]	1.9%
Kwon et al. 2016	1.70 [1.24; 2.32]	3.5%
Wi et al. 2015	1.65 [1.00; 2.70]	1.6%
Esteban−Vasallo et al. 2014	1.42 [1.38; 1.47]	16.3%
Peng et al. 2017	1.41 [1.30; 1.53]	13.3%
Chen et al. 2017	1.26 [1.17; 1.37]	13.5%
Batram et al. 2021	1.22 [1.17; 1.27]	15.9%
Forbes et al. 2014	1.21 [1.18; 1.24]	16.6%
Imafuku et al. 2020	1.16 [0.99; 1.37]	8.3%
Imafuku et al. 2019	1.15 [0.98; 1.36]	8.3%
Lasserre et al. 2012	0.78 [0.38; 1.59]	0.8%
**Random effects model (HK)**	**1.30 [1.19; 1.42]**	**100.0%**
**Prediction interval**	**[1.06; 1.58]**	

Heterogeneity: I^2 = 89%, τ^2 = 0.0065, p < 0.001

Cardiovascular Disorders

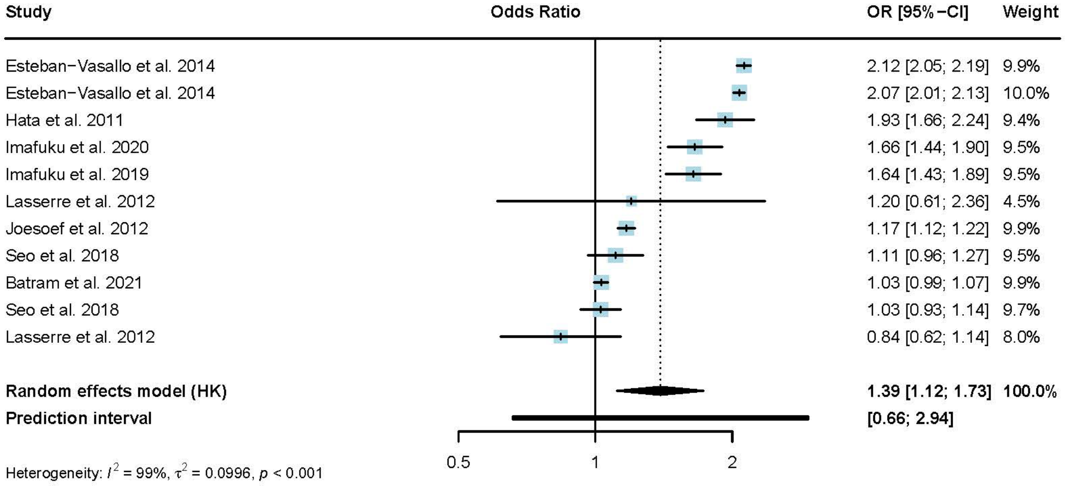

Study	OR [95%−CI]	Weight
Esteban−Vasallo et al. 2014	2.12 [2.05; 2.19]	9.9%
Esteban−Vasallo et al. 2014	2.07 [2.01; 2.13]	10.0%
Hata et al. 2011	1.93 [1.66; 2.24]	9.4%
Imafuku et al. 2020	1.66 [1.44; 1.90]	9.5%
Imafuku et al. 2019	1.64 [1.43; 1.89]	9.5%
Lasserre et al. 2012	1.20 [0.61; 2.36]	4.5%
Joesoef et al. 2012	1.17 [1.12; 1.22]	9.9%
Seo et al. 2018	1.11 [0.96; 1.27]	9.5%
Batram et al. 2021	1.03 [0.99; 1.07]	9.9%
Seo et al. 2018	1.03 [0.93; 1.14]	9.7%
Lasserre et al. 2012	0.84 [0.62; 1.14]	8.0%
**Random effects model (HK)**	**1.39 [1.12; 1.73]**	**100.0%**
**Prediction interval**	**[0.66; 2.94]**	

Heterogeneity: I^2 = 99%, τ^2 = 0.0996, p < 0.001

Chronic heart failure (CHF)

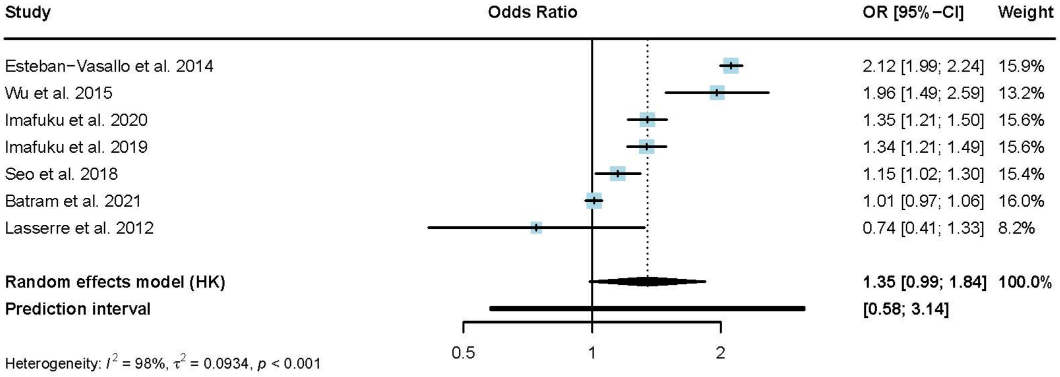

Study	OR [95%−CI]	Weight
Esteban−Vasallo et al. 2014	2.12 [1.99; 2.24]	15.9%
Wu et al. 2015	1.96 [1.49; 2.59]	13.2%
Imafuku et al. 2020	1.35 [1.21; 1.50]	15.6%
Imafuku et al. 2019	1.34 [1.21; 1.49]	15.6%
Seo et al. 2018	1.15 [1.02; 1.30]	15.4%
Batram et al. 2021	1.01 [0.97; 1.06]	16.0%
Lasserre et al. 2012	0.74 [0.41; 1.33]	8.2%
**Random effects model (HK)**	**1.35 [0.99; 1.84]**	**100.0%**
**Prediction interval**	**[0.58; 3.14]**	

Heterogeneity: I^2 = 98%, τ^2 = 0.0934, p < 0.001

Chronic obstructive pulmonary disorder (COPD)

Study	OR [95%−CI]	Weight
Thomson−Leduc et al. 2022	5.69 [5.55; 5.84]	10.4%
Esteban−Vasallo et al. 2014	2.20 [2.11; 2.28]	10.4%
Yang et al. 2011	1.72 [1.51; 1.96]	10.3%
Imafuku et al. 2020	1.40 [1.18; 1.67]	10.1%
Imafuku et al. 2019	1.39 [1.17; 1.66]	10.1%
Joesoef et al. 2012	1.35 [1.23; 1.48]	10.3%
Forbes et al. 2014	1.32 [1.28; 1.36]	10.4%
Munoz−Quiles et al. 2018	1.21 [1.18; 1.24]	10.4%
Batram et al. 2021	1.05 [1.01; 1.09]	10.4%
Lasserre et al. 2012	0.63 [0.30; 1.31]	7.0%
**Random effects model (HK)**	**1.55 [1.04; 2.31]**	**100.0%**
**Prediction interval**	**[0.42; 5.67]**	

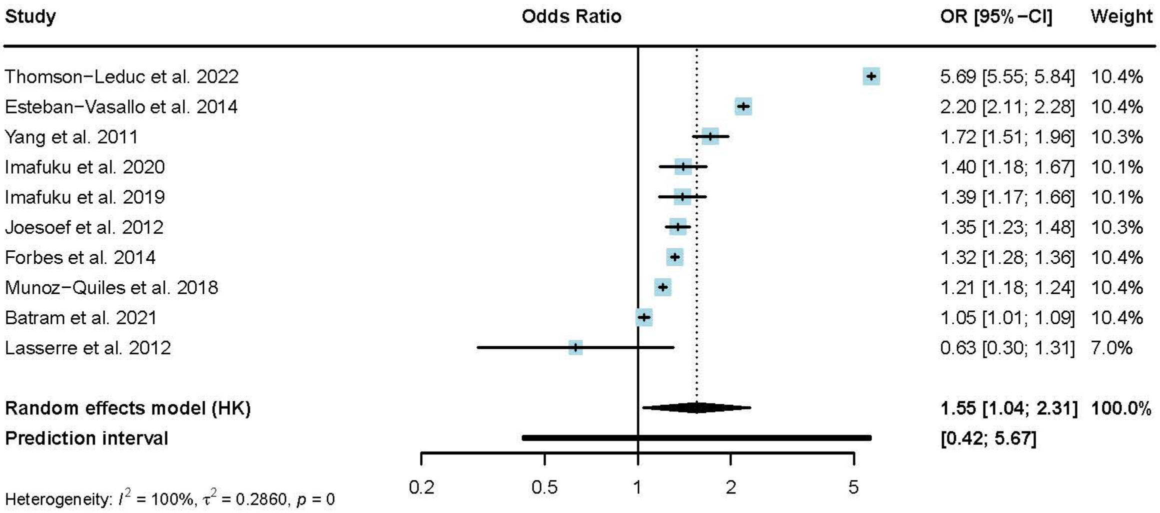

Heterogeneity: $I^2 = 100\%$, $\tau^2 = 0.2860$, $p = 0$

Depression

Study	OR [95%−CI]	Weight
Marin et al. 2016	3.81 [2.15; 6.76]	2.4%
Joesoef et al. 2012	1.52 [1.46; 1.58]	13.4%
Imafuku et al. 2019	1.31 [1.21; 1.43]	12.3%
Imafuku et al. 2020	1.31 [1.20; 1.43]	12.3%
Schmidt et al. 2018	1.18 [0.97; 1.43]	8.8%
Batram et al. 2021	1.17 [1.14; 1.21]	13.5%
Schmidt et al. 2018	1.16 [0.99; 1.35]	10.2%
Forbes et al. 2014	1.15 [1.12; 1.18]	13.6%
Choi et al. 2019	1.09 [1.05; 1.13]	13.4%
**Random effects model (HK)**	**1.27 [1.08; 1.49]**	**100.0%**
**Prediction interval**	**[0.90; 1.77]**	

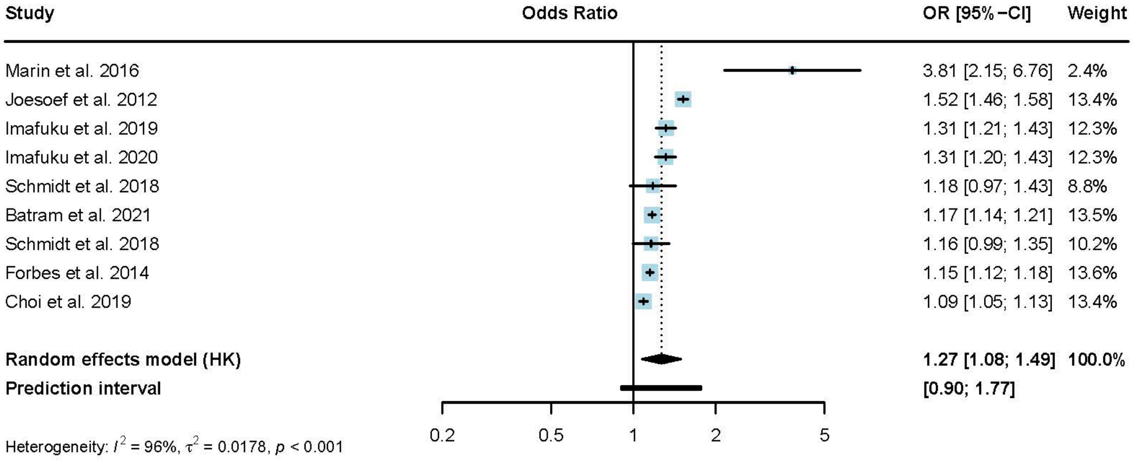

Heterogeneity: $I^2 = 96\%$, $\tau^2 = 0.0178$, $p < 0.001$

Diabetes

Study	OR [95%−CI]	Weight
Hata et al. 2011	2.38 [2.05; 2.76]	6.0%
Lasserre et al. 2012	1.95 [1.21; 3.13]	4.5%
Esteban−Vasallo et al. 2014	1.92 [1.88; 1.97]	6.2%
Chen et al. 2016	1.76 [1.36; 2.27]	5.6%
Suaya et al. 2014	1.75 [1.74; 1.77]	6.2%
Heymann et al. 2008	1.53 [1.45; 1.62]	6.2%
Imafuku et al. 2020	1.39 [1.24; 1.55]	6.1%
Imafuku et al. 2019	1.38 [1.23; 1.54]	6.1%
Ke et al. 2016	1.24 [1.20; 1.28]	6.2%
Lai et al. 2019	1.09 [1.04; 1.16]	6.2%
Joesoef et al. 2012	1.06 [1.03; 1.09]	6.2%
Kobayashi et al. 2019	1.06 [0.63; 1.78]	4.2%
Forbes et al. 2014	1.02 [1.00; 1.04]	6.2%
Batram et al. 2021	1.01 [0.98; 1.04]	6.2%
Cadogan et al. 2022	1.00 [0.80; 1.26]	5.7%
Batram et al. 2021	0.99 [0.91; 1.08]	6.1%
Weitzmann et al. 2013	0.44 [0.40; 0.48]	6.1%
**Random effects model (HK)**	**1.26 [1.03; 1.54]**	**100.0%**
**Prediction interval**	**[0.54; 2.93]**	

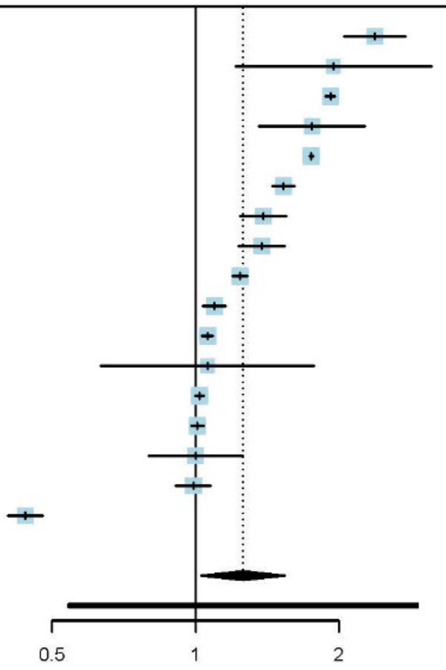

Heterogeneity: $I^2 = 100\%$, $\tau^2 = 0.1483$, $p = 0$

Rheumatoid arthritis (RA)

Study	OR [95%−CI]	Weight
Smitten et al. 2007	2.48 [2.36; 2.62]	8.1%
Chen et al. 2014	2.41 [2.37; 2.46]	8.1%
Imafuku et al. 2020	2.13 [1.64; 2.77]	7.2%
Imafuku et al. 2019	2.11 [1.62; 2.75]	7.2%
Hata et al. 2011	2.03 [1.66; 2.49]	7.5%
Smitten et al. 2007	2.02 [1.91; 2.13]	8.1%
Yanni et al. 2018	1.75 [1.67; 1.84]	8.1%
Forbes et al. 2014	1.46 [1.40; 1.52]	8.1%
Batram et al. 2021	1.44 [1.34; 1.54]	8.1%
Smitten et al. 2007	1.37 [1.12; 1.68]	7.5%
Smitten et al. 2007	1.27 [1.05; 1.54]	7.6%
Veetil et al. 2013	0.96 [0.66; 1.40]	6.4%
Ryu et al. 2021	0.71 [0.64; 0.78]	8.0%
**Random effects model (HK)**	**1.62 [1.29; 2.02]**	**100.0%**
**Prediction interval**	**[0.71; 3.69]**	

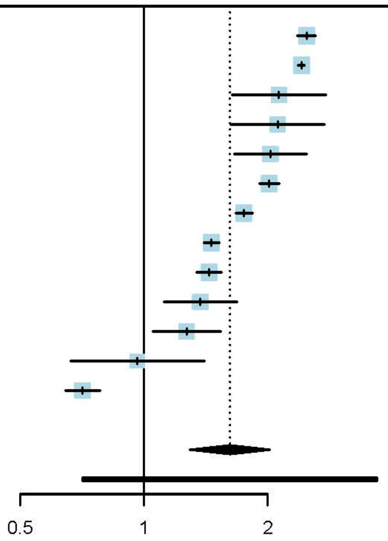

Heterogeneity: $I^2 = 99\%$, $\tau^2 = 0.1295$, $p < 0.001$

Mental health condition

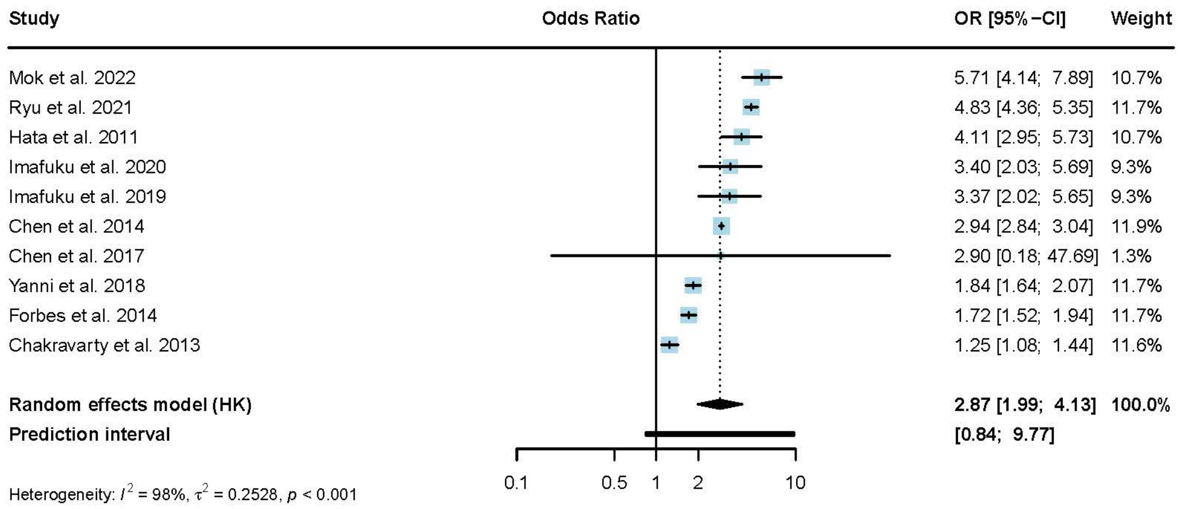

Systemic lupus erythematosus (SLE)

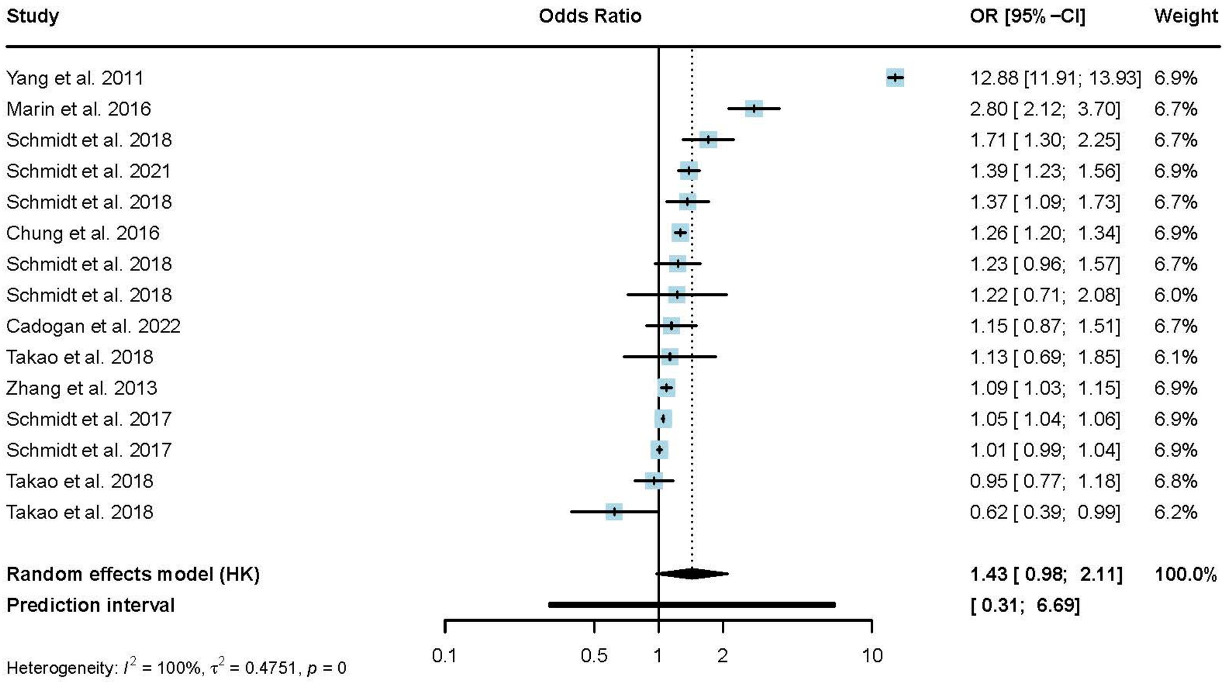

Autoimmune disorders

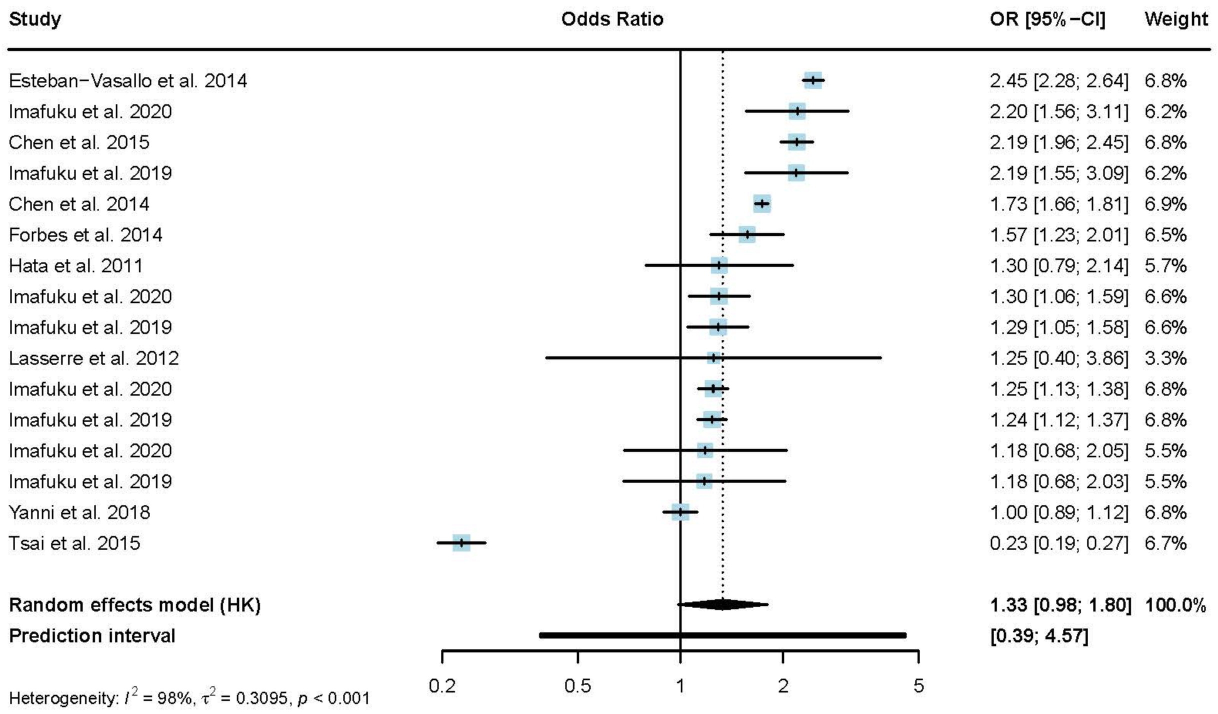

Psoriasis

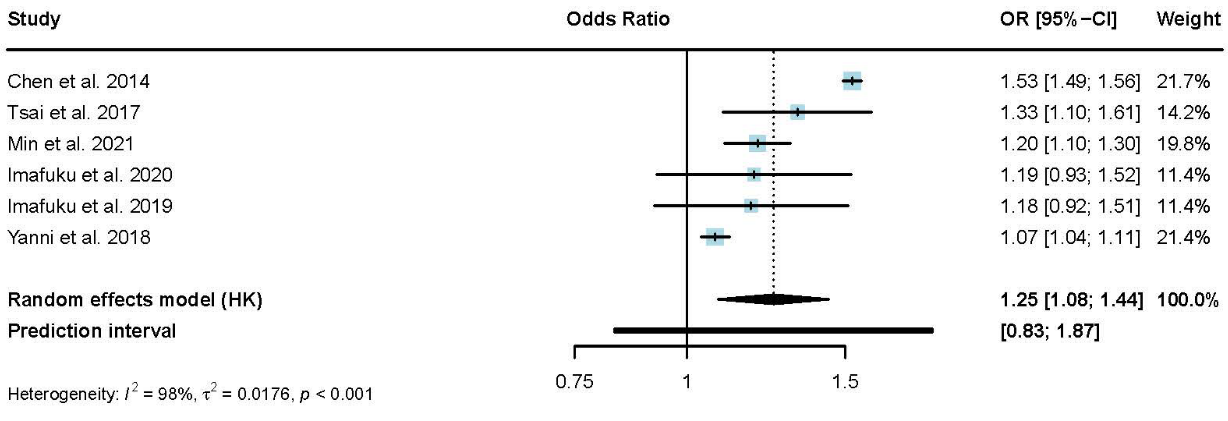

Inflammatory bowel disease (IBD)

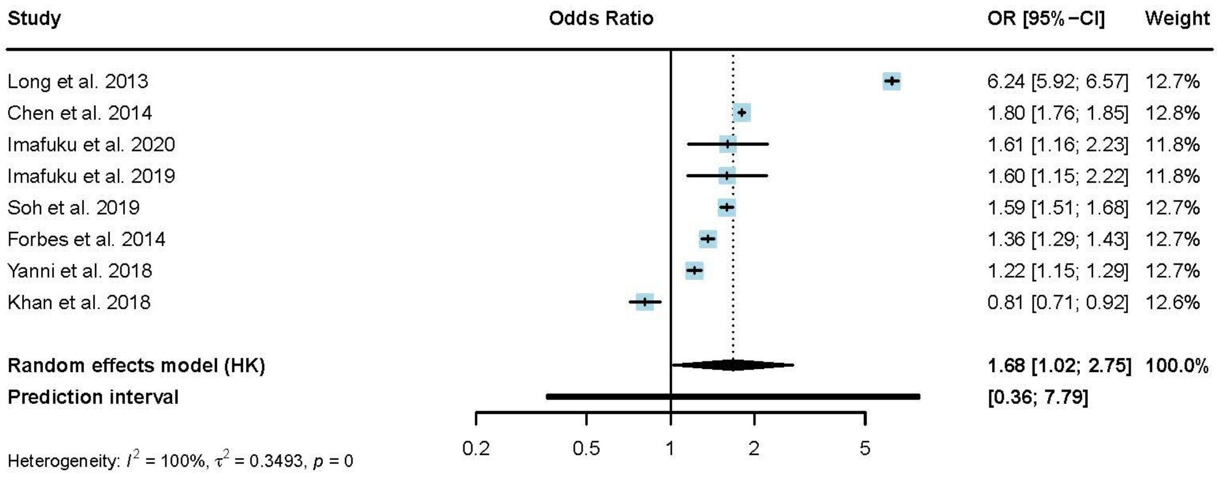

Renal disorders

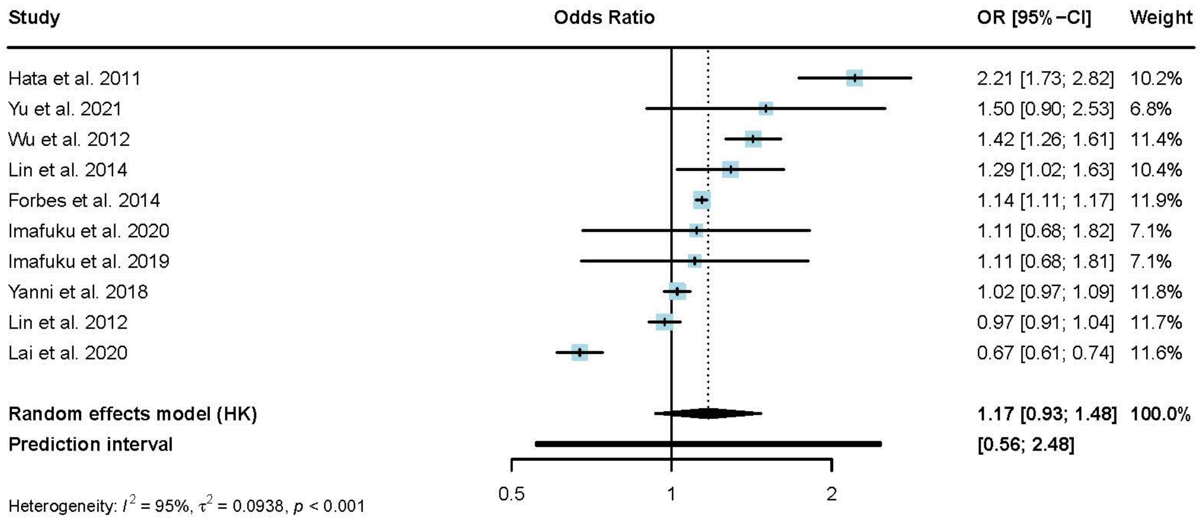

Hematological disorders

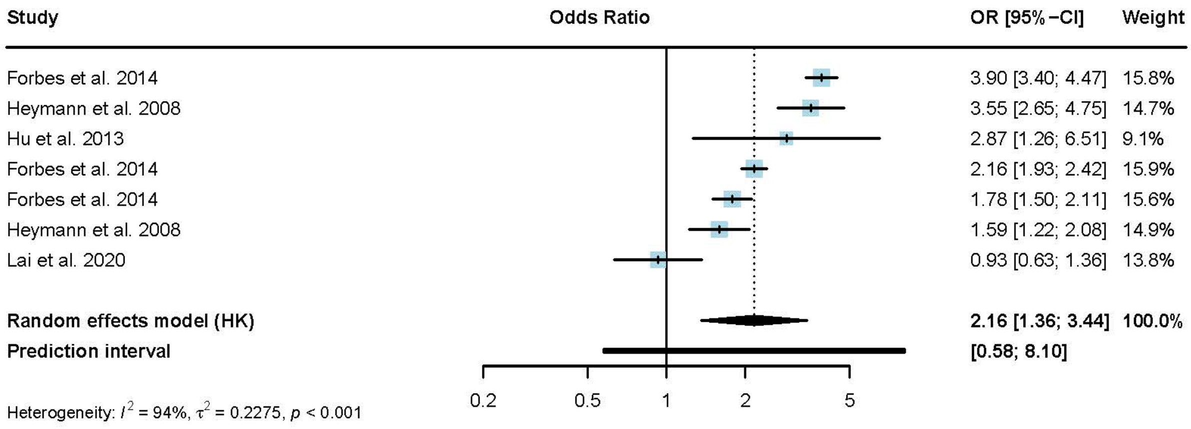

Endocrine and metabolic disorders

Study	OR [95%−CI]	Weight
Imafuku et al. 2020	1.62 [1.37; 1.92]	12.1%
Imafuku et al. 2019	1.61 [1.36; 1.90]	12.1%
Imafuku et al. 2020	1.52 [1.22; 1.89]	11.2%
Imafuku et al. 2019	1.51 [1.21; 1.87]	11.2%
Joesoef et al. 2012	1.16 [1.12; 1.20]	13.9%
Joesoef et al. 2012	1.11 [1.09; 1.13]	13.9%
Hsieh et al. 2022	1.10 [0.98; 1.24]	13.0%
Yanni et al. 2018	0.83 [0.73; 0.96]	12.6%
**Random effects model (HK)**	**1.26 [1.04; 1.54]**	**100.0%**
**Prediction interval**	**[0.70; 2.27]**	

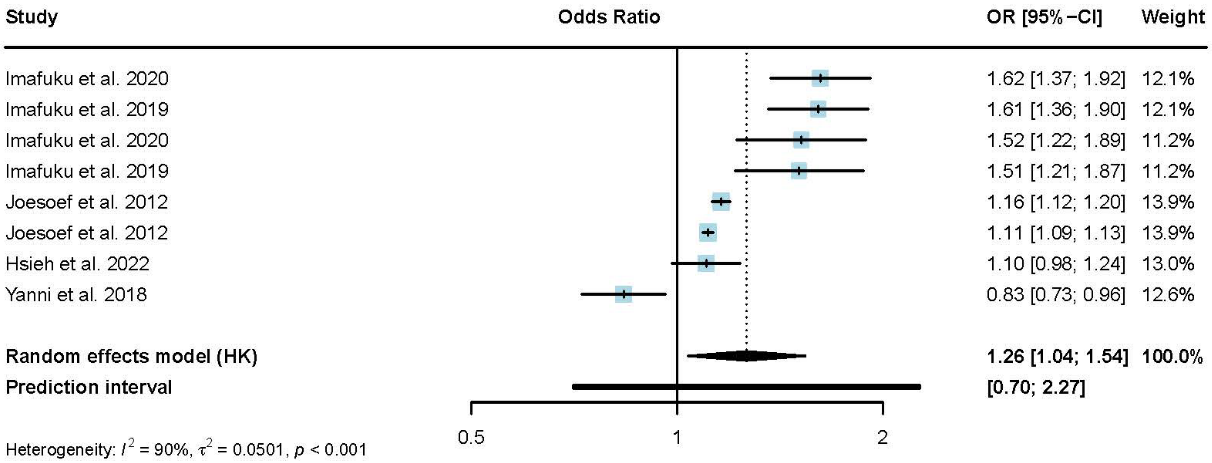

Heterogeneity: $I^2 = 90\%$, $\tau^2 = 0.0501$, $p < 0.001$

Musculoskeletal disorders

Study	OR [95%−CI]	Weight
Imafuku et al. 2020	2.20 [1.85; 2.60]	7.7%
Imafuku et al. 2019	2.18 [1.84; 2.58]	7.7%
Yanni et al. 2018	2.01 [1.91; 2.11]	8.6%
Imafuku et al. 2020	1.54 [1.45; 1.64]	8.6%
Imafuku et al. 2019	1.53 [1.44; 1.62]	8.6%
Hsu et al. 2021	1.36 [1.09; 1.68]	7.2%
Hsu et al. 2021	1.29 [1.12; 1.48]	8.0%
Hsu et al. 2021	1.29 [1.16; 1.42]	8.3%
Joesoef et al. 2012	1.24 [1.20; 1.28]	8.7%
Wang et al. 2020	1.19 [0.95; 1.47]	7.2%
Min et al. 2019	1.17 [1.11; 1.23]	8.6%
Lasserre et al. 2012	1.12 [0.82; 1.52]	6.1%
Lasserre et al. 2012	0.81 [0.49; 1.34]	4.1%
Lasserre et al. 2012	0.67 [0.13; 3.34]	0.7%
**Random effects model (HK)**	**1.43 [1.22; 1.67]**	**100.0%**
**Prediction interval**	**[0.83; 2.47]**	

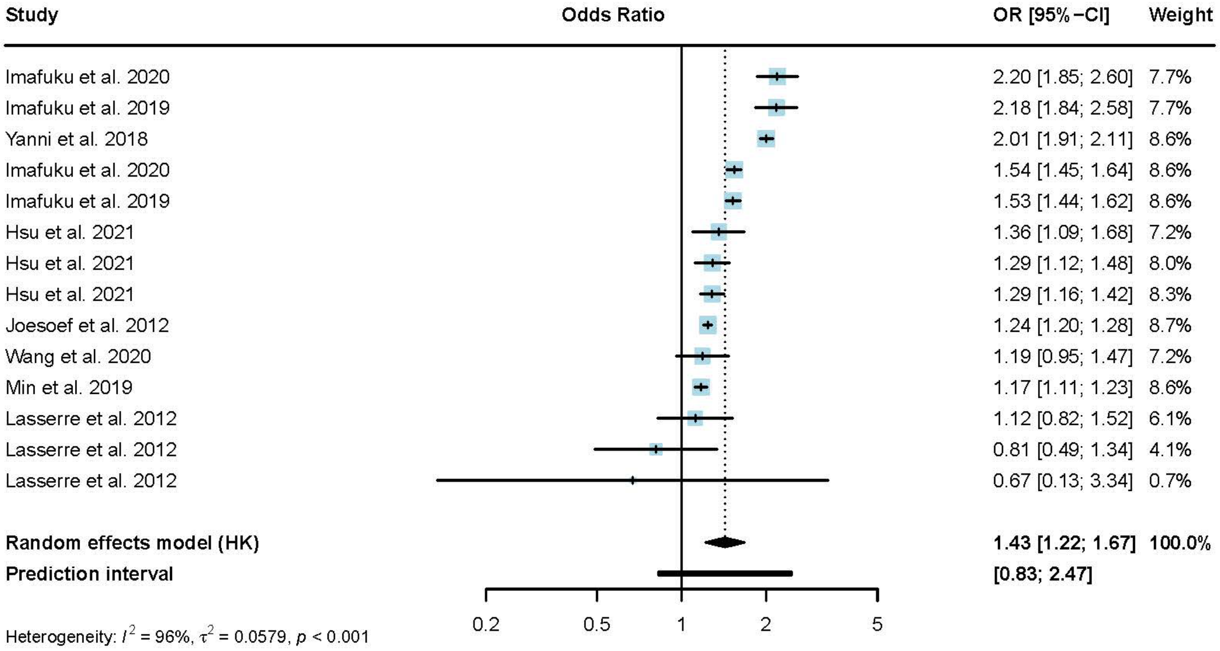

Heterogeneity: $I^2 = 96\%$, $\tau^2 = 0.0579$, $p < 0.001$

Neurological disorders

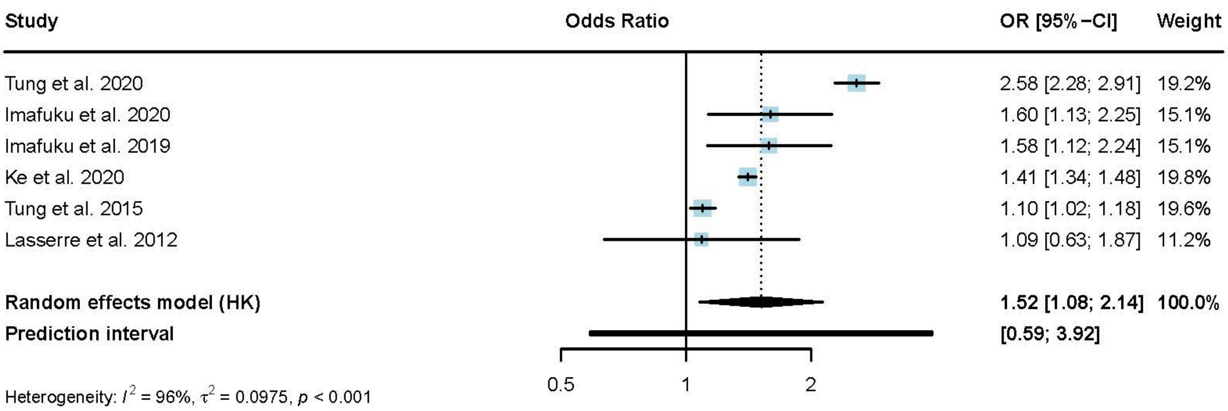

Supplement: Supplementary file 5 — (PDF 3613 kb) [file 15010_2023_2156_MOESM5_ESM.pdf]
