# Supplementary material for: Risk factors for herpes zoster infections: a systematic review and meta-analysis unveiling common trends and heterogeneity patterns
Source: Infection. 2024 Jan 18;52(3):1009–26. doi: 10.1007/s15010-023-02156-y (PMC11142967; doi:10.1007/s15010-023-02156-y)
Supplement: Supplementary file 6 — (PDF 338 kb) [file 15010_2023_2156_MOESM6_ESM.pdf]

S6: Subgroup analyses.

Note: n_k: number of studies; * indicates significant values (p < 0.005), p_h ‘ defines the p-value for heterogeneity between subgroups, p_h ‘’ defines the p-value for heterogeneity within subgroups.

Please note, that we only did subgroup analyses for risk factors, when at least k > 10 studies were available. This was done in order to provide enough statistical power and robust results (see Schwarzer, Guido, James R Carpenter, and Gerta Rücker. 2015. Meta-Analysis with r. Springer. Chapter 4.3).
.

	Effect size			Heterogeneity		
**Subgroups**	**n_k**	**OR**	**95% CI**	**I^2**	**p_h ‘**	**p_h ‘’**
**Asthma**						
All studies	11	1.30	[1.19-1.42]*	89.22%		
Study design					0.5954	< 0.0001
Cohort studies	6	1.28	[1.17- 1.41]	89.0%		
Case-control studies	5	1.36	[0.99-1.87]	69.2%		
Study year					0.2550	< 0.0001
2003-2008	-	-	-	-		
2011-2016	6	1.38	[1.13-1.68]	93.7%		
2017-2022	5	1.25	[1.13- 1.38]	64.5%		
Region					< 0.0001	< 0.0001
Europe	4	1.26	[1.09-1.46]	63.1%		
Northern America	3	1.78	[1.33-2.40]	0.00%		
Asia	4	1.27	[1.06-1.52]	96.0%		
Middle East	-	-	-	-		
Sample size					0.1130	< 0.0001
< 100.000	5	1.49	[1.16-1.90]	43.4%		
100.000- 999.999	2	1.23	[0.95-1.60]	0.0%		
≥ 1.000.000	4	1.26	[1.07-1.48]	92.8%		
**Autoimmune Disorders**						
All studies	16	1.33	[0.98-1.80]	98.28%		
Study design					0.6123	< 0.0001
Cohort studies	14	1.32	[0.93-1.87]	98.5%		
Case-control studies	2	1.45	[0.37- 5.74]	0.00%		
Study year					0.8321	< 0.0001
2003-2008	-	-	-	-		
2011-2016	7	1.28	[0.59-2.77]	99.2%		
2017-2022	9	1.37	[1.11-1.69]	76.9%		
Region					0.7088	< 0.0001
Europe	11	1.24	[0.81-1.90]	98.2%		
Northern America	1	1.73	[0.55-5.46]	-		
Asia	4	1.52	[0.80-2.89]	98.3%		
Middle East	-	-	-	-		
Sample size					0.4338	< 0.0001
< 100.000	4	0.90	[0.16- 4.92]	99.4%		
100.000- 999.999	2	1.25	[0.07-21.78]	90.5%		
≥ 1.000.000	10	1.55	[1.25-1.91]	95.4%		
**Cancer**						
All studies	20	2.42	[1.91-3.07]*	99.07%		
Study design					0.0481	0
Cohort studies	17	2.62	[2.03-3.38]	98.9%		

Case-control studies	3	1.56	[0.57-4.24]	99.4%		
Study year					0.4783	< 0.0001
2003-2008	-	-	-	-		
2011-2016	12	2.60	[1.90-3.56]	96.6%		
2017-2022	8	2.20	[1.39-3.47]	98.8%		
Region					0.0036	< 0.0001
Europe	12	2.98	[2.10-4.22]	96.8%		
Northern America	2	2.37	[1.13-4.99]	18.4%		
Asia	6	1.66	[1.25-2.22]	98.9%		
Middle East	-	-	-	-		
Sample size					0.0465	< 0.0001
< 100.000	12	2.48	[1.75-3.50]	98.0%		
100.000- 999.999	2	1.68	[0.43-6.51]	97.9%		
≥ 1.000.000	6	2.63	[1.51-4.57]	98.7%		
**Cardiovascular Disorders**						
All studies	11	1.39	[1.12-1.73]*	99.33%		
Study design					0.0002	< 0.0001
Cohort studies	6	1.69	[1.27-2.25]	56.9%		
Case-control studies	5	1.06	[0.90-1.25]	99.6%		
Study year					0.3094	< 0.0001
2003-2008	-	-	-	-		
2011-2016	6	1.53	[1.02-2.28]	99.1%		
2017-2022	5	1.26	[0.93-1.71]	94.9%		
Region					0.8660	< 0.0001
Europe	5	1.43	[1.01-2.01]	94.5%		
Northern America	1	1.17	[0.59-2.32]	-		
Asia	5	1.40	[0.82-2.39]	99.7%		
Middle East	-	-	-	-		
Sample size					0.1909	< 0.0001
< 100.000	5	1.18	[0.79-1.77]	92.7%		
100.000- 999.999	1	1.17	[0.65-2.11]	-		
≥ 1.000.000	5	1.65	[1.15-2.38]	99.6%		
**COPD**						
All studies	10	1.55	[1.04-2.31]*	99.91%		
Study design					0.1367	0
Cohort studies	7	1.77	[1.04-3.00]	99.9%		
Case-control studies	3	1.11	[0.41-2.98]	52.2%		
Study year					0.5915	0
2003-2008	-	-	-	-		
2011-2016	5	1.40	[0.82-2.39]	99.1%		
2017-2022	5	1.70	[0.72-4.01]	100.0%		
Region					0.4340	0
Europe	3	1.50	[1.12-2.02]	59.9%		
Northern America	2	2.78	[0.00-25982.13]	99.9%		
Asia	5	1.24	[0.74-2.09]	99.5%		
Middle East	-	-	-	-		
Sample size					0.5055	0
< 100.000	2	1.13	[0.00-609.23]	85.8%		

100.000- 999.999	2	1.33	[1.16-1.54]	0.0%		
≥ 1.000.000	6	1.78	[0.92-3.42]	99.9%		
**Diabetes**						
All studies	17	1.26	[1.03-1.54]*	99.70%		
Study design					0.8398	0
Cohort studies	12	1.25	[0.94-1.66]	99.6%		
Case-control studies	5	1.29	[0.94-1.77]	98.4%		
Study year					0.4794	0
2003-2008	1	1.53	[0.70-3.36]	-		
2011-2016	9	1.34	[0.90-2.00]	99.8%		
2017-2022	7	1.12	[0.98-1.29]	89.3%		
Region					0.7025	0
Europe	7	1.43	[1.10-1.86]	94.4%		
Northern America	2	1.36	[0.06-33.12]	99.9%		
Asia	6	1.23	[0.87- 1.74]	99.7%		
Middle East	2	0.82	[0.00-2287.12]	99.8%		
Sample size					0.3105	0
< 100.000	5	1.56	[0.96-2.53]	90.7%		
100.000- 999.999	5	1.18	[0.96-1.44]	98.4%		
≥ 1.000.000	7	1.16	[0.73-1.84]	99.8%		
**Digestive Disorders**						
All studies	11	1.26	[0.98-1.61]	97.18%		
Study design					0.3841	< 0.0001
Cohort studies	10	1.23	[0.95-1.60]	97.5%		
Case-control studies	1	2.09	[0.65-6.69]	-		
Study year					0.0022	< 0.0001
2003-2008	-	-	-	-		
2011-2016	2	1.83	[0.69-4.87]	0.0%		
2017-2022	9	1.19	[0.90-1.56]	97.2%		
Region					< 0.0001	< 0.0001
Europe	6	1.33	[0.91-1.95]	98.2%		
Northern America	2	0.80	[0.50-1.30]	0.0%		
Asia	3	1.59	[1.14-2.22]	0.0%		
Middle East	-	-	-	-		
Sample size					0.3545	< 0.0001
< 100.000	8	1.20	[0.84- 1.73]	97.9%		
100.000- 999.999	3	1.40	[1.10-1.78]	85.9%		
≥ 1.000.000	-	-	-	-		
**HIV**						
All studies	12	1.81	[1.21-2.69]*	93.67%		
Study design					0.5455	< 0.0001
Cohort studies	9	1.66	[1.09-2.53]	94.5%		
Case-control studies	3	2.34	[0.24-23.03]	92.1%		
Study year					0.0355	< 0.0001
2003-2008	3	1.14	[0.57- 2.26]	0.0%		
2011-2016	7	2.48	[1.32-4.66]	89.4%		
2017-2022	2	1.46	[0.04-59.82]	73.7%		
Region					0.3870	< 0.0001

Europe	1	2.03	[0.56-7.41]	-		
Northern America	4	1.88	[0.72-4.91]	81.3%		
Asia	4	2.28	[0.72-7.23]	96.4%		
Middle East	3	1.06	[0.27-4.14]	10.1%		
Sample size					0.8924	< 0.0001
< 100.000	6	1.60	[1.01-2.53]	0.0%		
100.000- 999.999	3	1.91	[0.22-16.44]	97.5%		
≥ 1.000.000	3	1.92	[0.27-13.46]	95.1%		
**Mental Health Condition**						
All studies	15	1.43	[0.98-2.11]	99.65%		
Study design					0.7481	0
Cohort studies	7	1.53	[0.61-3.85]	99.8%		
Case-control studies	8	1.35	[1.02-1.79]	91.0%		
Study year					0.1409	0
2003-2008	-	-	-	-		
2011-2016	4	2.65	[0.43-16.22]	99.9%		
2017-2022	11	1.14	[0.96-1.36]	82.1%		
Region					0.6920	0
Europe	5	1.66	[0.37-7.43]	99.8%		
Northern America	2	1.73	[0.00-693.76]	97.6%		
Asia	8	1.25	[1.08-1.44]	86.0%		
Middle East	-	-	-	-		
Sample size					0.4062	0
< 100.000	6	1.21	[0.72-2.03]	89.9%		
100.000- 999.999	9	1.60	[0.86-2.98]	99.8%		
≥ 1.000.000	-	-	-	-		
**Musculoskeletal Disorders**						
All studies	14	1.43	[1.22-1.67]*	96.45%		
Study design					0.0006	< 0.0001
Cohort studies	6	1.74	[1.36-2.21]	94.3%		
Case-control studies	8	1.21	[1.09-1.35]	24.4%		
Study year					0.0078	< 0.0001
2003-2008	-	-	-	-		
2011-2016	4	1.08	[0.78-1.51]	18.6%		
2017-2022	10	1.53	[1.29-1.82]	96.6%		
Region					0.7310	< 0.0001
Europe	9	1.48	[1.23-1.78]	93.5%		
Northern America	1	1.24	[0.73-2.09]	-		
Asia	4	1.31	[0.63-2.69]	88.9%		
Middle East	-	-	-	-		
Sample size					0.0025	< 0.0001
< 100.000	7	1.20	[1.03-1.39]	0.0%		
100.000- 999.999	3	1.43	[0.68-2.98]	99.3%		
≥ 1.000.000	4	1.81	[1.30-2.50]	89.9%		
**Renal Disorders**						
All studies	10	1.17	[0.93-1.48]	94.82%		
Study design					0.6750	< 0.0001
Cohort studies	7	1.20	[0.84-1.73]	95.7%		

Case-control studies	3	1.12	[0.79-1.60]	90.3%		
Study year					0.1222	< 0.0001
2003-2008	-	-	-	-		
2011-2016	5	1.33	[0.91-1.94]	93.8%		
2017-2022	5	0.99	[0.69-1.43]	92.9%		
Region					0.4398	< 0.0001
Europe	8	1.20	[0.89-1.64]	95.1%		
Northern America	-	-	-	-		
Asia	2	1.08	[0.55-2.13]	90.8%		
Middle East	-	-	-	-		
Sample size					0.7355	< 0.0001
< 100.000	6	1.23	[0.80-1.90]	96.5%		
100.000- 999.999	2	1.08	[0.55-2.13]	90.8%		
≥ 1.000.000	2	1.11	[1.06-1.16]	0.00%		
**Rheumatoid Arthritis**						
All studies	13	1.62	[1.29-2.02]*	99.03%		
Study design					0.1071	< 0.0001
Cohort studies	10	1.70	[1.27-2.29]	99.0%		
Case-control studies	3	1.37	[1.15-1.63]	11.5%		
Study year					0.8548	< 0.0001
2003-2008	4	1.73	[1.04-2.87]	96.3%		
2011-2016	4	1.66	[0.89-3.10]	99.4%		
2017-2022	5	1.50	[0.85-2.64]	98.6%		
Region					0.9895	< 0.0001
Europe	4	1.57	[0.65-3.77]	98.1%		
Northern America	4	1.64	[0.87-3.10]	96.5%		
Asia	5	1.64	[1.18-2.26]	98.6%		
Middle East	-	-	-	-		
Sample size					0.0821	< 0.0001
< 100.000	4	1.38	[0.85-2.23]	82.6%		
100.000- 999.999	4	1.46	[0.63-3.37]	99.5%		
≥ 1.000.000	5	1.99	[1.56-2.54]	98.2%		
**Systemic Lupus Erythematosus**						
All studies	10	2.87	[1.99-4.13]*	97.59%		
Study design					0.2756	< 0.0001
Cohort studies	9	3.07	[2.08-4.52]	97.4%		
Case-control studies	1	1.72	[0.64-4.61]	-		
Study year					0.1464	< 0.0001
2003-2008	-	-	-	-		
2011-2016	4	2.23	[0.96-5.16]	98.5%		
2017-2022	6	3.54	[2.23-5.62]	96.9%		
Region					< 0.0001	< 0.0001
Europe	6	4.31	[3.42-5.43]	9.3%		
Northern America	2	1.94	[0.01-444.11]	99.2%		
Asia	2	1.78	[1.15-2.76]	0.0%		
Middle East	-	-	-	-		
Sample size					0.7397	< 0.0001
< 100.000	4	3.02	[0.92-9.85]	96.9%		

100.000- 999.999	3	2.48	[0.59-10.42]	99.1%		
≥ 1.000.000	3	3.21	[2.61-3.95]	0.0%		
**Transplantation**						
All studies	10	4.51	[1.90-10.70]*	98.43%		
Study design					0.3768	< 0.0001
Cohort studies	9	4.08	[1.60-10.38]	98.6%		
Case-control studies	1	13.46	[1.08-168.42]	-		
Study year					0.4654	< 0.0001
2003-2008	2	2.75	[0.00-3659.43]	78.8%		
2011-2016	4	3.33	[0.32-34.85]	98.5%		
2017-2022	4	7.35	[1.10-49.38]	99.2%		
Region					< 0.0001	< 0.0001
Europe	2	19.84	[18.94-20.77]	0.0%		
Northern America	4	3.65	[1.48-9.04]	98.4%		
Asia	3	3.73	[0.39-35.69]	97.6%		
Middle East	1	0.34	[0.057-1.99]	-		
Sample size					0.7209	< 0.0001
< 100.000	2	2.75	[0.00-3659.12]	78.8%		
100.000- 999.999	3	4.36	[0.37-51.60]	97.6%		
≥ 1.000.000	5	5.50	[0.78-38.93]	99.0%		
