# Supplementary material for: Risk factors for herpes zoster infections: a systematic review and meta-analysis unveiling common trends and heterogeneity patterns
Source: Infection. 2024 Jan 18;52(3):1009–26. doi: 10.1007/s15010-023-02156-y (PMC11142967; doi:10.1007/s15010-023-02156-y)
Supplement: Supplementary file 7 — (PDF 29379 kb) [file 15010_2023_2156_MOESM7_ESM.pdf]

S7. Diagnostic plots for each risk group.

The following diagnostic plots were employed to analyze heterogeneity patterns and identify influential cases in the analysis:

- **Figure 1: Baujat Plots:** Based on the "Leave-One-Out" paradigm, these plots proposed by Baujat et al. (2002) explore meta-analysis heterogeneity. They display the contribution to overall heterogeneity on the x-axis and the influence on the overall effect size on the y-axis. Studies in the upper-right corner of the plot may be particularly influential.
- **Figure 2: Influence Diagnostics:** Viechtbauer and Cheung (2010) present various outlier and influential case diagnostic plots based on the "Leave-One-Out" paradigm. These include standardized residuals, Difference In Fits (DFFITS) values, Cook's distance, covariance ratios, and other statistics. A study may be considered influential based on certain thresholds, such as DFFITS values exceeding $3\sqrt{(p/(k-p))}$ or specific criteria related to hat values and DFBETAS values.
- **Figure 3: Forest Plots for Leave-One-Out Analyses:** Two forest plots display the overall effect and heterogeneity when omitting a specific study. These plots are sorted by I^2 and OR values, respectively.
- **Figure 4: GOSH Plots and GOSH Plots Diagnostics:** These plots, proposed by Olkin, Dahabreh, and Trikalinos (2012), assess effect size heterogeneity. Graphical Display of Study Heterogeneity (GOSH) plots aid in detecting both outliers and distinct homogeneous subgroups within the data. They were generated by fitting a random-effects model to subsets of studies in the analysis. While GOSH plots detect heterogeneity patterns and subgroups, identifying the studies responsible for specific patterns can be challenging. To address this, data points from GOSH plots were randomly sampled, and clustering algorithms were applied to identify contributing studies.

These diagnostic plots help assess heterogeneity and identify studies that may significantly impact the analysis.

Asthma

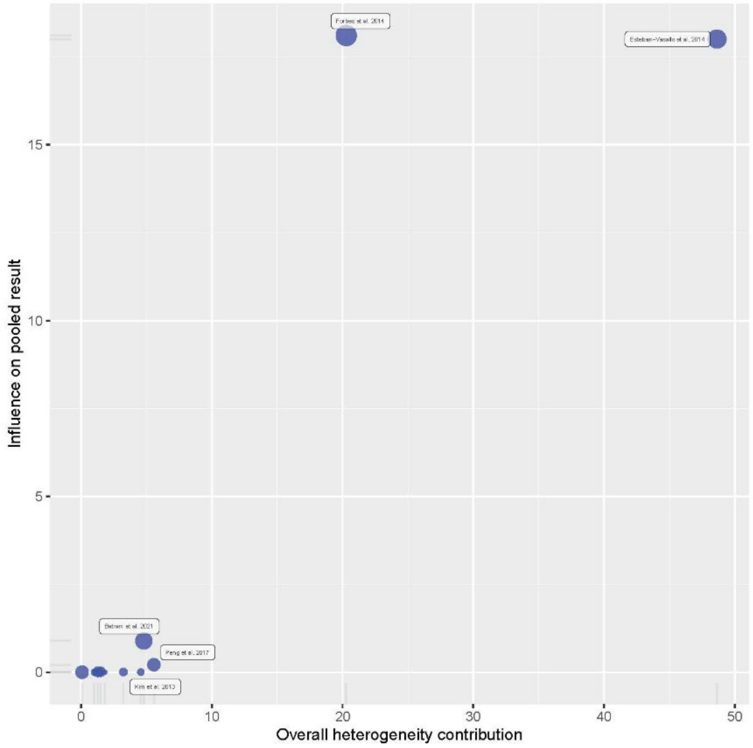

Figure 1: Baujat plot.

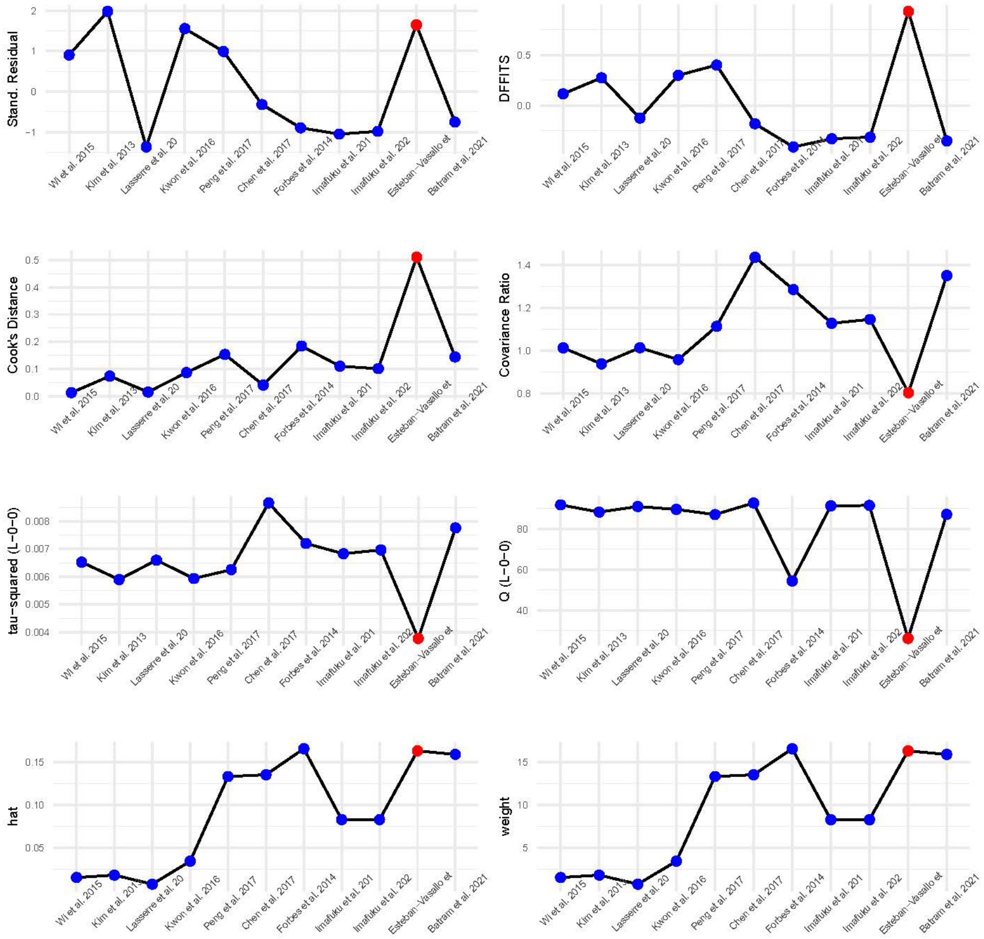

Figure 2: Influence diagnostics.

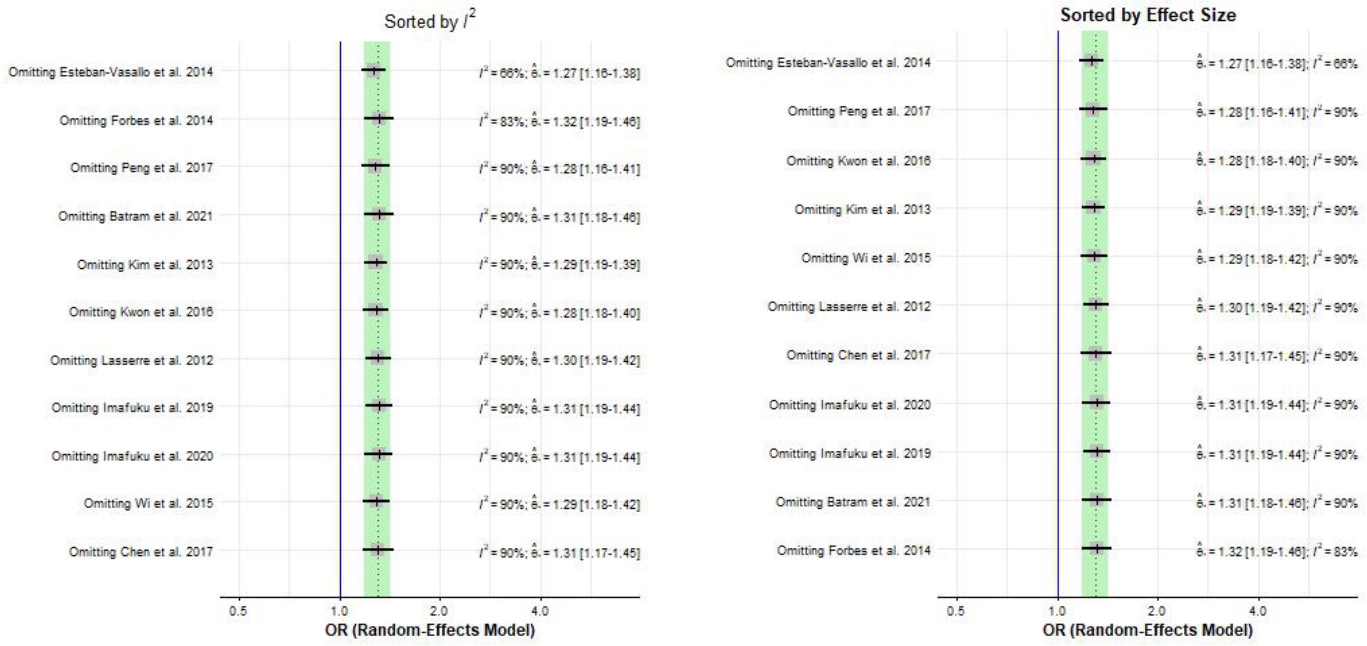

Figure 3: Forest plots for Leave-One-Out Analyses.

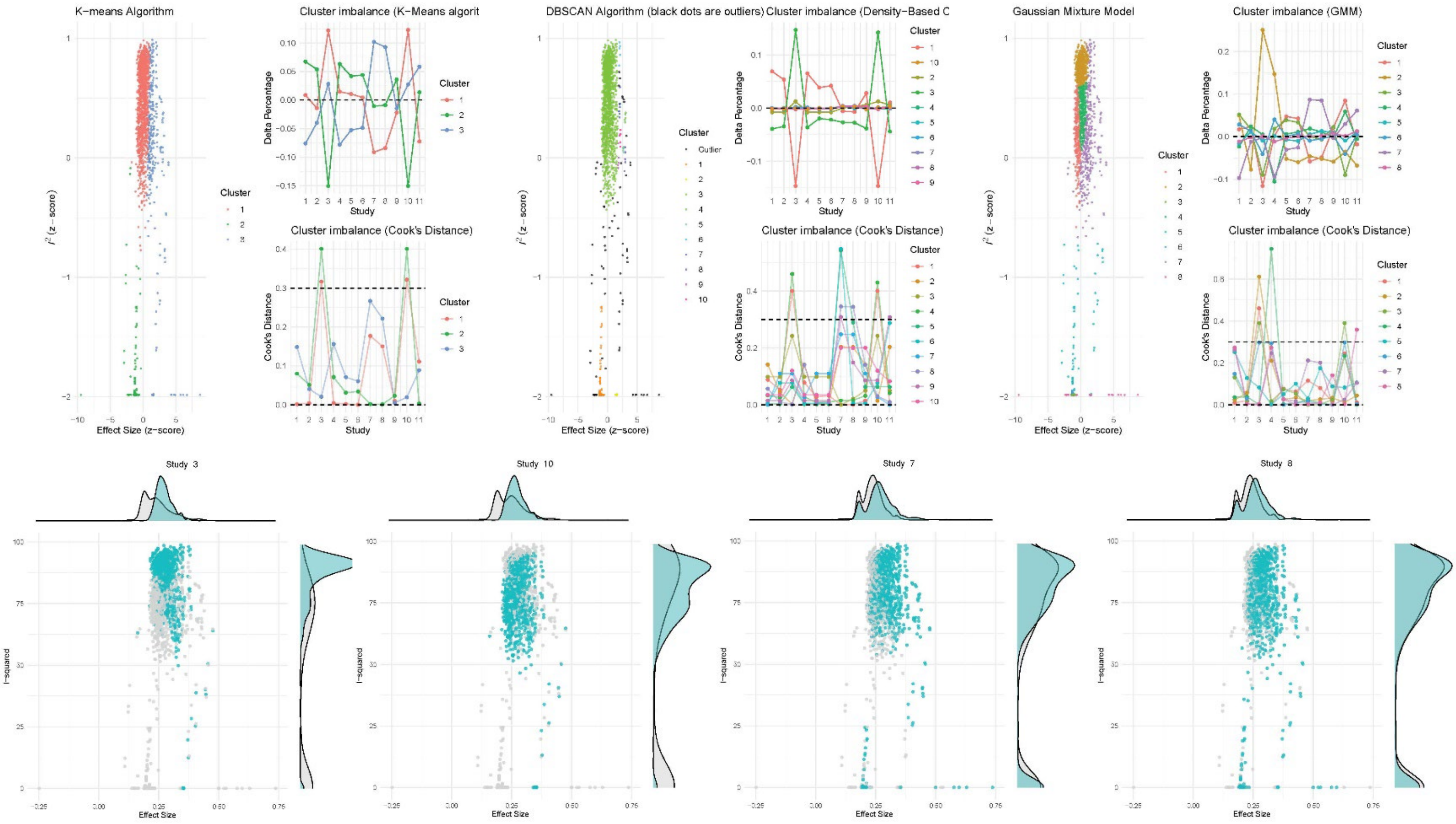

Figure 4: GOSH Plots and GOSH Plot Diagnostics.

Cardiovascular disorders

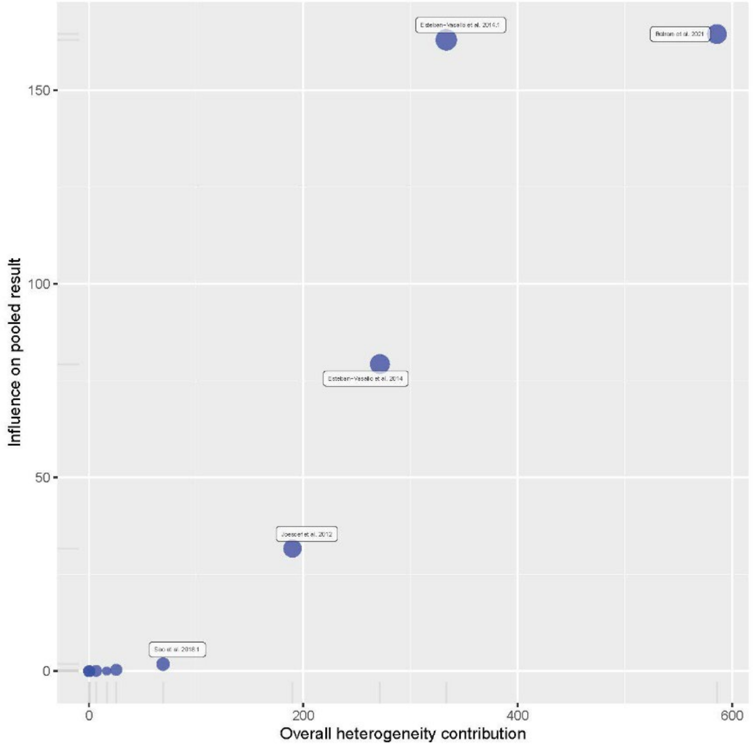

Figure 1: Baujat plot.

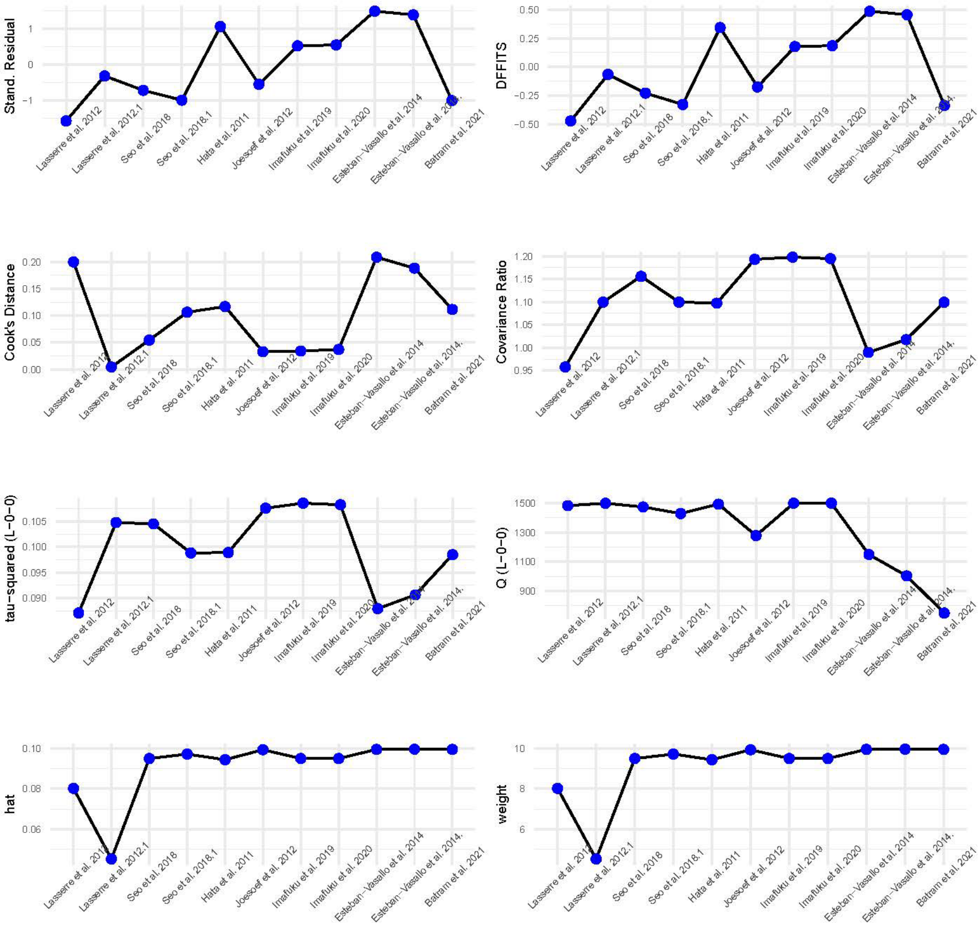

Figure 2: Influence diagnostics.

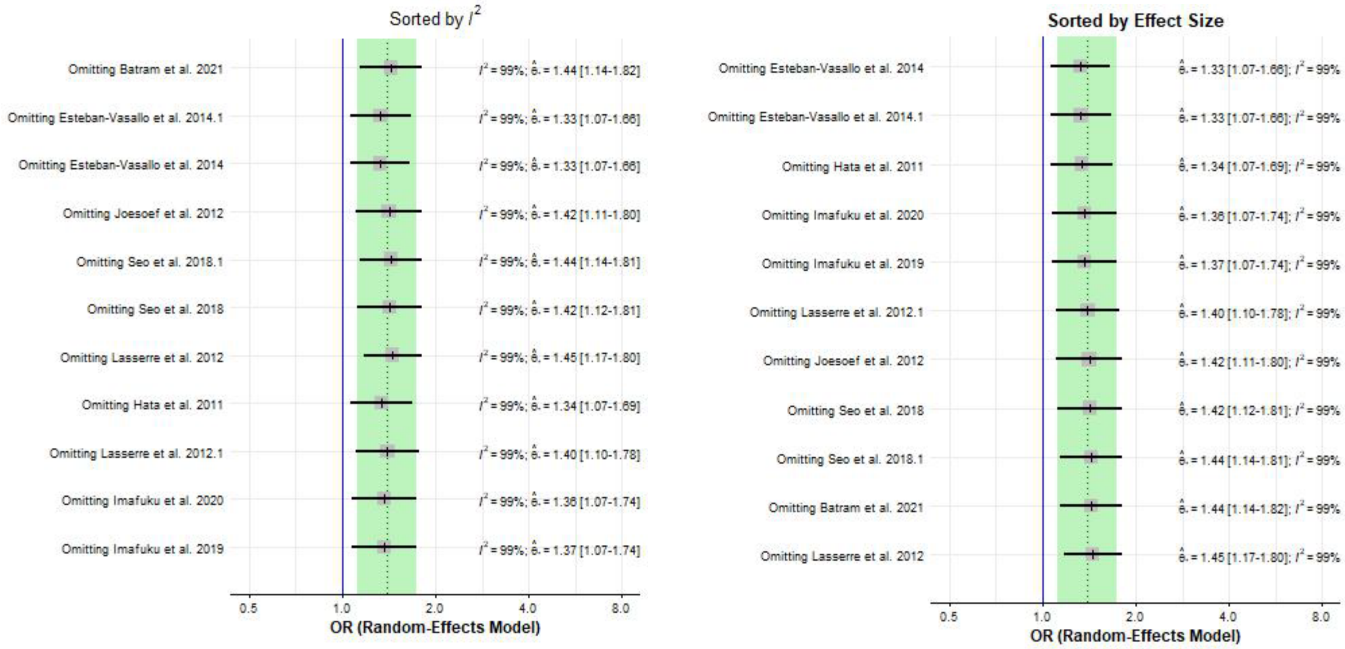

Figure 3: Forest plots for Leave-One-Out Analyses.

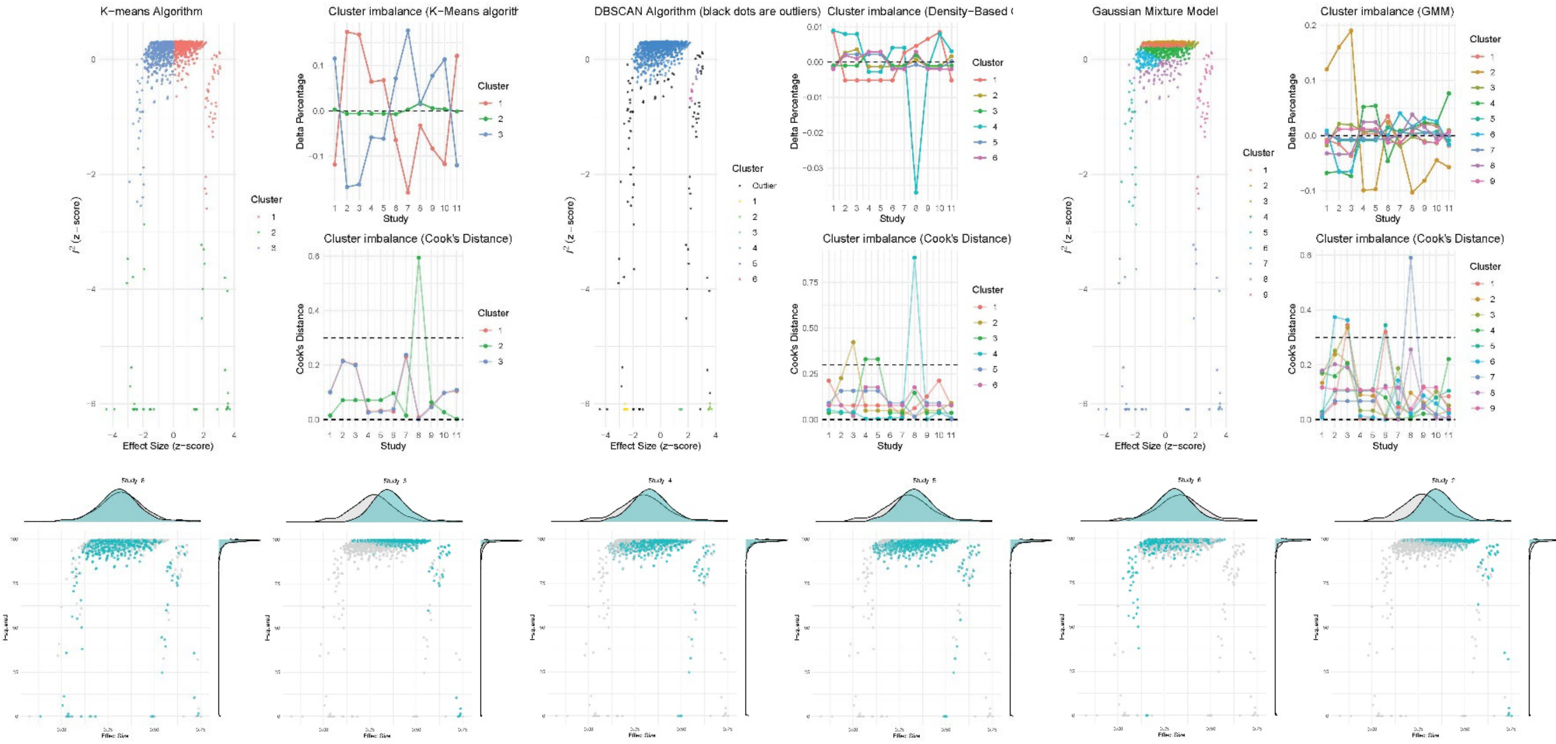

Figure 4: GOSH Plots and GOSH Plot Diagnostics.

CHF

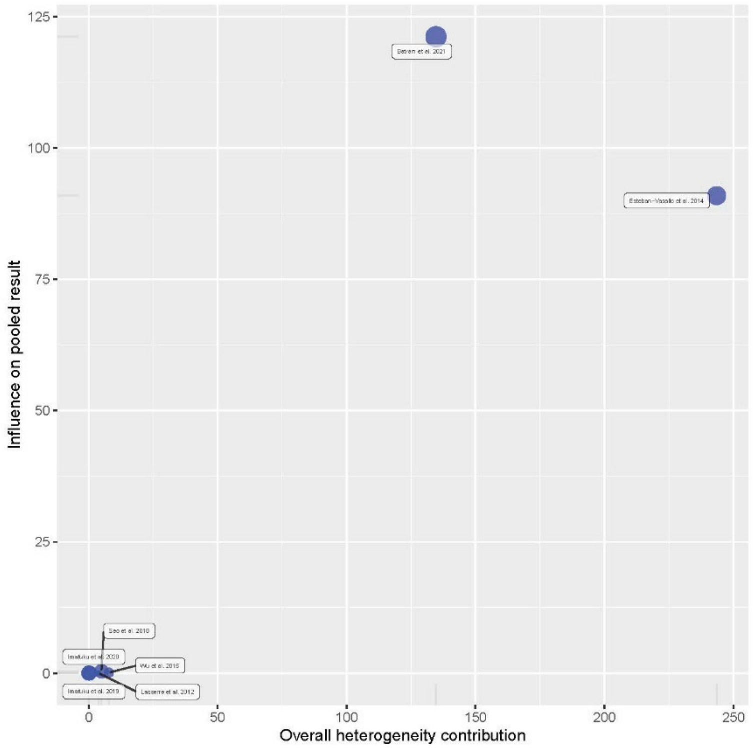

Figure 1: Baujat plot.

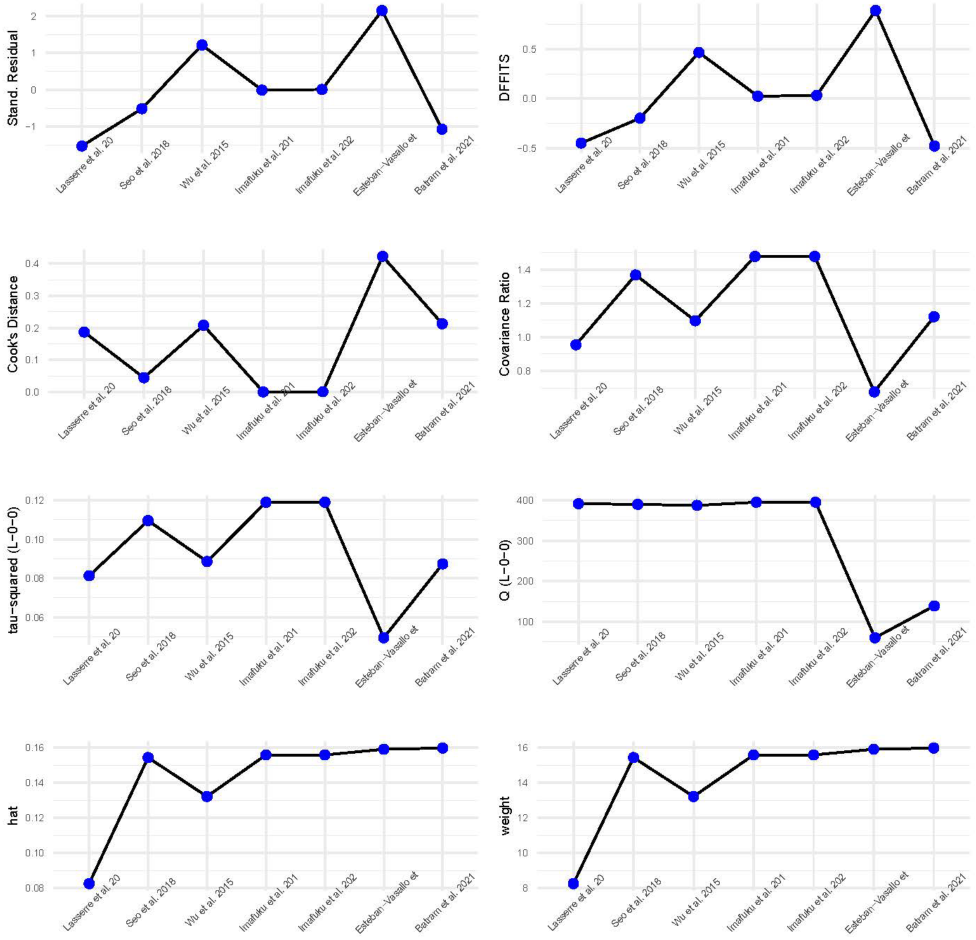

Figure 2: Influence diagnostics.

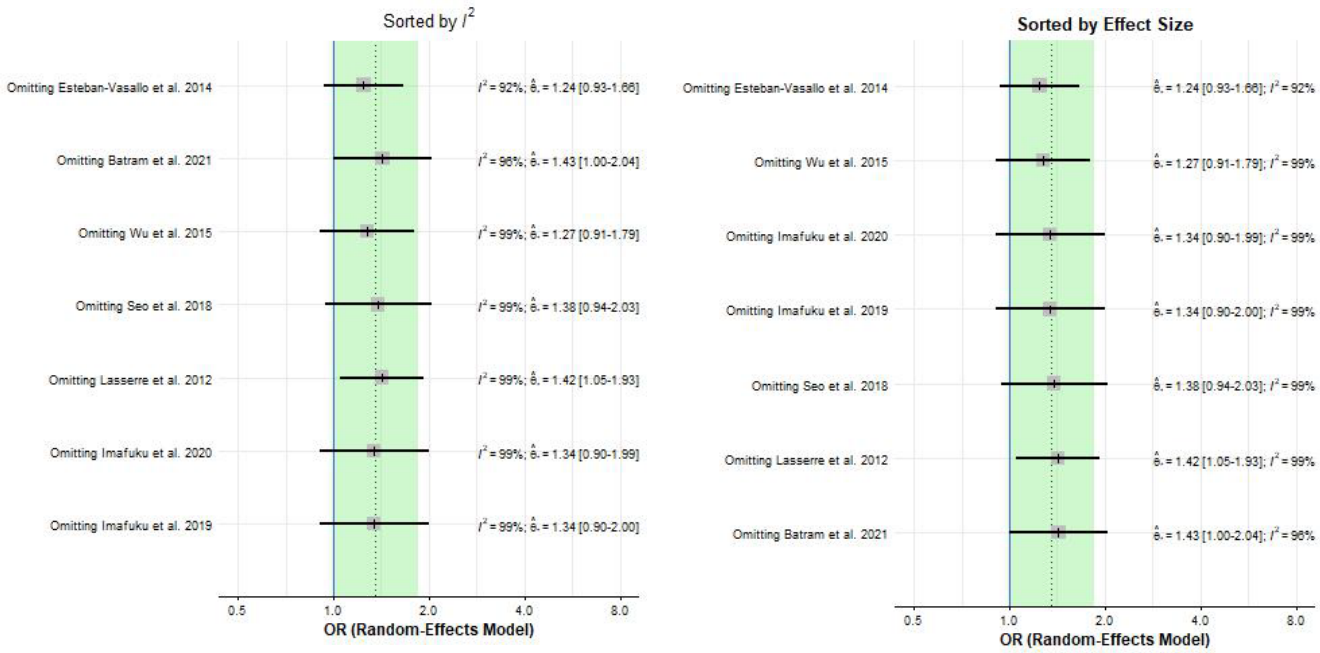

Figure 3: Forest plots for Leave-One-Out Analyses.

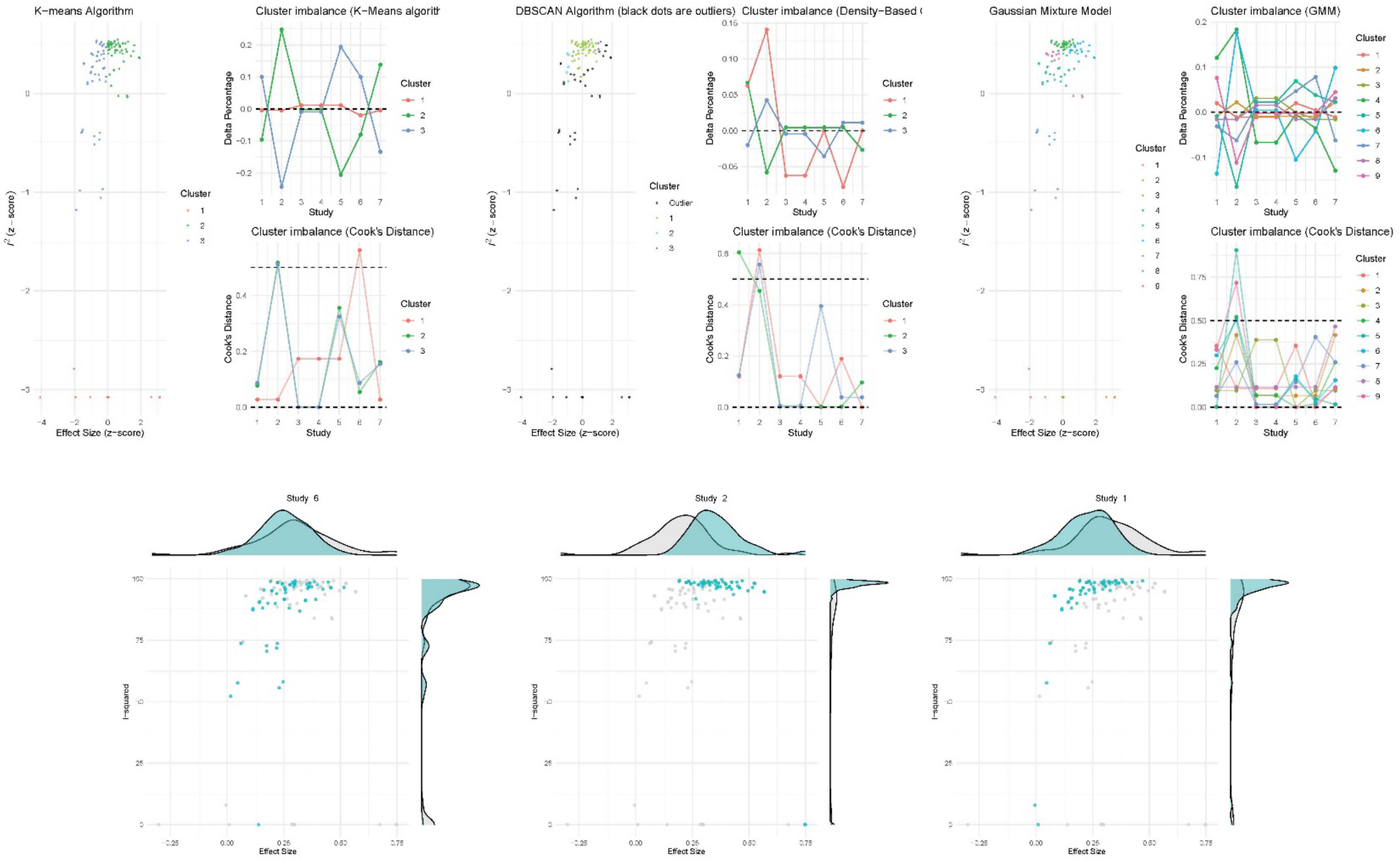

Figure 4: GOSH Plots and GOSH Plot Diagnostics.

COPD

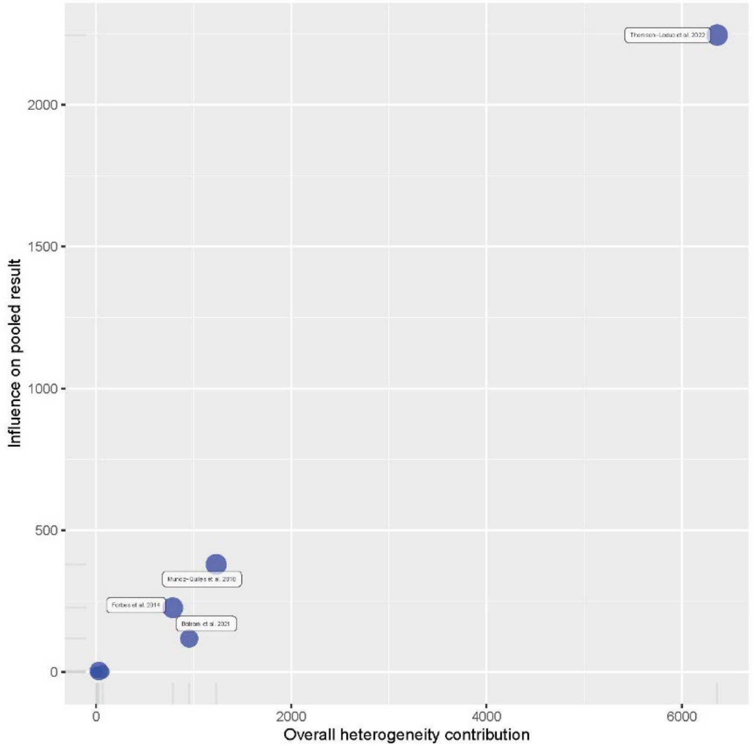

Figure 1: Baujat plot.

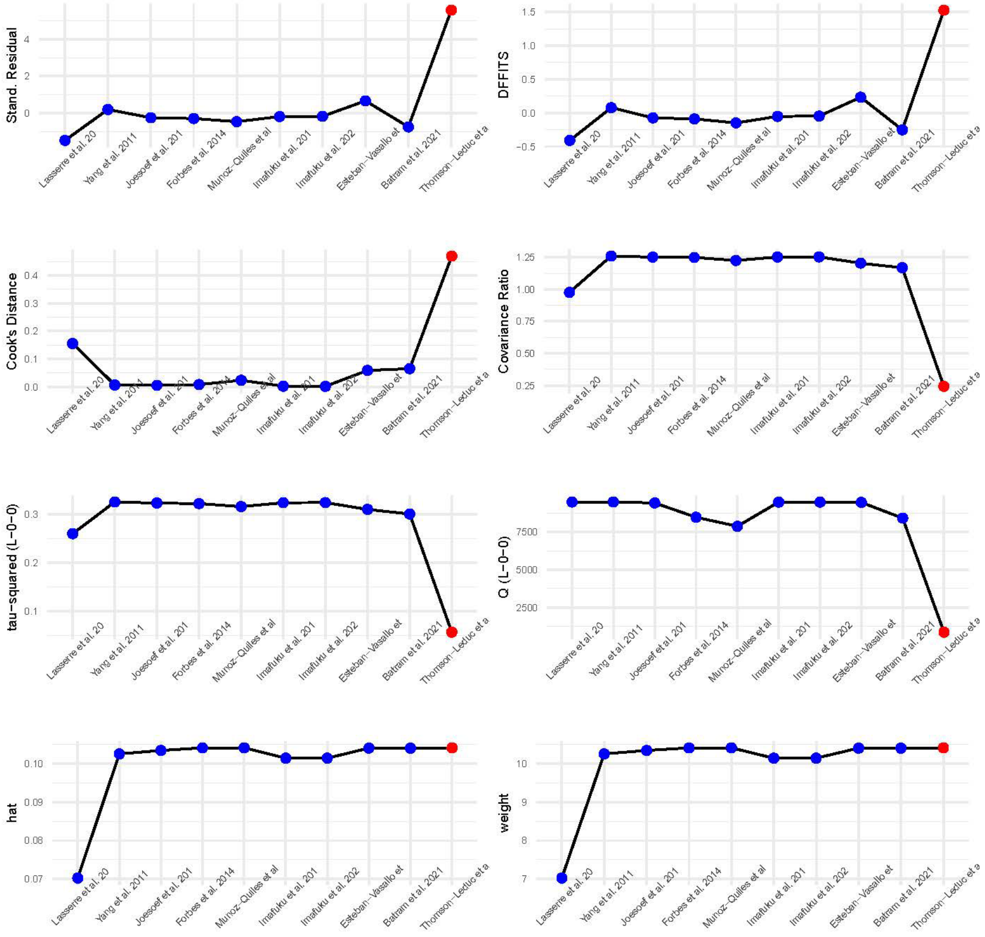

Figure 2: Influence diagnostics.

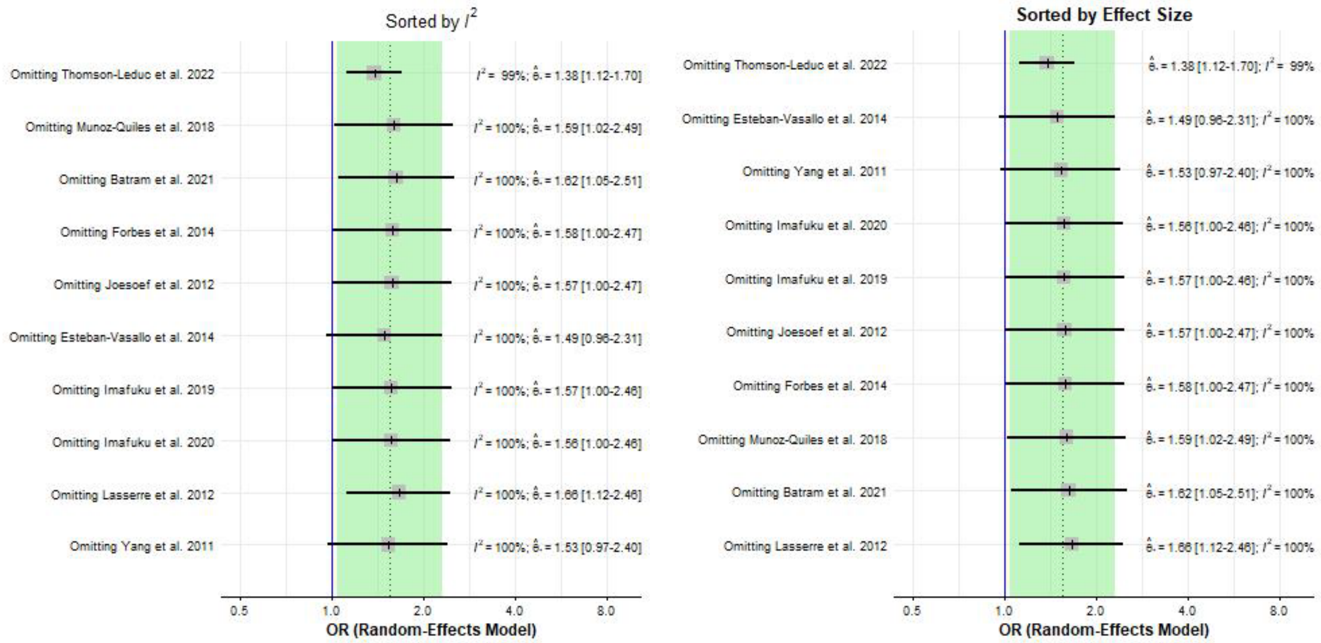

Figure 3: Forest plots for Leave-One-Out Analyses.

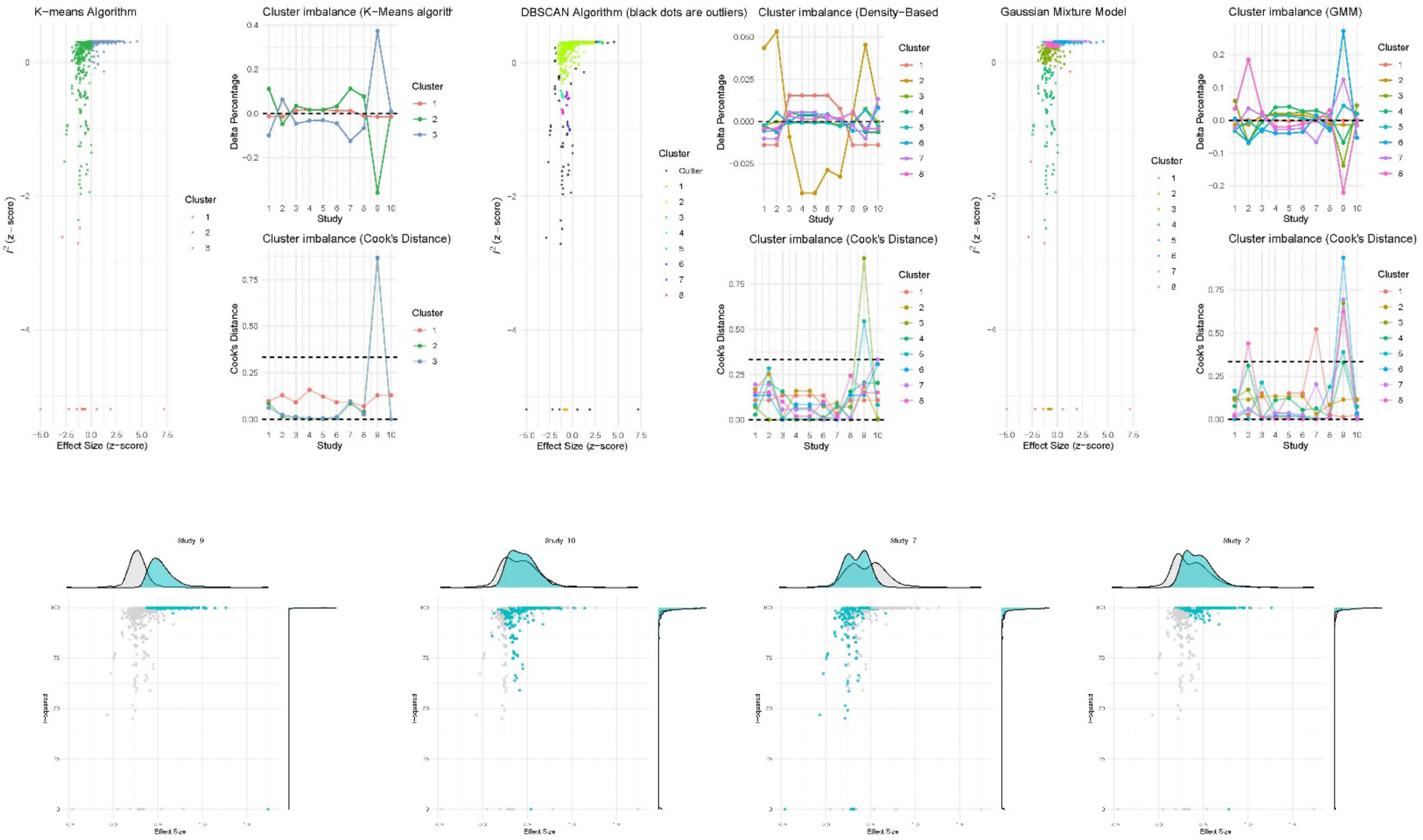

Figure 4: GOSH Plots and GOSH Plot Diagnostics.

Depression

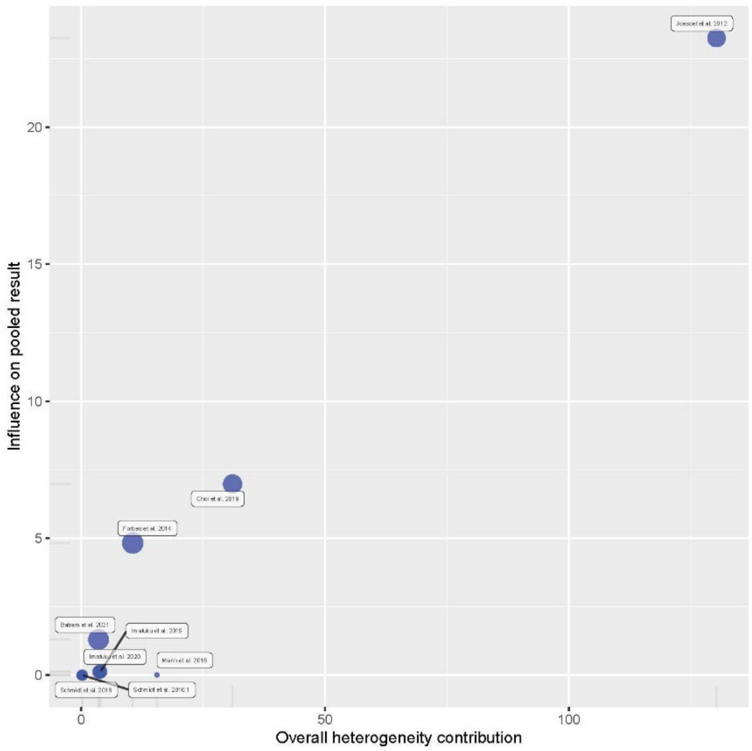

Figure 1: Baujat plot.

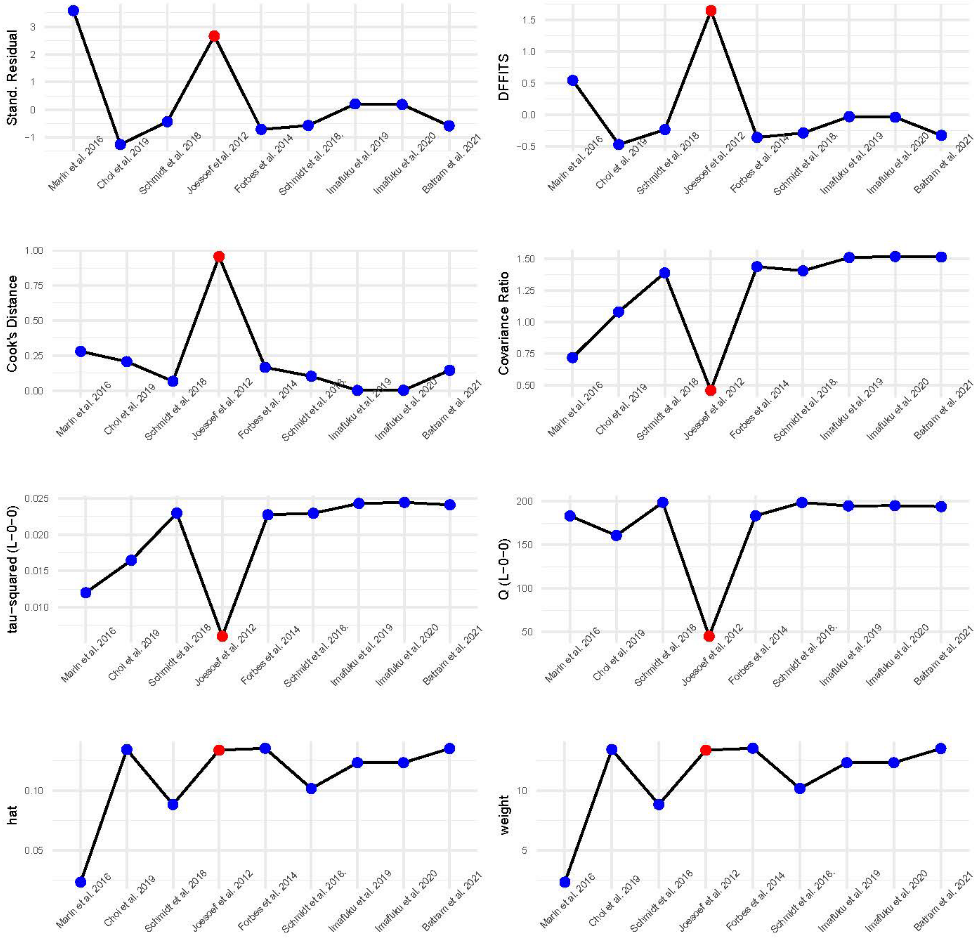

Figure 2: Influence diagnostics.

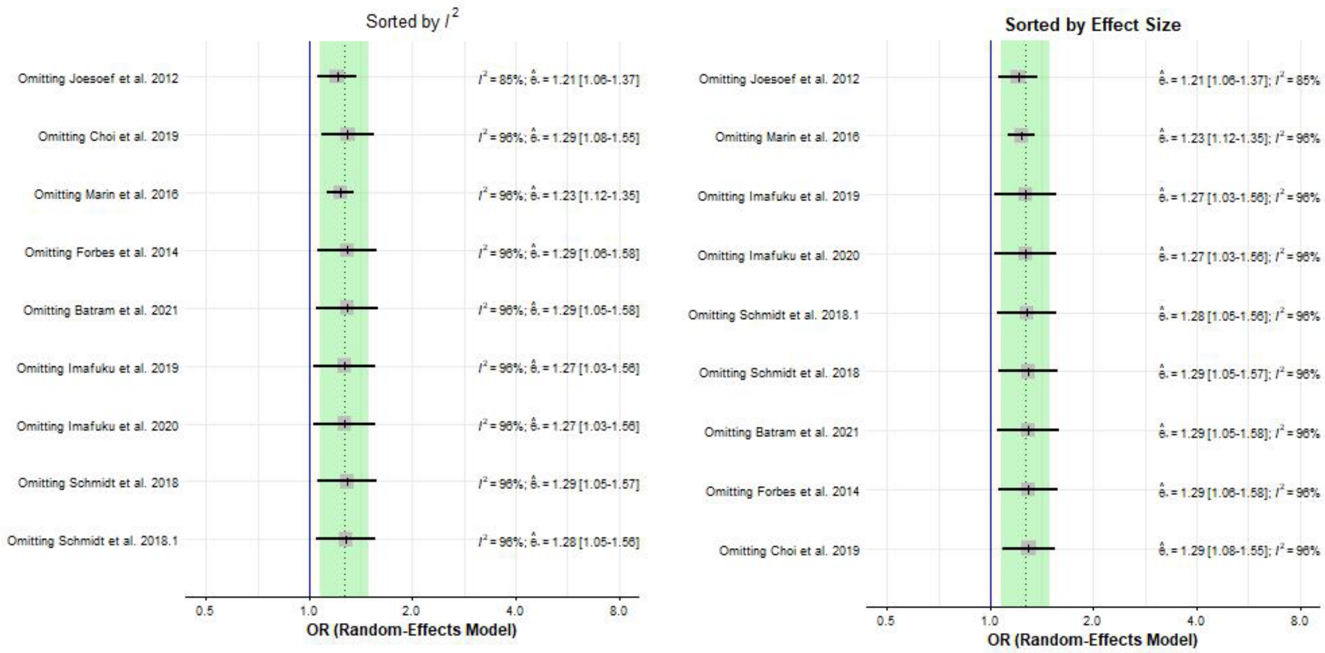

Figure 3: Forest plots for Leave-One-Out Analyses.

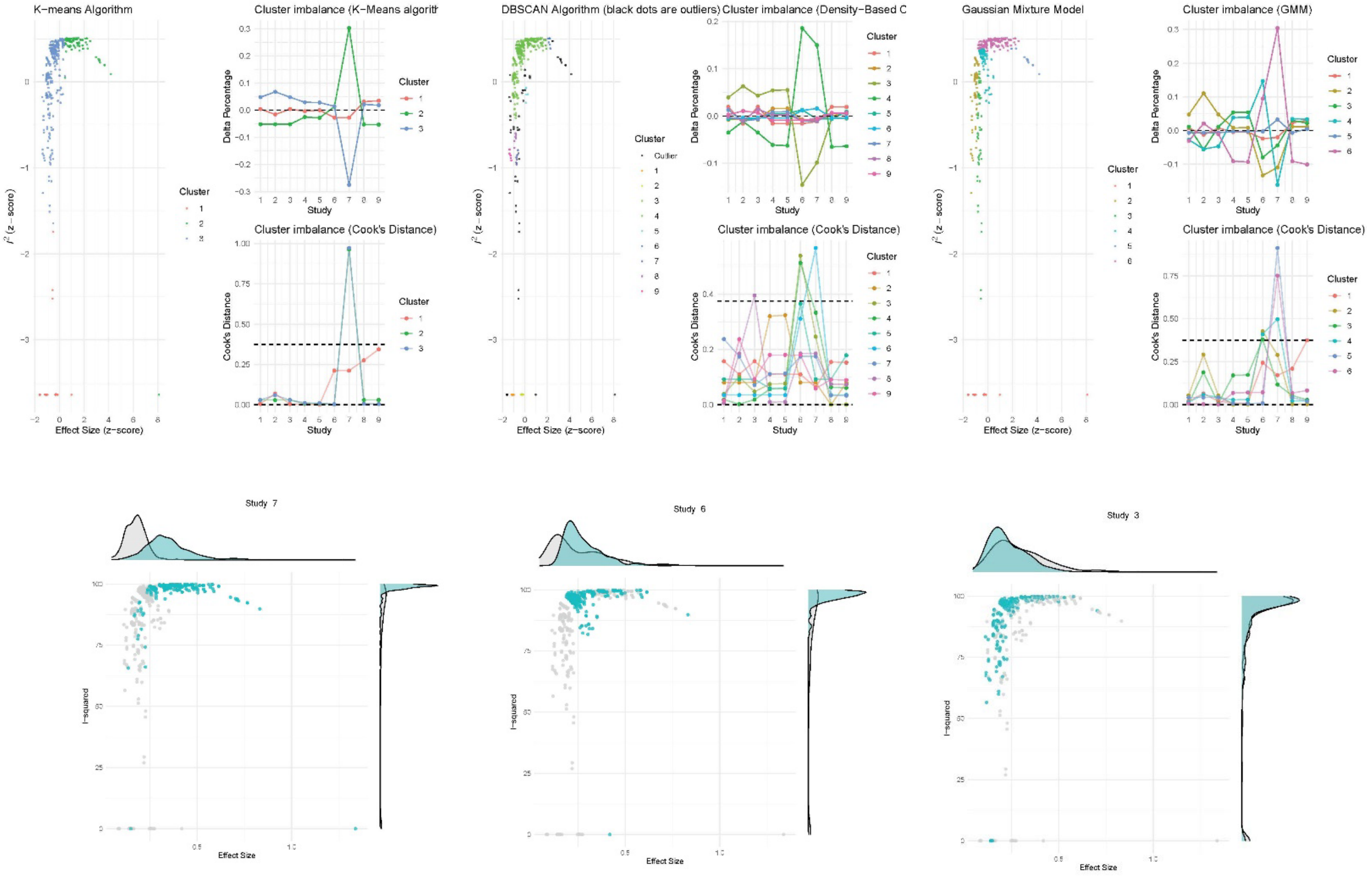

Figure 4: GOSH Plots and GOSH Plot Diagnostics.

Diabetes

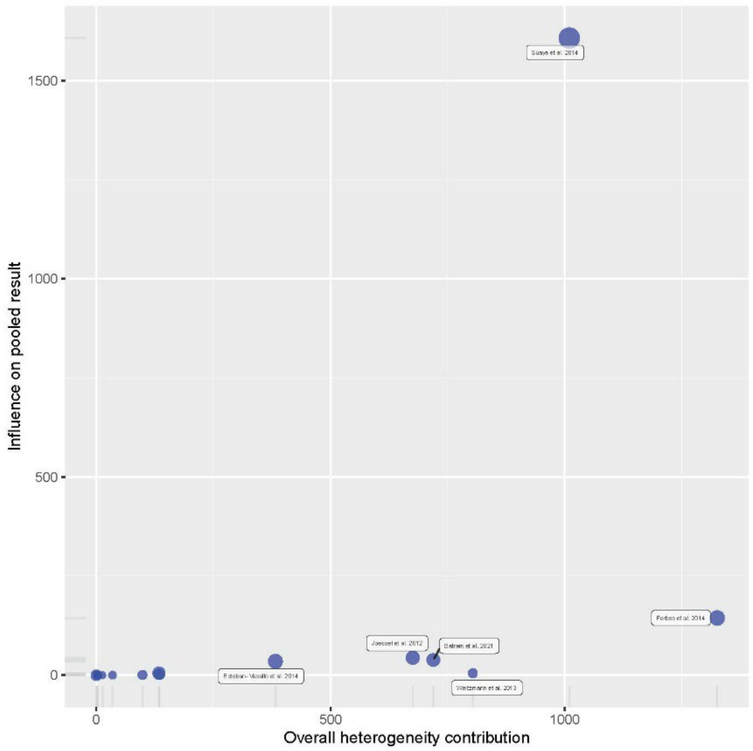

Figure 1: Baujat plot.

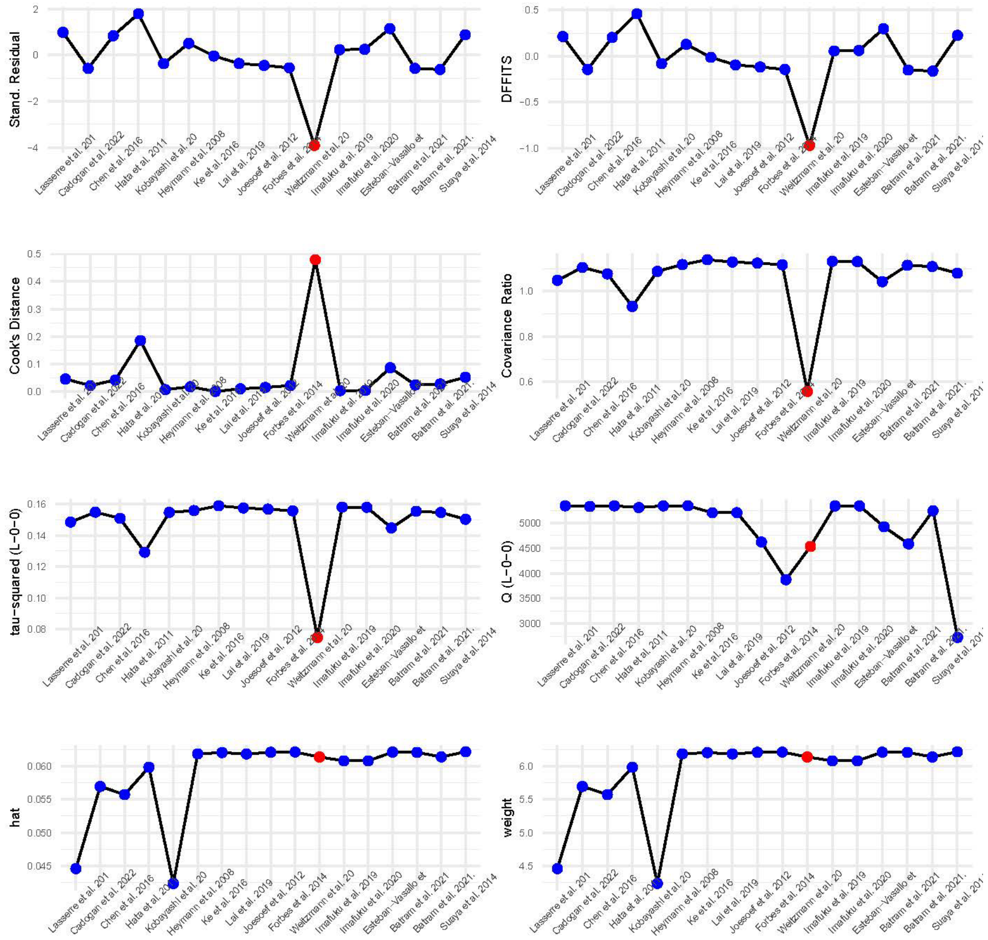

Figure 2: Influence diagnostics.

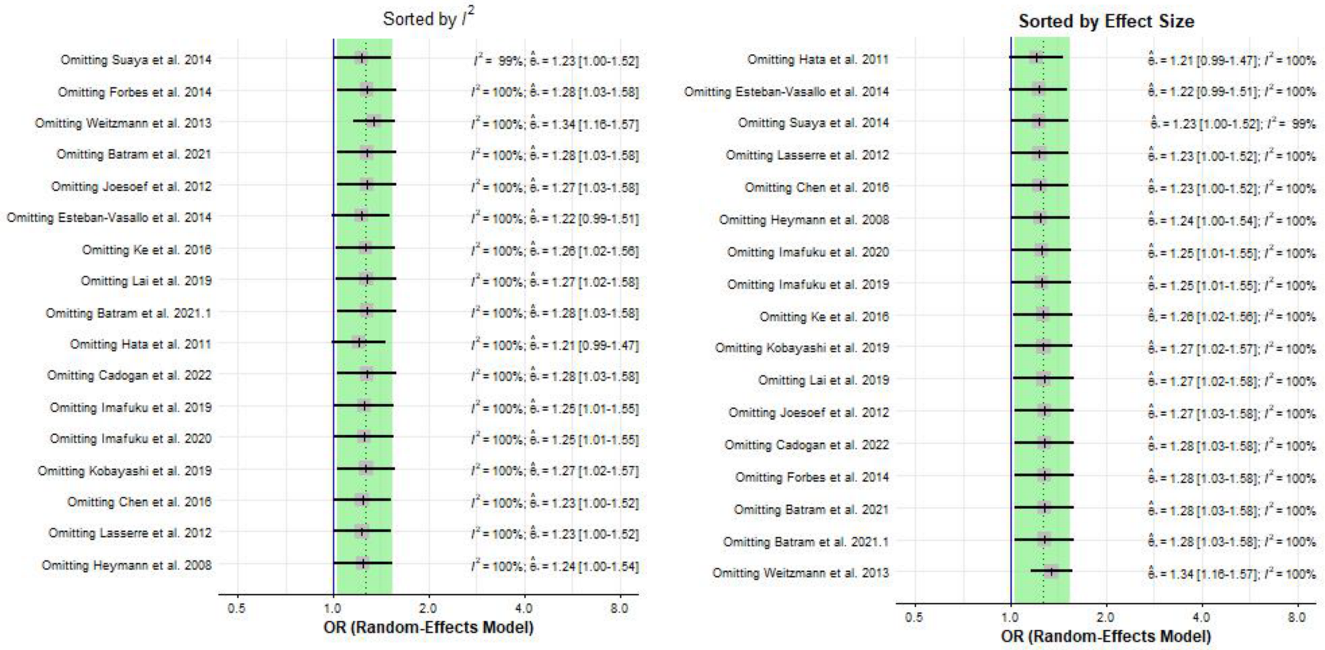

Figure 3: Forest plots for Leave-One-Out Analyses.

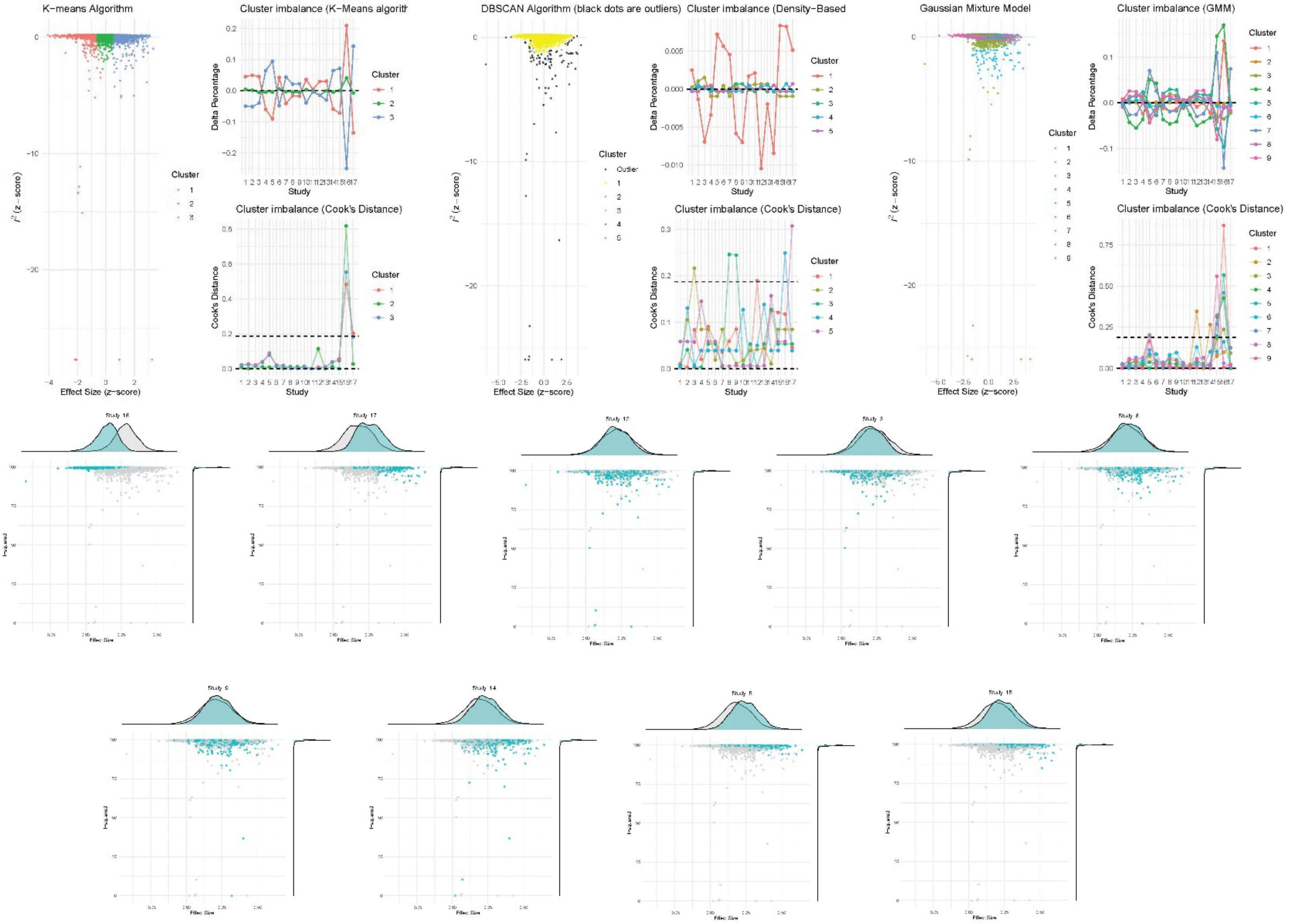

Figure 4: GOSH Plots and GOSH Plot Diagnostics.

RA

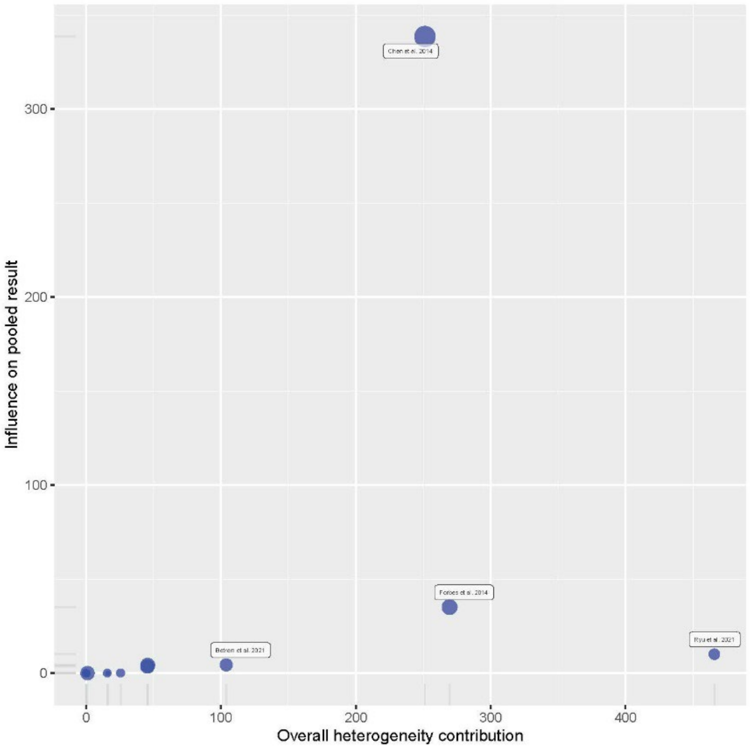

Figure 1: Baujat plot.

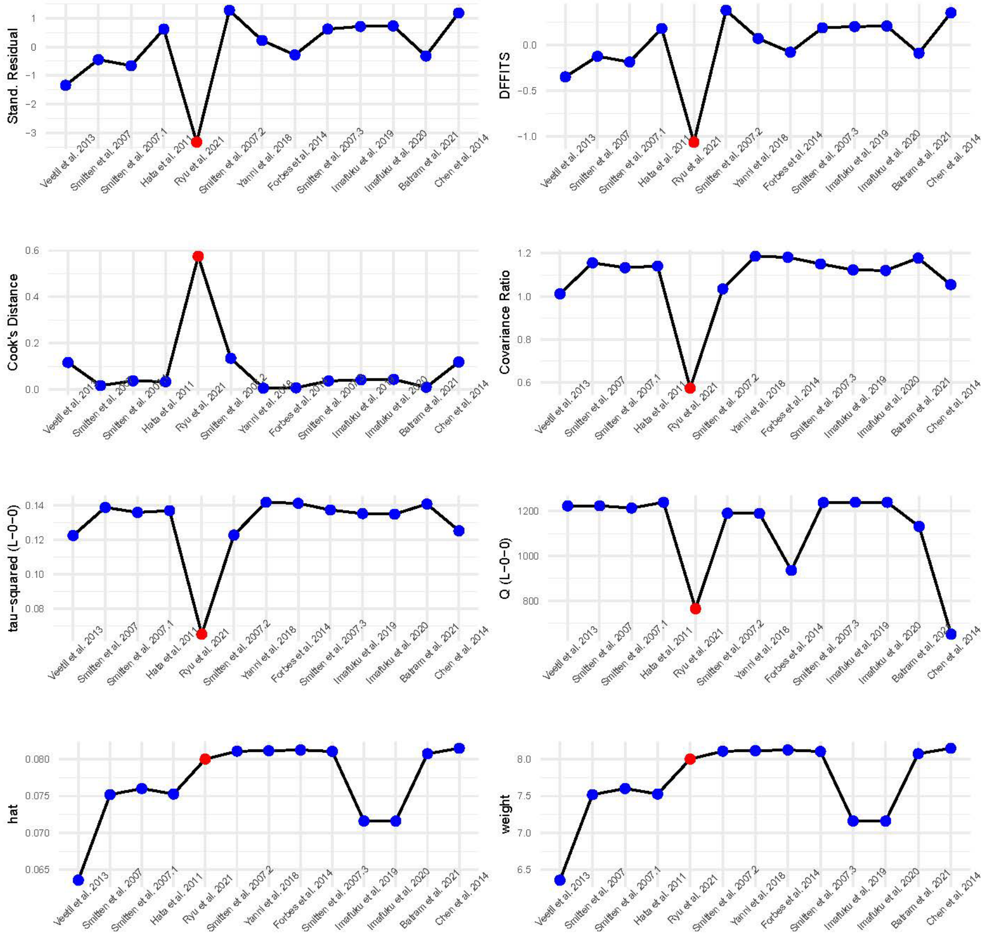

Figure 2: Influence diagnostics.

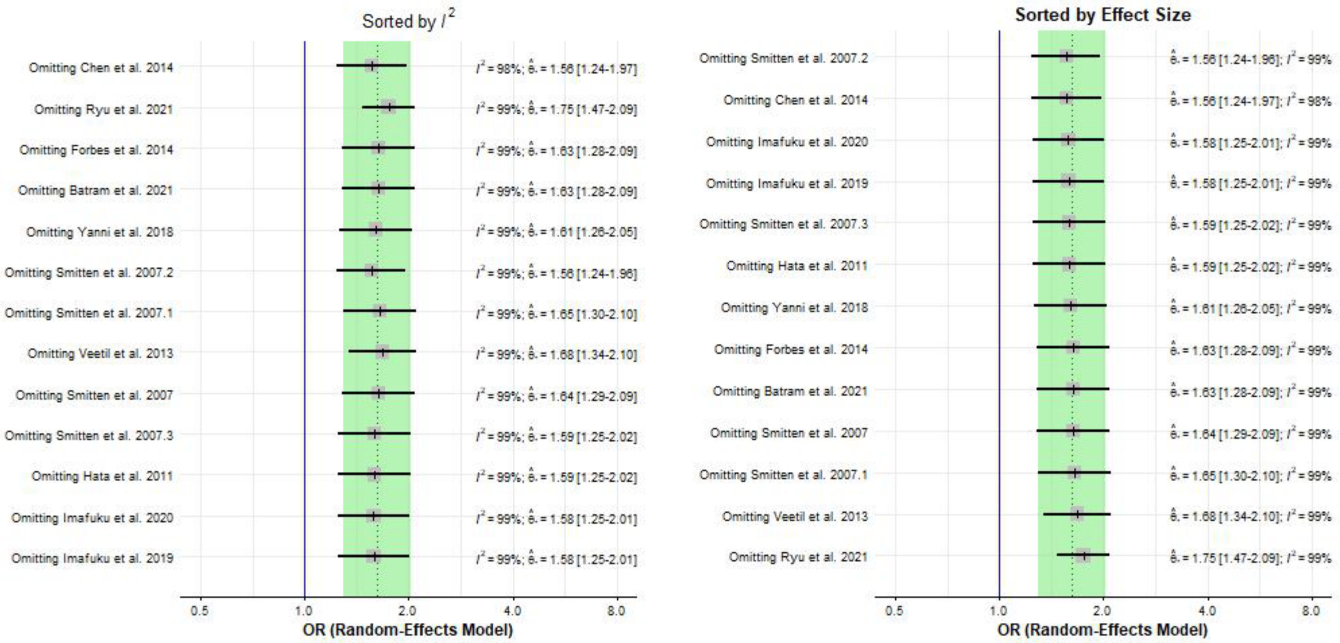

Figure 3: Forest plots for Leave-One-Out Analyses.

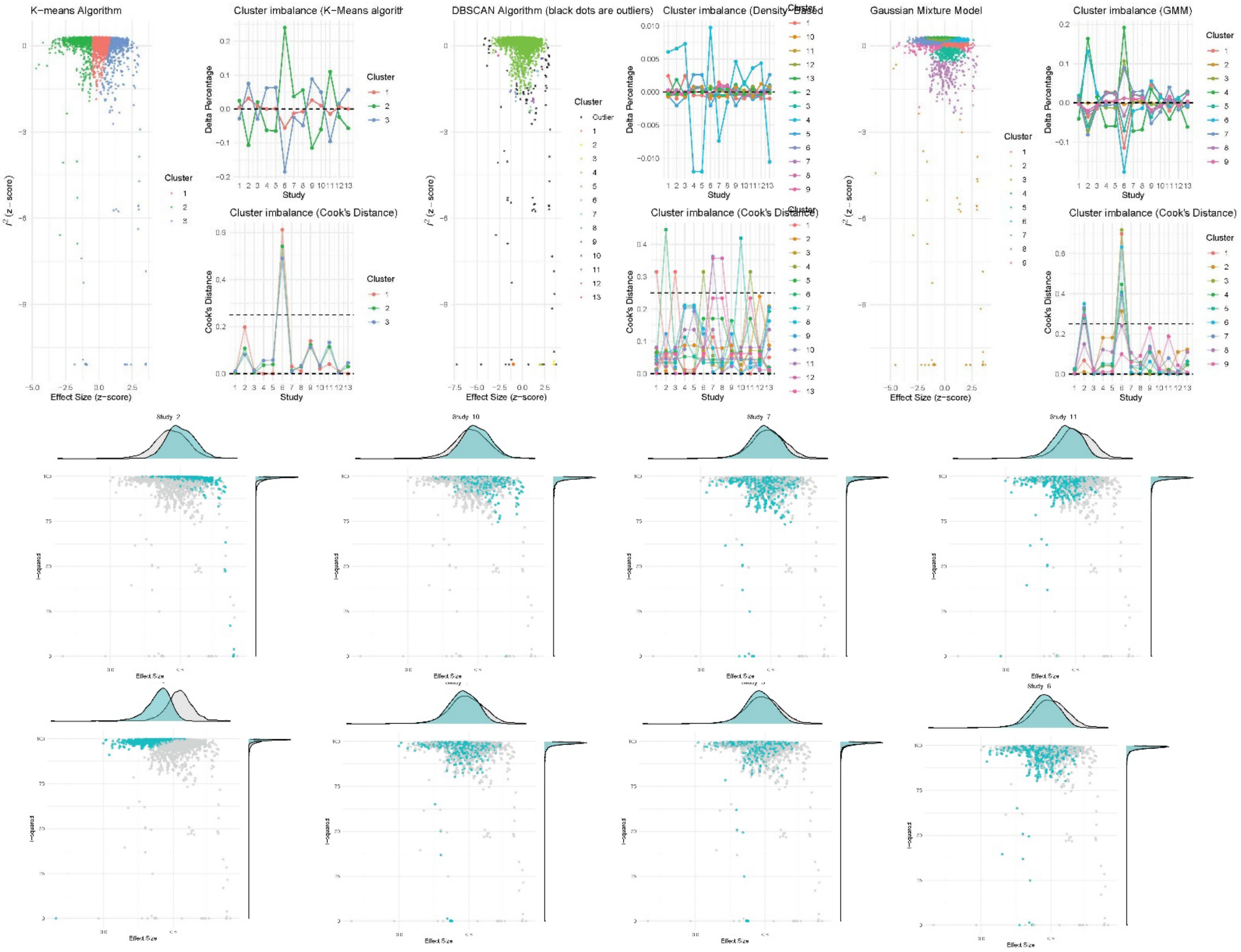

Figure 4: GOSH Plots and GOSH Plot Diagnostics.

Transplantation

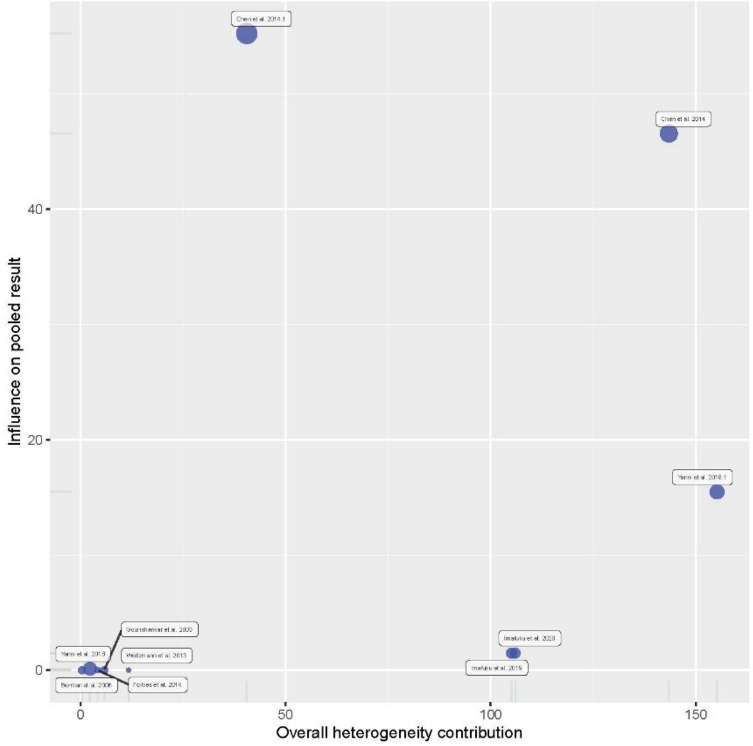

Figure 1: Baujat plot.

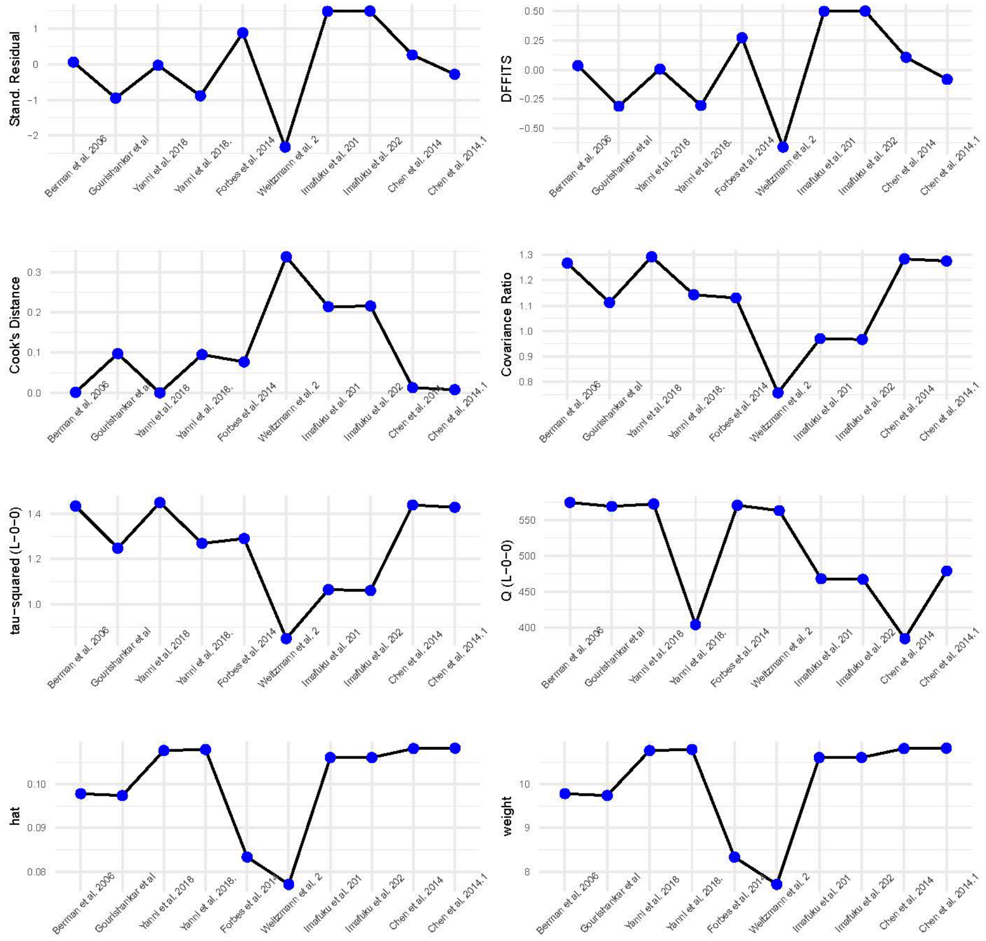

Figure 2: Influence diagnostics.

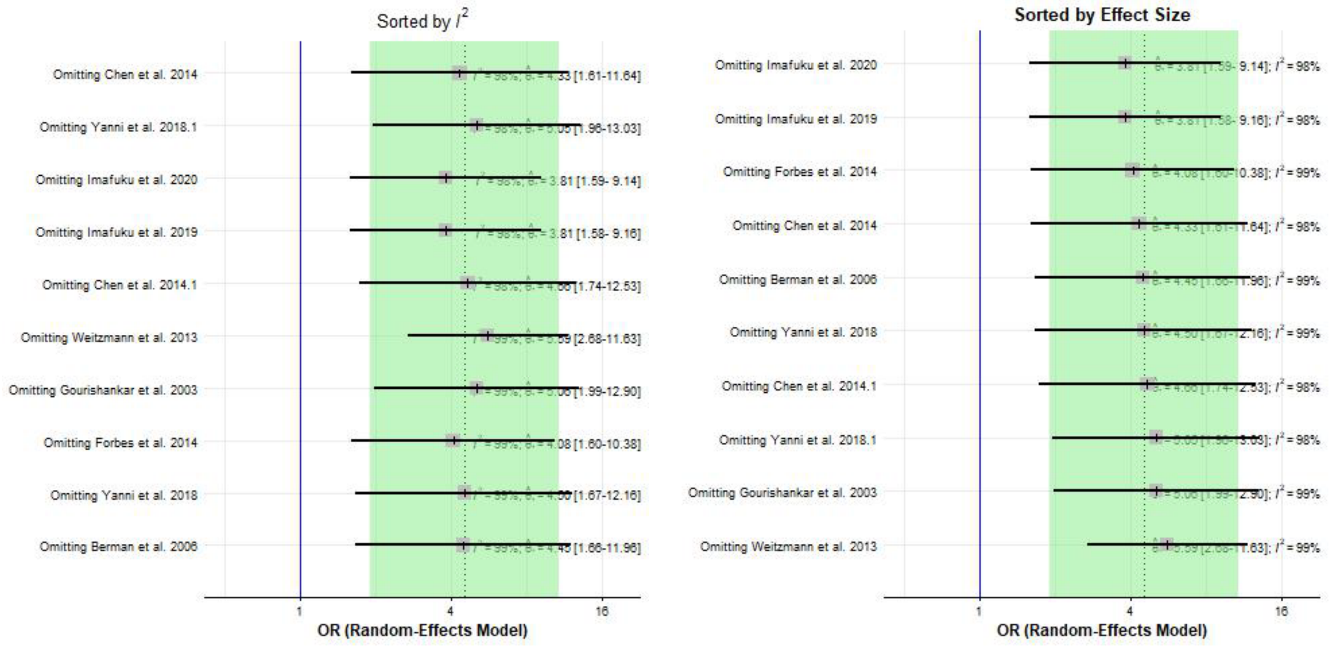

Figure 3: Forest plots for Leave-One-Out Analyses.

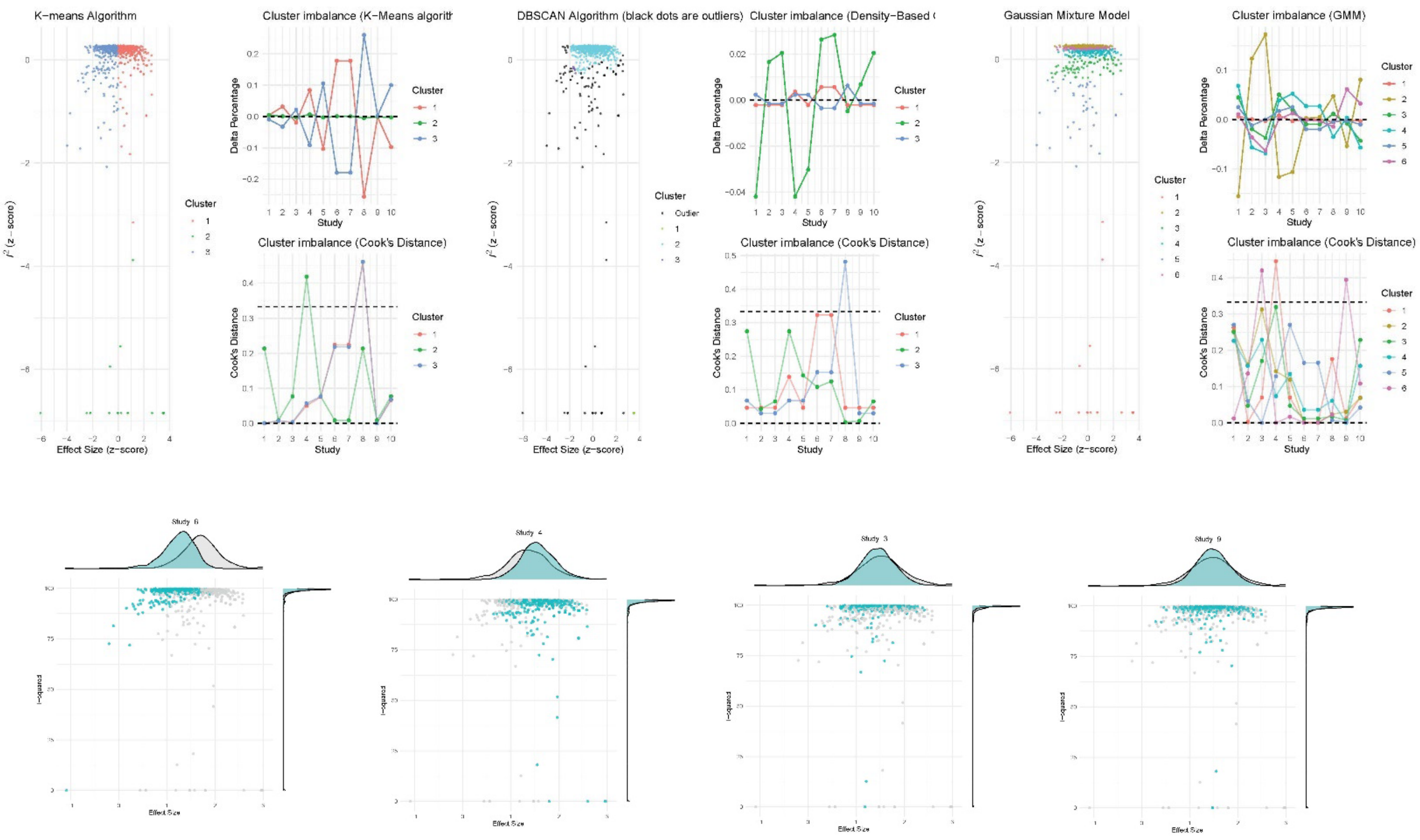

Figure 4: GOSH Plots and GOSH Plot Diagnostics.

HIV

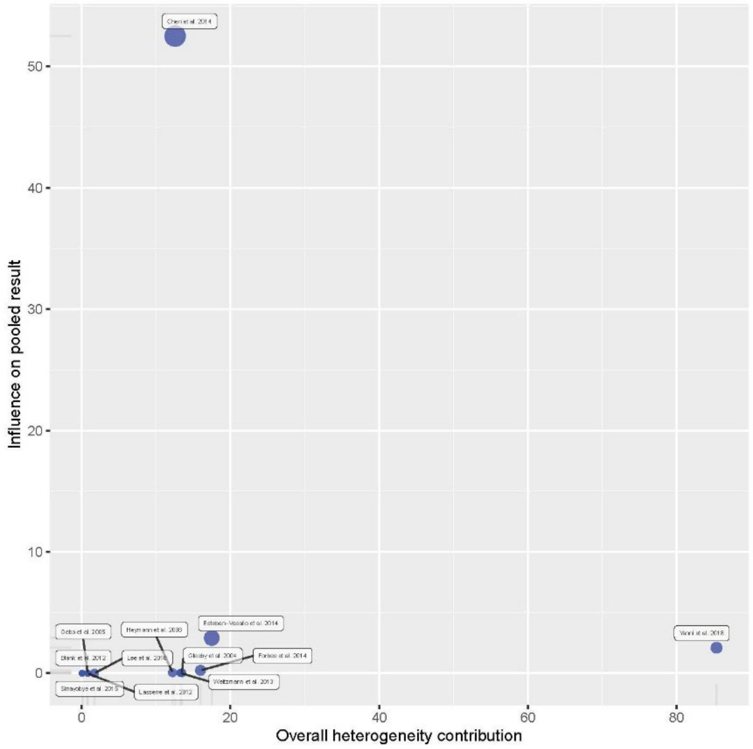

Figure 1: Baujat plot.

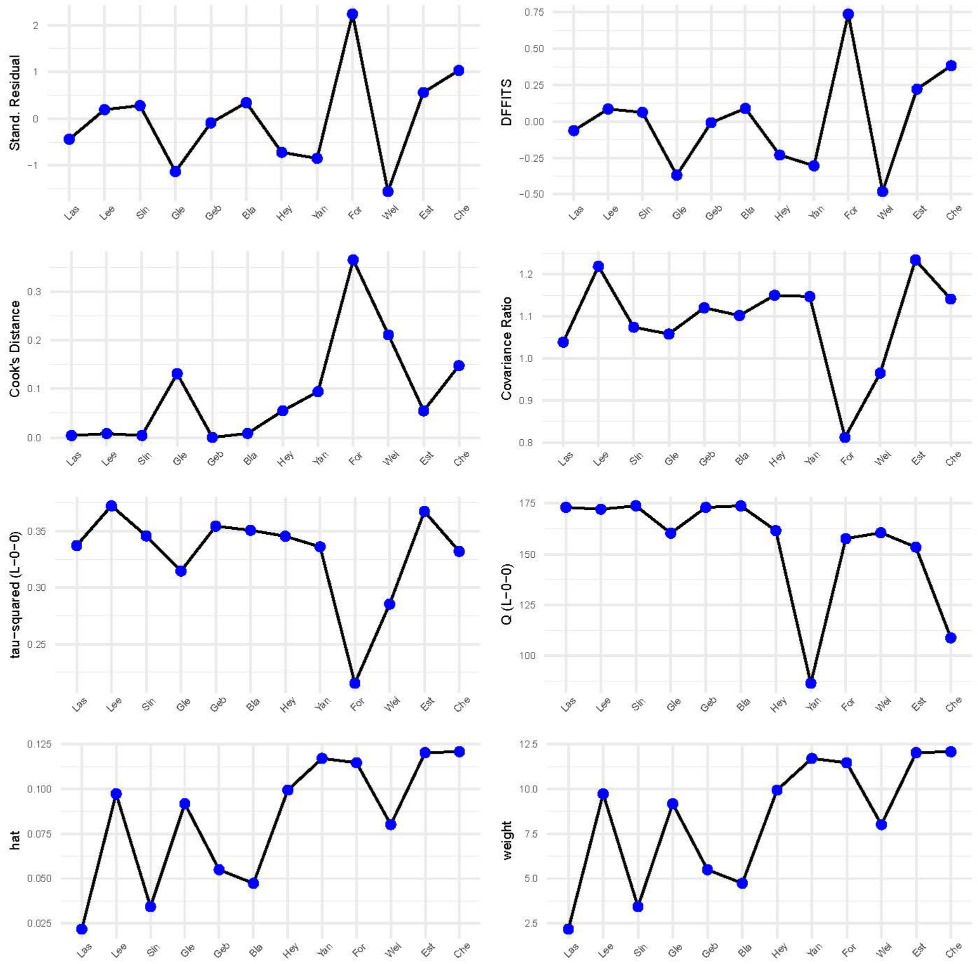

Figure 2: Influence diagnostics.

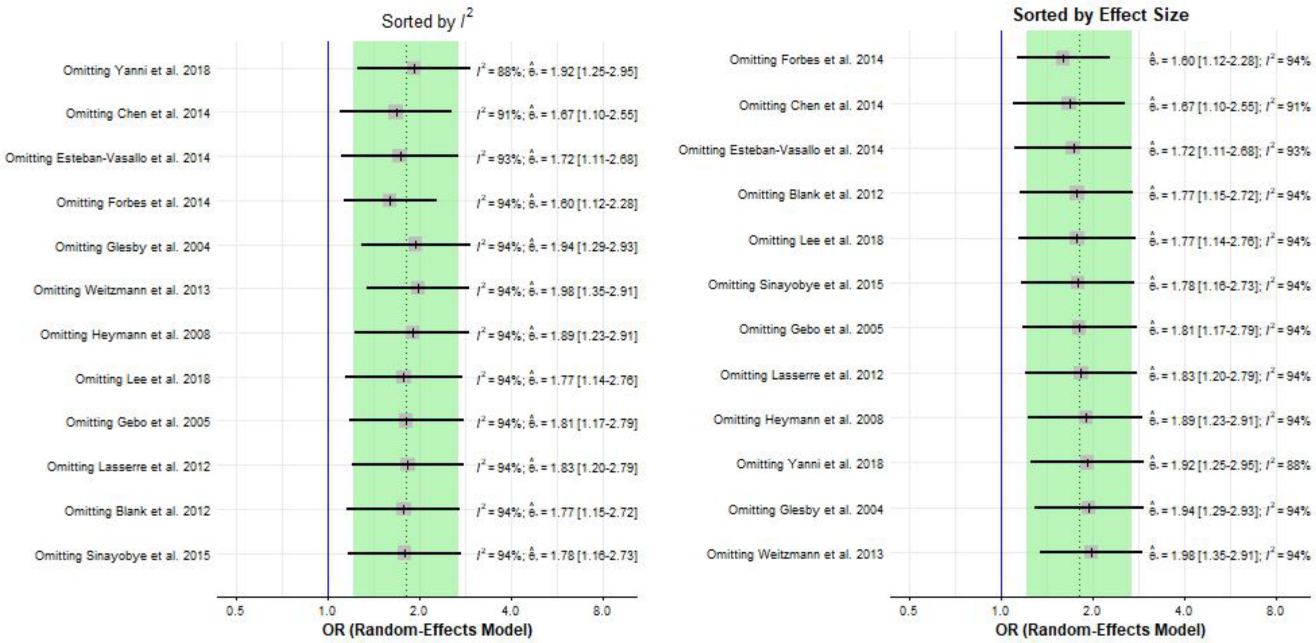

Figure 3: Forest plots for Leave-One-Out Analyses.

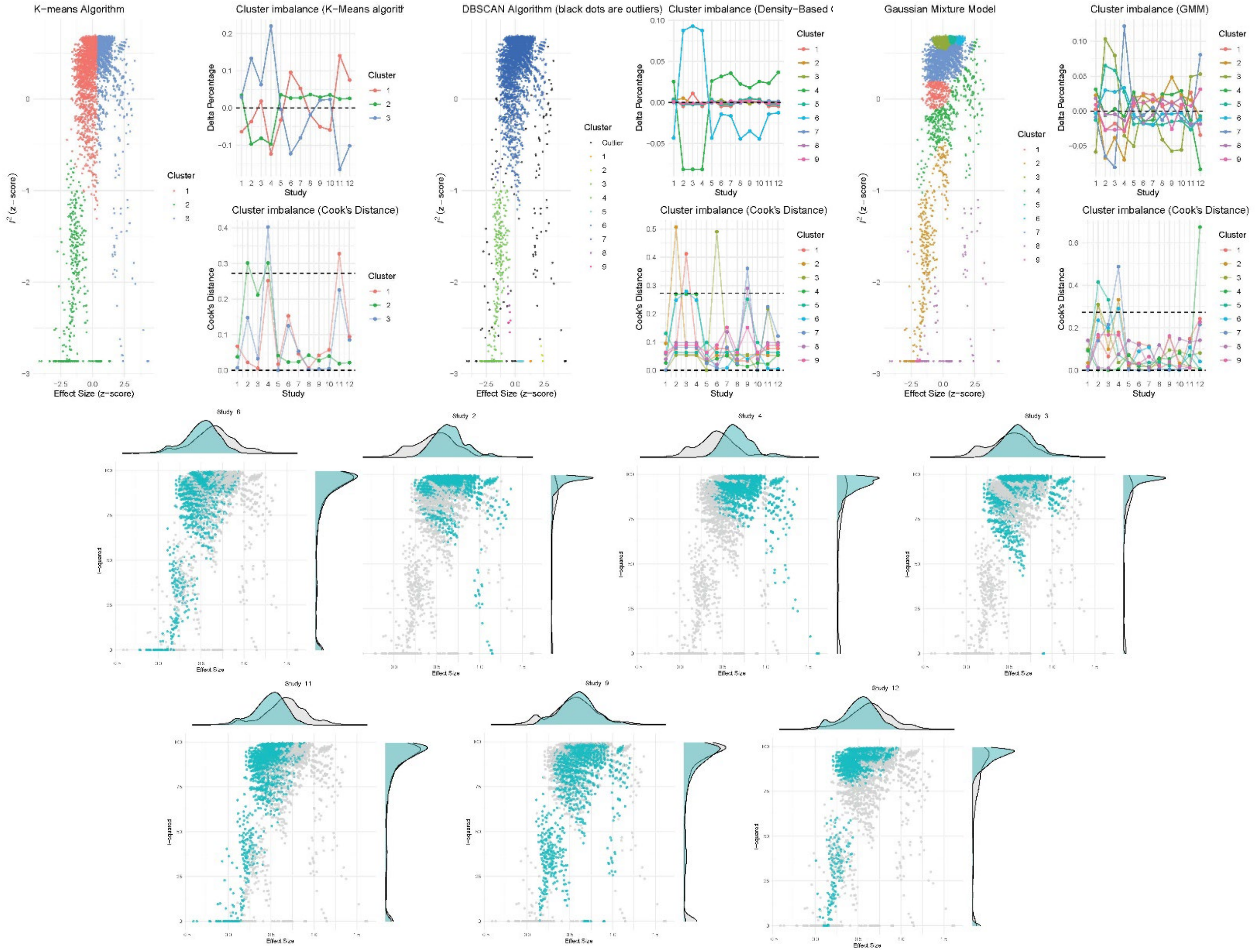

Figure 4: GOSH Plots and GOSH Plot Diagnostics.

Digestive disorders

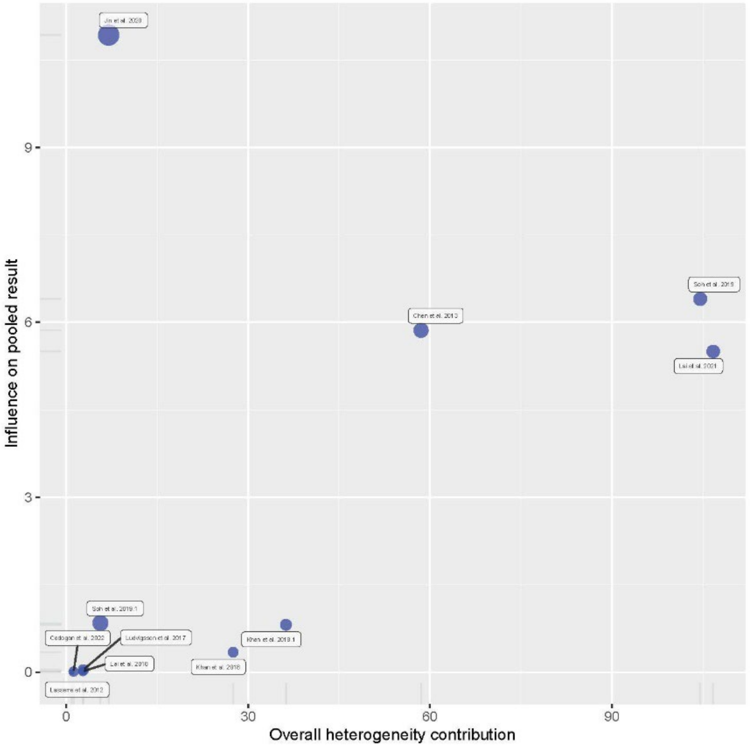

Figure 1: Baujat plot.

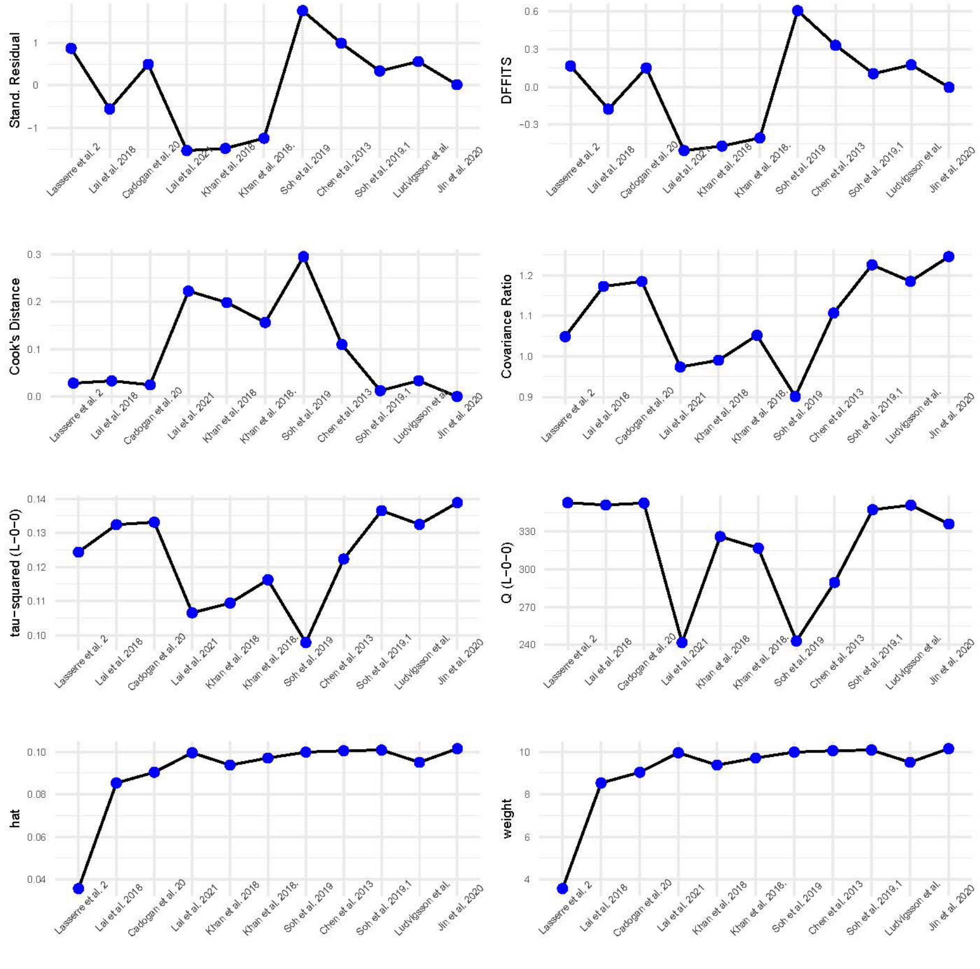

Figure 2: Influence diagnostics.

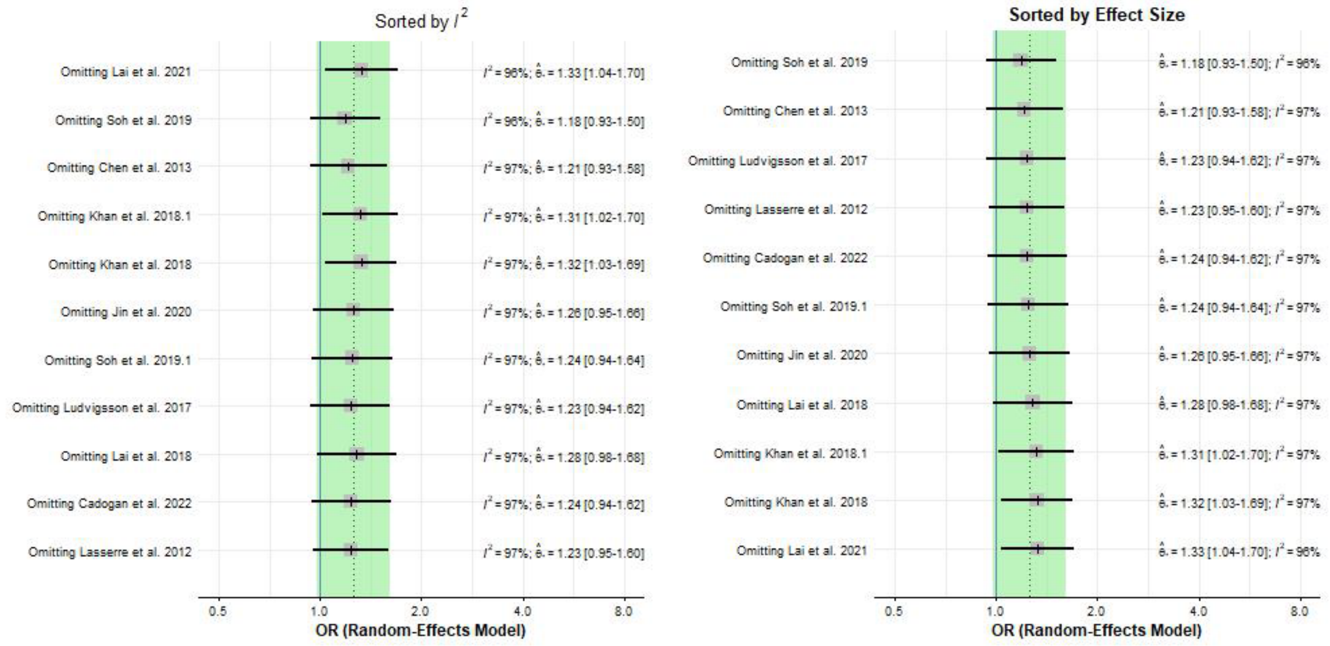

Figure 3: Forest plots for Leave-One-Out Analyses.

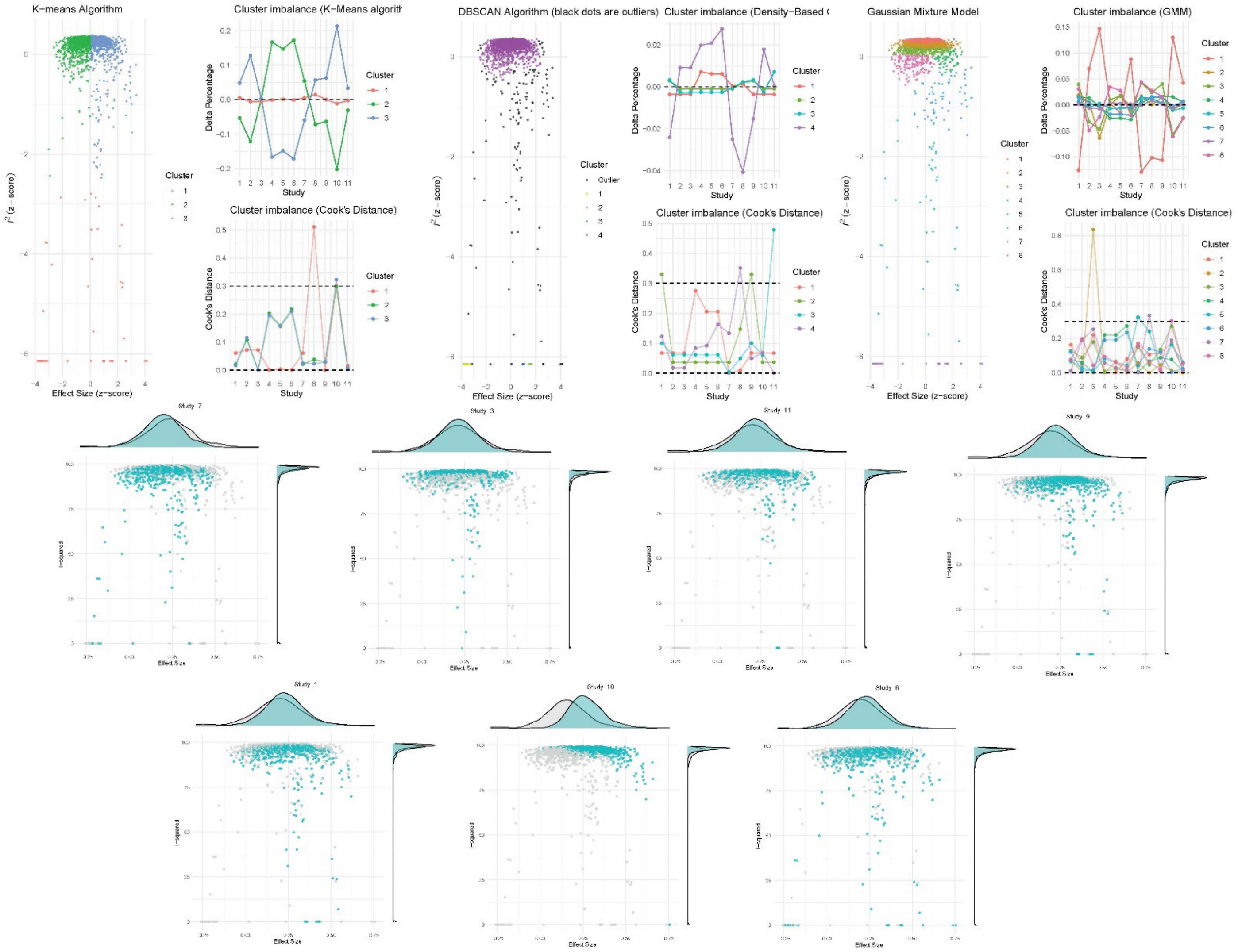

Figure 4: GOSH Plots and GOSH Plot Diagnostics.

Mental health condition

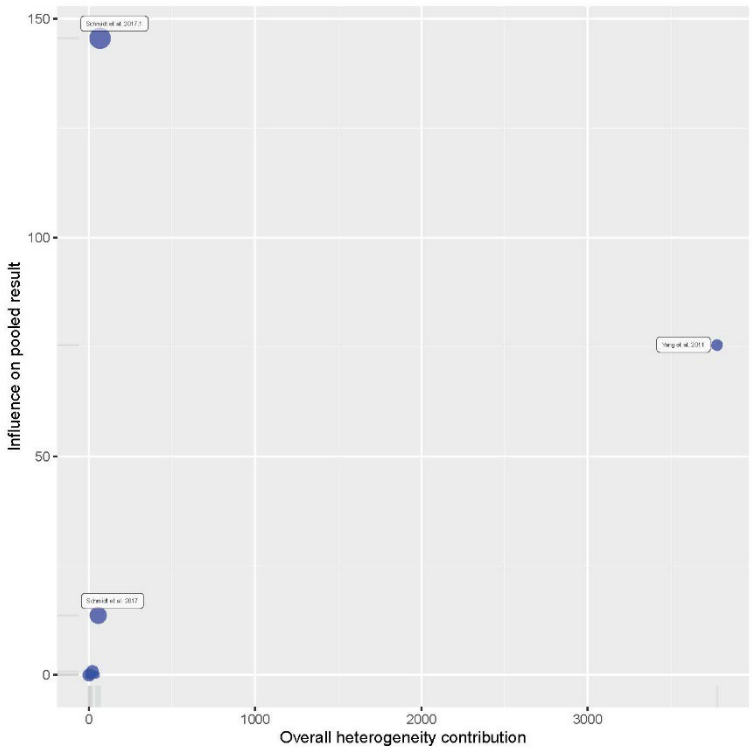

Figure 1: Baujat plot.

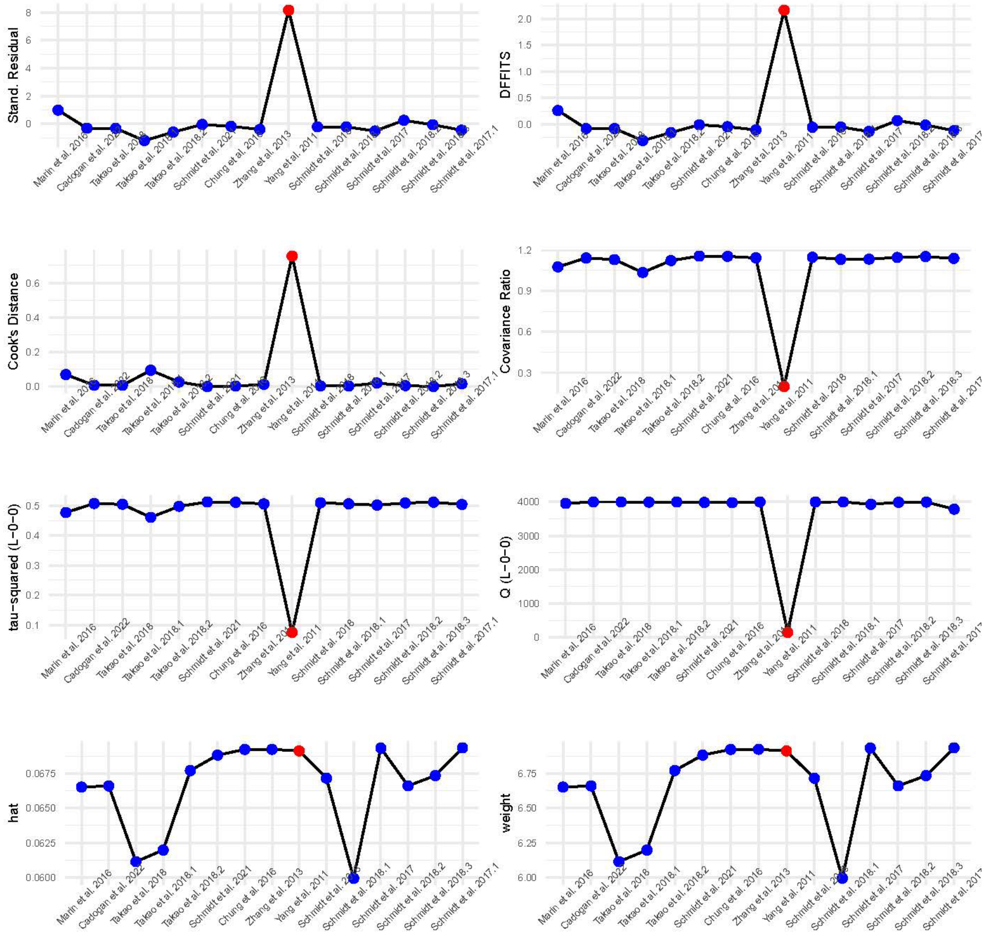

Figure 2: Influence diagnostics.

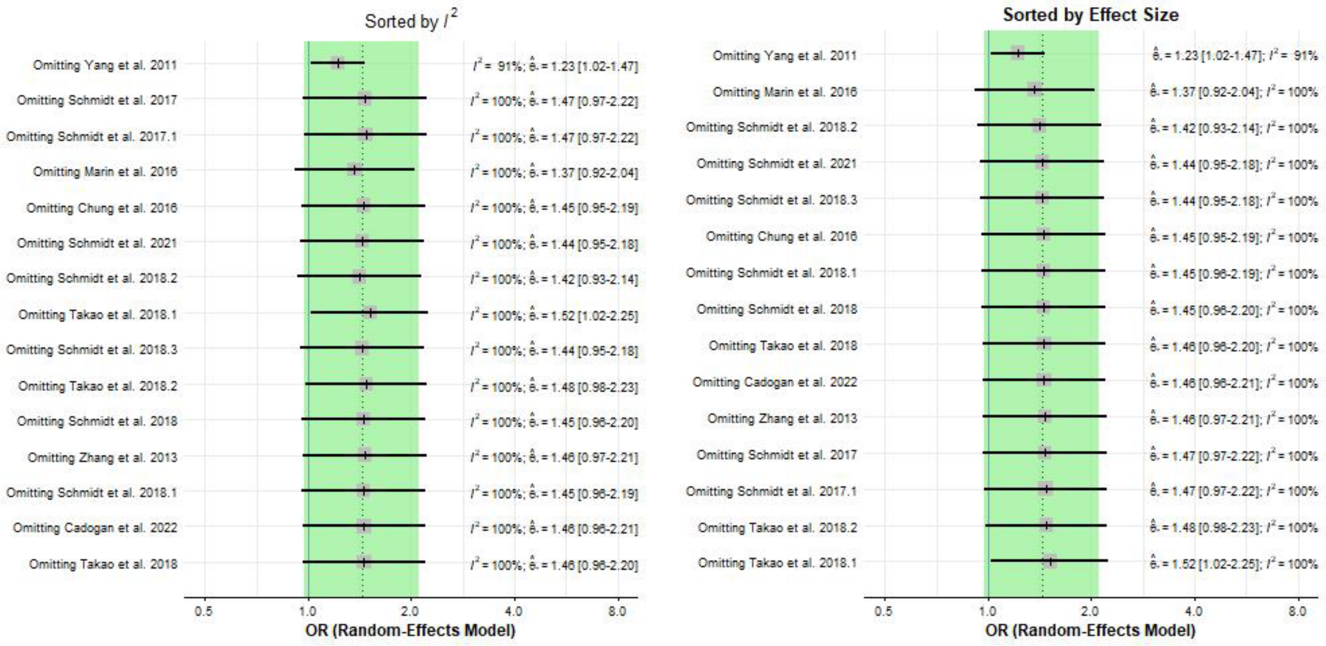

Figure 3: Forest plots for Leave-One-Out Analyses.

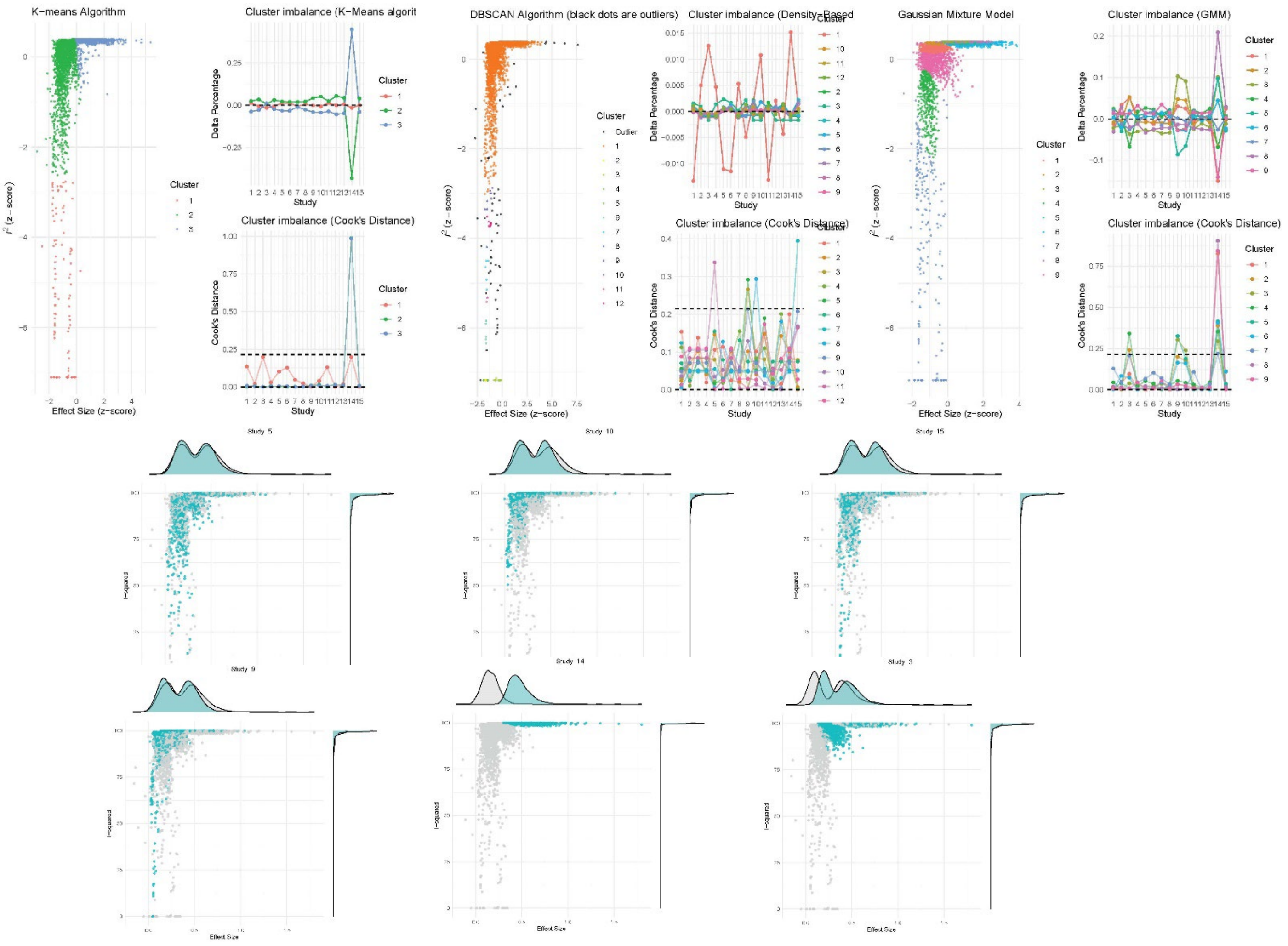

Figure 4: GOSH Plots and GOSH Plot Diagnostics.

SLE

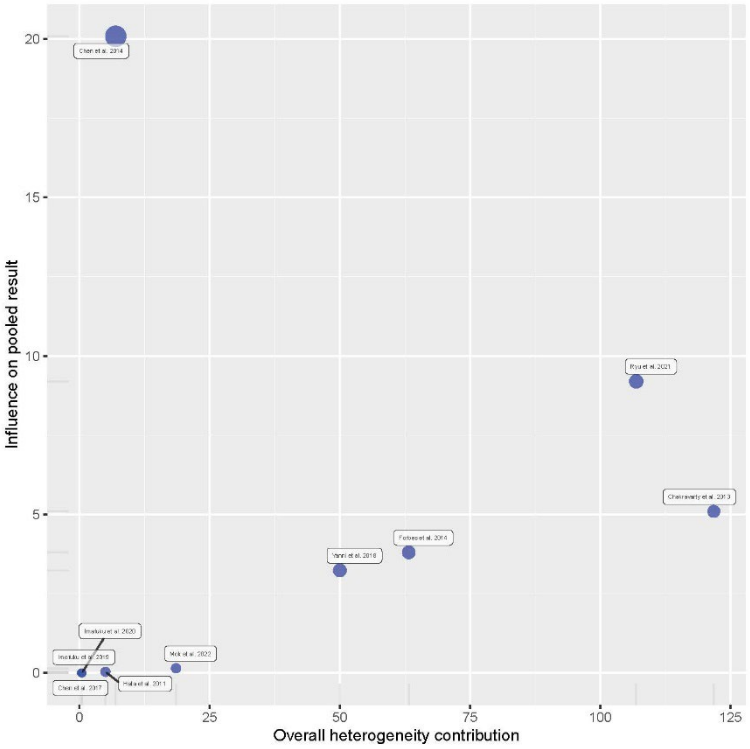

Figure 1: Baujat plot.

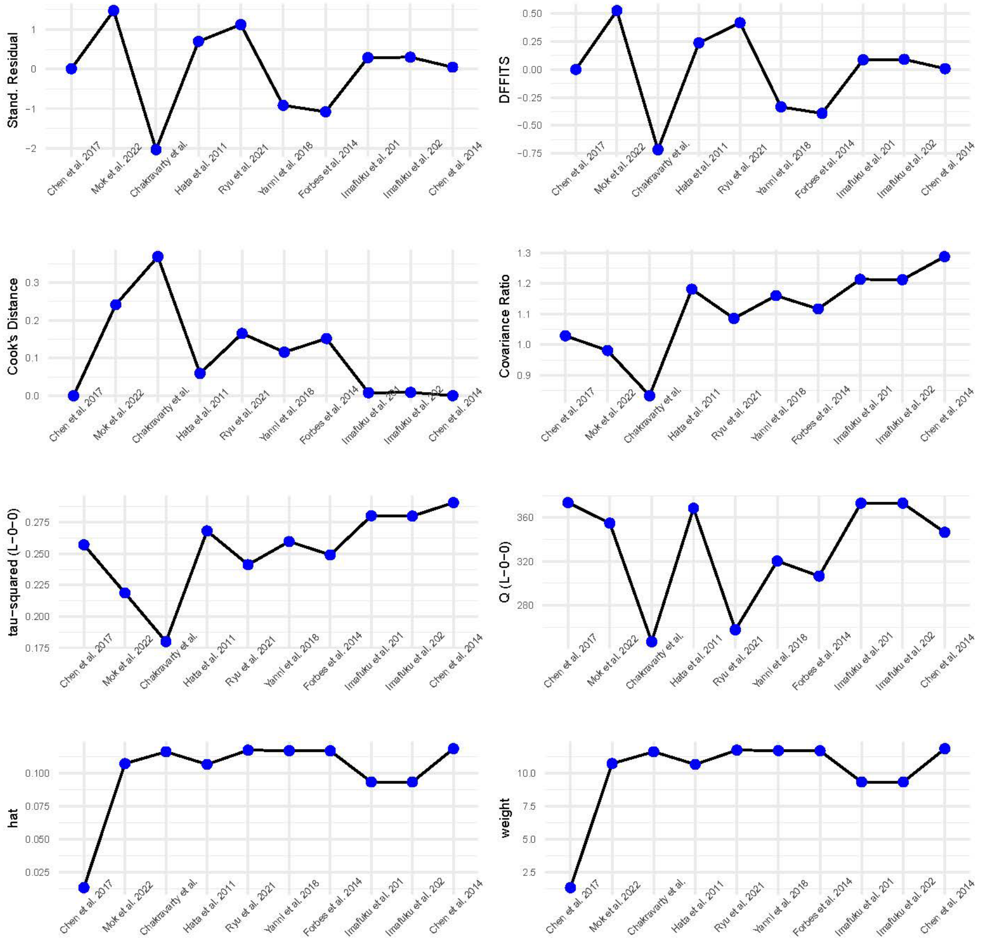

Figure 2: Influence diagnostics.

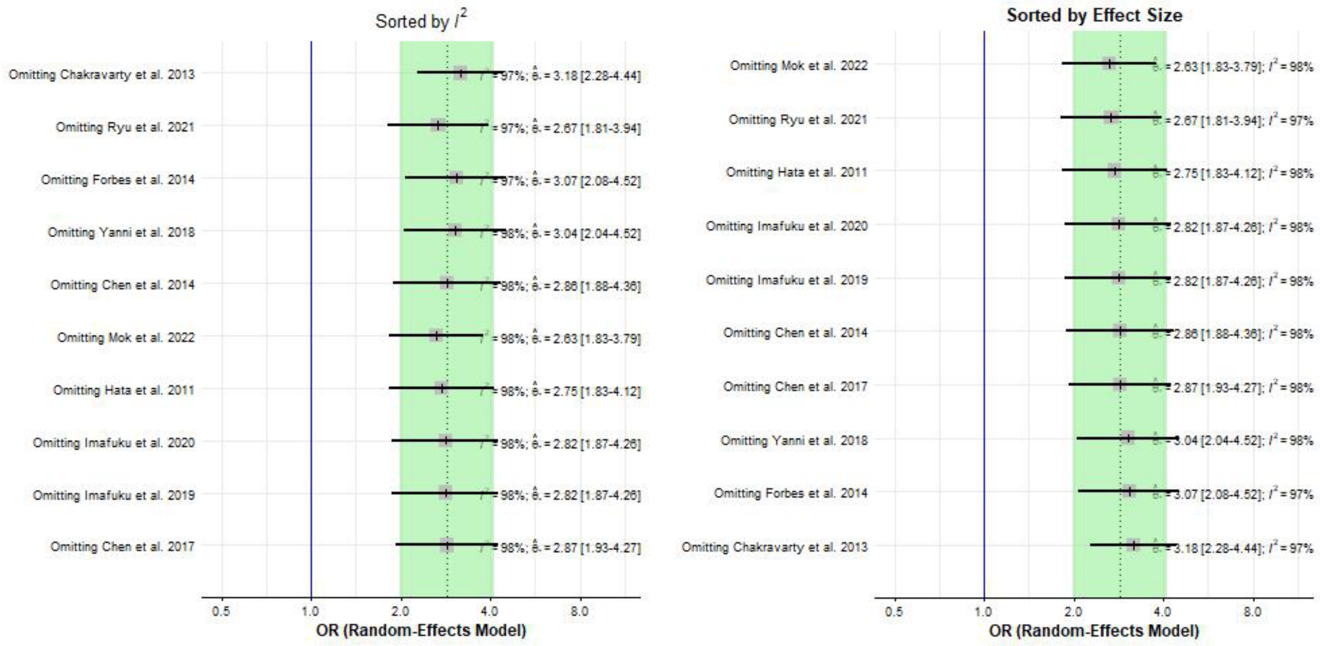

Figure 3: Forest plots for Leave-One-Out Analyses.

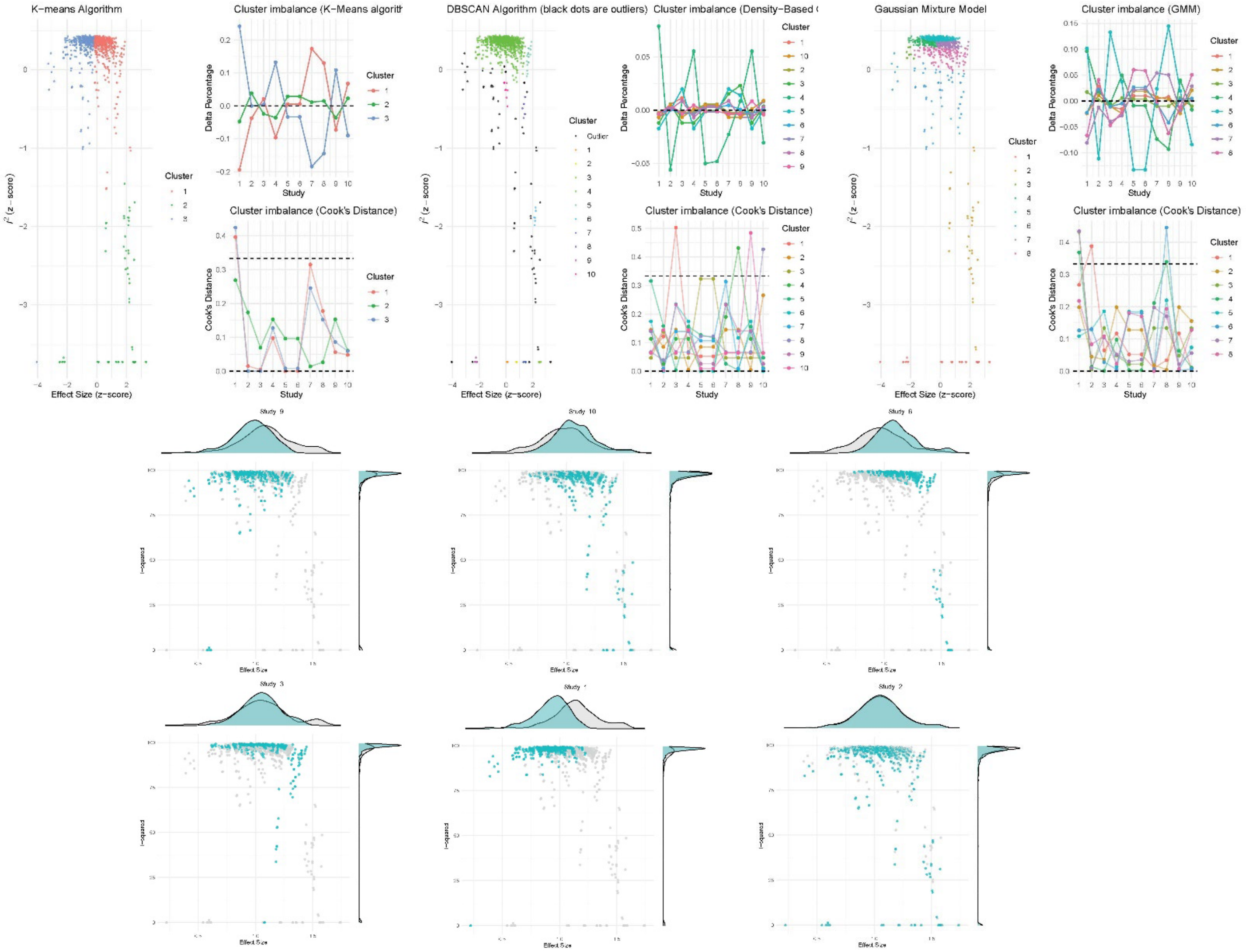

Figure 4: GOSH Plots and GOSH Plot Diagnostics.

Autoimmune disorders

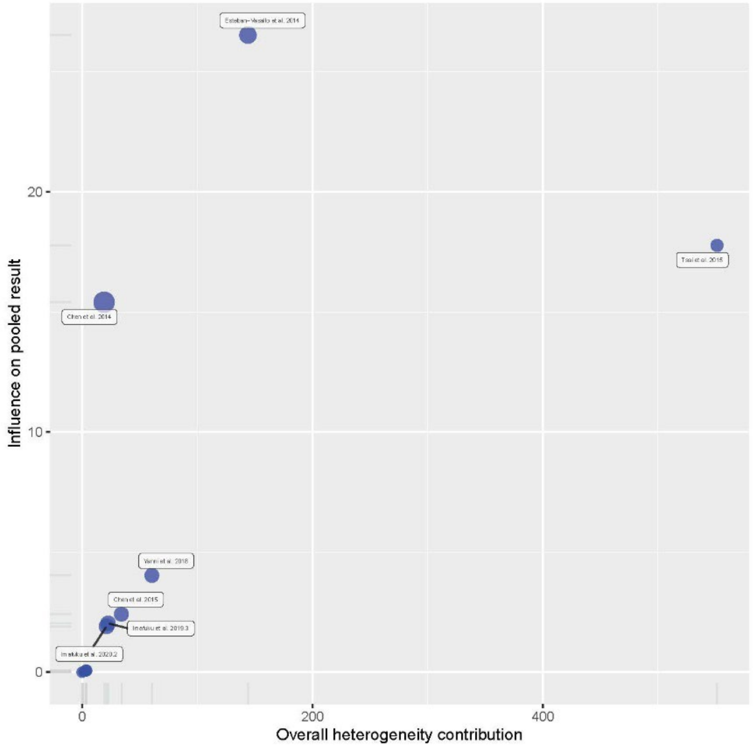

Figure 1: Baujat plot.

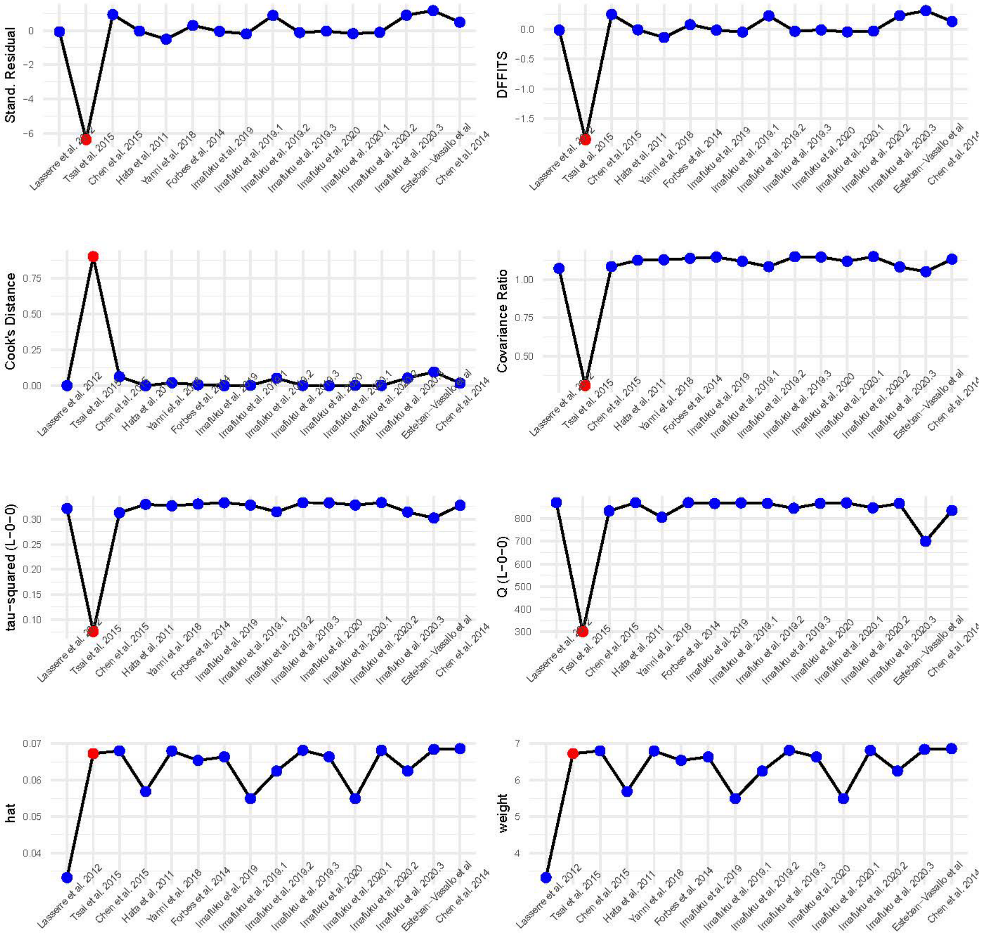

Figure 2: Influence diagnostics.

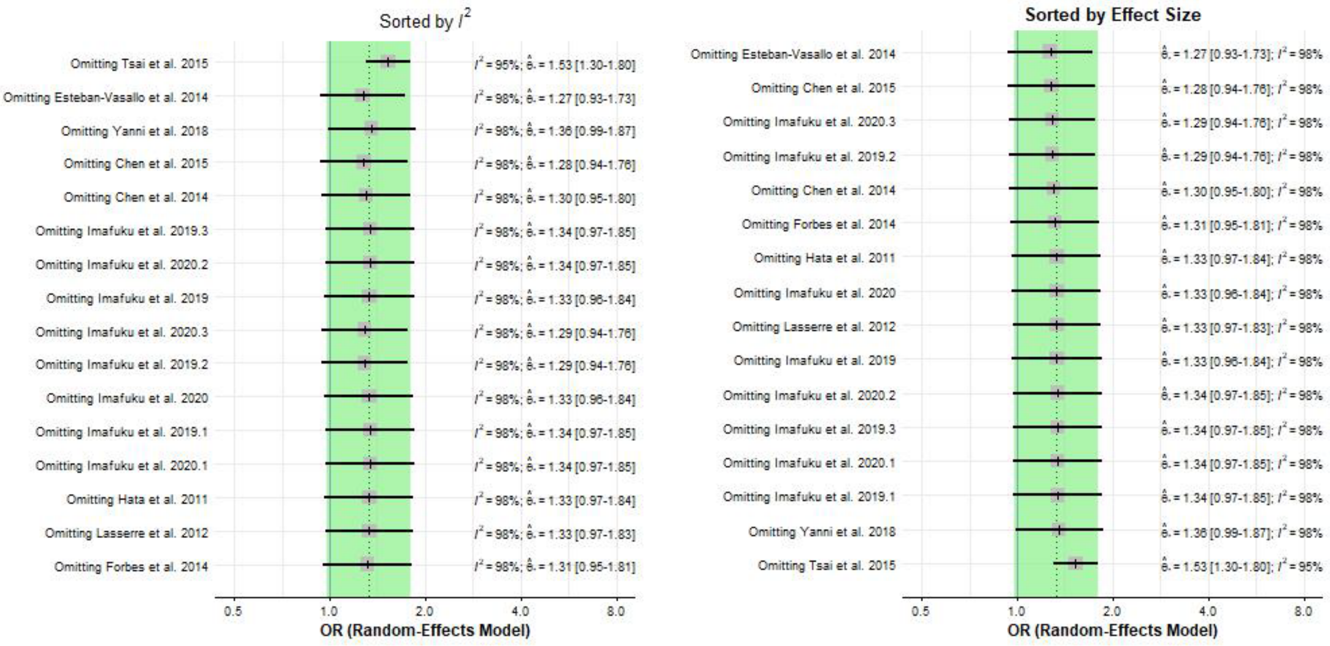

Figure 3: Forest plots for Leave-One-Out Analyses.

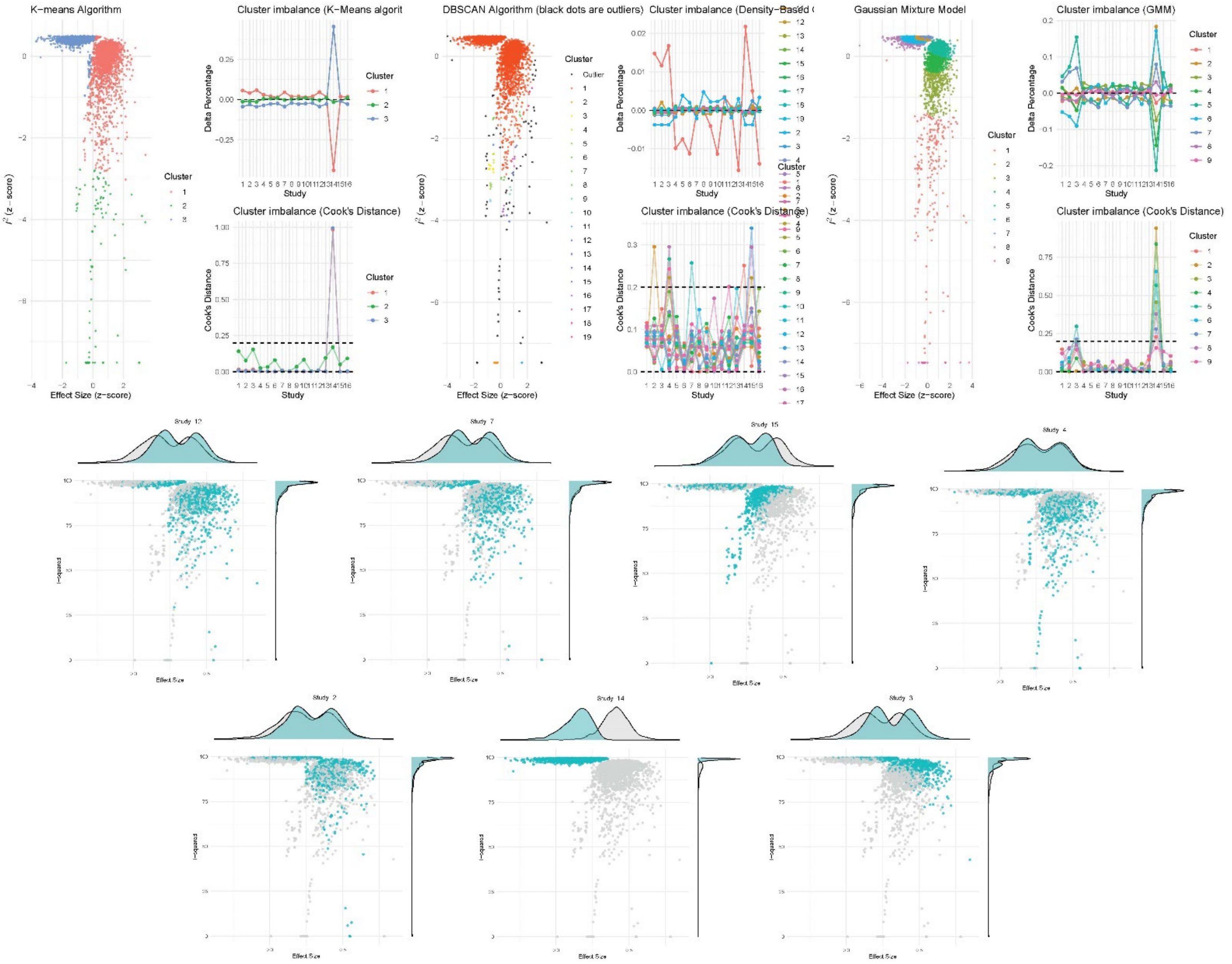

Figure 4: GOSH Plots and GOSH Plot Diagnostics.

Psoriasis

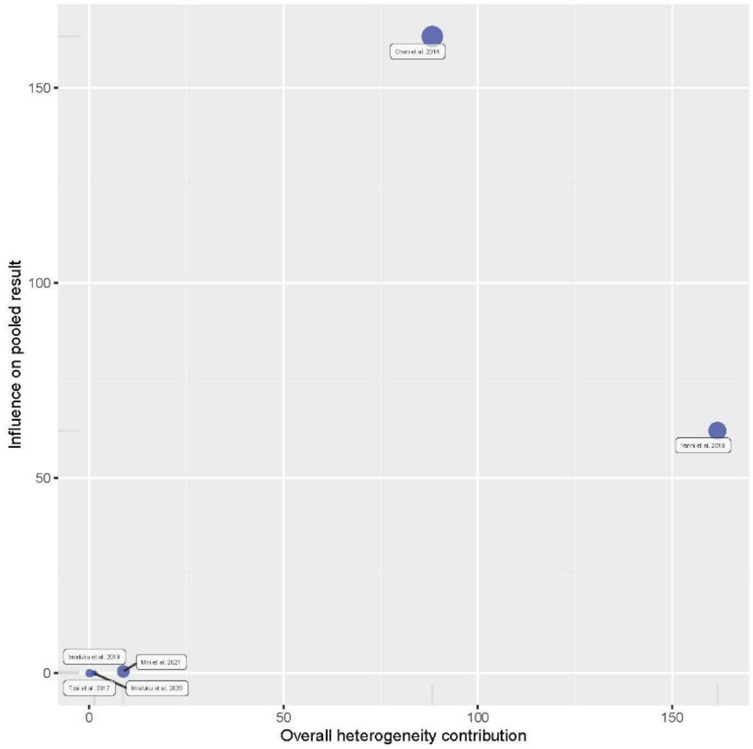

Figure 1: Baujat plot.

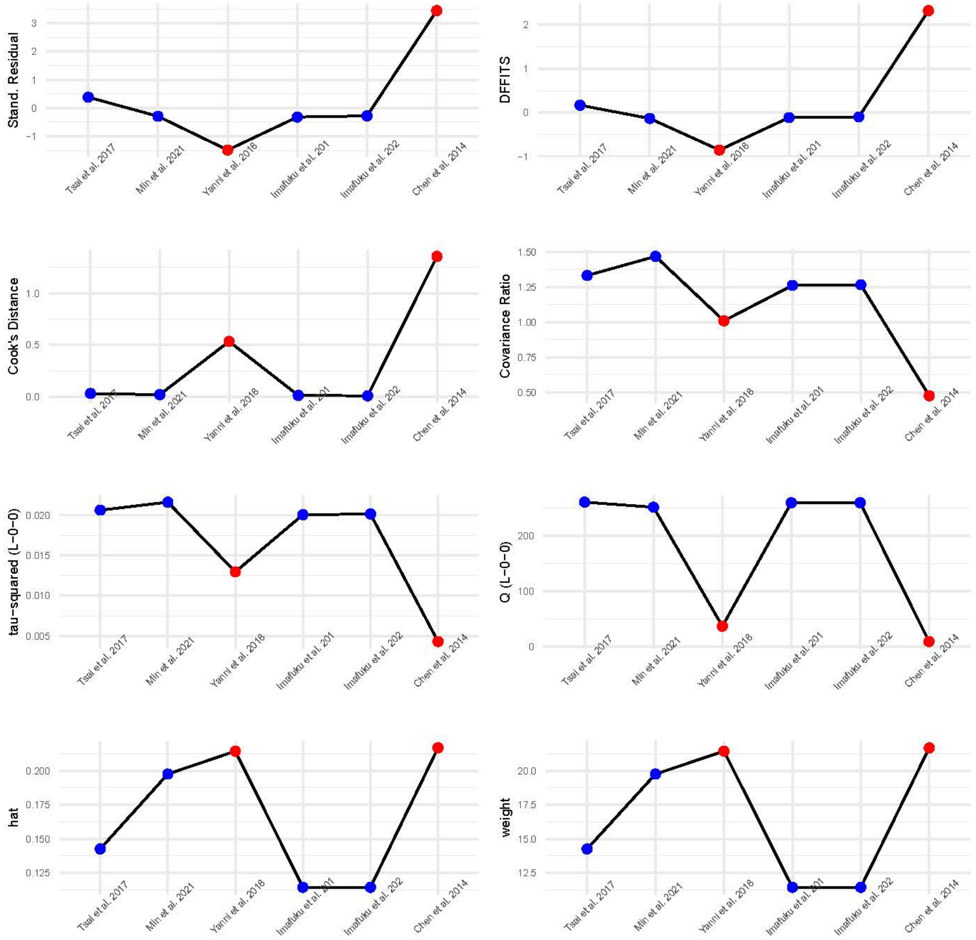

Figure 2: Influence diagnostics.

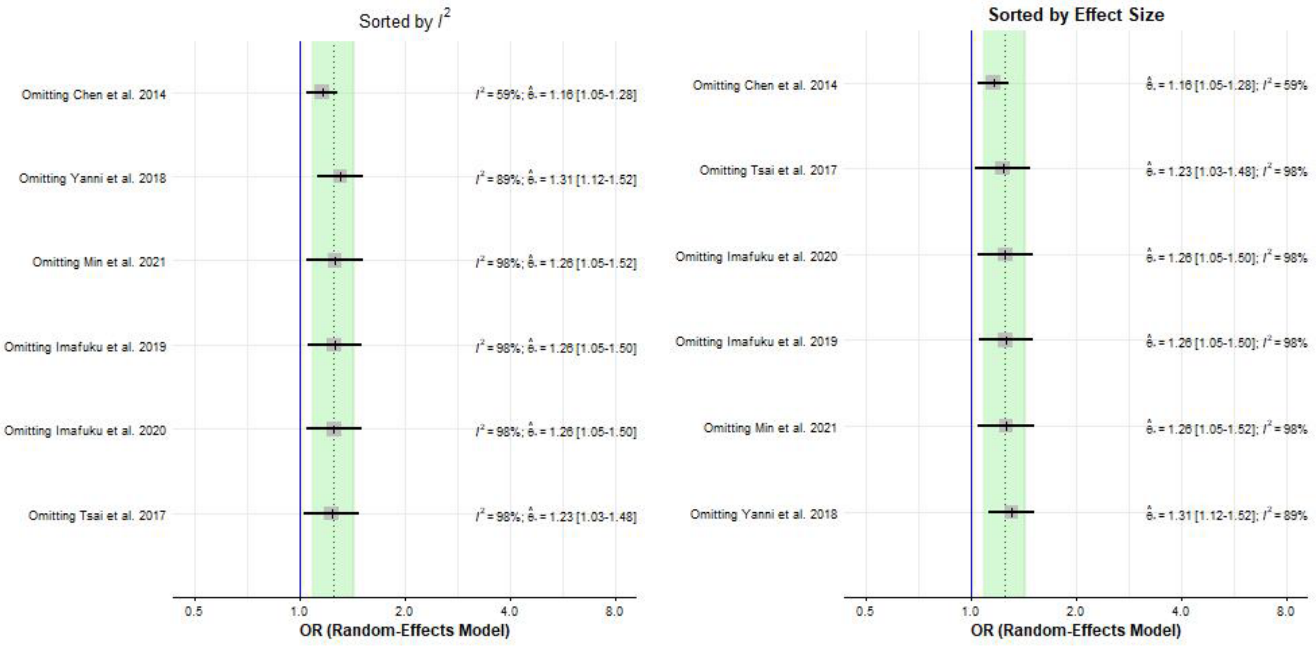

Figure 3: Forest plots for Leave-One-Out Analyses.

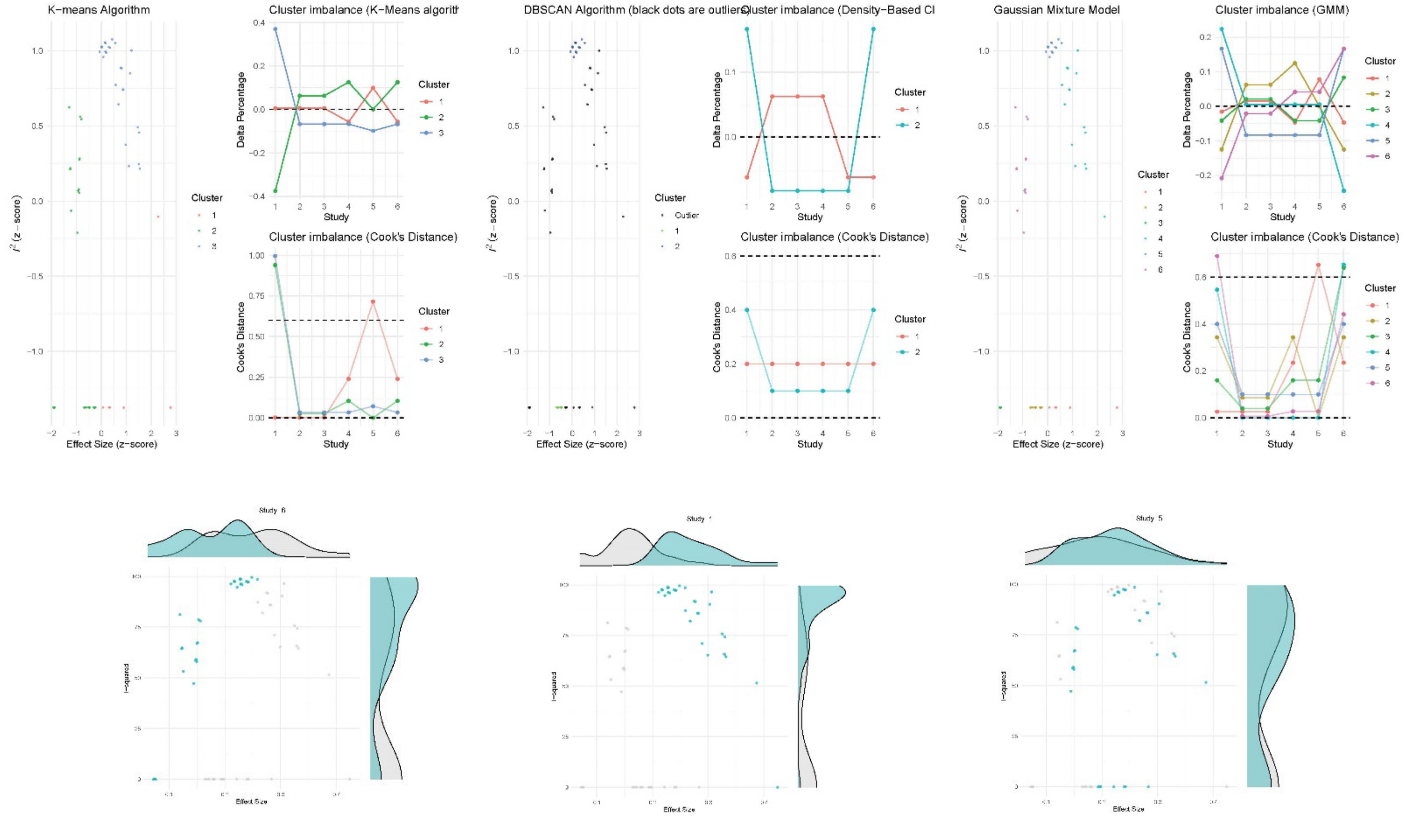

Figure 4: GOSH Plots and GOSH Plot Diagnostics.

IBD

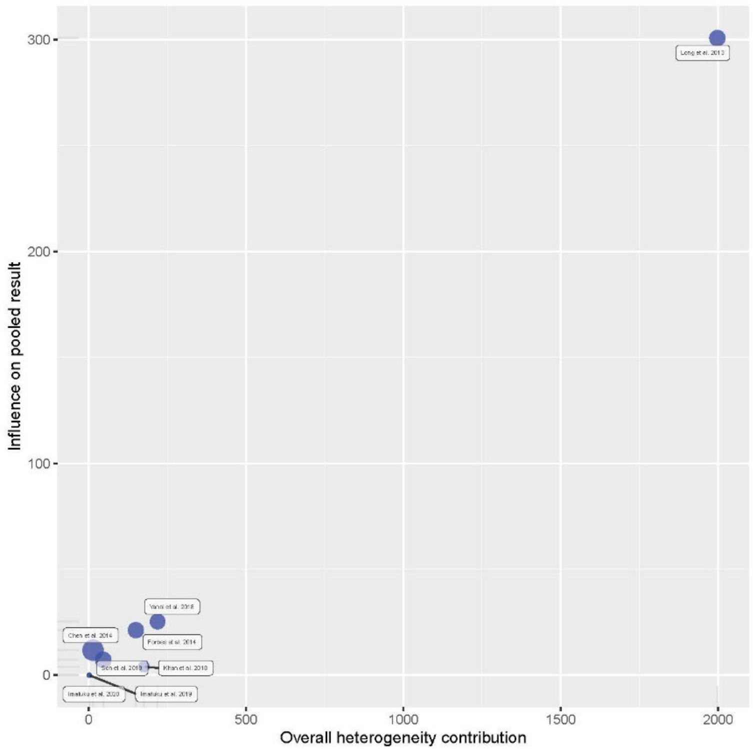

Figure 1: Baujat plot.

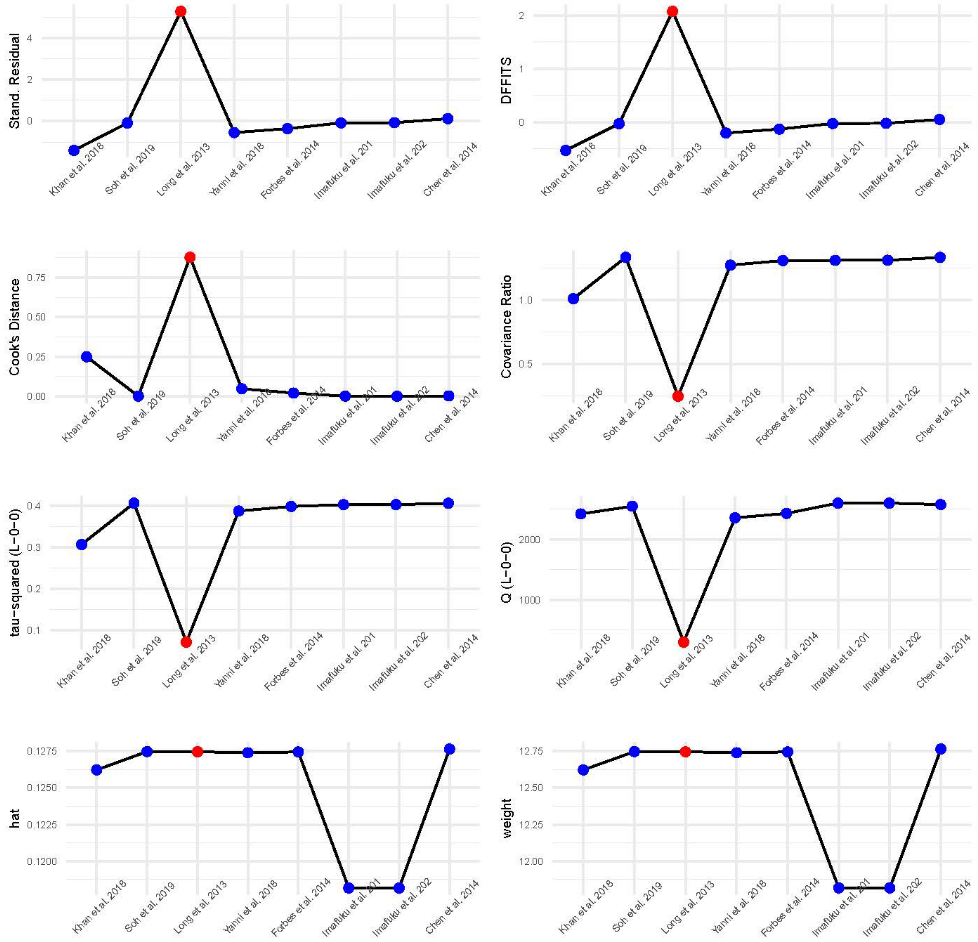

Figure 2: Influence diagnostics.

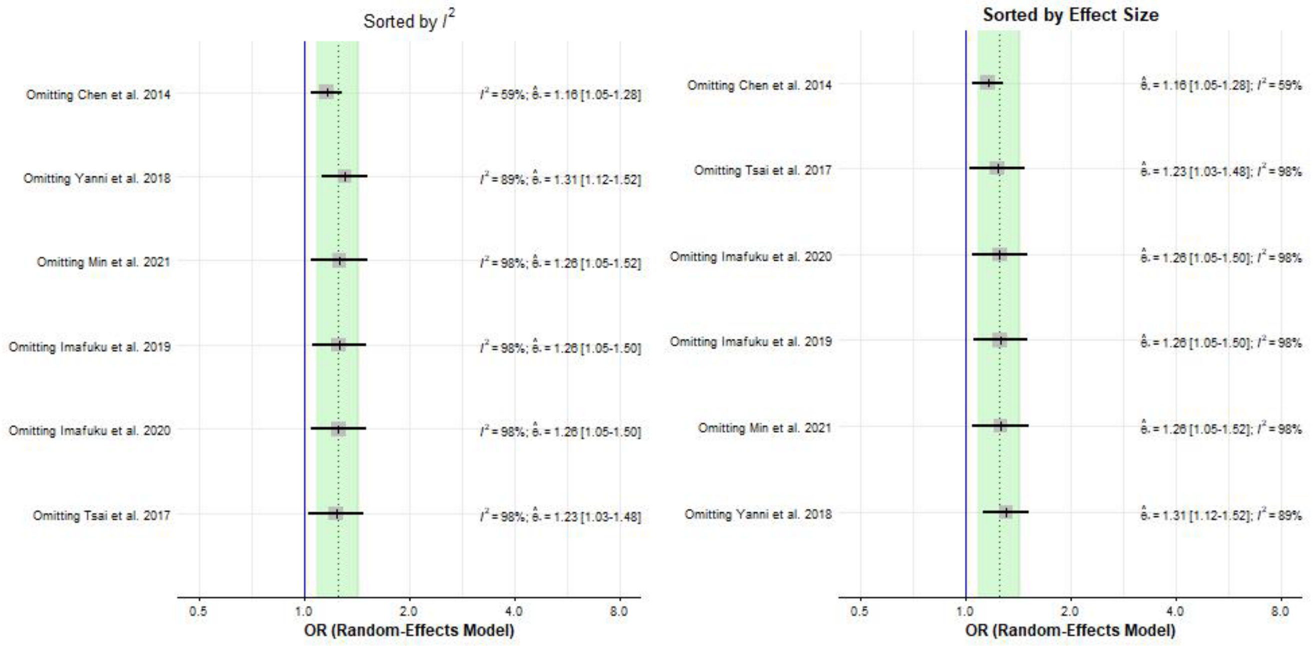

Figure 3: Forest plots for Leave-One-Out Analyses.

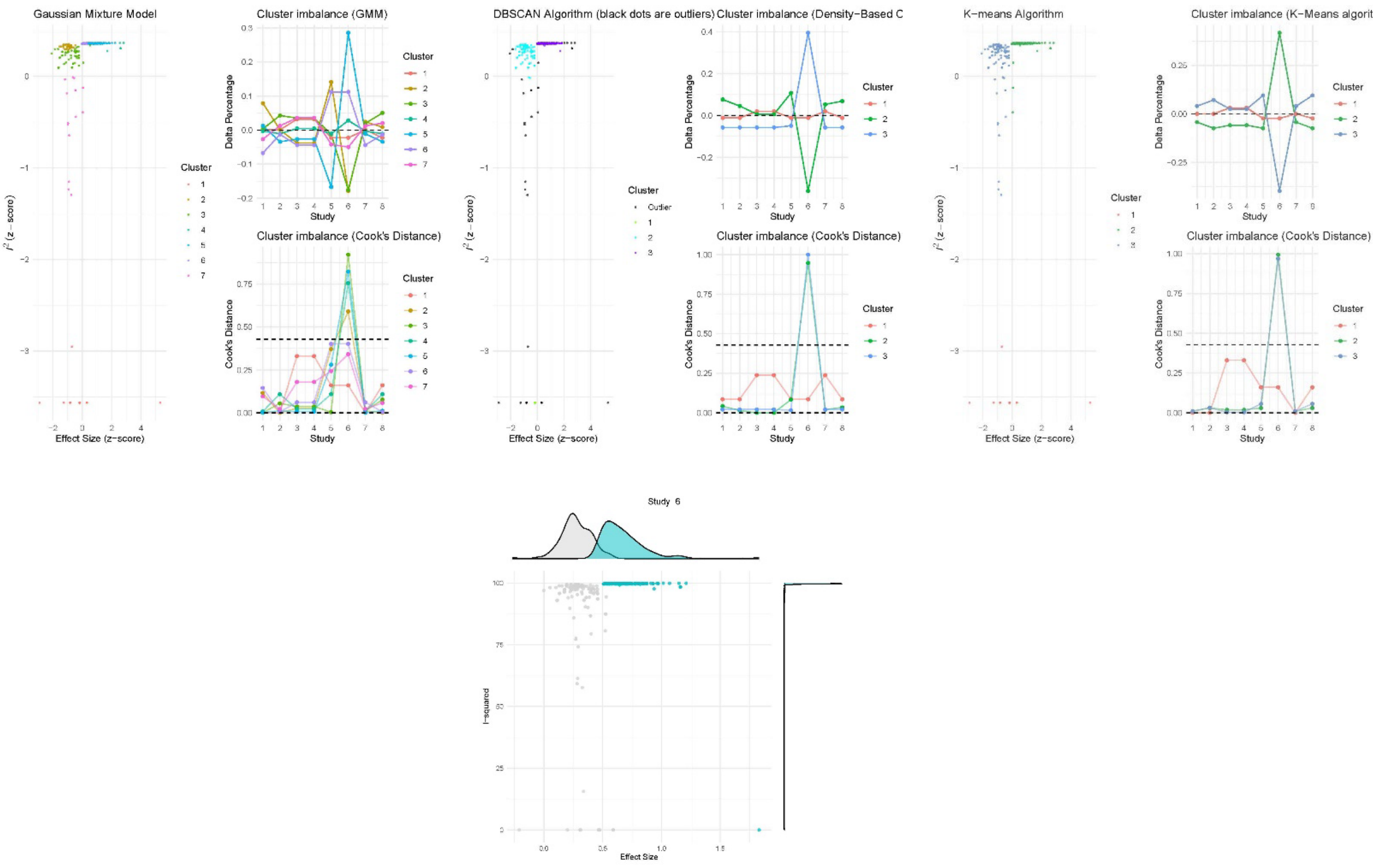

Figure 4: GOSH Plots and GOSH Plot Diagnostics.

Cancer

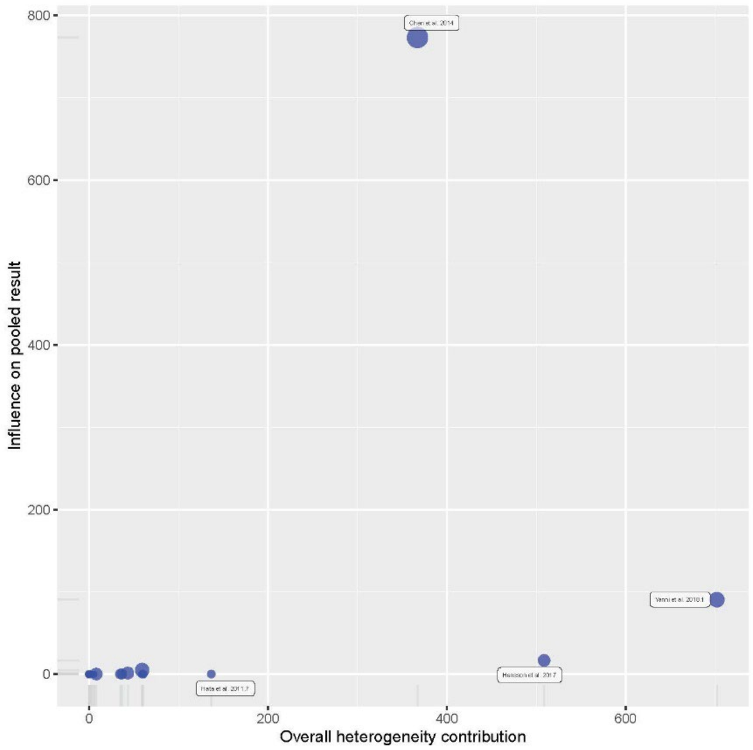

Figure 1: Baujat plot.

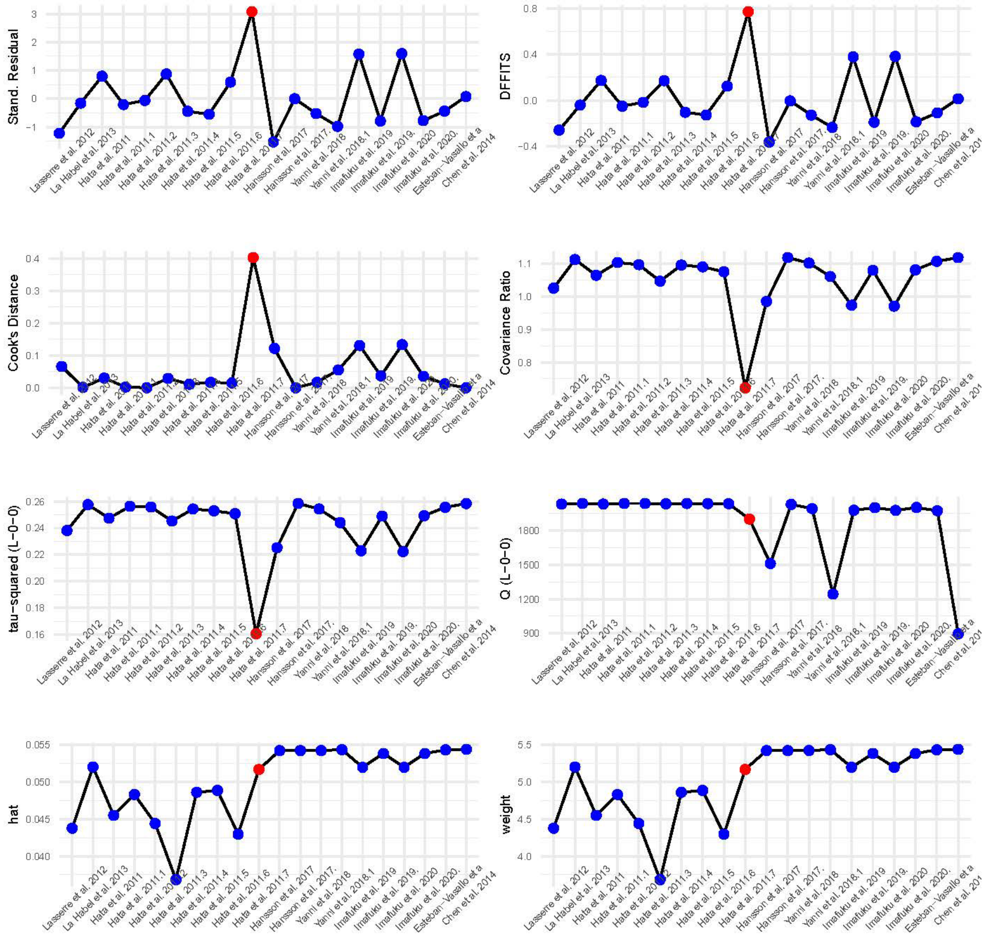

Figure 2: Influence diagnostics.

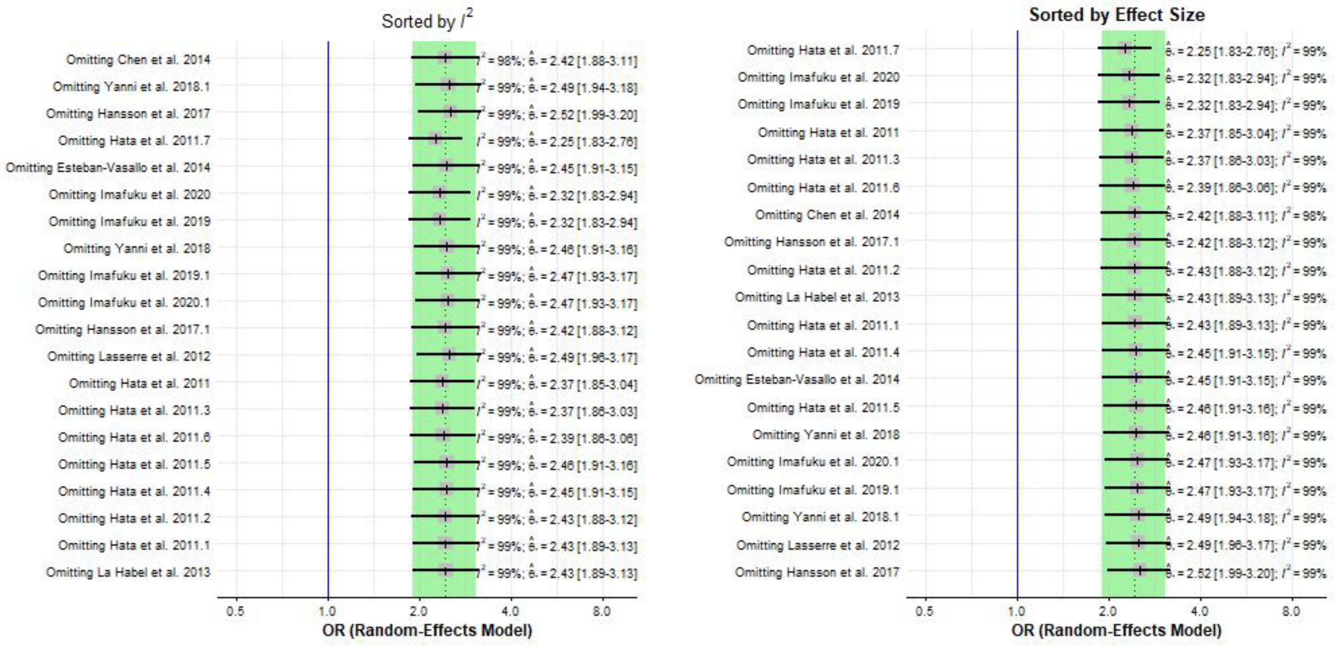

Figure 3: Forest plots for Leave-One-Out Analyses.

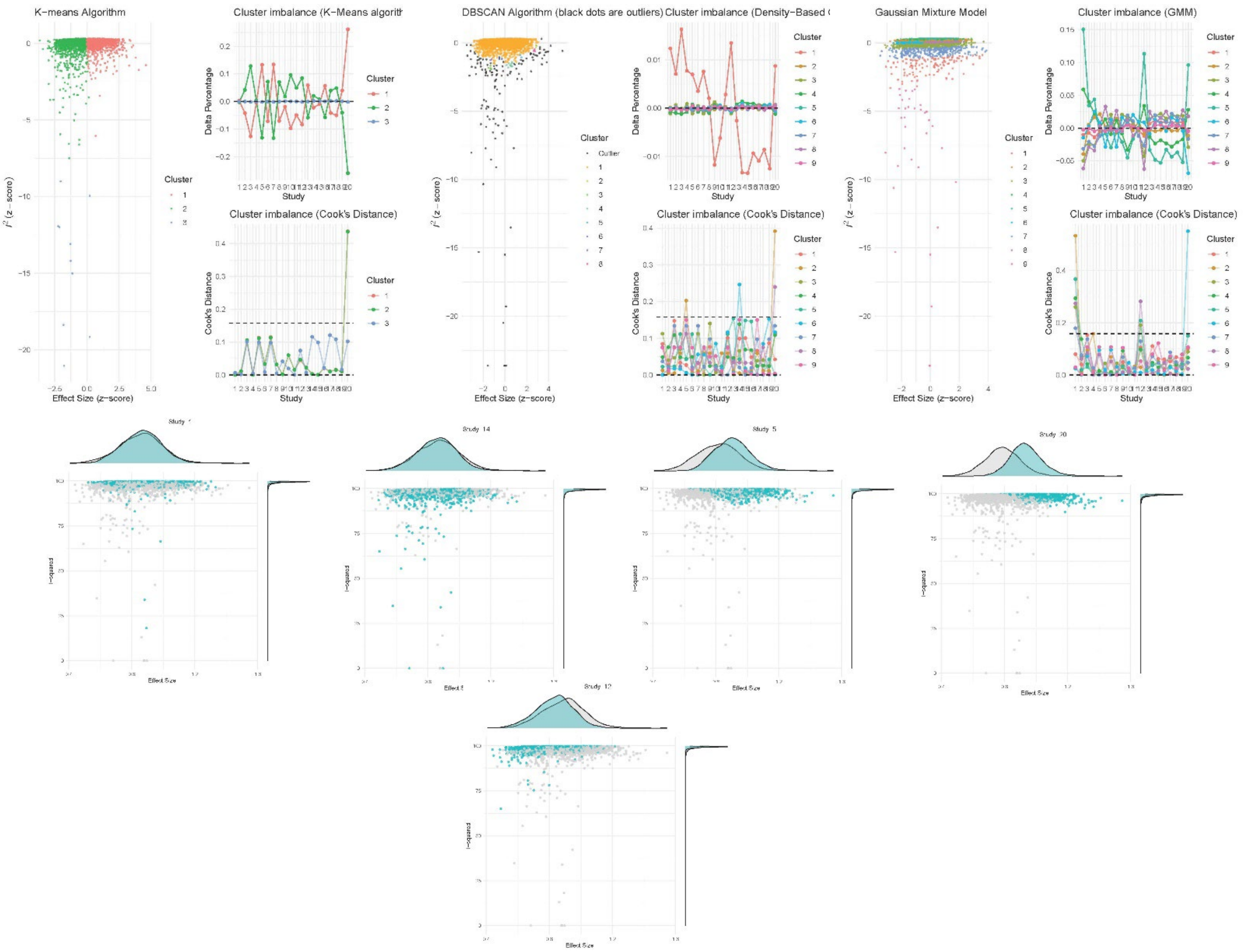

Figure 4: GOSH Plots and GOSH Plot Diagnostics.

Renal disorders

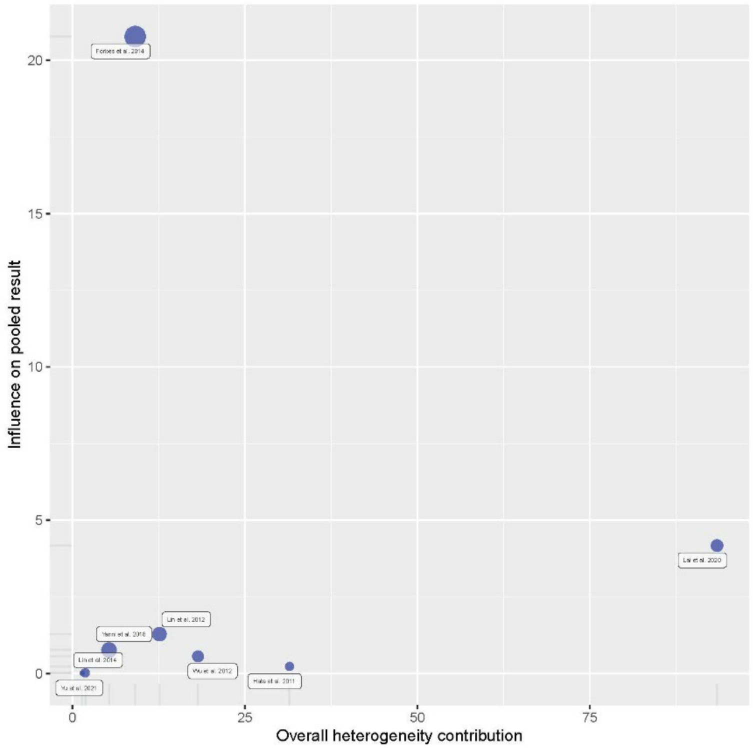

Figure 1: Baujat plot.

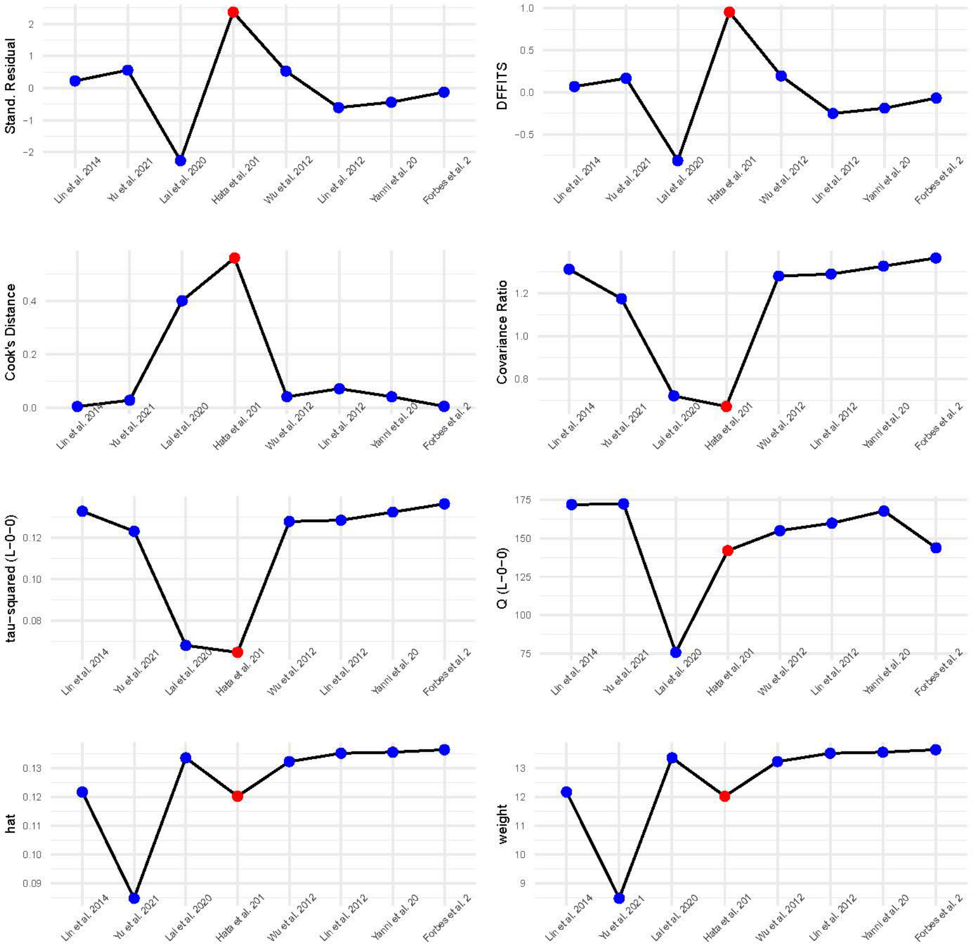

Figure 2: Influence diagnostics.

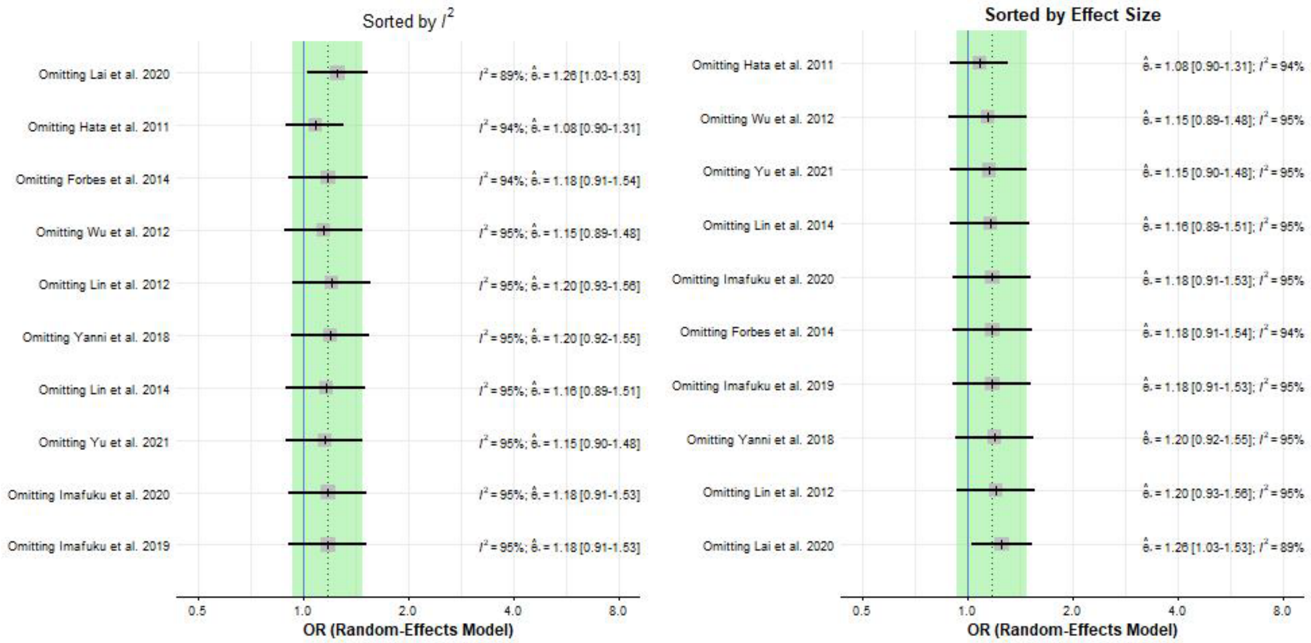

Figure 3: Forest plots for Leave-One-Out Analyses.

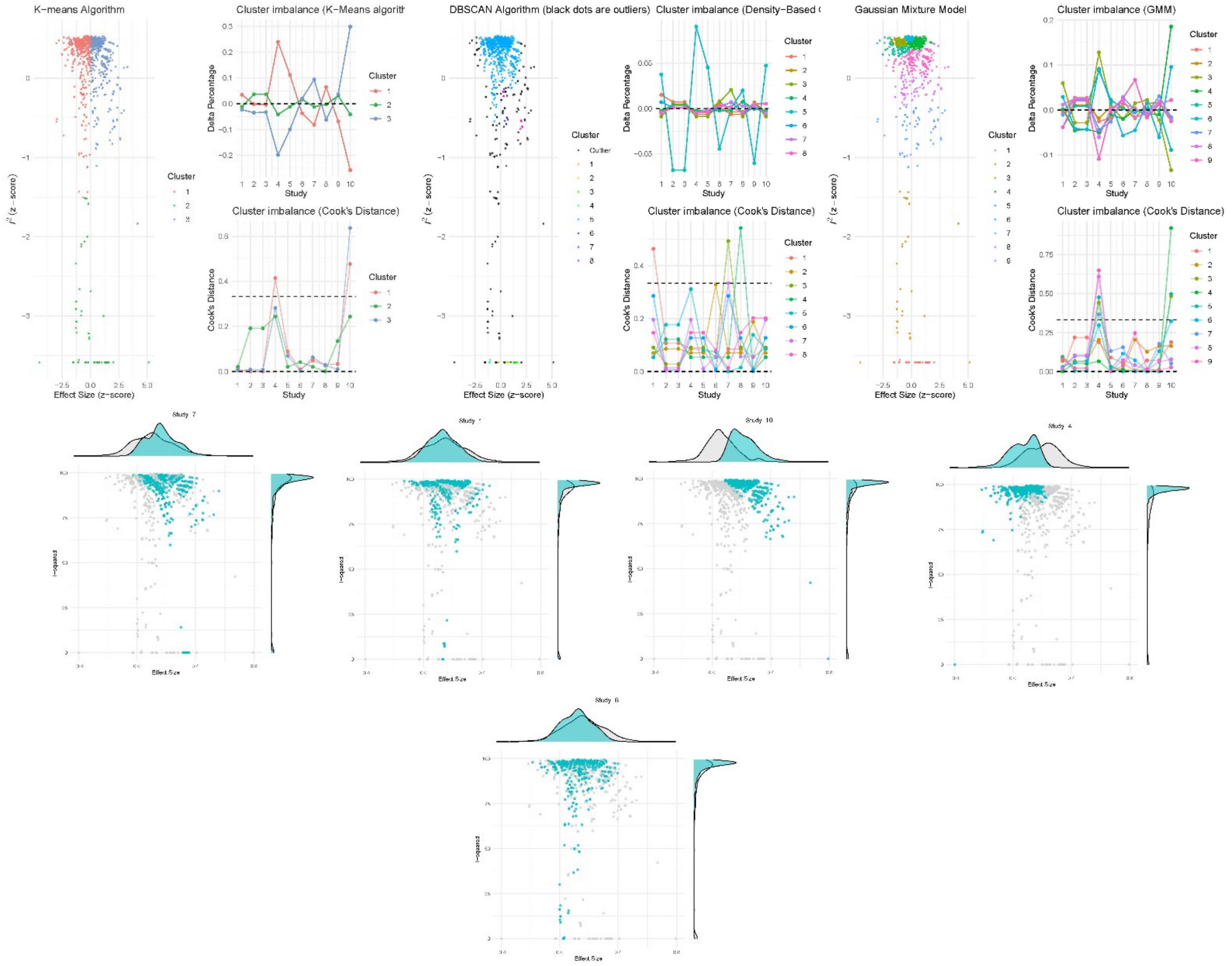

Figure 4: GOSH Plots and GOSH Plot Diagnostics.

Hematological disorders

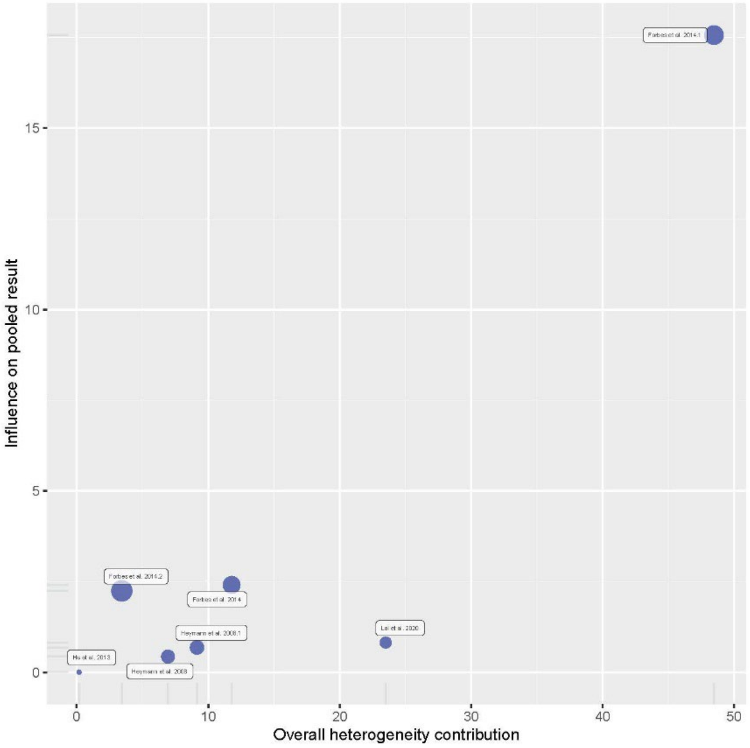

Figure 1: Baujat plot.

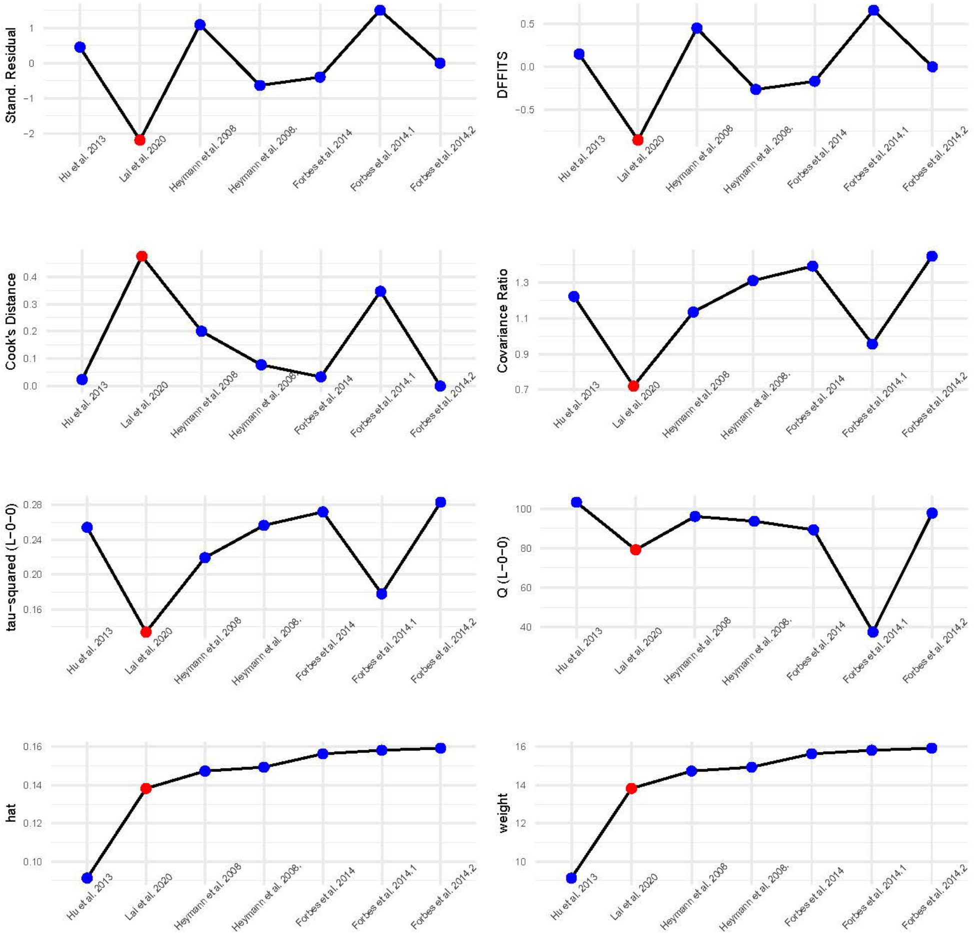

Figure 2: Influence diagnostics.

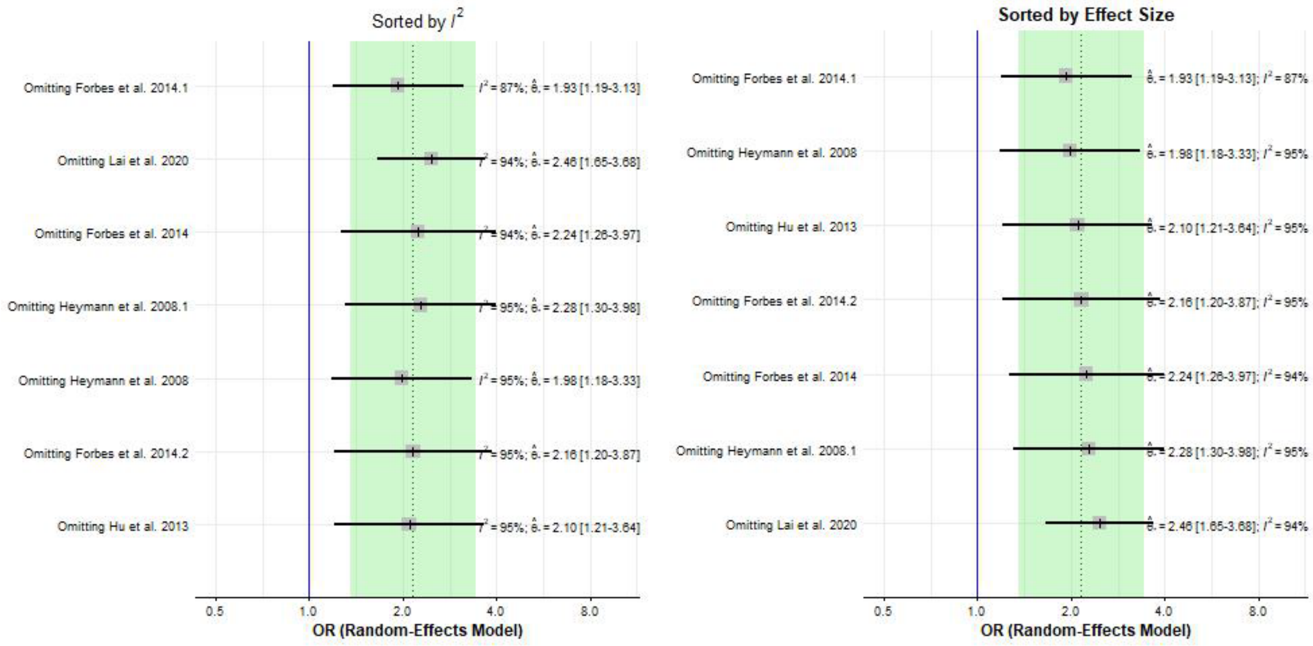

Figure 3: Forest plots for Leave-One-Out Analyses.

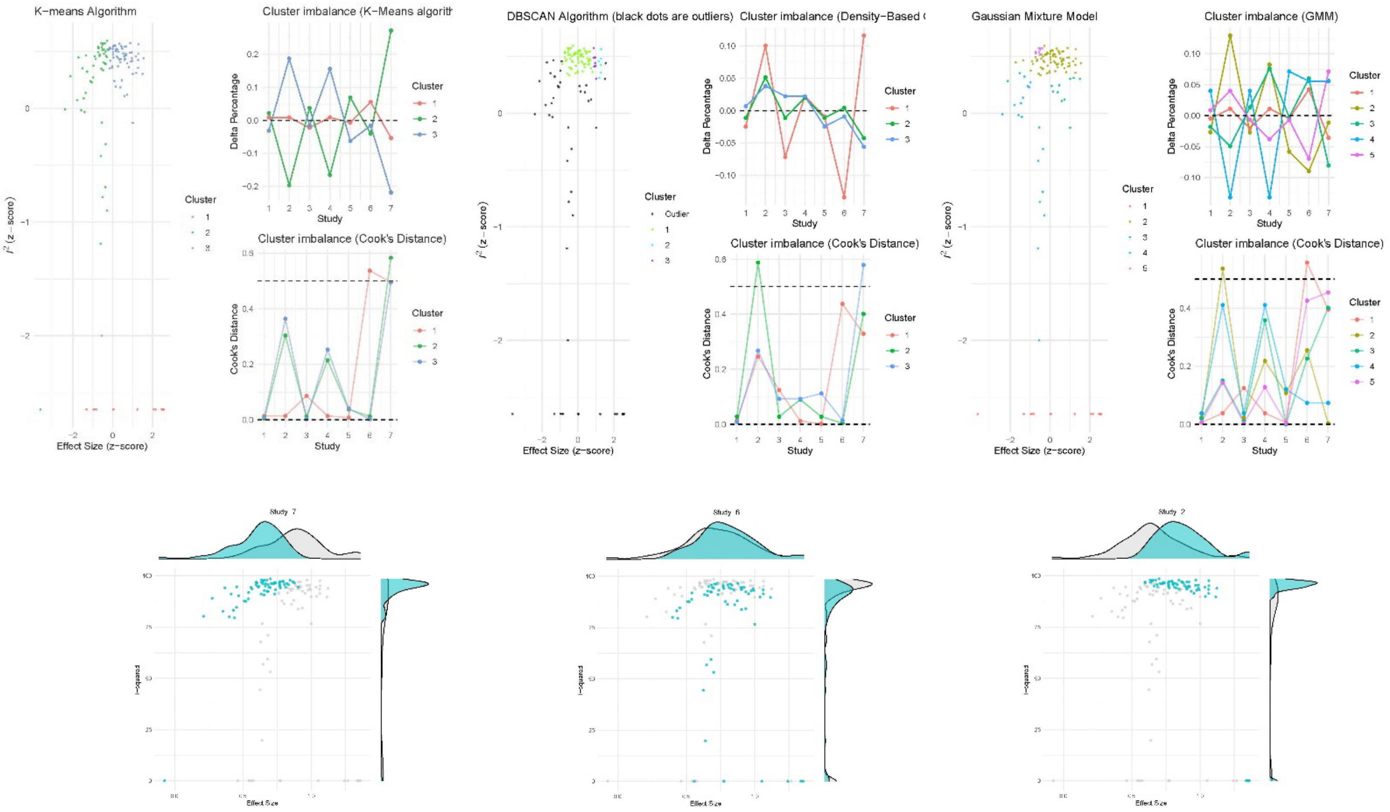

Figure 4: GOSH Plots and GOSH Plot Diagnostics.

Endocrine and metabolic disorders

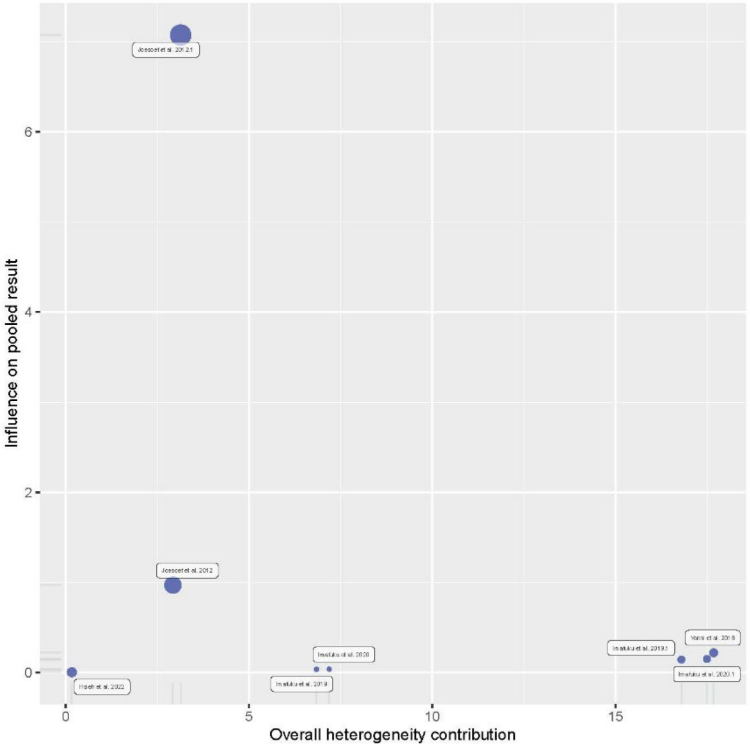

Figure 1: Baujat plot.

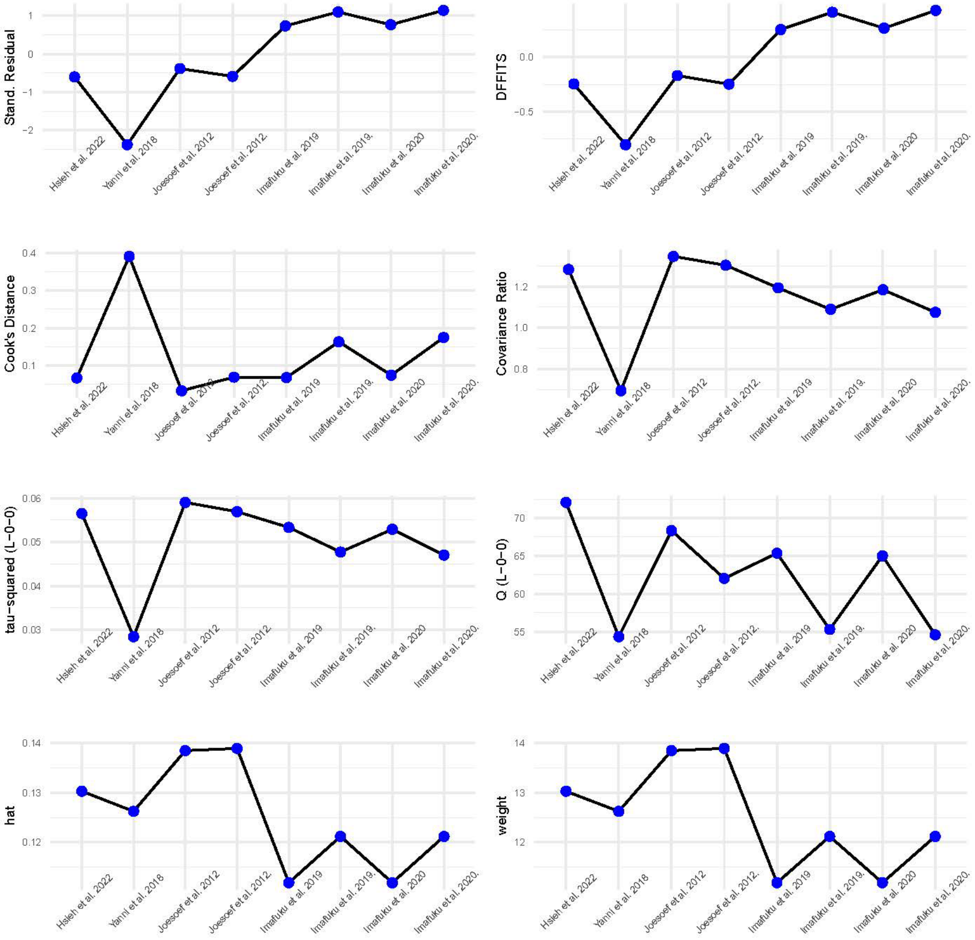

Figure 2: Influence diagnostics.

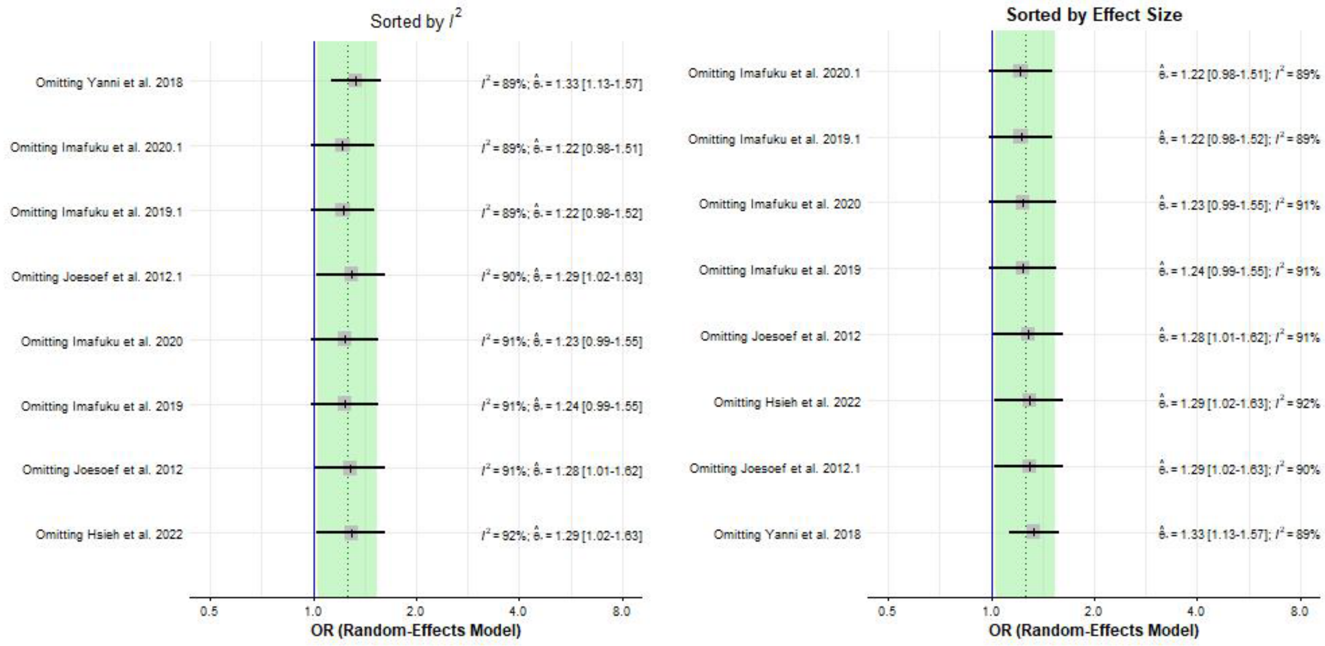

Figure 3: Forest plots for Leave-One-Out Analyses.

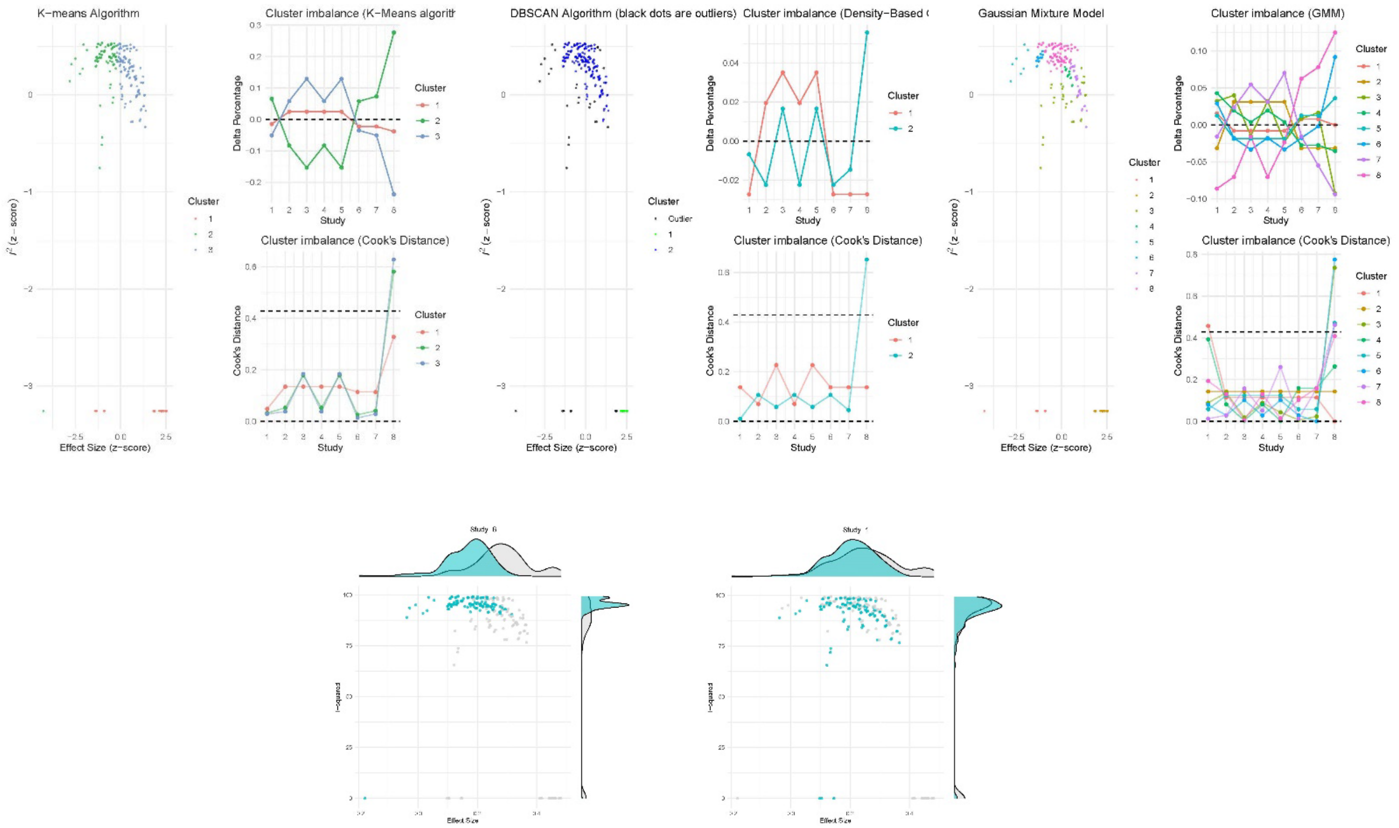

Figure 4: GOSH Plots and GOSH Plot Diagnostics.

Musculoskeletal disorders

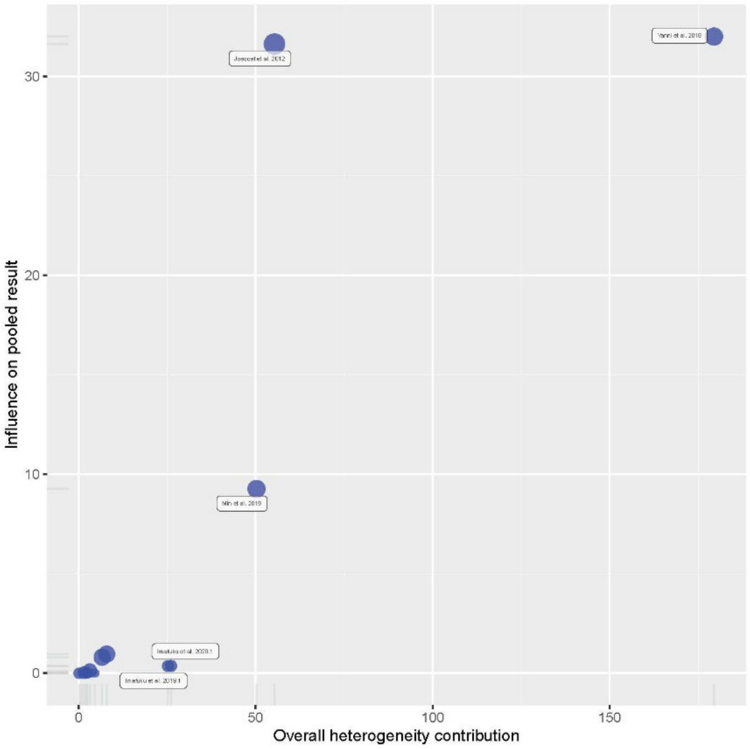

Figure 1: Baujat plot.

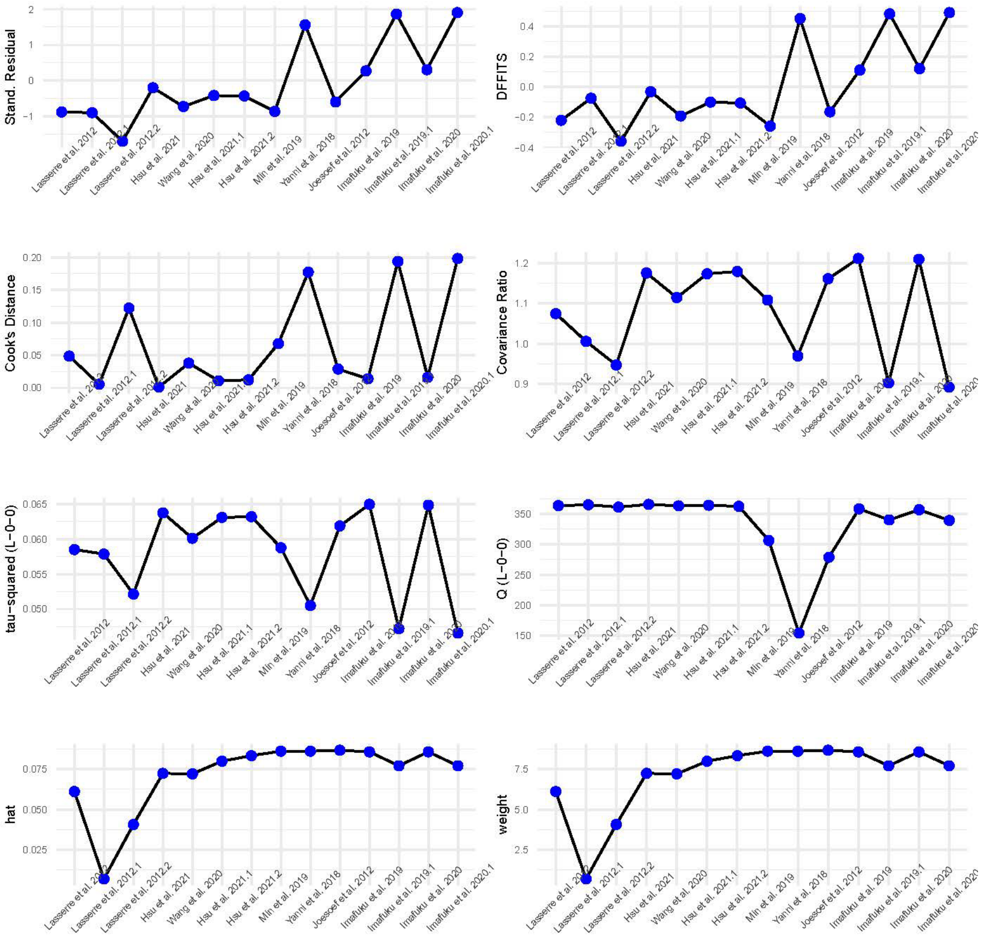

Figure 2: Influence diagnostics.

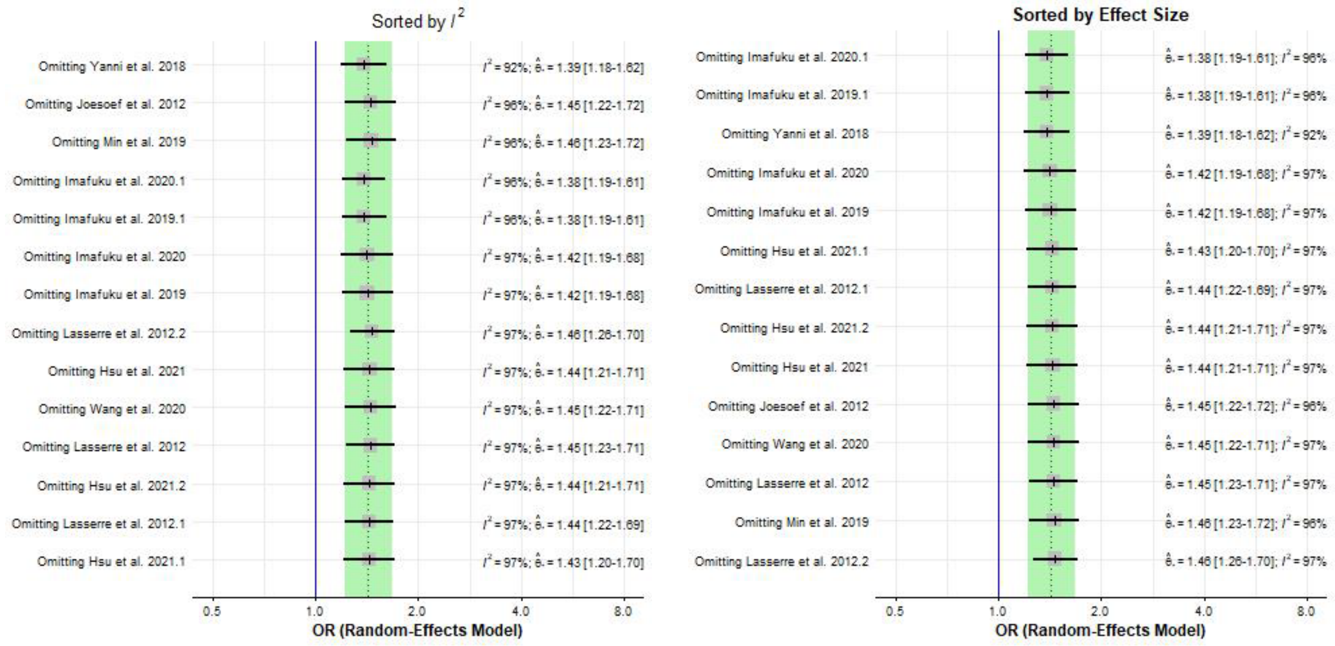

Figure 3: Forest plots for Leave-One-Out Analyses.

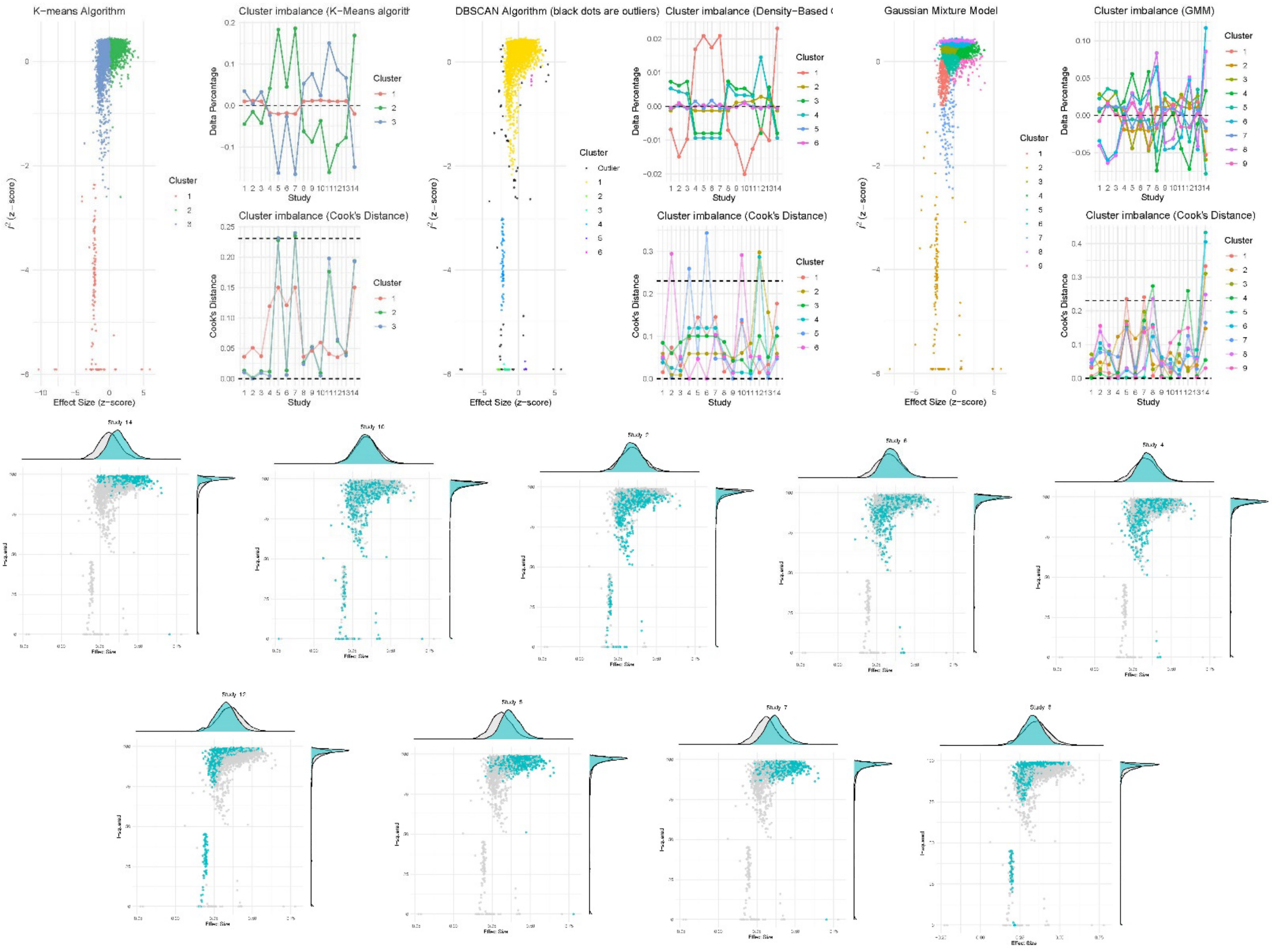

Figure 4: GOSH Plots and GOSH Plot Diagnostics.

Neurological disorders

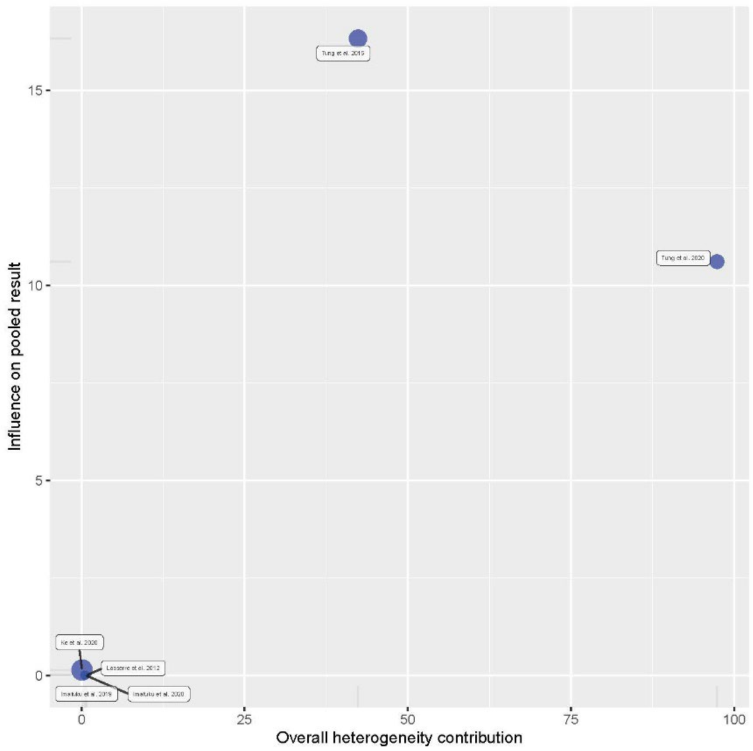

Figure 1: Baujat plot.

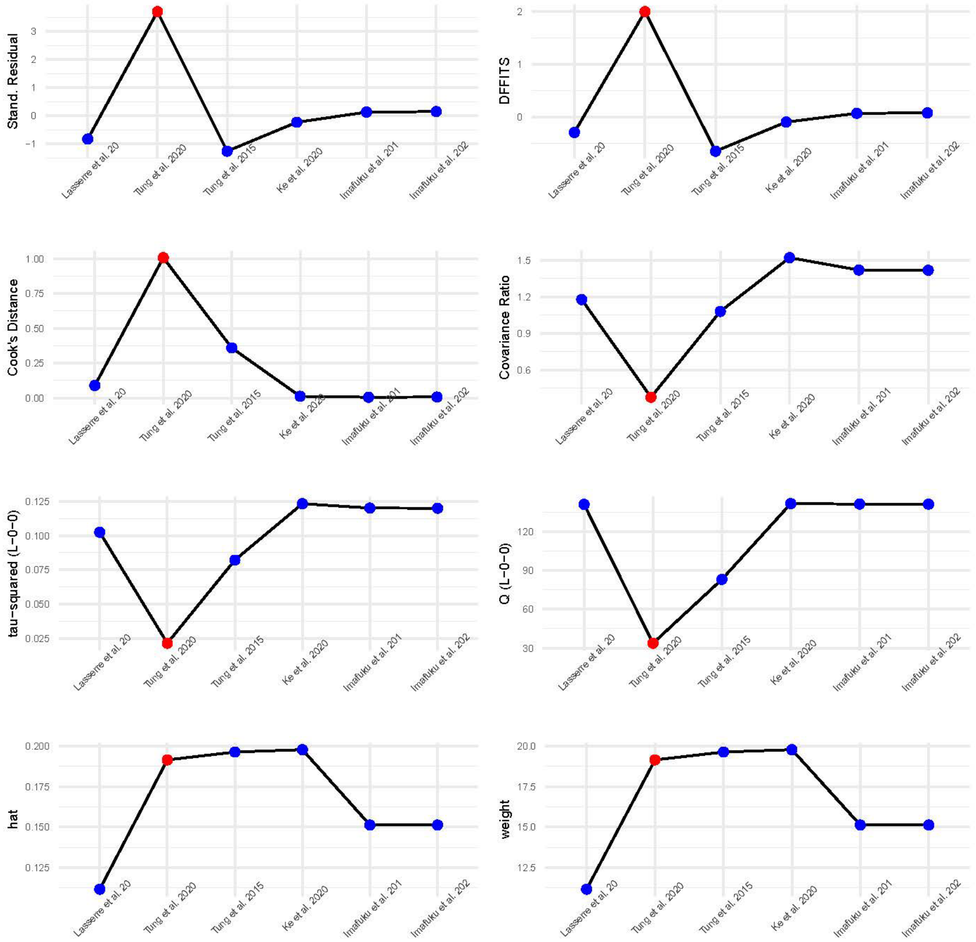

Figure 2: Influence diagnostics.

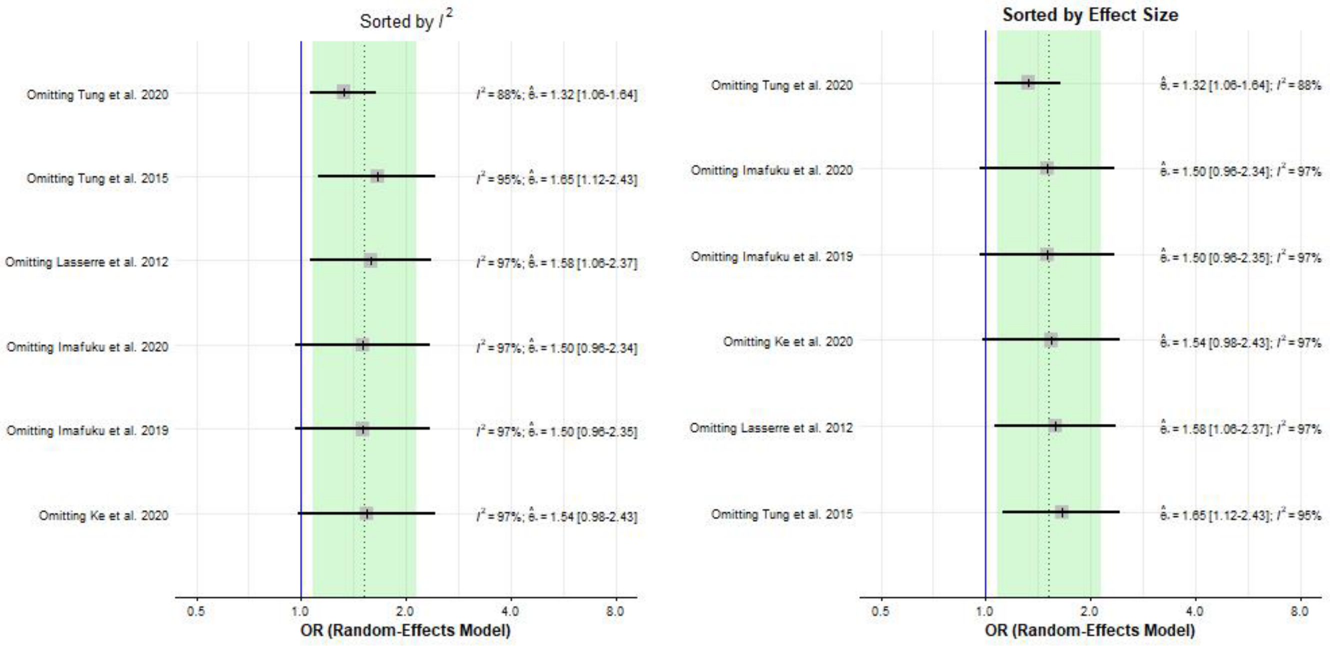

Figure 3: Forest plots for Leave-One-Out Analyses.

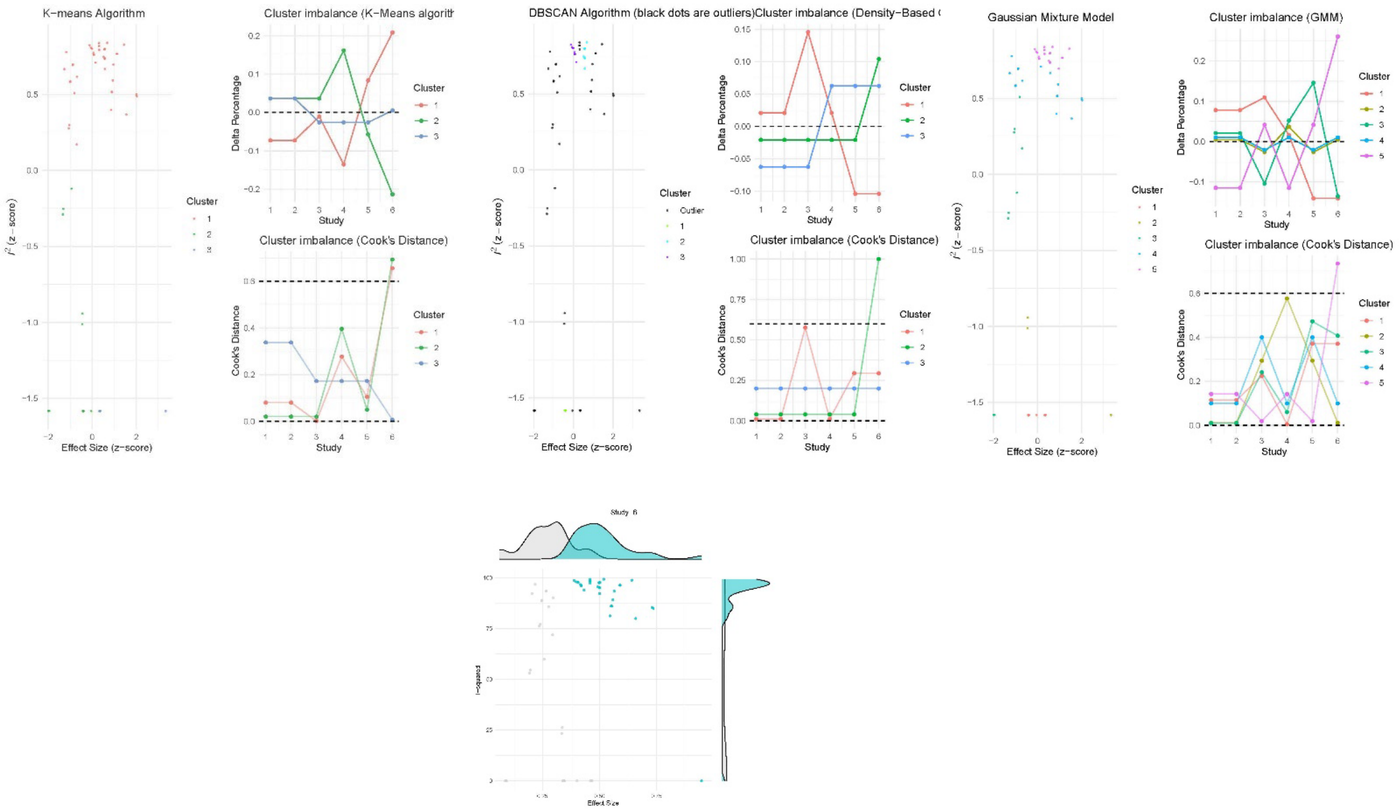

Figure 4: GOSH Plots and GOSH Plot Diagnostics.
