# Supplementary material for: Risk factors for herpes zoster infections: a systematic review and meta-analysis unveiling common trends and heterogeneity patterns
Source: Infection. 2024 Jan 18;52(3):1009–26. doi: 10.1007/s15010-023-02156-y (PMC11142967; doi:10.1007/s15010-023-02156-y)
Supplement: Supplementary file 8 — (PDF 190 kb) [file 15010_2023_2156_MOESM8_ESM.pdf]

S8. Meta-regression analyses.

Note: * show significance for R^2 (explained heterogeneity) at $p < 0.005$ before conducting permutation tests.

Moderator	R^2 (in %)	Estimate	SE	z-value	p-value (permutation)	95% CI
**Asthma**	0.00					
Year		-0.0429	0.0386	-1.1113	0.3860	[-0.119; 0.033]
Study design		0.2563	0.2992	0.8567	0.4290	[-0.330; 0.843]
Region		0.1699	0.1893	0.8971	0.4360	[-0.201; 0.541]
Sample Size		0.1495	0.1872	0.7981	0.4890	[-0.218; 0.517]
**Autoimmune disorders**	0.00					
Year		0.0346	0.0654	0.5294	0.5780	[-0.094; 0.163]
Study design		0.0549	0.7028	0.0781	0.9010	[-1.323; 1.432]
Region		-0.1387	0.2508	-0.5530	0.4740	[-0.630; 0.353]
Sample Size		0.1058	0.1734	0.6104	0.3780	[-0.234; 0.446]
**Cancer**	7.90					
Year		-0.0039	0.0327	-0.1188	0.9120	[-0.068; 0.060]
Study design		-0.1188	0.3913	-0.3037	0.7510	[-0.886; 0.648]
Region		0.2599	0.1576	1.6485	0.1160	[-0.049; 0.569]
Sample Size		0.0212	0.1121	0.1894	0.8320	[-0.199; 0.241]
**Cardiovascular disorders**	72.78					
Year		-0.0824	0.0335	-2.4632	0.0620	[-0.148; -0.017]
Study design		-0.2298	0.2791	-0.8234	0.4340	[-0.777; 0.317]
Region		0.2996	0.1781	1.6821	0.1680	[-0.050; 0.649]
Sample Size		0.3226	0.2456	1.3135	0.2590	[-0.159; 0.804]
**CHF**	71.88					
Year		-0.1585*	0.0569	-2.7869	0.1020	[-0.270; -0.047]
Study design		0.0121	0.3601	0.0337	0.9760	[-0.694; 0.718]
Region		0.5151	0.2715	1.8974	0.2100	[-0.017; 1.047]
Sample Size		0.5677	0.3567	1.5917	0.2600	[-0.131; 1.267]
**COPD**	45.22					
Year		-0.0789	0.0542	-1.4558	0.2050	[-0.185; 0.027]
Study design		0.1165	0.4007	0.2907	0.8220	[-0.669; 0.902]
Region		0.2882	0.1740	1.6567	0.1420	[-0.053; 0.629]
Sample Size		0.6826*	0.2345	2.9112	0.0240	[0.223; 1.142]
**Depression**	0.00					
Year		-0.0357	0.0593	-0.6018	0.5600	[-0.152; 0.081]
Study design		-0.1239	0.7354	-0.1685	0.8560	[-1.565; 1.318]
Region		0.0366	0.2247	0.1627	0.8820	[-0.404; 0.477]
Sample Size		-0.0579	0.3488	-0.1659	0.9130	[-0.741; 0.626]
**Diabetes**	0.00					
Year		-0.0462	0.0356	-1.2994	0.2210	[-0.116; 0.024]
Study design		-0.1820	0.3041	-0.5985	0.5490	[-0.778; 0.414]
Region		-0.0934	0.1087	-0.8593	0.4430	[-0.306; 0.120]
Sample Size		0.0291	0.1134	0.2563	0.7780	[-0.193; 0.251]
**Digestive disorders**	0.00					
Year		-0.0322	0.0562	-0.5732	0.6140	[-0.142; 0.078]
Study design		0.3965	0.8023	0.4941	0.6100	[-1.176; 1.969]
Region		-0.0185	0.1660	-0.1117	0.9140	[-0.344; 0.307]
Sample Size		0.0994	0.1372	0.7250	0.4970	[-0.169; 0.368]

Moderator	R² (in %)	Estimate	SE	z-value	p-value (permutation)	95% CI
**Endocrine and metabolic disorders**	98.63					
Year		0.0073	0.1000	0.0727	0.9220	[-0.189; 0.203]
Study design		0.1773	0.7704	0.2301	0.8010	[-1.333; 1.687]
Region		0.1646	0.1791	0.9189	0.4010	[-0.187; 0.516]
Sample Size		0.1686	0.1183	1.4257	0.2380	[-0.063; 0.401]
**Hematological disorders**	10.36					
Year		0.0591	0.2202	0.2683	0.8119	[-0.372; 0.491]
Study design		1.5403	2.1824	0.7058	0.5214	[-2.737; 5.818]
Region		0.1043	0.4373	0.2386	0.8333	[-0.753; 0.961]
Sample Size		-	-	-	-	-
**HIV**	32.61					
Year		0.0346	0.0415	0.8328	0.4610	[-0.047; 0.116]
Study design		0.6169	0.4198	1.4694	0.2200	[-0.206; 1.440]
Region		-0.2086	0.1560	-1.3371	0.2830	[-0.514; 0.097]
Sample Size		0.2127	0.1526	1.3940	0.2240	[-0.086; 0.512]
**IBD**	53.24					
Year		-0.2714*	0.0804	-3.3739	0.1160	[-0.429; -0.114]
Study design		-0.7395	0.5532	-1.3366	0.3360	[-1.824; 0.345]
Region		0.3978	0.2489	1.5980	0.2060	[-0.090; 0.886]
Sample Size		-0.2939	0.1820	-1.6152	0.2800	[-0.651; 0.063]
**Mental health condition**	41.97					
Year		-0.2908*	0.0827	-3.5146	0.0420	[-0.453; -0.129]
Study design		-1.0695	0.5202	-2.0559	0.1000	[-2.089; -0.050]
Region		-0.6516	0.3265	-1.9955	0.0650	[-1.292; -0.012]
Sample Size		-0.0480	0.2251	-0.2132	0.8300	[-0.490; 0.393]
**Musculoskeletal disorders**	62.94					
Year		0.0520	0.0272	1.9098	0.0790	[-0.001; 0.105]
Study design		-0.0195	0.1836	-0.1064	0.9310	[-0.379; 0.340]
Region		-0.2011	0.1125	-1.7876	0.1110	[-0.422; 0.019]
Sample Size		0.1819	0.0922	1.9723	0.0800	[0.001; 0.363]
**Neurological disorders**	0.00					
Year		0.1075	0.0777	1.3831	0.3042	[0.045; 0.260]
Study design		0.3051	0.6732	0.4532	0.6500	[-1.014; 1.625]
Region		-	-	-	-	-
Sample Size		-0.0648	0.1716	-0.3775	0.7931	[-0.401; 0.272]
**Psoriasis**	100.00					
Year		-0.0440	0.0467	-0.9404	0.4903	[-0.136; 0.048]
Study design		0.0792	0.1433	0.5524	0.6944	[-0.202; 0.360]
Region		0.0814	0.0357	2.2821	0.2278	[0.012; 0.151]
Sample Size		0.0381	0.0807	0.4727	0.6819	[-0.120; 0.196]
**Rheumatoid arthritis**	1.83					
Year		-0.0274	0.0210	-1.3035	0.2200	[-0.069; 0.014]
Study design		-0.3512	0.2860	-1.2279	0.2300	[-0.912; 0.209]
Region		-0.0413	0.1345	-0.3066	0.7430	[-0.305; 0.223]
Sample Size		0.1109	0.1058	1.0480	0.2950	[-0.097; 0.318]

Moderator	R^2 (in %)	Estimate	SE	z-value	p-value (permutation)	95% CI
**Renal disorders**	11.95					
Year		-0.0675	0.0347	-1.9438	0.1210	[-0.136; 0.001]
Study design		-0.2832	0.2459	-1.1516	0.3250	[-0.765; 0.199]
Region		0.0091	0.1210	0.0755	0.9310	[-0.228; 0.246]
Sample Size		0.0491	0.1362	0.3602	0.7620	[-0.218; 0.316]
**Systemic lupus erythematosus**	55.16					
Year		0.0499	0.0368	1.3565	0.2720	[-0.022; 0.122]
Study design		0.3276	0.4652	0.7043	0.4980	[-0.584; 1.239]
Region		0.4796*	0.1867	2.5685	0.0450	[0.114; 0.846]
Sample Size		0.1173	0.1233	0.9517	0.3920	[-0.124; 0.359]
**Transplantation**	0.00					
Year		0.0924	0.0803	1.1498	0.3260	[-0.065; 0.250]
Study design		1.2534	1.6464	0.7613	0.4670	[-1.973; 4.480]
Region		-0.0007	0.5025	-0.0014	0.9980	[-0.986; 0.984]
Sample Size		0.0686	0.4462	0.1537	0.8930	[-0.806; 0.943]
