# Supplementary material for: Risk factors for herpes zoster infections: a systematic review and meta-analysis unveiling common trends and heterogeneity patterns
Source: Infection. 2024 Jan 18;52(3):1009–26. doi: 10.1007/s15010-023-02156-y (PMC11142967; doi:10.1007/s15010-023-02156-y)

S9. Correlation Matrices for each outcome.

Asthma

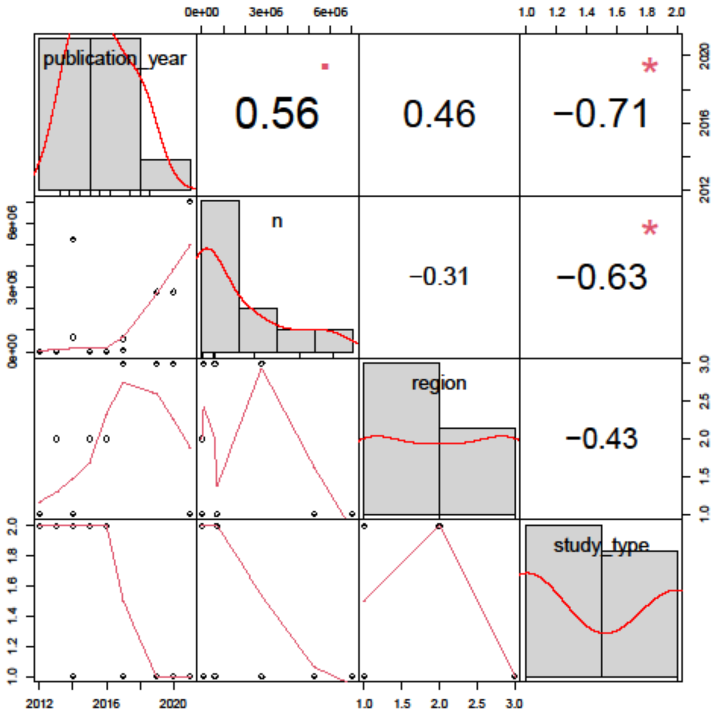

Autoimmune disorders

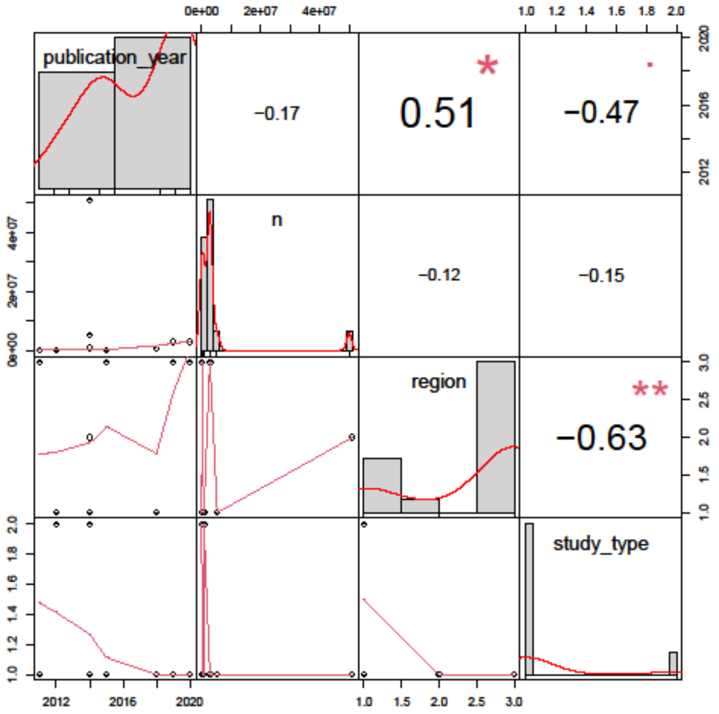

Cancer

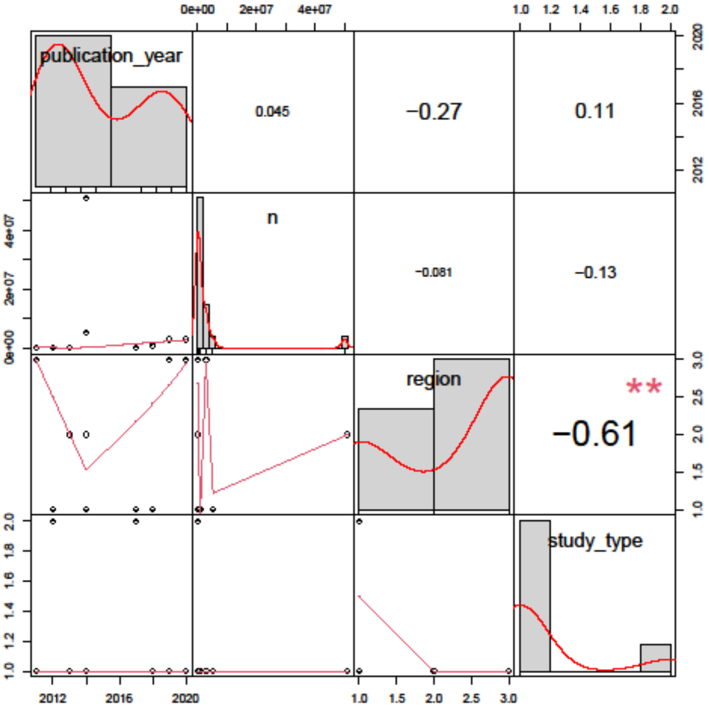

Cardiovascular disorders

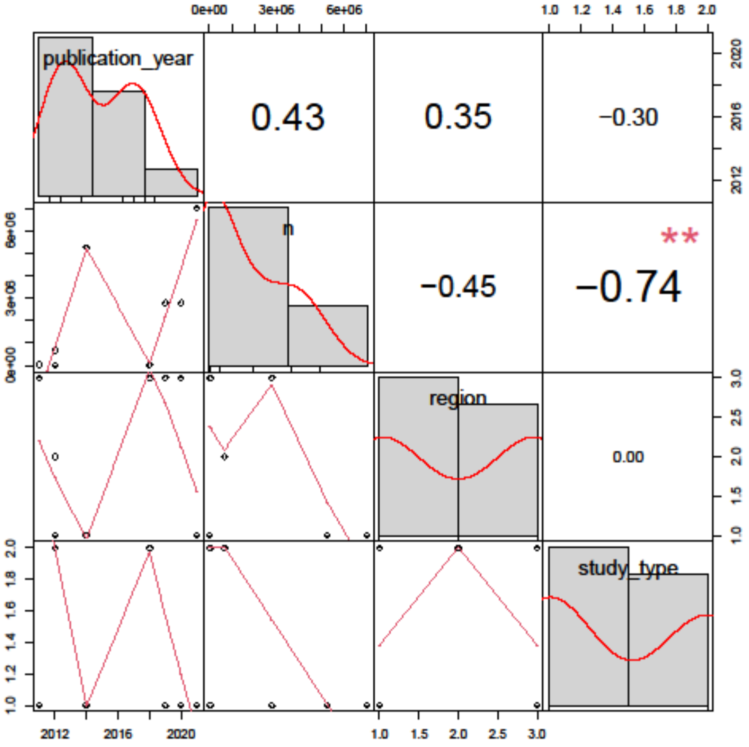

CHF

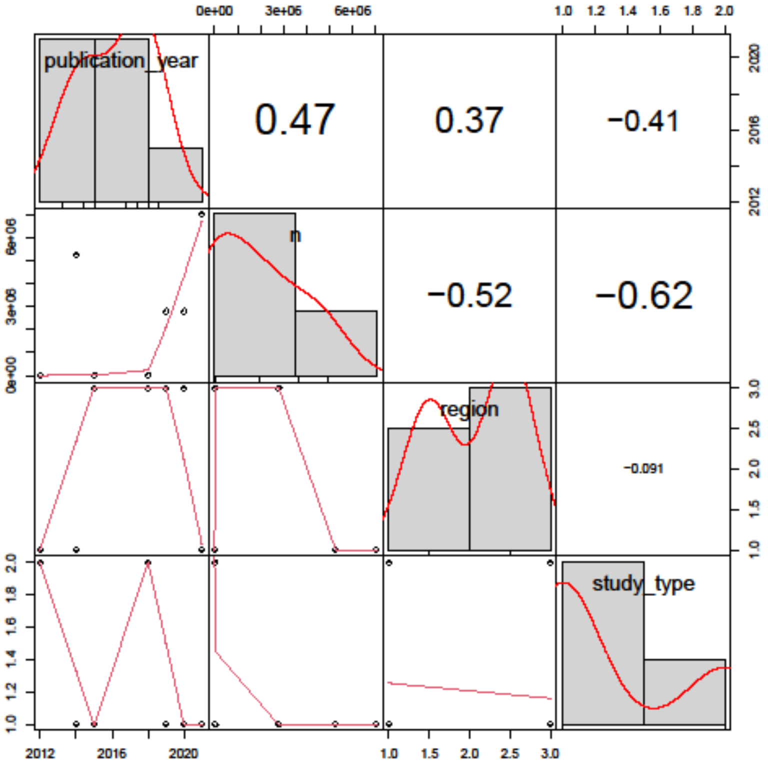
publication_year
n
region
study_type
0.47
0.37
−0.41
−0.52
−0.62
−0.091

COPD

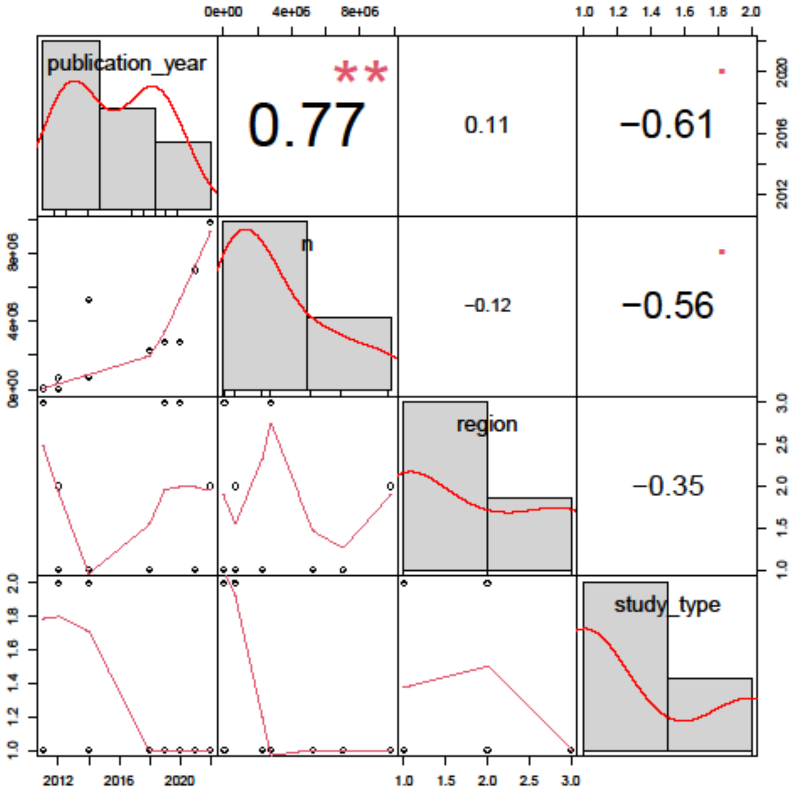
publication_year
n
region
study_type
0.77
0.11
−0.61
−0.12
−0.56
−0.35

Depression

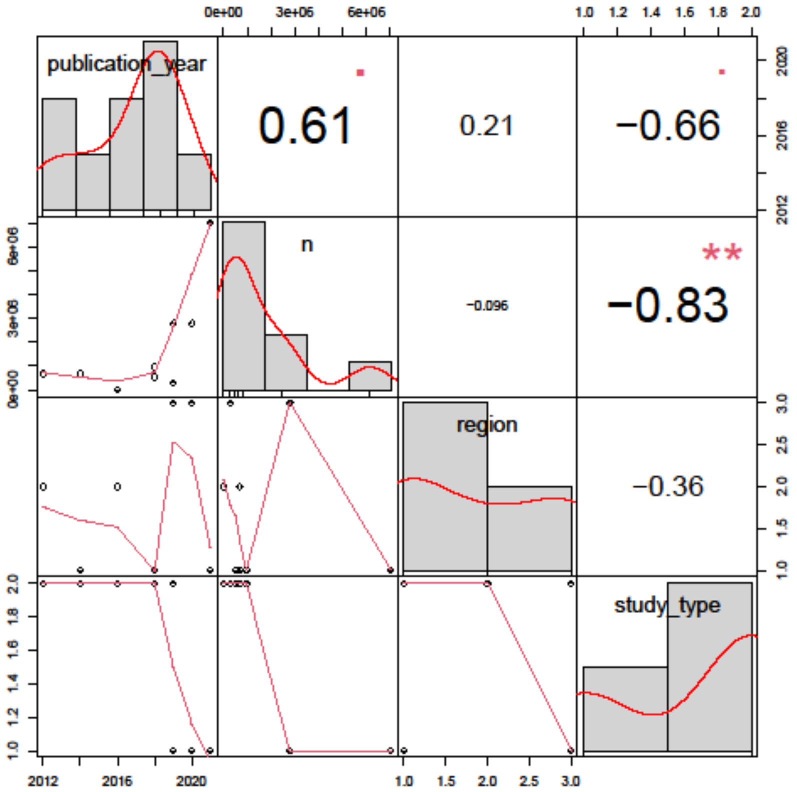

Diabetes

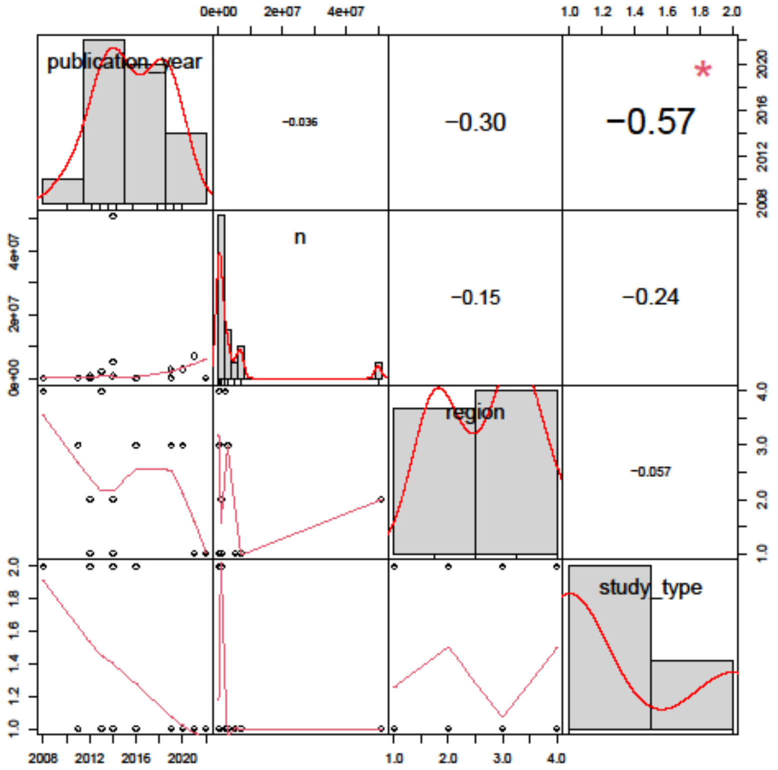

Digestive disorders

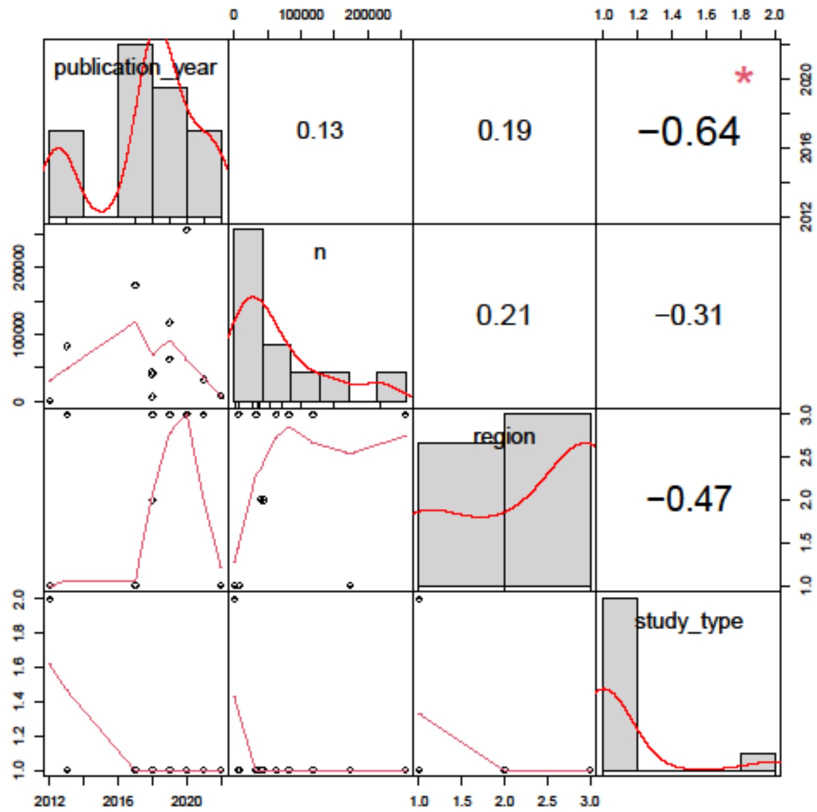

Endocrine and metabolic disorders

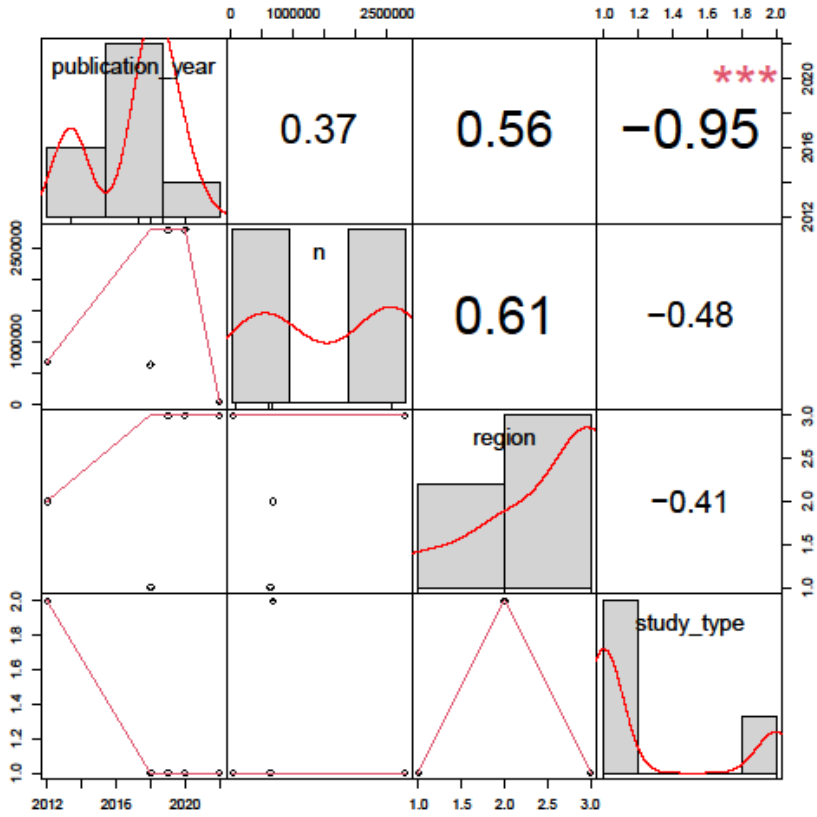

Hematological disorders

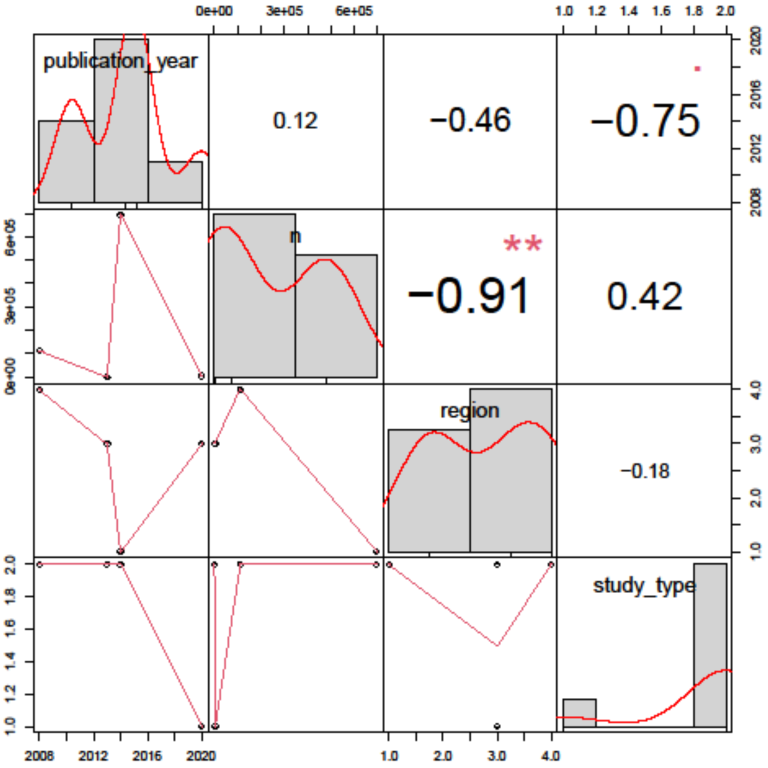

HIV

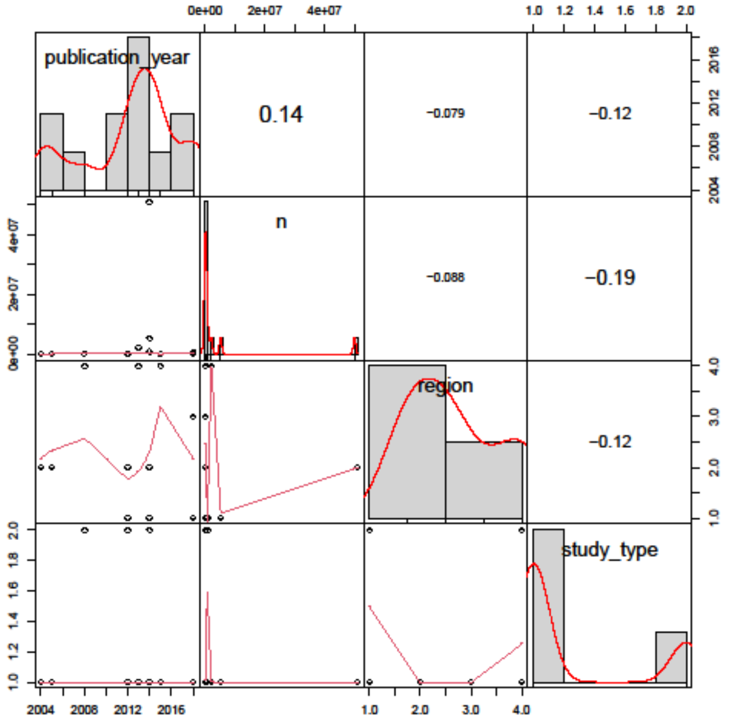

IBD

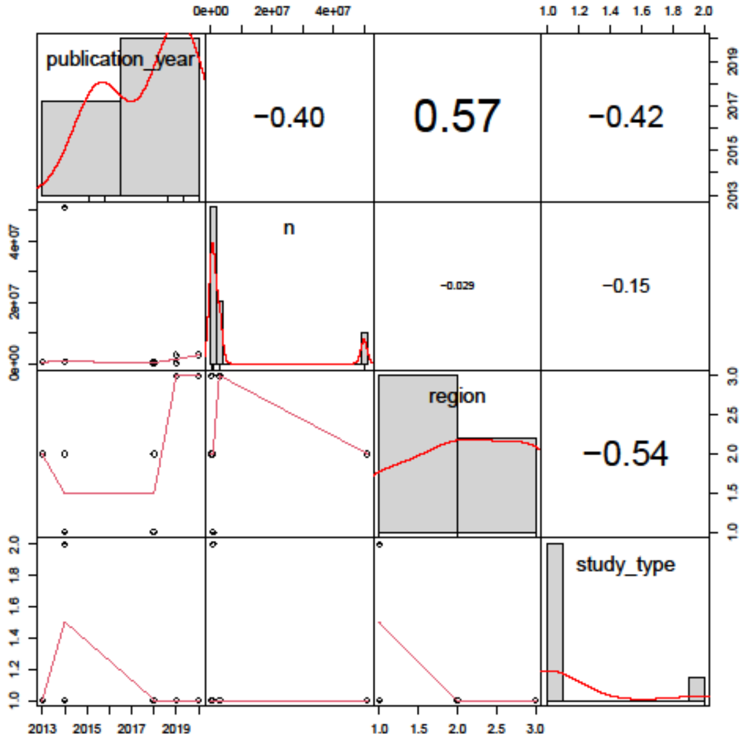

Mental health condition

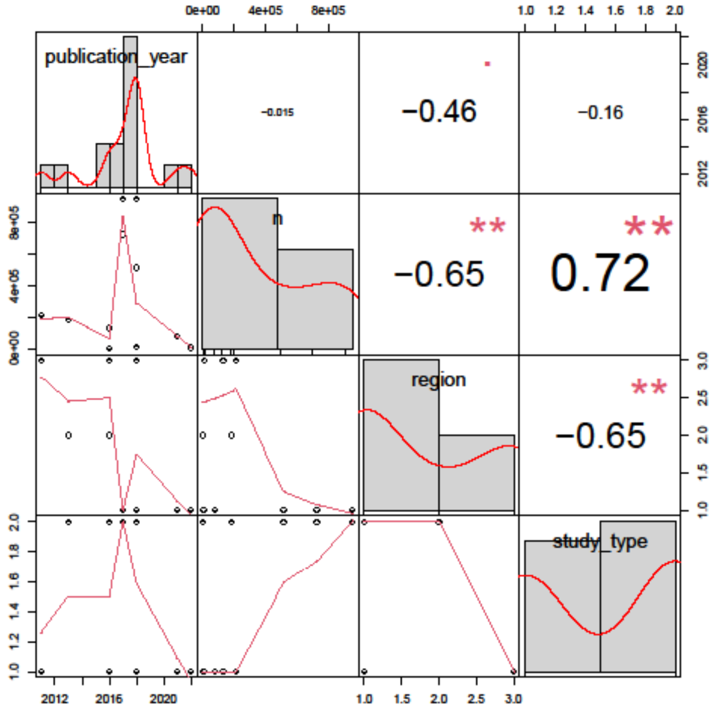

Musculoskeletal disorders

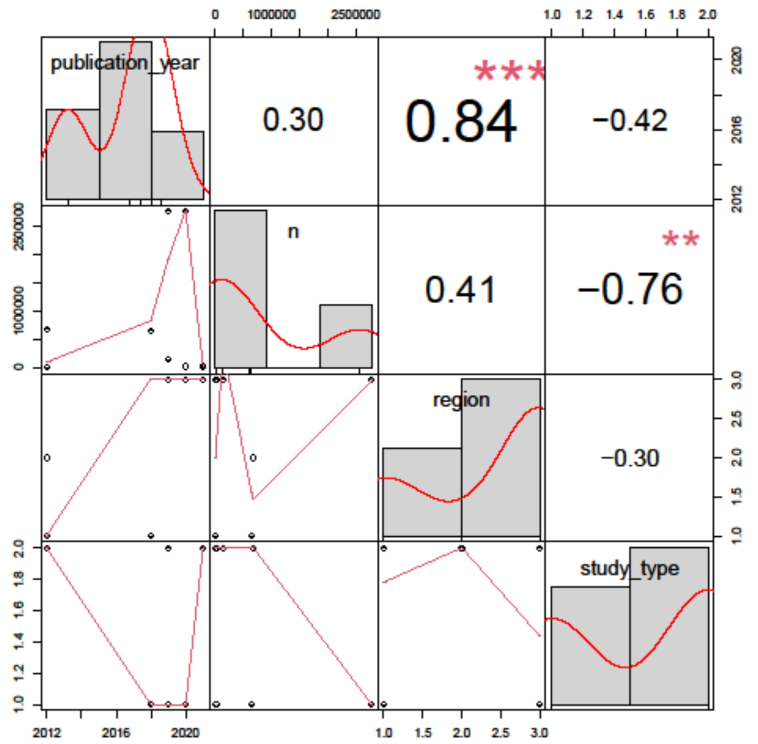

Neurological disorders

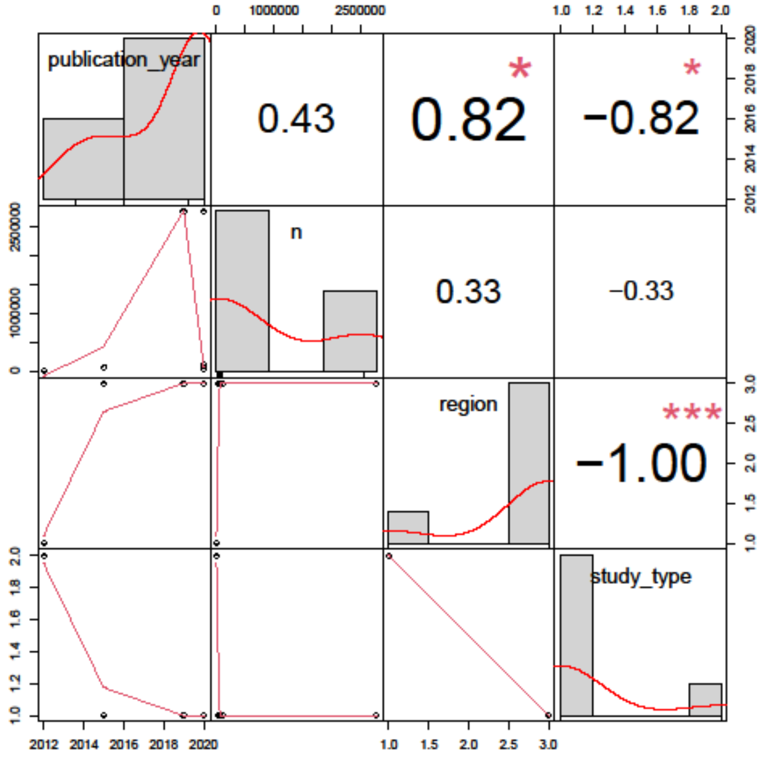

Psoriasis

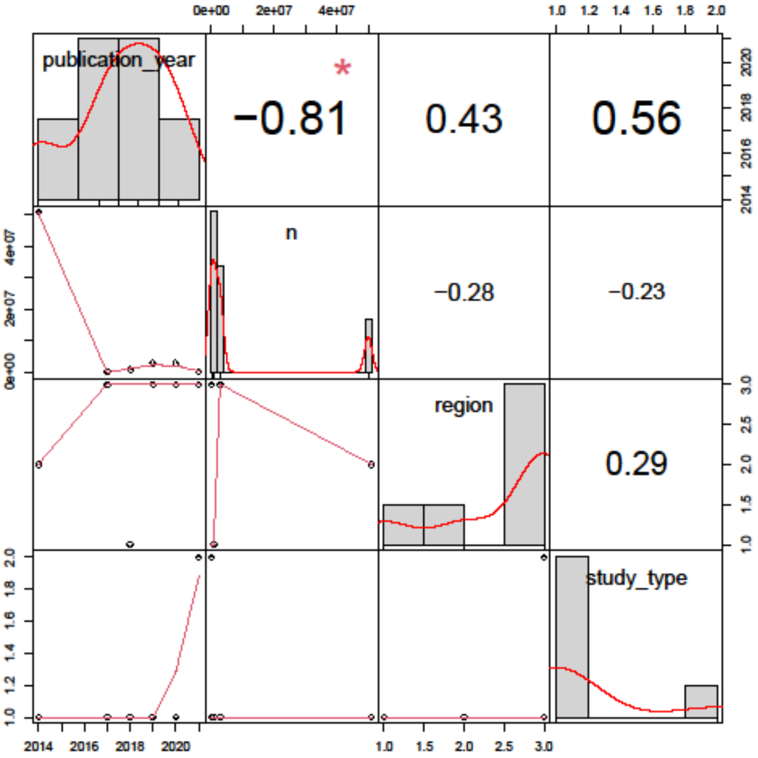

Renal disorders

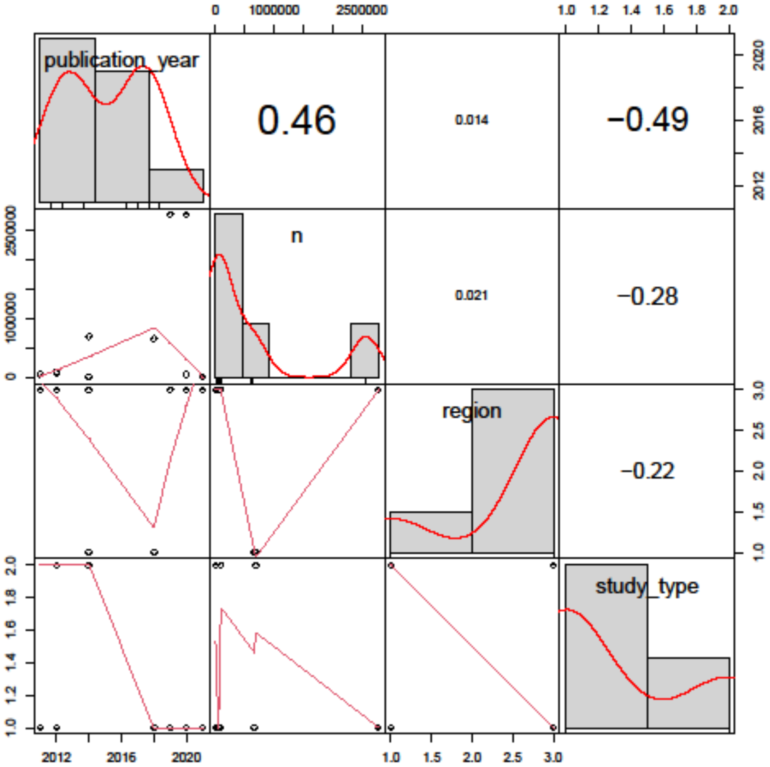

RA

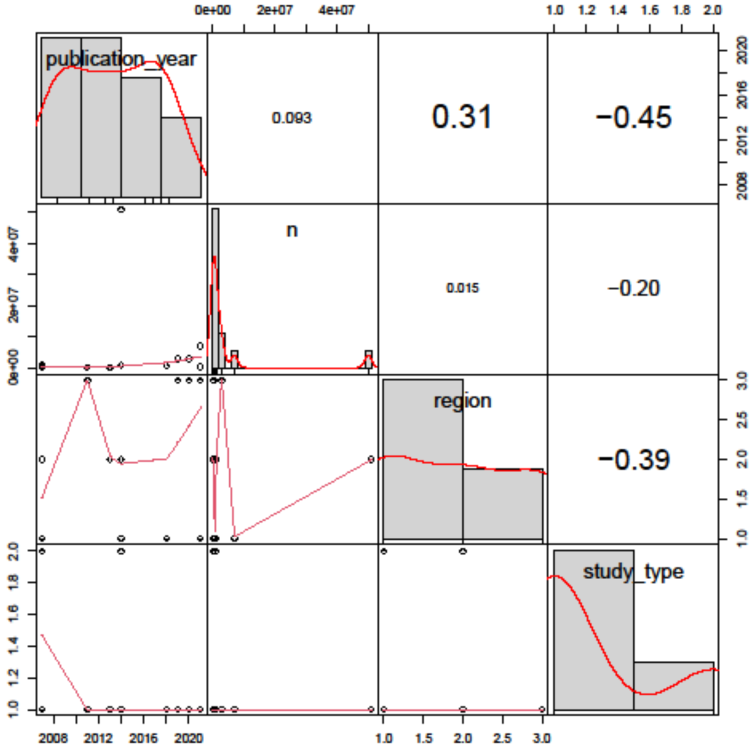
publication_year
n
region
study_type
0.093
0.31
−0.45
0.015
−0.20
−0.39

SLE

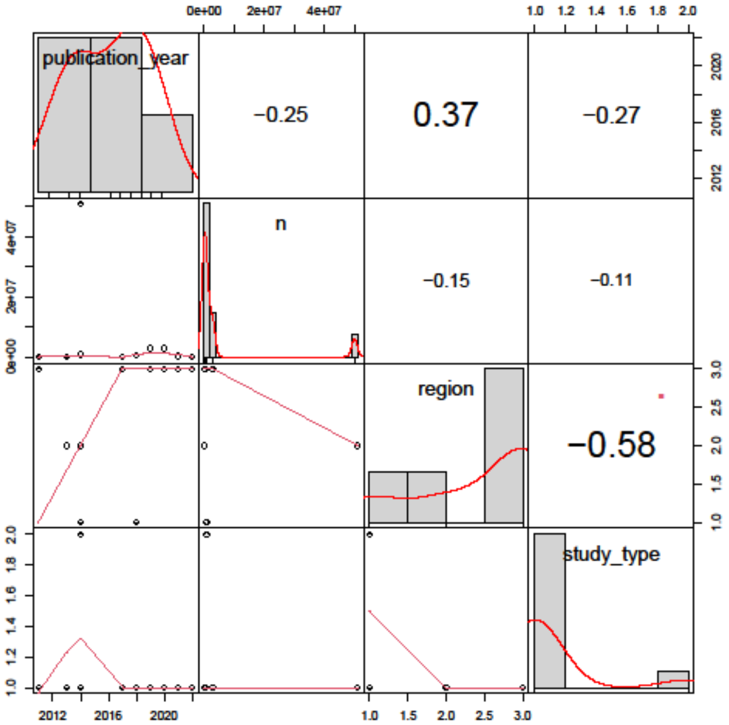
publication_year
n
region
study_type
−0.25
0.37
−0.27
−0.15
−0.11
−0.58

Transplantation

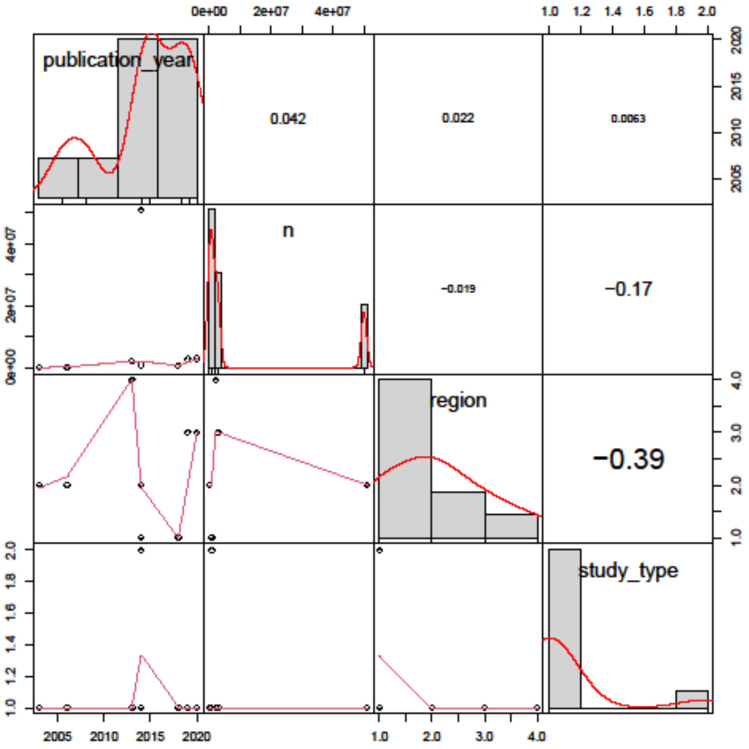

Supplement: Supplementary file 9 — (PDF 693 kb) [file 15010_2023_2156_MOESM9_ESM.pdf]
